# Decomposing the global burden of cancers: quantifying the contributions of disease severity changes across regions and time (1990–2021)

**DOI:** 10.7189/jogh.15.04161

**Published:** 2025-06-02

**Authors:** Zijian Qiu, Yitong Huang, Zhaoqi Qiu, Zeying Guo, Liejiong Wang, Maoyi Xu, Feng Xuan

**Affiliations:** 1 Department of Radiation Oncology, The Quzhou Affiliated Hospital of Wenzhou Medical University, Quzhou People’s Hospital, Quzhou, China; 2 Department of Internal Medicine, Zhuji Maternal and Child Health Hospital, Shaoxing, China; 3 Department of Radiation Oncology, Zhuji Affiliated Hospital of Wenzhou Medical University, Shaoxing, China; 4 Department of Medical Oncology, Zhuji Affiliated Hospital of Wenzhou Medical University, Shaoxing, China; 5 Department of Medical Oncology, The Affiliated Hospital of Jiaxing University, Jiaxing, China

## Abstract

**Background:**

Cancer poses a significant challenge to society and public health. Our objective was to systematically quantify the effect of disease severity changes on cancer-related disability-adjusted life years (DALYs), thereby enabling cross-regional, temporal, and cancer-type comparisons.

**Methods:**

This secondary analysis obtained data on prevalence, DALYs, and population size from 1990 to 2021 from the Global Burden of Disease 2021. Unlike conventional analyses, a four-factor decomposition analysis was applied to attribute DALY changes to population growth, aging, prevalence shifts, and disease severity changes from 1990 to each subsequent year. We focused on the absolute and relative contributions of disease severity changes to DALY variations. Comparative analyses were subsequently stratified by region, sociodemographic index (SDI), sex, and cancer type.

**Results:**

Between 1990 and 2021, a reduction in neoplasms-related DALYs attributed to disease severity changes was observed globally for both sexes combined, with a total decrease of 81 963.31 thousand (52.68%) by 2021. Significantly, males (n = −56 172.28 thousand; −62.73%) exhibited greater absolute and relative contributions than females (n = −28 558.95 thousand; −42.35%). High (n = −30 317.14 thousand; −67.01%) and middle SDI (n = −32 958.22 thousand; −80.36%) regions showed a more pronounced decline than low-middle SDI (n = −5869.18 thousand; −37.48%) and low SDI (n = −2881.96 thousand; −41.62%) regions. Nationally, China (n = −44 306.96 thousand) demonstrated the largest absolute contribution, while Qatar (−337.34%) exhibited the highest relative proportion. Globally, absolute contributions were greatest for lung (n = −12 228.72 thousand), colorectal (n = −9334.10 thousand), and gastric (n = −8504.91 thousand) cancers. The highest relative proportions were for testicular (−80.81%), thyroid (−75.28%), and nasopharyngeal (−75.11%) cancers. Notably, mesothelioma saw an increase in DALYs due to disease severity changes.

**Conclusions:**

Variations in cancer-related DALYs attributed to disease severity changes exhibited substantial heterogeneity. These findings could offer valuable insights for policy makers to develop and improve cancer control, with the vision of a world where cancer in preventable and every survivor remains healthy.

Cancer remains the second most common cause of death worldwide, with its burden steadily rising over the past decades [1]. In 2022, approximately 20.0 million new cancer cases and 9.7 million cancer-related deaths were reported globally [2]. Concerningly, these figures translated to approximately one in five men or women developing cancer during their lifetime, with mortality rates being one in nine for males and one in twelve for females. Between 2020 and 2050, cancers will impose a cost of international dollars 25.2 trillion on the global economy, equivalent to an annual levy of 0.55% on global gross domestic product (GDP) [3]. These statistics reflected significant challenges of cancer on health care systems and societies, not only due to its direct health impacts but also its broader socioeconomic consequences.

Previous epidemiological studies on cancer burden have concentrated on the analysis of temporal trends and regional variations in incidence, prevalence, mortality, and disability-adjusted life years (DALYs). For instance, Tu et al. reported that the DALYs of tracheal, bronchus, and lung cancer (TBLC) cancer increased by 49.88% globally from 1992 to 2021 [4]. Huang et al. documented a 21% rise in DALYs of liver cancer from 2010 to 2019 [5]. Additionally, Yi et al. examined the DALYs of women's cancers from 1990 to 2019 [6]. Disability-adjusted life years are a composite measure of disease burden, combining years of life lost (YLL) due to premature mortality and years lived with disability (YLD). Such studies have provided significant understanding of the changes in cancer-related DALYs. However, the changes in DALYs may be driven by multiple factors. For instance, an increase in the total population can directly cause a higher number of cases, even if the prevalence per person remains stable. This is particularly important in regions with rapid population growth or migration. Since cancer is more prevalent among the elderly, a growing proportion of older individuals can significantly increase the overall disease burden. Changes in prevalence reflect intrinsic alterations in disease risk or incidence, which are crucial for evaluating the impact of cancer interventions or policies. For example, a decline in smoking rates may reduce the prevalence of lung cancer, whereas early screening programmes can increase the prevalence of thyroid cancer. Higher DALYs suggest greater disease severity, and lower prevalence points to rapid mortality after disease onset. Therefore, a larger ratio of DALYs to prevalence corresponds to greater disease severity. While significant disparities in disease severity exist across cancers, regions, and time periods, the specific contribution of changes in disease severity on the cancer-related disease burden remain poorly understood. Addressing this knowledge gap is essential for developing targeted policies and interventions to optimise the allocation of health care resources and reduce global disparities in cancer outcomes.

In this study, we employed a decomposition analysis to quantify the impact of disease severity on the cancer burden, adhering to the methodology established by Das Gupta and improved by Cheng et al. [7,8]. Based on the assumptions of factor additivity, independence, and linearity, this method decomposes changes in cancer DALYs into four distinct components: population growth, population aging, shifts in disease prevalence rates, and alterations in disease severity [8]. The methodology of Das Gupta offers several advantages. First, it enables a systematic examination of the interplay between demographic changes, epidemiological trends, and disease severity. Second, it provides a granular understanding of the contributions of each factor to the observed burden. Third, it allows for cross-comparisons between regions, highlighting disparities and guiding resource prioritisation where they are most needed. Furthermore, Chang et al.'s strategy for decomposing DALYs variations into four factors was consistent with the analytical structure of our research.

In summary, this study utilised the GBD 2021 data and employed a four-factor decomposition analysis to quantify the contribution of changes in disease severity to cancer-related DALYs. We further examined whether this contribution varied by cancer type, gender, or region. Our research objectives include deepening our understanding of cancer epidemiologic patterns and provide data support for global cancer prevention and control. Concurrently, we aimed to provide policymakers with actionable insights for the equitable allocation of cancer-related health resources, aligning with the United Nations Sustainable Development Goals (SDGs) 3.4 (reducing non-communicable disease mortality), 3.8 (universal health coverage), and 3.9 (mitigating health risks from hazardous chemicals) [9].

## METHODS

### Data sources

An updated and comprehensive perspective on epidemiological data for 371 diseases, injuries [10], 88 risk factors [11], and 288 causes of death [12] categorised by age and gender across 204 countries and territories, is now available through the GBD 2021. Descriptions and detailed methodologies of the GBD 2021 can be found in previous studies [10–12].

In our secondary analysis, we extracted the estimated rates and numbers of prevalence, DALYs for cause-specific from the GBD 2021 Results Tool for the period between 1990 and 2021 [13]. The population size data were also downloaded. We defined the adult population as individuals aged 20 and older. Consequently, the age parameter was divided into sixteen age groups, including 20–24, 25–29, 30–34, 35–39, 40–44, 45–49, 50–54, 55–59, 60–64, 65–69, 70–74, 75–79, 80–84, 85–89, 90–94, 95+. The location parameter included global, five sociodemographic index (SDI) regions, 21 GBD regions, and 204 countries/territories.

### Definition of disease burden and cancer

Employing the GBD database, our research examined the adult disease burden of neoplasms and 20 types of cancer, including nasopharynx cancer (NPC), larynx cancer (LarC), lip and oral cavity cancer (LOC), thyroid cancer(ThyC), tracheal, bronchus, and lung cancer (TBLC), mesothelioma(Meso), oesophageal cancer (EC), stomach cancer (SC), colon and rectum cancer (CRC), liver cancer (LivC), gallbladder and biliary tract cancer (GBTC), pancreatic cancer (PanC), kidney cancer (KC), bladder cancer(BlaC), prostate cancer (ProC), testicular cancer (TesC), breast cancer (BreC), ovarian cancer (OC), cervical cancer(CC), uterine cancer (UC). Additionally, we assessed the burden of disease through the metric of DALYs. In the GBD 2021 study, specific cancer types were identified using diagnostic codes from the International Classification of Diseases, including ICD-10 and ICD-9 (Table S1 in the **Online Supplementary Document**) [12]. In our secondary analysis, we directly utilised these predefined cancer categories to estimate trends in disease burden and severity, with no additional modifications.

### GBD regions and sociodemographic index (SDI)

Aiming to enhance inter-regional comparisons, researchers in the GDB database categorised 204 countries and territories into 21 GBD regions based on epidemiological homogeneity and geographic contiguity. In addition, the SDI, ranging from 0 to 1, is a comprehensive measure assessing the development status of various regions [14]. It is calculated by educational levels for those aged 15 and above, averaging the rankings of the fertility rate for women under 25, and time-adjusted per capita income. SDI scores vary from a near-zero baseline, representing low development, to a high maximum, signifying advanced development. This metric categorises countries and territories into quintiles based on their SDI values: low SDI, low-middle SDI, middle SDI, high-middle SDI, and high SDI. In our research, SDI values for all countries and territories were sourced from the download link (https://ghdx.healthdata.org/record/global-burden-disease-study-2021-gbd-2021-socio-demographic-index-sdi-1950-2021) (Table S2–3 in the **Online Supplementary Document**) [15].

### Statistical analysis

#### *DALY_a,d,y_* as the product of four factors

Unlike the traditional three-factor decomposition analysis of cancer DALYs, we adopted the strategy of Chang et al. to identify four factors contributing to changes in cancer-related DALYs: population size, age structure, disease-specific prevalence, and case fatality and disease severity (formula 1) [8,13]. Here, the *DALY_a_*_,_*_d_*_,_*_y_*/*prevalence_a_*_,_*_d_*_,_*_y_* ratio serves as an indicator of disease severity. With 1990 as the baseline, we examined the temporal trends of absolute and relative contributions of disease severity changes to DALY variations from 1991 to 2021. The absolute contribution was defined by the attributed numbers of DALYs. Additionally, the relative contribution, labelled ‘attributable proportion’, was quantified by the percentage of attributable DALYs out of the 1990 total DALYs.



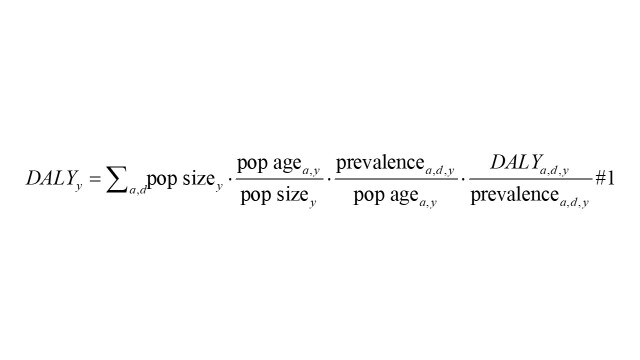



The formula can be broken down into the following components:

1) *pop size_y_*: the overall population for year *y*;

2) *pop age_a_*_,_*_y_*: the number of individuals in 5-year age group *a* in year *y*;

3) *prevalence_a_*_,_*_d_*_,_*_y_*: the prevalence of disease *d* among age group *a* in year *y*;

4) *DALY_a_*_,_*_d_*_,_*_y_*: the DALY associated with disease *d* for age group *a* in year *y*.

#### Simplifying the formula

Consistent with Das Gupta's methodology [7,16], the end product is the result of four factors multiplied together as formula 2. *D_asly_* denotes the disease burden of DALYs for cancers at year *y*. *A_sly_* is the total adult population for a given sex *s* and location *l* in year *y*; *B_asly_* denotes the proportion of this population within age group *a*, corresponding to the same sex *s* and location *l* in year *y*; *P_asly_* represents the prevalence of cancer-related diseases within age group *a*, sex *s* and location *l* in year *y*; and *S_asly_* signifies the impact or severity of these diseases within a defined age group *a*, sex *s* and location *l* in year *y*.



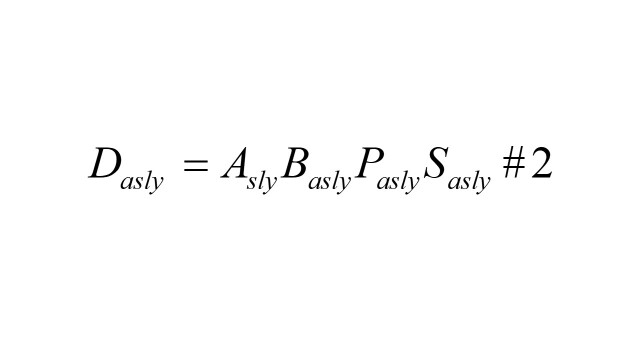



#### Calculate the additive contribution of disease severity

Finally, the contribution of disease severity (*S*) to changes in the total disease burden from 1990 (*S_90_*) to year *y* (*S_y_*: each year between 1991 and 2021) was quantified according to formula 3, where *E_S_* represents the proportion of change resulting from factor *S* [7,8,16].



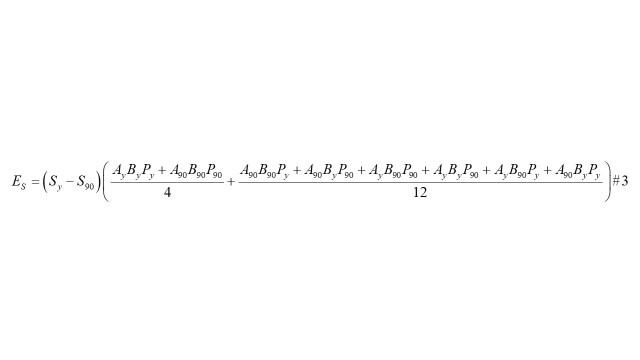



Based on formulas 1–3 [8,17], absolute and relative contributions of disease severity were quantified by examining the changes in cancer-related DALYs between 1990 and year y (from 1991 to 2021). Comparative analyses were subsequently stratified by region, SDI, sex, year, and cancer type.

The execution of statistical analyses was accomplished through the application of R software, version 4.4.1 (R Core Team, Vienna, Austria). Detailed explanations of the R code are provided in the **Online Supplementary Document**.

## RESULTS

### Change in DALYs attributable to disease severity at global level

**Figure 1:**
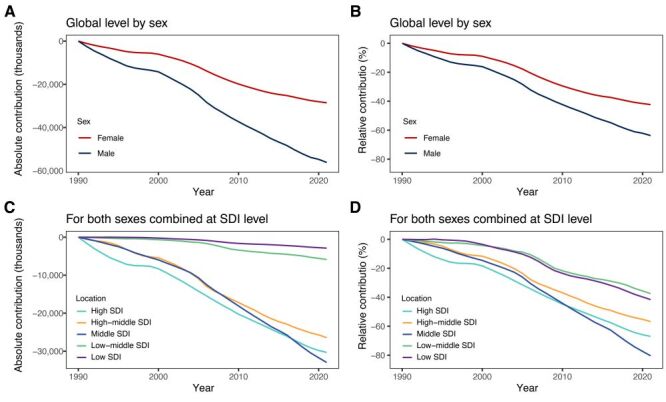
Changes in neoplasms-related DALYs attributable to disease severity from 1990 to 2021 with 1990 as the reference for each year. **Panel A.** Absolute contribution by sex at global level. **Panel B.** Relative contribution by sex at global level. **Panel C.** Absolute contribution in both sexes combined at SDI regional level. **Panel D.** Relative contribution in both sexes combined at SDI regional level. DALYs – disability-adjusted life-years, GBD – Global Burden of Disease, SDI – sociodemographic index.

With 1990 as the reference year, a steady decrease in neoplasms-related DALYs attributable to disease severity was observed for both sexes combined globally from 1991 to 2021, reaching −81 963.31 thousand in 2021(**Table 1**; Table S4 in the **Online Supplementary Document**). Notably, the decline was more pronounced in males (n = −56 172.28 thousand) compared to females (n = −28 558.95 thousand) (**Table 1**; **Figure 1**, Panel A). In addition, the global relative contribution associated with disease severity changes for neoplasms exhibited a parallel downward trend (−52.68%), with males (−63.72%) experiencing a significantly greater reduction than females (−42.35%) (**Figure 1**, Panel B, **Table 1**).

**Table 1:** Contribution to disease severity changes for neoplasms by sex at global, SDI regional, and GBD regional levels between 1990 and 2021

Location	Both sexes combined		Male		Female	
	**Absolute contribution (thousands)**	**Relative contribution (%)**	**Absolute contribution (thousands)**	**Relative contribution (%)**	**Absolute contribution (thousands)**	**Relative contribution (%)**
**Global**	−81 963.31	−52.68%	−56 172.28	−63.72%	−28 558.95	−42.35%
**SDI regions**						
High SDI	−30 317.14	−67.01%	−20 164.15	−79.30%	−11 238.66	−56.72%
High-middle SDI	−26 445.21	−56.78%	−20 128.27	−71.19%	−8371.11	−45.74%
Middle SDI	−32 958.22	−80.36%	−19 728.93	−83.86%	−13 198.93	−75.48%
Low-middle SDI	−5869.18	−37.48%	−2538.29	−32.93%	−2997.64	−37.69%
Low SDI	−2881.96	−41.62%	−1135.30	−36.34%	−1660.62	−43.69%
**GBD regions**						
High-income North America	−11 472.46	−79.29%	−6934.50	−89.68%	−4747.90	−70.48%
Central Latin America	−2328.20	−89.73%	−1133.45	−96.31%	−1187.16	−83.73%
Andean Latin America	−657.00	−91.53%	−353.53	−108.99%	−316.18	−80.37%
Caribbean	−430.29	−48.45%	−279.19	−62.06%	−163.50	−37.30%
Tropical Latin America	−2187.18	−69.07%	−1310.84	−77.82%	−839.53	−56.65%
Southern Latin America	−1129.96	−56.67%	−782.85	−71.81%	−393.79	−43.57%
Western Europe	−12 062.35	−52.10%	−8906.88	−67.71%	−3986.78	−39.87%
Central Europe	−2895.55	−42.46%	−2259.61	−56.28%	−909.01	−32.41%
Eastern Europe	−5618.11	−45.02%	−4297.28	−59.94%	−1825.37	−34.37%
Central Asia	−973.85	−48.61%	−621.55	−55.58%	−354.06	−39.99%
East Asia	−45 158.85	−99.71%	−29 142.72	−103.15%	−16 643.99	−97.68%
South Asia	−7014.36	−49.34%	−2668.47	−37.40%	−3857.02	−54.47%
Southeast Asia	−3783.74	−48.69%	−2010.74	−52.40%	−1873.74	−47.64%
High-income Asia Pacific	−5181.41	−67.70%	−3437.67	−73.83%	−1850.42	−61.73%
Australasia	−586.84	−61.94%	−382.16	−72.20%	−219.17	−52.41%
Oceania	−17.46	−19.52%	−10.84	−25.94%	−6.58	−13.81%
North Africa and Middle East	−4206.66	−90.11%	−2636.38	−95.31%	−1496.67	−78.69%
Western sub-Saharan Africa	−713.14	−35.79%	−307.03	−32.44%	−345.83	−33.06%
Central sub-Saharan Africa	−279.77	−38.31%	−129.82	−39.80%	−142.09	−35.16%
Eastern sub-Saharan Africa	−1357.88	−45.22%	−528.15	−41.23%	−817.96	−47.50%
Southern sub-Saharan Africa	−37.71	−3.97%	−69.71	−14.69%	38.82	8.19%

GBD *–* Global Burden of Disease, SDI – sociodemographic index

**Figure 2:**
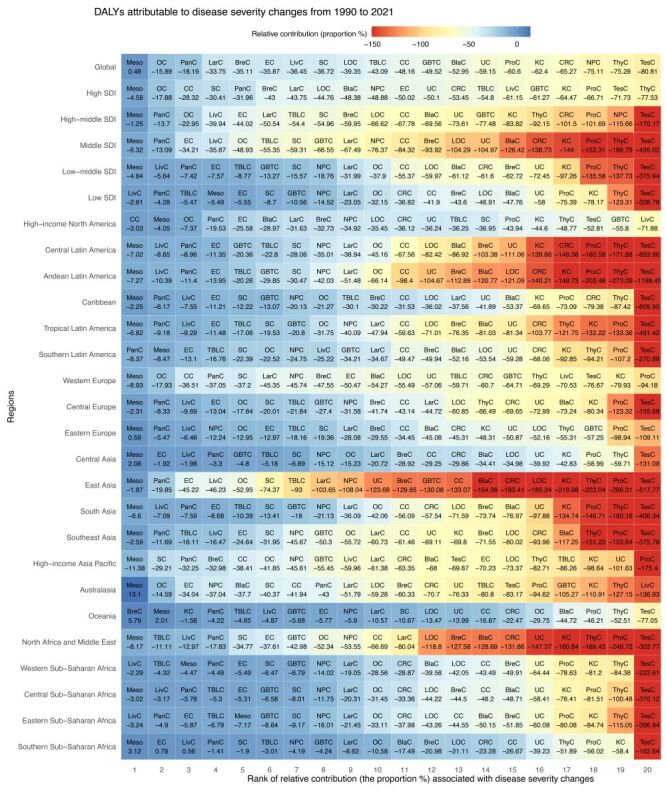
Relative contribution (the proportion %) associated with disease severity changes for 20 types of cancer in both sexes combined between 1990 and 2021 at global, SDI regional, and GBD regional levels. BlaC – bladder cancer, BreC – breast cancer, CC – cervical cancer, CRC – colon and rectum cancer, DALYs – disability-adjusted life-years, EC – oesophageal cancer, GBD – Global Burden of Disease, GBTC – gallbladder and biliary tract cancer, KC – kidney cancer, LarC – larynx cancer, LivC – liver cancer, LOC – lip and oral cavity cancer, Meso – mesothelioma, NPC – nasopharynx cancer, OC – ovarian cancer, PanC – pancreatic cancer, ProC – prostate cancer, SC – stomach cancer, SDI – sociodemographic index, TBLC – tracheal, bronchus, and lung cancer, TesC – testicular cancer, ThyC – thyroid cancer, UC – uterine cancer.

**Figure 3:**
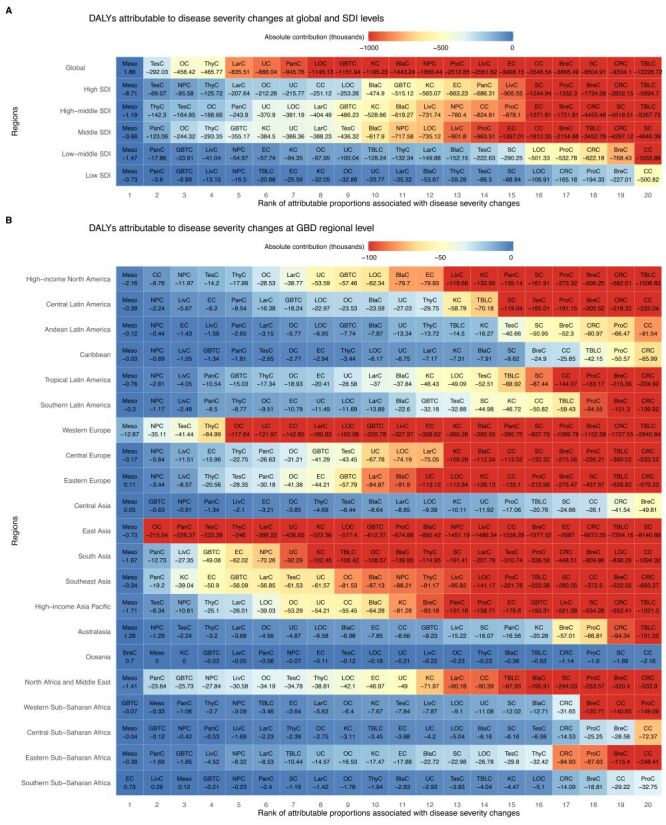
Absolute contribution (the number) associated with disease severity changes for 20 types of cancer in both sexes combined between 1990 and 2021 at global, SDI regional, and GBD regional levels. **Panel A.** At global, SDI regional levels. **Panel B.** At GBD regional levels. BlaC – bladder cancer, BreC – breast cancer, CC – cervical cancer, CRC – colon and rectum cancer, DALYs – disability-adjusted life-years, EC – oesophageal cancer, GBD – Global Burden of Disease, GBTC – gallbladder and biliary tract cancer, KC – kidney cancer, LarC – larynx cancer, LivC – liver cancer, LOC – lip and oral cavity cancer, meso – mesothelioma, NPC – nasopharynx cancer, OC – ovarian cancer, PanC – pancreatic cancer, ProC – prostate cancer, SC – stomach cancer, SDI – sociodemographic index, TBLC – tracheal, bronchus, and lung cancer, TesC – testicular cancer, ThyC – thyroid cancer, UC – uterine cancer.

Over the study period, most cancers experienced a global reduction in both absolute and relative contribution due to changes in disease severity, except for mesothelioma, where a slight increase of 1.86 thousand (0.48%) was observed (**Table 2**; Table S4 in the **Online Supplementary Document**). Consequently, notable disparities were observed among the 20 cancer types in this trend, with the most significant relative reduction in testicular cancer (−80.81%), and the largest reduction in absolute contribution noted in tracheal, bronchus, and lung cancer (n = −12 228.72 thousand) (**Figure 2**, **Figure 3**, **Table 2**). For males, tracheal, bronchus, and lung cancer (n = −8035.21 thousand) exhibited the most significant reduction of absolute contribution among the 20 cancer types, while breast cancer (n = −3866.49 thousand) was the most reduced in females (**Table 2**). Thyroid cancer, meanwhile, experienced the highest decrease in relative contribution in both males (−92.34%) and females (−73.32%) (**Table 2**).

**Table 2:** Contribution to disease severity changes for 20 types of cancer by sex at global level between 1990 and 2021

Cancers	Both sexes combined		Male		Female	
**Absolute contribution (thousands)**	**Relative contribution (%)**	**Absolute contribution (thousands)**	**Relative contribution (%)**	**Absolute contribution (thousands)**	**Relative contribution (%)**
Nasopharynx cancer	−1665.44	−75.11%	−1233.38	−81.77%	−432.56	−61.01%
Larynx cancer	−835.51	−33.75%	−726.39	−33.3%	−105.41	−35.77%
Lip and oral cavity cancer	−1145.13	−39.35%	−707.16	−34.45%	−413.81	−48.27%
Thyroid cancer	−465.77	−75.28%	−197.61	−92.34%	−296.71	−73.32%
Tracheal, bronchus, and lung cancer	−12228.72	−43.09%	−8035.21	−37.84%	−3805.45	−53.24%
Mesothelioma	1.86	0.48%	7.31	2.60%	−4.79	−4.55%
Oesophageal cancer	−3498.15	−35.87%	−2426.03	−34.49%	−1111.37	−40.88%
Stomach cancer	−8504.91	−36.72%	−6288.22	−41.81%	−2069.64	−25.49%
Colon and rectum cancer	−9334.10	−65.27%	−5533.41	−73.24%	−3865.96	−57.3%
Liver cancer	−2561.62	−36.45%	−1915.22	−37.87%	−633.95	−32.2%
Gallbladder and biliary tract cancer	−1151.94	−49.52%	−605.51	−66.46%	−509.16	−35.98%
Pancreatic cancer	−945.76	−18.19%	−485.55	−16.46%	−465.58	−20.7%
Kidney cancer	−1195.23	−62.4%	−843.58	−67.61%	−387.95	−58.1%
Bladder cancer	−1443.24	−52.95%	−1130.03	−55.79%	−314.02	−44.85%
Prostate cancer	NA	NA	−2512.85	−60.6%	NA	NA
Testicular cancer	NA	NA	−292.03	−80.81%	NA	NA
Breast cancer	NA	NA	NA	NA	−3866.49	−35.11%
Ovarian cancer	NA	NA	NA	NA	−456.42	−15.89%
Cervical cancer	NA	NA	NA	NA	−3546.54	−48.16%
Uterine cancer	NA	NA	NA	NA	−888.04	−59.15%

### Change in DALYs attributable to disease severity at SDI regional level

Over the period from 1990 to 2021, the contribution of disease severity changes to neoplasms-related DALYs consistently declined across all SDI regions (**Figure 1**, Panels C–D, **Table 1**). The decline was most pronounced in middle SDI (n = −32 958.22 thousand; −80.36%) and high SDI (n = −30 317.14 thousand; −67.01%) regions, compared to more moderate reductions in low-middle SDI (n = −5869.18 thousand; −37.48%) and low SDI (n = −2881.96 thousand; −41.62%) regions. Similar patterns were observed in both sexes, with middle SDI regions showing the highest attributable proportion decline among males (n = −19 728.93 thousand; −83.86%) and females (n = −13 198.93 thousand; −75.48%) (**Table 1**; Figure S1 in the **Online Supplementary Document**).

The influence of disease severity changes varied across cancers and SDI levels. In terms of relative contribution, the impact declined for 20 cancers across all SDI regions (**Figure 2**). Thyroid cancer exhibited the steepest reduction in high SDI (−77.53%), while testicular cancer showed the sharpest declines in high-middle (−170.17%), middle (−495.02%), lower-middle (−375.94%), and low SDI (−328.78%) regions (Table S5–6 in the **Online Supplementary Document**). Among males, mesothelioma in low-middle SDI (0.19%) was the only cancer with a slight increase (Table S7 and Figure S2 in the **Online Supplementary Document**). Among females, thyroid cancer demonstrated the largest decline across high (−76.35%), middle (−176.70%), lower-middle (−136.27%), and low SDI (−124.83%) regions, while nasopharyngeal cancer saw the most significant decrease in high-middle SDI (−97.12%) (Table S5, Table S8, and Figure S3 in the **Online Supplementary Document**). Besides, Middle SDI regions recorded the highest number of cancers with relative contributions exceeding −150% (**Figure 2**; Figures S2–3 in the **Online Supplementary Document**).

In absolute contribution terms, tracheal, bronchus, and lung cancer exhibited the largest DALY reductions in high (n = −5694.70 thousand) and high-middle SDI (n = −5367.75 thousand) regions, stomach cancer in middle SDI (n = −4846.39 thousand), and cervical cancer in low-middle (n = −1055.88 thousand) and low SDI (n = −500.82 thousand) regions (**Figure 3**, Panel A; Table S9–S11 in the **Online Supplementary Document**). Among males, tracheal, bronchus, and lung cancer showed the greatest decline in high (n = −3745.09 thousand), high-middle (n = −3662.53 thousand), and middle SDI (n = −3517.90 thousand) regions, while prostate cancer had the largest reduction in low-middle (n = −532.78 thousand) and low SDI (n = −194.33 thousand) regions (Table S9, Table S12, and Figure S4 in the **Online Supplementary Document**). Among females, the pattern was similar, except that breast cancer experienced the most significant decrease in middle SDI (n = −2154.68 thousand) (Table S13 and Figure S5 in the **Online Supplementary Document**).

### Change in DALYs attributable to disease severity at GBD regional level

Between 1990 and 2021, all 21 GBD regions experienced declines in the number and proportion of cancer-related DALYs due to disease severity, with the most pronounced reduction in East Asia (n = −45 158.85 thousand; −99.71%) (**Table 1**, Figure S6–7 in the **Online Supplementary Document**). Furthermore, the smallest decline in absolute contribution was observed in Oceania at −17.46 thousand, while southern sub-Saharan Africa had the lowest relative contribution decrease at −3.97%. Females showed similar patterns, with East Asia having the highest absolute and relative contributions (n = −16 643.99 thousand; −97.68%) (**Table 1**). Notably, females in southern sub-Saharan Africa (n = 38.82 thousand; 8.19%) demonstrated an increase in both types of contributions. In males, East Asia experienced the steepest absolute contribution decline (n = 29 142.72 thousand), and Andean Latin America the relative contribution (−108.99%) (**Table 1**). Furthermore, the 21 GBD regions predominantly demonstrated a steady downward trend from 1990 to 2021 with 1990 as the reference year. In contrast, southern sub-Saharan Africa followed an inverted U-shaped trend, with an initial increase followed by a decrease (Figure S6–7 in the **Online Supplementary Document**).

The 21 GBD regions exhibited substantial heterogeneity in the impact of disease severity changes across 20 cancer types (**Figure 2**; Figure S2–5 in the **Online Supplementary Document**). In attributable proportion terms, a more considerable decrease was noted in testicular, liver (in high-income North America and Australasia), and prostate cancers, compared to the less substantial decrease in mesothelioma, pancreatic, and liver cancers (in western and eastern sub-Saharan Africa) (**Figure 2**; Table S14–15 in the **Online Supplementary Document**). Mesothelioma, even in Australasia, Central Europe, Central Asia, and southern sub-Saharan Africa, shown an upward trend (**Figure 2**). In addition, testicular and liver cancers in men, along with thyroid and kidney cancers in women, experienced more pronounced declines (Figure S2–3 and Table S16 in the **Online Supplementary Document**). Considering their absolute contribution, lung and colorectal cancers were among the major types with larger decreases, while mesothelioma continued to have the smallest decreases (**Figure 3**, Panel B; Table S17 in the **Online Supplementary Document**). Additionally, more pronounced decreases were also observed in prostate, oesophageal, and testicular cancers among men, and breast, cervical, and oesophageal cancers among women (Figure S4–5 and Table S18 in the **Online Supplementary Document**).

### Change DALYs attributable to disease severity at national level

Between 1990 and 2021, disease severity changes led to increased neoplasms-related DALYs in several African nations, including Zimbabwe (n = 65.28 thousand, 41.06%), Lesotho (18.22 thousand, 77.80%), Eswatini (3.29 thousand, 26.56%), and Chad (2.17 thousand, 3.65%), while declining in 200 countries and territories (Table S4, and Figure S8 in the **Online Supplementary Document**). Among males, the upward trend was observed in Zimbabwe, Mozambique, Lesotho, Kenya, Eswatini, and Gambia, whereas among females, increases were noted in Zimbabwe, Lesotho, Chad, Eswatini, Sierra Leone, Gambia, Solomon Islands, Vanuatu, American Samoa, and the Marshall Islands (Figure S8 in the **Online Supplementary Document**). In contrast, China recorded the most significant absolute decline in DALYs (n = −44 306.96 thousand overall; n = −28 570.33 thousand in males, n = −16 314.08 thousand in females), while Qatar exhibited the largest relative reduction (−337.34% overall; −364.33% in males, −321.90% in females) (Table S4, and Figure S8 in the **Online Supplementary Document**).

Among 20 types of cancer, thyroid, kidney, prostate, and testicular were the ones with a considerable number of countries where the attributable proportion decreased beyond −100% (Table S5–24 in the **Online Supplementary Document**). Notably, mesothelioma in men exhibited a relative contribution greater than 0% in 112 countries and territories (Table S25 in the **Online Supplementary Document**). In general, most countries exhibited a relative contribution range of disease severity associated with cancer-related DALYs from 0% to −60% (Table S25 in the **Online Supplementary Document**).

## DISCUSSION

This secondary analysis is the first to explore the effect of disease severity on cancers-related DALYs from 1990 to 2021. By utilising the GBD 2021 data set, it allows for in-depth comparisons of various cancers across countries, and over an extended period, thereby improving our understanding of the impact of changes in disease severity. Our research has uncovered several important insights. First, the global neoplasms-related DALYs attributed to changes in disease severity declined from 1991 to 2021, with a more significant decrease observed in men than in women. Second, with the exception of a slight rise in mesothelioma-related DALYs due to changes in disease severity, all other cancers exhibited declines. Lung cancer experienced the most significant absolute contribution, and testicular cancer demonstrated the highest relative contribution. Third, high SDI and middle SDI regions experienced more significant reductions compared to low-middle and low SDI regions. In 21 GBD regions, East Asia demonstrated the largest absolute and relative contributions, while southern sub-Saharan Africa exhibited an inverted U-shaped curve trend. Among 204 countries and territories, China shown the largest absolute contribution to the decline in neoplasms-related DALYs due to changes in disease severity, and Qatar had the highest relative contribution.

Cancer has become one of the most significant challenges to global public health, with its burden on human health continuously increasing. According to a study by Freddie Bray and his team, cancer has surpassed cardiovascular diseases to become the leading cause of premature death in 57 countries [1]. Additionally, it was estimated that by the end of this century, cancer would likely surpass cardiovascular diseases to become the leading cause of premature death in most countries [1]. In the GBD data from 2017, the primary causes of cancer-related DALYs were tracheal, bronchus, and lung cancer, liver cancer, stomach cancer, colon and rectum cancer, and breast cancer, with values of 40.9 million, 20.8 million, 19.1 million, 19.0 million, and 17.7 million, respectively [18]. This considerable cancer-related DALYs impose a significant burden on public health systems, socioeconomic development, and health care infrastructure in countries around the world. Cancer patients frequently endure extended treatment courses, including surgery, radiotherapy, chemotherapy, and targeted therapies. These interventions not only induce physical distress but also have the potential to adversely impact patients' psychological well-being, family dynamics, and social functioning. Furthermore, prolonged recovery and ongoing care are often required, leading to work disruptions and reduced productivity, which in turn increases socioeconomic costs. Studies have shown that the financial burden of cancer treatment represents a significant economic strain for many families, especially in low-and middle-income countries (LMICs), where the high costs of care are frequently unaffordable [19,20]. This financial hardship can delay early diagnosis and obstruct timely, effective treatment. The key finding of our study indicated that, despite the continued rise in cancer incidence and mortality, the DALYs resulting from changes in the severity of cancer has steadily declined from 1991 to 2021. This trend reflected substantial advancements in cancer treatment and management, which could be attributed to several potential contributing factors. First, a growing number of countries have introduced nationwide screening programmes for lung cancer, breast cancer, stomach cancer, colorectal cancer, thyroid cancer, and cervical cancer. These programmes have proven effective in reducing the severity of these cancers through early diagnosis. Evidence indicates that early screening and timely treatment of breast cancer can substantially reduce mortality and improve survival rates [21]. Similarly, regular screening for colorectal cancer allows for the detection of early-stage abnormalities, thereby preventing the treatment failures and mortality associated with late-stage diagnosis [22]. Second, ongoing advancements in medical imaging, molecular biology, and related technologies have substantially improved the early detection of cancer. Concurrently, the issue of unequal distribution of cancer treatment resources worldwide has gradually improved. Although regional disparities persist, some developing countries have made notable progress in enhancing cancer treatment facilities, particularly those with higher income levels and stronger health care systems. Third, there is a global increase in cancer prevention awareness. This includes the improvement of public health policies such as smoking bans, promoting healthy diets, and encouraging physical exercise, as well as the strengthening of health education. Moreover, the widespread adoption of the hepatitis B virus vaccine has also played a critical role in these efforts. The introduction of large-scale *Helicobacter pylori* screening and eradication programmes has significantly strengthened gastrointestinal cancer prevention strategies [23]. Fourthly, the development of targeted therapies, immunotherapies, and precision medicine has led to a significant enhancement in cancer treatment efficacy. These approaches have not only increased patient survival rates but also reduced treatment-related side effects and minimised disability caused by cancer-related health issues. For example, lung cancer patients receiving immune checkpoint inhibitors and breast cancer patients treated with breast-conserving surgery and radiation therapy have experienced a notable increase in survival, and some have also reported an improvement in their quality of life [24–26].

Our research found notable gender disparities in the decline of cancers-related DALYs resulting from changes in disease severity. For absolute DALYs reductions due to changes in disease severity, lung cancer had the most substantial impact in males, and breast cancer had the most in females. In terms of relative DALYs reductions, thyroid cancer demonstrated the greatest decrease in both males and females. Overall, males experienced a more substantial reduction in DALYs than females, with lung cancer accounting for a significant proportion of this disparity. This observed significant gender disparities highlighted complex interactions between biological, behavioural, and differences in cancer types. Biological factors, such as differential metabolism of carcinogens and variations in immune response, may partially explain the observed trends [27,28]. Behaviourally, targeted public health initiatives, including smoking cessation programmes, taxation, and restrictions on tobacco advertising, have predominantly focused on male-dominated populations [29]. Such measures have proven effective in reducing tobacco-related cancers [30]. In contrast, less aggressive implementation of similar interventions among women in certain regions may contribute to the slower decline in female cancer DALYs. Gender-specific cancer types also play a role in shaping these disparities. For example, breast cancer, the most common cancer among females, has seen significant advancements in early detection and treatment. However, due to its high baseline incidence and longer survival rates, the relative reduction in DALYs has been less pronounced compared to the sharp declines in lung cancer-related DALYs among males. Conversely, cancers more prevalent among males, such as oesophageal and stomach cancer, have benefited disproportionately from improvements in diagnosis and treatment modalities, contributing to the overall gender gap. Screening uptake is another crucial factor that can explain disparities between genders. For example, women may benefit from early detection programmes for breast and cervical cancers. However, in some parts of the world, cultural stigmas around female cancers, particularly cervical cancer, may discourage women from seeking care or undergoing screening [31,32]. Furthermore, gender norms, limited autonomy, and socioeconomic inequalities may further restrict women's access to early diagnosis and treatment options for cancers, especially in low-resource settings.

This research underscores the complex pattern of DALYs alterations in different cancers globally due to changes in disease severity. Lung, colorectal and stomach cancers demonstrated the largest decline in absolute contribution from 1990 to 2021. Meanwhile, testicular, thyroid, and nasopharyngeal cancers experienced the most significant decline in relative contribution. On the contrary, mesothelioma showed a slight increase in both absolute and relative contributions. For lung cancer, smoking cessation programmes and tobacco control policies have contributed to a decline in male smoking rates [29]. The introduction of low-dose computed tomography has revolutionised lung cancer screening, allowing for the detection of early-stage cancers that are more likely to be curable. The progress in precision medicine, with Epidermal Growth Factor Receptor (EGFR) inhibitors, Anaplastic Lymphoma Kinase (ALK) inhibitors, and immune checkpoint inhibitors, has significantly improved survival and quality of life for patients with advanced lung cancer [24,33]. These combined efforts may reduce the severity of lung cancer, resulting in a significant reduction in lung cancer-related DALYs. For colorectal cancer, colonoscopy and faecal immunochemical test enable early diagnosis, often at a stage more amenable to curative treatment. The advancement of adjuvant and neoadjuvant chemotherapy, particularly with the incorporation of fluoropyrimidine and oxaliplatin, has improved survival outcomes [34]. Additionally, the introduction of more effective systemic and targeted therapies, such as anti-EGFR and anti-Vascular Endothelial Growth Factor treatments, has further increased survival rates [34]. Regarding stomach cancer, several factors are significant: public health measures like antibiotic programmes targeting *Helicobacter pylori* in high-incidence areas, dietary improvements including reduced consumption of pickled and salty foods, and the wider implementation of population-based screening in East Asia for early detection [35]. Additionally, the progress in endoscopic techniques, such as endoscopic submucosal dissection, has made curative treatment of early stomach cancer possible [35]. For nasopharyngeal cancer, early detection using Epstein-Barr virus DNA as a biomarker helps identify asymptomatic or early-stage cases [36]. Advances in radiotherapy, particularly the adoption of intensity-modulated radiation therapy, have improved local control rates while minimising treatment-related toxicity [37]. Concurrent chemoradiotherapy further enhances overall survival in advanced disease and reduces the long-term disability burden. For testicular cancer, cisplatin-based chemotherapy has been notably enhanced survival rates, achieving a cure rate of more than 95% for all patients and approximately 90% for metastatic cases [38]. For thyroid cancer, the combined advancements in ultrasound imaging, fine-needle aspiration biopsy, and molecular diagnostics have significantly improved the early and accurate identification of thyroid nodules and malignant cases. Concurrently, surgical techniques have developed to be more precise, which not only preserves thyroid function but also reduces the incidence of postoperative complications [39]. Malignant mesothelioma was the only cancer with rising DALYs due to changes in disease severity. Despite bans or strict restrictions on asbestos use in many countries, it remains widely used in some areas, leading to ongoing occupational and environmental exposure. Moreover, given the lengthy latency period of 30 to 40 years, the incidence of mesothelioma may continue to increase [40]. Over the past three decades, international conventions, notably the C162 Asbestos Convention and the Basel Convention, have guided governments in regulating asbestos use in workplaces and managing asbestos as waste [41]. By 2021, more than 60 countries, including European Union member states, Australia, and Japan, had implemented comprehensive bans on asbestos. Furthermore, countries that have not fully banned asbestos, such as the United States, have introduced stricter regulations on its use and handling to limit its harm. We anticipate that with reduced exposure levels, the increase in new cases will gradually decelerate in the medium to long term. Meanwhile, immunotherapy, an emerging treatment, provides improved survival outcomes for patients with advanced conditions [42].

A striking observation was the substantial decrease of cancer-related DALYs due to changes in disease severity in high-SDI and middle-SDI regions compared to low-SDI and low-middle-SDI regions. These patterns suggest that socioeconomic development significantly influences changes in cancer-related DALYs, mediated by factors such as health care accessibility, cancer screening and early detection programmes, and improved oncology treatments. Lung, stomach, and colorectal cancers contributed the most to absolute reductions in high-SDI regions, underscoring the advancements in preventive measures and therapeutic interventions in these settings. Conversely, cervical, breast, and prostate cancers ranked high in low SDI regions. Although the absolute number of cervical cancer-related DALYs due to changes in disease severity was highest in Low and low-middle SDI regions between 1990 and 2021, nearly 94% of cervical cancer deaths in 2022 were in LMICs, and vaccination rates were still lower than in high-income countries [43,44]. The World Health Assembly, in August 2020, formally adopted the Global Strategy for eliminate cervical cancer [43]. Accordingly, in low and low-middle SDI regions, Human Papillomavirus (HPV) vaccination programmes and routine screening, such as visual inspection with acetic acid (VIA) and HPV testing, should be widely implemented [45].

Among 21 GBD regions, southern sub-Saharan Africa was the only region where the trend of neoplasms-related DALYs, affected by changes in disease severity, shown an initial increase followed by a decrease from 1991 to 2021. Notably, in Eswatini, Lesotho, and Zimbabwe, both the number and proportion of neoplasms-related DALYs remained positive between 1990 and 2021. One possible explanation is that the widespread prevalence of Human Immunodeficiency Virus and tuberculosis has created a sustained competition for health care resources, diverting funding and infrastructure away from cancer prevention and treatment [46]. Furthermore, the lack of comprehensive cancer screening programmes contributes to the high severity rates of neoplasms. In Zimbabwe, only 21% of women undergo cervical cancer screening, which is well below the 70% coverage recommended by the World Health Organization (WHO) [47]. Without early detection and diagnosis, cancers are often identified at advanced stages when they are more difficult to treat and have a worse prognosis. This delay in diagnosis leads to higher DALYs as individuals suffer from the disease for longer periods and may experience more severe complications. Furthermore, disparities in radiotherapy and chemotherapy availability hinder effective cancer treatment, as many countries in southern sub-Saharan Africa lack comprehensive cancer centres [48,49]. On the contrary, East Asia experienced the most considerable absolute and relative contributions between 1990 and 2021. China, notably, has demonstrated the largest absolute contribution across 204 countries and territories. This epidemiological shift can be attributed to China’s rapid economic growth, which has enabled the allocation of substantial fiscal resources to support extensive investment in oncology-related infrastructure, including state-of-the-art diagnostic equipment and advanced therapeutic technologies. These investments have driven the widespread adoption of advanced radiotherapy, molecular diagnostics, and precision medicine, which have in turn increased early cancer detection rates and decreased the incidence of advanced disease. Studies indicates that increased financial investment in health care is closely associated with improved cancer outcomes. With economic growth, the introduction and expansion of national health insurance programmes – namely, the New Rural Cooperative Medical Scheme, Urban Resident Basic Medical Insurance, and Urban Employee Basic Medical Insurance – have significantly reduced the financial barriers to cancer treatment [50,51]. These reforms have enabled a more equitable distribution of health care services between urban and rural populations, ensuring broader access to early screening and timely treatment across various socioeconomic strata. Recently, government-led price negotiation mechanisms have notably increased the accessibility of targeted cancer drugs. As a result, the prices of key drugs have been significantly reduced, with the median treatment cost reduced by 48.9% and procurement increased by over 143.0% [52]. These efforts have successfully expanded the inclusion of high-cost drugs in the National Reimbursement Drug List (NRDL), making expensive cancer drugs more affordable for cancer patients [52]. Furthermore, targeted public health initiatives strategies play a crucial role, including cancer knowledge education, tobacco control efforts, full coverage of hepatitis B vaccination, and free health screenings for the elderly. In the context of continuous economic and social changes, cancer has become a significant public health issue in China. Despite increasing cancer incidence, enhanced early detection and standardised therapies have contributed to a relative reduction in disease severity. The combination of comprehensive health insurance and strong public health investments to facilitate early intervention and effective management is vital for reducing the severity of cancer cases. These systemic reforms are critical for achieving positive outcomes and offer a valuable framework for other countries grappling with comparable challenges.

We believe that the findings of this study provide important insights for global health policy. Specifically, a substantial reduction in neoplasm-related DALYs was observed globally from 1990 to 2021, with a more significant decline in men than in women. High and middle SDI regions experienced a greater decrease compared with low-middle and low SDI regions. These results highlight the need for global health institutions to focus on the cancer burden in women by strengthening health education and early diagnosis strategies. Additionally, expanding preventive measures for female-specific cancers, such as breast and cervical cancer, through increased HPV vaccination and screening coverage, is critical to narrowing the gender gap. Furthermore, through international collaboration, financial and technical aid should be extended to low-SDI regions to enhance their medical infrastructure and improve cancer prevention and treatment capabilities. Such efforts will ultimately advance global health equity. Successful strategies from China, including universal health insurance, nationwide cancer screening, and primary health care services, should also be considered as models for cancer control. The substantial reduction in disease severity observed in lung, colorectal, and gastric cancers highlight the success of interventions such as tobacco control policies, population-based screening programmes, and standardised treatment protocols. For mesothelioma, these experiences could be adapted through stricter asbestos control, early screening method development, optimised multimodal treatment strategies, and deeper exploration of its biological features. All these efforts align with the goals of SDGs 3.4, 3.8, and 3.9 [9].

In this secondary analysis study of GBD data, we sought to quantify the contribution of changes in disease severity to the burden of cancer, as measured by DALYs. Nevertheless, when interpreting our findings, it is important to acknowledge several limitations. First, it lacked detailed pathological subtype data for cancers such as lung, breast, liver, and oesophageal cancers, which were clinically heterogeneous. As a result, our ability to explore heterogeneity within these cancers was constrained, potentially affecting the accuracy and relevance of our findings. Future studies may explore these subtypes as more comprehensive data become available through improved cancer registries or subsequent GBD updates. Second, regional disparities in quality of GBD data were another concern, particularly in LMICs with underdeveloped health care systems that might not capture accurate cancer data. To address these challenges, the GBD employs advanced statistical models, including Bayesian meta-regression disease modelling (DisMod-MR) and spatiotemporal Gaussian process regression (ST-GPR) disease models. These models impute databased on geographical proximity, temporal trends, and covariate associations. Moving forward, it is essential to strengthen health information systems in low-income countries to enhance the equity and accuracy of global health assessments. Third, beyond its inability to infer causality, the decomposition analysis may not account for unmeasured confounders. These confounders include lifestyle changes (*e.g*. increased smoking cessation, improved diet, and reduced alcohol consumption) and advancements in health care systems (*e.g*. early detection, improved diagnosis, and enhanced treatment). Additionally, the expansion of palliative care services, observed in 82% of countries in the WHO European Region between 2005 and 2019 [53], may reduce DALYs by improving quality of life in advanced cancer stages. Furthermore, due to the constraints of the decomposition framework, our results are based on deterministic calculations, similar to some prior GBD studies [54–58]. Consequently, confidence intervals for the results were not estimated. These limitations may be addressed through future methodological advancements, providing a more comprehensive understanding of the factors driving changes in cancer burden.

## CONCLUSIONS

In conclusion, our secondary analysis of the GBD database revealed a significant decrease in neoplasms-related DALYs globally due to changes in disease severity between 1990 and 2021, with a greater decrease observed in men than in women. Additionally, cancer-related DALYs demonstrated significant heterogeneity in the impact of disease severity changes, with marked regional variations and substantial differences observed among various cancer types. These findings offer valuable insights for policymakers to enhance local cancer control strategies, thereby improving equity in cancer care and contributing to a world where cancer in preventable and every survivor remains healthy.


Online Supplementary Document

## References

[1] Bray F Laversanne M Weiderpass E Soerjomataram I The ever-increasing importance of cancer as a leading cause of premature death worldwide. Cancer. 2021;127:3029–30. 10.1002/cncr.33587 34086348

[2] Bray F Laversanne M Sung H Ferlay J Siegel RL Soerjomataram I Global cancer statistics 2022: GLOBOCAN estimates of incidence and mortality worldwide for 36 cancers in 185 countries. CA Cancer J Clin. 2024;74:229–63. 10.3322/caac.21834 38572751

[3] Chen S Cao Z Prettner K Kuhn M Yang J Jiao L Estimates and Projections of the Global Economic Cost of 29 Cancers in 204 Countries and Territories From 2020 to 2050. JAMA Oncol. 2023;9:465–72. 10.1001/jamaoncol.2022.7826 36821107 PMC9951101

[4] Tu Z Liao S Chen C Li C Hu Q Cai C The long-term spatiotemporal trends in lung cancer burden and its risk factors at global, regional, and national levels, 1992-2021: The Global Burden of Disease Study 2021. Cancer Commun (Lond). 2024;44:1418–21. 10.1002/cac2.12622 39422330 PMC12015974

[5] Huang DQ Singal AG Kono Y Tan DJH El-Serag HB Loomba R Changing global epidemiology of liver cancer from 2010 to 2019: NASH is the fastest growing cause of liver cancer. Cell Metab. 2022;34:969–77.e2. 10.1016/j.cmet.2022.05.003 35793659 PMC9762323

[6] Yi M Li T Niu M Luo S Chu Q Wu K Epidemiological trends of women’s cancers from 1990 to 2019 at the global, regional, and national levels: a population-based study. Biomark Res. 2021;9:55. 10.1186/s40364-021-00310-y 34233747 PMC8261911

[7] Das Gupta P. Standardization and decomposition of rates: a user's manual. Washington, DC, USA: U.S. Dept. of Commerce, Economics and Statistics Administration, Bureau of the Census: 1993.

[8] Chang AY Skirbekk VF Tyrovolas S Kassebaum NJ Dieleman JL Measuring population ageing: an analysis of the Global Burden of Disease Study 2017. Lancet Public Health. 2019;4:e159–67. 10.1016/S2468-2667(19)30019-2 30851869 PMC6472541

[9] United Nations. Ensure healthy lives and promote well-being for all at all ages. 2015. Available: https://sdgs.un.org/goals/goal3#targets_and_indicators. Accessed: 25 December 2024.

[10] GBD 2021 Diseases and Injuries Collaborators Global incidence, prevalence, years lived with disability (YLDs), disability-adjusted life-years (DALYs), and healthy life expectancy (HALE) for 371 diseases and injuries in 204 countries and territories and 81 1 subnational locations, 1990-2021: a systematic analysis for the Glob al Burden of Disease Study 2021. Lancet. 2024;403:2133–61. 10.1016/S0140-6736(24)00757-8 38642570 PMC11122111

[11] GBD 2021 Risk Factors Collaborators Global burden and strength of evidence for 88 risk factors in 204 coun tries and 811 subnational locations, 1990-2021: a systematic analysis for the Global Burden of Disease Study 2021. Lancet. 2024;403:2162–203. 10.1016/S0140-6736(24)00933-4 38762324 PMC11120204

[12] GBD 2021 Causes of Death Collaborators Global burden of 288 causes of death and life expectancy decomposition in 204 countries and territories and 811 subnational locations, 1990- 2021: a systematic analysis for the Global Burden of Disease Study 2021. Lancet. 2024;403:2100–32. 10.1016/S0140-6736(24)00367-2 38582094 PMC11126520

[13] Institute for Health Metrics and Evaluation. GBD Results. 2024. Available: https://vizhub.healthdata.org/gbd-results/. Accessed: 20 July 2024.

[14] Institute for Health Metrics and Evaluation (IHME). Global Burden of Disease Study 2021 (GBD 2021) Socio-Demographic Index (SDI) 1950–2021. 2024. Available: https://ghdx.healthdata.org/record/global-burden-disease-study-2021-gbd-2021-socio-demographic-index-sdi-1950%E2%80%932021. Accessed: 25 December 2024.

[15] Institute for Health Metrics and Evaluation (IHME). Global Burden of Disease Study 2021 (GBD 2021) Socio-Demographic Index (SDI) 1950–2021. 2024. Available: https://ghdx.healthdata.org/record/global-burden-disease-study-2021-gbd-2021-socio-demographic-index-sdi-1950%E2%80%932021. Accessed: 20 July 2024.

[16] GBD 2015 Risk Factors Collaborators Global, regional, and national comparative risk assessment of 79 behavioural, environmental and occupational, and metabolic risks or clusters of risks, 1990-2015: a systematic analysis for the Global Burden of Disease Study 2015. Lancet. 2016;388:1659–724. 10.1016/S0140-6736(16)31679-8 27733284 PMC5388856

[17] Chen J Chen X Zhu Y Li Z Chen X Cao X Quantifying the impact of disease severity changes on the burden of blindness: A global decomposition analysis. J Glob Health. 2024;14:04248. 10.7189/jogh.14.04248 39485018 PMC11529148

[18] Fitzmaurice C Abate D Abbasi N Abbastabar H Abd-Allah F Abdel-Rahman O Global, Regional, and National Cancer Incidence, Mortality, Years of Life Lost, Years Lived With Disability, and Disability-Adjusted Life-Years for 29 Cancer Groups, 1990 to 2017: A Systematic Analysis for the Global Burden of Disease Study. JAMA Oncol. 2019;5:1749–68. 10.1001/jamaoncol.2019.2996 31560378 PMC6777271

[19] Smith GL Lopez-Olivo MA Advani PG Ning MS Geng Y Giordano SH Financial Burdens of Cancer Treatment: A Systematic Review of Risk Factors and Outcomes. J Natl Compr Canc Netw. 2019;17:1184–92. 10.6004/jnccn.2019.7305 31590147 PMC7370695

[20] Gelband H Sankaranarayanan R Gauvreau CL Horton S Anderson BO Bray F Costs, affordability, and feasibility of an essential package of cancer control interventions in low-income and middle-income countries: key messages from Disease Control Priorities, 3rd edition. Lancet. 2016;387:2133–44. 10.1016/S0140-6736(15)00755-2 26578033

[21] Plevritis SK Munoz D Kurian AW Stout NK Alagoz O Near AM Association of Screening and Treatment With Breast Cancer Mortality by Molecular Subtype in US Women, 2000-2012. JAMA. 2018;319:154–64. 10.1001/jama.2017.19130 29318276 PMC5833658

[22] Kanth P Inadomi JM Screening and prevention of colorectal cancer. BMJ. 2021;374:n1855. 10.1136/bmj.n1855 34526356

[23] Liou JM Malfertheiner P Lee YC Sheu BS Sugano K Cheng HC Screening and eradication of Helicobacter pylori for gastric cancer prevention: the Taipei global consensus. Gut. 2020;69:2093–112. 10.1136/gutjnl-2020-322368 33004546

[24] Tachihara M Tsujino K Ishihara T Hayashi H Sato Y Kurata T Durvalumab Plus Concurrent Radiotherapy for Treatment of Locally Advanced Non-Small Cell Lung Cancer: The DOLPHIN Phase 2 Nonrandomized Controlled Trial. JAMA Oncol. 2023;9:1505–13. 10.1001/jamaoncol.2023.3309 37676681 PMC10485744

[25] Hanson SE Lei X Roubaud MS DeSnyder SM Caudle AS Shaitelman SF Long-term Quality of Life in Patients With Breast Cancer After Breast Conservation vs Mastectomy and Reconstruction. JAMA Surg. 2022;157:e220631. 10.1001/jamasurg.2022.0631 35416926 PMC9008558

[26] van Maaren MC de Munck L de Bock GH Jobsen JJ van Dalen T Linn SC 10 year survival after breast-conserving surgery plus radiotherapy compared with mastectomy in early breast cancer in the Netherlands: a population-based study. Lancet Oncol. 2016;17:1158–70. 10.1016/S1470-2045(16)30067-5 27344114

[27] Ye Y Jing Y Li L Mills GB Diao L Liu H Sex-associated molecular differences for cancer immunotherapy. Nat Commun. 2020;11:1779. 10.1038/s41467-020-15679-x 32286310 PMC7156379

[28] Haupt S Caramia F Klein SL Rubin JB Haupt Y Sex disparities matter in cancer development and therapy. Nat Rev Cancer. 2021;21:393–407. 10.1038/s41568-021-00348-y 33879867 PMC8284191

[29] World Health Organization. WHO report on the global tobacco epidemic 2021: addressing new and emerging products. Geneva, Switzerland: World Health Organization; 2021. Available: https://www.who.int/publications/i/item/9789240032095. Accessed: 19 May 2025.

[30] Austoker J Sanders D Fowler G Smoking and cancer: smoking cessation. BMJ. 1994;308:1478–82. 10.1136/bmj.308.6942.1478 8019283 PMC2540295

[31] Ongtengco N Thiam H Collins Z De Jesus EL Peterson CE Wang T Role of gender in perspectives of discrimination, stigma, and attitudes relative to cervical cancer in rural Sénégal. PLoS One. 2020;15:e0232291. 10.1371/journal.pone.0232291 32343755 PMC7188246

[32] Morse RM Brown J Gage JC Prieto BA Jurczuk M Matos A “Easy women get it”: pre-existing stigma associated with HPV and cervical cancer in a low-resource setting prior to implementation of an HPV screen-and-treat program. BMC Public Health. 2023;23:2396. 10.1186/s12889-023-17324-w 38042779 PMC10693157

[33] Ramalingam SS Owonikoko TK Khuri FR Lung cancer: New biological insights and recent therapeutic advances. CA Cancer J Clin. 2011;61:91–112. 10.3322/caac.20102 21303969

[34] Dekker E Tanis PJ Vleugels JLA Kasi PM Wallace MB Colorectal cancer. Lancet. 2019;394:1467–80. 10.1016/S0140-6736(19)32319-0 31631858

[35] Smyth EC Nilsson M Grabsch HI van Grieken NC Lordick F Gastric cancer. Lancet. 2020;396:635–48. 10.1016/S0140-6736(20)31288-5 32861308

[36] Li T Li F Guo X Hong C Yu X Wu B Anti-Epstein-Barr Virus BNLF2b for Mass Screening for Nasopharyngeal Cancer. N Engl J Med. 2023;389:808–19. 10.1056/NEJMoa2301496 37646678

[37] Tseng M Ho F Leong YH Wong LC Tham IW Cheo T Emerging radiotherapy technologies and trends in nasopharyngeal cancer. Cancer Commun (Lond). 2020;40:395–405. 10.1002/cac2.12082 32745354 PMC7494066

[38] Chovanec M Cheng L Advances in diagnosis and treatment of testicular cancer. BMJ. 2022;379:e070499. 10.1136/bmj-2022-070499 36442868

[39] Wang TS Sosa JA Thyroid surgery for differentiated thyroid cancer - recent advances and future directions. Nat Rev Endocrinol. 2018;14:670–83. 10.1038/s41574-018-0080-7 30131586

[40] Asciak R George V Rahman NM Update on biology and management of mesothelioma. Eur Respir Rev. 2021;30:200226. 10.1183/16000617.0226-2020 33472960 PMC9489032

[41] Lin RT Chien LC Jimba M Furuya S Takahashi K Implementation of national policies for a total asbestos ban: a global comparison. Lancet Planet Health. 2019;3:e341–8. 10.1016/S2542-5196(19)30109-3 31439315

[42] Peters S Scherpereel A Cornelissen R Oulkhouir Y Greillier L Kaplan MA First-line nivolumab plus ipilimumab versus chemotherapy in patients with unresectable malignant pleural mesothelioma: 3-year outcomes from CheckMate 743. Ann Oncol. 2022;33:488–99. 10.1016/j.annonc.2022.01.074 35124183

[43] World Health Organization. Cervical Cancer Elimination Initiative. 2024. Available: https://www.who.int/initiatives/cervical-cancer-elimination-initiative. Accessed: 25 December 2024.

[44] Brisson M Kim JJ Canfell K Drolet M Gingras G Burger EA Impact of HPV vaccination and cervical screening on cervical cancer elimination: a comparative modelling analysis in 78 low-income and lower-middle-income countries. Lancet. 2020;395:575–90. 10.1016/S0140-6736(20)30068-4 32007141 PMC7043009

[45] Denny L de Sanjose S Mutebi M Anderson BO Kim J Jeronimo J Interventions to close the divide for women with breast and cervical cancer between low-income and middle-income countries and high-income countries. Lancet. 2017;389:861–70. 10.1016/S0140-6736(16)31795-0 27814963

[46] Das M Poor cancer care in Zimbabwe. Lancet Oncol. 2021;22:1504. 10.1016/S1470-2045(21)00576-3 34627501

[47] Zibako P Tsikai N Manyame S Ginindza TG Cervical cancer management in Zimbabwe (2019-2020). PLoS One. 2022;17:e0274884. 10.1371/journal.pone.0274884 36129898 PMC9491541

[48] Beltrán Ponce SE Abunike SA Bikomeye JC Sieracki R Niyonzima N Mulamira P Access to Radiation Therapy and Related Clinical Outcomes in Patients With Cervical and Breast Cancer Across Sub-Saharan Africa: A Systematic Review. JCO Glob Oncol. 2023;9:e2200218. 10.1200/GO.22.00218 36795990 PMC10166435

[49] Nadella P Iyer HS Manirakiza A Vanderpuye V Triedman SA Shulman LN Geographic Accessibility of Radiation Therapy Facilities in Sub-Saharan Africa. Int J Radiat Oncol Biol Phys. 2023;115:557–63. 10.1016/j.ijrobp.2022.10.018 36725167

[50] Yip W Fu H Jian W Liu J Pan J Xu D Universal health coverage in China part 2: addressing challenges and recommendations. Lancet Public Health. 2023;8:e1035–42. 10.1016/S2468-2667(23)00255-4 38000883

[51] Fang H Eggleston K Hanson K Wu M Enhancing financial protection under China’s social health insurance to achieve universal health coverage. BMJ. 2019;365:l2378. 10.1136/bmj.l2378 31227485 PMC6598720

[52] Zhang Y Wushouer H Han S Fu M Guan X Shi L The impacts of government reimbursement negotiation on targeted anticancer medication price, volume and spending in China. BMJ Glob Health. 2021;6:e006196. 10.1136/bmjgh-2021-006196 34266848 PMC8286756

[53] Arias-Casais N López-Fidalgo J Garralda E Pons JJ Rhee JY Lukas R Trends analysis of specialized palliative care services in 51 countries of the WHO European region in the last 14 years. Palliat Med. 2020;34:1044–56. 10.1177/0269216320931341 32519584 PMC7388149

[54] Chen J Chen X Zhu Y Li Z Chen X Cao X Quantifying the impact of disease severity changes on the burden of blindness: A global decomposition analysis. J Glob Health. 2024;14:04248. 10.7189/jogh.14.04248 39485018 PMC11529148

[55] Cao F Xu Z Li XX Fu ZY Han RY Zhang JL Trends and cross-country inequalities in the global burden of osteoarthritis, 1990-2019: A population-based study. Ageing Res Rev. 2024;99:102382. 10.1016/j.arr.2024.102382 38917934

[56] Bai Z Wang H Shen C An J Yang Z Mo X The global, regional, and national patterns of change in the burden of nonmalignant upper gastrointestinal diseases from 1990 to 2019 and the forecast for the next decade. Int J Surg. 2025;111:80–92. 10.1097/JS9.0000000000001902 38959095 PMC11745775

[57] Bai Z Han J An J Wang H Du X Yang Z The global, regional, and national patterns of change in the burden of congenital birth defects, 1990-2021: an analysis of the global burden of disease study 2021 and forecast to 2040. EClinicalMedicine. 2024;77:102873. 10.1016/j.eclinm.2024.102873 39416384 PMC11474384

[58] Liang X Lyu Y Li J Li Y Chi C Global, regional, and national burden of preterm birth, 1990-2021: a systematic analysis from the global burden of disease study 2021. EClinicalMedicine. 2024;76:102840. 10.1016/j.eclinm.2024.102840 39386159 PMC11462015

