# Supplementary material for: Decomposing the global burden of cancers: quantifying the contributions of disease severity changes across regions and time (1990–2021)
Source: J Glob Health. 2025 Jun 2;15:04161. doi: 10.7189/jogh.15.04161 (PMC12127834; doi:10.7189/jogh.15.04161)
Supplement: Online Supplementary Document [file jogh-15-04161-s001.pdf]

Supplement to: Qiu Z, Huang Y, Qiu Z, Guo Z, Wang L, Xu M, Xuan F. Decomposing the global burden of cancers: quantifying the contributions of disease severity changes across regions and time (1990–2021). J Glob Health. 2025;15:04161.

Decomposing the global burden of cancers: Quantifying the contributions of disease severity changes across regions and time (1990–2021)

Zijian Qiu[1], Yitong Huang[2], Zhaoqi Qiu[3], Zeying Guo[4], Liejiong Wang[3], Maoyi Xu[5], Feng Xuan,[3 *]

[1]Department of Radiation Oncology, The Quzhou Affiliated Hospital of Wenzhou Medical University, Quzhou People's Hospital, Quzhou, China

[2]Department of Internal Medicine, Zhuji Maternal and Child Health Hospital, Shaoxing, Zhejiang, China

[3]Department of Radiation Oncology, Zhuji Affiliated Hospital of Wenzhou Medical University, Shaoxing, China.

[4]Department of Medical Oncology, Zhuji Affiliated Hospital of Wenzhou Medical University, Shaoxing, China

[5]Department of Medical Oncology, The Affiliated Hospital of Jiaxing University, Jiaxing, Zhejiang, China.

*** Correspondence:** Feng Xuan

Email: xfeng8901@outlook.com

Index:

Table S1. International Classification of Diseases (ICD) diagnostic criteria for neoplasms and 20 types of cancers in the Global Burden of Disease (GBD) data. 6

Table S2. The Socio-demographic Index (SDI) reference values from the Global Burden of Disease (GBD) data released by the Institute for Health Metrics and Evaluation (IHME) in 2021. 8

Table S3. The Socio-demographic Index (SDI) values from the Global Burden of Disease (GBD) data across 204 countries/territories released by the Institute for Health Metrics and Evaluation (IHME) in 2021. 9

Table S4. Absolute (the number) and relative contribution (the proportion) associated with disease severity changes for neoplasms by sex at global, SDI regional, GBD regional level between 1990 and 2021. 19

Table S5. Absolute (the number) and relative contribution (the proportion) associated with disease severity changes for thyroid cancer (ThyC) by sex at global, SDI regional, GBD regional level between 1990 and 2021. 31

Table S6. Absolute (the number) and relative contribution (the proportion) associated with disease severity changes for testicular cancer (TesC), by sex at global, SDI regional, GBD regional level between 1990 and 2021. 43

Table S7. Absolute (the number) and relative contribution (the proportion) associated with disease severity changes for mesothelioma (Meso), by sex at global, SDI regional, GBD regional level between 1990 and 2021. 54

Table S8. Absolute (the number) and relative contribution (the proportion) associated with disease severity changes for nasopharynx cancer (NPC) by sex at global, SDI regional, GBD regional level between 1990 and 2021. 66

Table S9. Absolute (the number) and relative contribution (the proportion) associated with disease severity changes for tracheal, bronchus, and lung cancer (TBLC), by sex at

global, SDI regional, GBD regional level between 1990 and 2021. 78

Table S10. Absolute (the number) and relative contribution (the proportion) associated with disease severity changes for stomach cancer (SC), by sex at global, SDI regional, GBD regional level between 1990 and 2021. 91

Table S11. Absolute (the number) and relative contribution (the proportion) associated with disease severity changes for cervical cancer (CC), by sex at global, SDI regional, GBD regional level between 1990 and 2021. 104

Table S12. Absolute (the number) and relative contribution (the proportion) associated with disease severity changes for prostate cancer (ProC), by sex at global, SDI regional, GBD regional level between 1990 and 2021. 115

Table S13. Absolute (the number) and relative contribution (the proportion) associated with disease severity changes for breast cancer (BreC), by sex at global, SDI regional, GBD regional level between 1990 and 2021. 126

Table S14. Absolute (the number) and relative contribution (the proportion) associated with disease severity changes for liver cancer (LivC), by sex at global, SDI regional, GBD regional level between 1990 and 2021. 137

Table S15. Absolute (the number) and relative contribution (the proportion) associated with disease severity changes for pancreatic cancer (PanC), by sex at global, SDI regional, GBD regional level between 1990 and 2021. 149

Table S16. Absolute (the number) and relative contribution (the proportion) associated with disease severity changes for kidney cancer (KC), by sex at global, SDI regional, GBD regional level between 1990 and 2021. 161

Table S17. Absolute (the number) and relative contribution (the proportion) associated with disease severity changes for colon and rectum cancer (CRC), by sex at global, SDI regional, GBD regional level between 1990 and 2021. 173

Table S18. Absolute (the number) and relative contribution (the proportion) associated with disease severity changes for oesophageal cancer (EC), by sex at global, SDI regional, GBD regional level between 1990 and 2021. 187

Table S19. Absolute (the number) and relative contribution (the proportion) associated with disease severity changes for lip and oral cavity cancer (LOC) by sex at global, SDI regional, GBD regional level between 1990 and 2021. 200

Table S20. Absolute (the number) and relative contribution (the proportion) associated with disease severity changes for larynx cancer (LarC) by sex at global, SDI regional, GBD regional level between 1990 and 2021. 212

Table S21. Absolute (the number) and relative contribution (the proportion) associated with disease severity changes for Gallbladder and biliary tract cancer (GBTC), by sex at global, SDI regional, GBD regional level between 1990 and 2021. 224

Table S22. Absolute (the number) and relative contribution (the proportion) associated with disease severity changes for bladder cancer (BlaC), by sex at global, SDI regional, GBD regional level between 1990 and 2021. 236

Table S23. Absolute (the number) and relative contribution (the proportion) associated with disease severity changes for ovarian cancer (OC), by sex at global, SDI regional, GBD regional level between 1990 and 2021. 248

Table S24. Absolute (the number) and relative contribution (the proportion) associated with disease severity changes for uterine cancer (UC), by sex at global, SDI regional, GBD regional level between 1990 and 2021. 259

Table S25. Number of countries and territories with different relative contribution (the proportion) levels associated with disease severity changes for 20 types of cancer by sex at national level between 1990 and 2021. 270

Figure S1. Changes in neoplasms-related DALYs attributable to disease severity in males and females from 1990 to 2021 with 1990 as the reference for each year. 274

Figure S2. Relative contribution (the proportion %) associated with disease severity changes for 16 types of cancer in male between 1990 and 2021 at global, GBD regional, and SDI regional levels. 275

Figure S3. Relative contribution (the proportion %) associated with disease severity changes for 18 types of cancer in female between 1990 and 2021 at global, GBD regional, and SDI regional levels. 276

Figure S4. Absolute contribution (the number) associated with disease severity changes for 16 types of cancer in male between 1990 and 2021 at global, GBD regional, and SDI regional levels. 277

Figure S5. Absolute contribution (the number) associated with disease severity changes for 18 types of cancer in female between 1990 and 2021 at global, GBD regional, and SDI regional levels. 278

Figure S6. Absolute contribution (the number) associated with disease severity changes across 21 GBD regions between 1990 and 2021. 279

Figure S7. Relative contribution (the proportion %) associated with disease severity changes across 21 GBD regions between 1990 and 2021 280

Figure S8. Changes in neoplasms-related DALYs attributable to disease severity between 1990 and 2021 at national levels. 281

R codes 282

Table S1. International Classification of Diseases (ICD) diagnostic criteria for neoplasms and 20 types of cancers in the Global Burden of Disease (GBD) data.

Cancers	Abbreviation	ICD10	ICD9
Neoplasms		C00-C13.9, C15-C22.8, C23-C25.9, C30-C34.9, C37-C38.8, C40-C41.9, C43-C45.9, C47-C54.9, C56-C57.8, C60-C63.8, C64-C67.9, C68.0-C68.8, C69.0-C69.8, C70- C73.9, C75-C75.8, C81-C86.6, C88-C91.0, C91.2-C91.3, C91.6, C92-C92.6, C93-C93.1, C93.3, C93.8, C94-C96.9, D00.1-D00.2, D01.0-D01.3, D02.0-D02.3, D03- D06.9, D07.0-D07.2, D07.4-D07.5, D09.0, D09.2-D09.3, D09.8, D10.0-D10.7, D11- D12.9, D13.0-D13.7, D14.0-D14.3, D15-D16.9, D22-D24.9, D26.0-D27.9, D28.0-D28.1, D28.7, D29.0-D29.8, D30.0-D30.8, D31-D36, D36.1-D36.7, D37.1-D37.5, D38.0-D38.5, D39.1-D39.2, D39.8, D40.0-D40.8, D41.0-D41.8, D42-D43.9, D44.0- D44.8, D45-D47.9, D48.0-D48.6, D49.2-D49.4, D49.6, K62.0-K62.1, K63.5, N60-N60.9, N84.0-N84.1, N87-N87.9	140-148.9, 150-155.1, 155.3-158.9, 160-164.9, 170-175.9, 180-180.9, 182-183.8, 184.0-184.4, 184.8, 185-186.9, 187.1-187.8, 188-188.9, 189.0-189.8, 190-190.8, 191-193.9, 194.1-194.8, 200-204.0, 204.2, 205-205.3, 206-206.1, 207-208.9, 209.0-209.1, 209.4-209.5, 210.0-210.9, 211.0-211.8, 212.0-212.8, 213-213.9, 217-217.8, 219.0, 220-220.9, 221.0-221.8, 222.0-222.8, 223.0-223.8, 224-228.9, 229.0, 229.8, 230.1-230.8, 231.0-231.2, 232-232.9, 233.0-233.2, 233.4-233.5, 233.7, 234.0-234.8, 235.0,235.4, 235.6-235.8, 236.1-236.2, 236.4-236.5, 236.7, 237-237.3, 237.5-237.9, 238.0-238.9, 239.2-239.4, 239.6, 569.0, 610-610.9, 622.1-622.2, 622.7
Nasopharynx cancer	NPC	C11-C11.9, D10.6	147-147.9, 210.7-210.9
Larynx cancer	LarC	C32-C32.9, D02.0, D14.1, D38.0	161-161.9, 212.1, 231.0, 235.6
Lip and oral cavity cancer	LOC	C00-C08.9, D10.0-D10.5, D11-D11.9	140-145.9, 210.0-210.6, 235.0
Thyroid cancer	ThyC	C73-C73.9, D09.3, D09.8, D34-D34.9, D44.0	193-193.9, 226-226.9
Tracheal, bronchus, and lung cancer	TBLC	C33-C34.9, D02.1-D02.3, D14.2-D14.3, D38.1	162-162.9, 212.2-212.3, 231.1-231.2, 235.7
Mesothelioma	Meso	C45-C45.9	

Cancers	Abbreviation	ICD10	ICD9
Oesophageal cancer	EC	C15-C15.9, D00.1, D13.0	150-150.9, 211.0, 230.1
Stomach cancer	SC	C16-C16.9, D00.2, D13.1, D37.1	151-151.9, 211.1, 230.2
Colon and rectum cancer	CRC	C18-C21.9, D01.0-D01.3, D12-D12.9, D37.3-D37.5	153-154.9, 209.1, 209.5, 211.3-211.4, 230.3-230.6, 569.0
Liver cancer	LivC	C22-C22.8, D13.4	155-155.1, 155.3-155.9, 211.5
Gallbladder and biliary tract cancer	GBTC	C23-C24.9, D13.5	156-156.9
Pancreatic cancer	PanC	C25-C25.9, D13.6-D13.7	157-157.9, 211.6-211.7
Kidney cancer	KC	C64-C65.9, D30.0-D30.1, D41.0-D41.1	189.0-189.1, 189.5-189.6, 223.0-223.1
Bladder cancer	BlaC	C67-C67.9, D09.0, D30.3, D41.4-D41.8, D49.4	188-188.9, 223.3, 233.7, 236.7, 239.4
Prostate cancer	ProC	C61-C61.9, D07.5, D29.1, D40.0	185-185.9, 222.2, 236.5
Testicular cancer	TesC	C62-C62.9, D29.2-D29.8, D40.1-D40.8	186-186.9, 222.0, 222.3, 236.4
Breast cancer	BreC	C50-C50.9, D05-D05.9, D24-D24.9, D48.6, D49.3	174-175.9, 217-217.8, 233.0, 238.3, 239.3, 610-610.9
Ovarian cancer	OC	C56-C56.9, D27-D27.9, D39.1	183-183.0, 220-220.9, 236.2
Cervical cancer	CC	C53-C53.9, D06-D06.9, D26.0	180-180.9, 219.0, 233.1, 622.1-622.2, 622.7
Uterine cancer	UC	C54-C54.9, D07.0-D07.2, D26.1-D26.9	182-182.9, 233.2

List of International Classification of Diseases (ICD) codes mapped to the Global Burden of Diseases cause list for causes of death.

Table S2. The Socio-demographic Index (SDI) reference values from the Global Burden of Disease (GBD) data released by the Institute for Health Metrics and Evaluation (IHME) in 2021.

location id	SDI region	lower bound	upper bound
44637	Low SDI	0	0.46581580319161997
44636	Low-middle SDI	0.46581580319161997	0.6188294452454329
44639	Middle SDI	0.6188294452454329	0.7119746219361235
44634	High-middle SDI	0.7119746219361235	0.8102959891918925
44635	High SDI	0.8102959891918925	1

Table S3. The Socio-demographic Index (SDI) values from the Global Burden of Disease (GBD) data across 204 countries/territories released by the Institute for Health Metrics and Evaluation (IHME) in 2021.

Location	GBD region	GBD super region	SDI	SDI quitile
Somalia	Eastern Sub-Saharan Africa	Sub-Saharan Africa	0.081	Low SDI
Niger	Western Sub-Saharan Africa	Sub-Saharan Africa	0.162	Low SDI
Chad	Western Sub-Saharan Africa	Sub-Saharan Africa	0.238	Low SDI
Burkina Faso	Western Sub-Saharan Africa	Sub-Saharan Africa	0.257	Low SDI
Mali	Western Sub-Saharan Africa	Sub-Saharan Africa	0.263	Low SDI
Central African Republic	Central Sub-Saharan Africa	Sub-Saharan Africa	0.274	Low SDI
Burundi	Eastern Sub-Saharan Africa	Sub-Saharan Africa	0.284	Low SDI
Mozambique	Eastern Sub-Saharan Africa	Sub-Saharan Africa	0.307	Low SDI
Guinea	Western Sub-Saharan Africa	Sub-Saharan Africa	0.325	Low SDI
Afghanistan	North Africa and Middle East	North Africa and Middle East	0.343	Low SDI
Ethiopia	Eastern Sub-Saharan Africa	Sub-Saharan Africa	0.343	Low SDI
Sierra Leone	Western Sub-Saharan Africa	Sub-Saharan Africa	0.347	Low SDI
Benin	Western Sub-Saharan Africa	Sub-Saharan Africa	0.352	Low SDI
Guinea-Bissau	Western Sub-Saharan Africa	Sub-Saharan Africa	0.355	Low SDI
South Sudan	Eastern Sub-Saharan Africa	Sub-Saharan Africa	0.363	Low SDI
Liberia	Western Sub-Saharan Africa	Sub-Saharan Africa	0.37	Low SDI
Democratic Republic of the Congo	Central Sub-Saharan Africa	Sub-Saharan Africa	0.382	Low SDI
Malawi	Eastern Sub-Saharan Africa	Sub-Saharan Africa	0.384	Low SDI
Senegal	Western Sub-Saharan Africa	Sub-Saharan Africa	0.389	Low SDI

Location	GBD region	GBD super region	SDI	SDI quitile
Papua New Guinea	Oceania	Southeast Asia, east Asia, and Oceania	0.394	Low SDI
Eritrea	Eastern Sub-Saharan Africa	Sub-Saharan Africa	0.396	Low SDI
Madagascar	Eastern Sub-Saharan Africa	Sub-Saharan Africa	0.396	Low SDI
Gambia	Western Sub-Saharan Africa	Sub-Saharan Africa	0.399	Low SDI
Uganda	Eastern Sub-Saharan Africa	Sub-Saharan Africa	0.404	Low SDI
Solomon Islands	Oceania	Southeast Asia, east Asia, and Oceania	0.407	Low SDI
Coted'Ivoire	Western Sub-Saharan Africa	Sub-Saharan Africa	0.408	Low SDI
Yemen	North Africa and Middle East	North Africa and Middle East	0.412	Low SDI
Togo	Western Sub-Saharan Africa	Sub-Saharan Africa	0.417	Low SDI
Nepal	South Asia	South Asia	0.422	Low SDI
United Republic of Tanzania	Eastern Sub-Saharan Africa	Sub-Saharan Africa	0.423	Low SDI
Rwanda	Eastern Sub-Saharan Africa	Sub-Saharan Africa	0.429	Low SDI
Haiti	Caribbean	Latin America and Caribbean	0.432	Low SDI
Pakistan	South Asia	South Asia	0.449	Low SDI
Bhutan	South Asia	South Asia	0.455	Low-middle SDI
Comoros	Eastern Sub-Saharan Africa	Sub-Saharan Africa	0.455	Low-middle SDI
Djibouti	Eastern Sub-Saharan Africa	Sub-Saharan Africa	0.459	Low-middle SDI
Cambodia	Southeast Asia	Southeast Asia, east Asia, and Oceania	0.469	Low-middle SDI
Angola	Central Sub-Saharan Africa	Sub-Saharan Africa	0.47	Low-middle SDI
Zimbabwe	Southern Sub-Saharan Africa	Sub-Saharan Africa	0.476	Low-middle SDI
Bangladesh	South Asia	South Asia	0.483	Low-middle SDI

Location	GBD region	GBD super region	SDI	SDI quitile
Vanuatu	Oceania	Southeast Asia, east Asia, and Oceania	0.485	Low-middle SDI
Cameroon	Western Sub-Saharan Africa	Sub-Saharan Africa	0.49	Low-middle SDI
Lao People's Democratic Republic	Southeast Asia	Southeast Asia, east Asia, and Oceania	0.49	Low-middle SDI
Honduras	Central Latin America	Latin America and Caribbean	0.496	Low-middle SDI
Mauritania	Western Sub-Saharan Africa	Sub-Saharan Africa	0.496	Low-middle SDI
Sao Tome and Principe	Western Sub-Saharan Africa	Sub-Saharan Africa	0.502	Low-middle SDI
Zambia	Eastern Sub-Saharan Africa	Sub-Saharan Africa	0.505	Low-middle SDI
Lesotho	Southern Sub-Saharan Africa	Sub-Saharan Africa	0.507	Low-middle SDI
Kenya	Eastern Sub-Saharan Africa	Sub-Saharan Africa	0.508	Low-middle SDI
Timor-Leste	Southeast Asia	Southeast Asia, east Asia, and Oceania	0.514	Low-middle SDI
Nigeria	Western Sub-Saharan Africa	Sub-Saharan Africa	0.515	Low-middle SDI
Sudan	North Africa and Middle East	North Africa and Middle East	0.515	Low-middle SDI
Nicaragua	Central Latin America	Latin America and Caribbean	0.517	Low-middle SDI
Myanmar	Southeast Asia	Southeast Asia, east Asia, and Oceania	0.521	Low-middle SDI
Cabo Verde	Western Sub-Saharan Africa	Sub-Saharan Africa	0.525	Low-middle SDI
Guatemala	Central Latin America	Latin America and Caribbean	0.526	Low-middle SDI
Kiribati	Oceania	Southeast Asia, east Asia, and Oceania	0.527	Low-middle SDI
Tajikistan	Central Asia	Central Europe, eastern Europe, and central Asia	0.539	Low-middle SDI
Marshall Islands	Oceania	Southeast Asia, east Asia, and Oceania	0.544	Low-middle SDI
Morocco	North Africa and Middle East	North Africa and Middle East	0.548	Low-middle SDI
Ghana	Western Sub-Saharan Africa	Sub-Saharan Africa	0.557	Low-middle SDI

Location	GBD region	GBD super region	SDI	SDI quitile
Democratic People's Republic of Korea	East Asia	Southeast Asia, east Asia, and Oceania	0.558	Low-middle SDI
Maldives	Southeast Asia	Southeast Asia, east Asia, and Oceania	0.562	Low-middle SDI
Bolivia (Plurinational State of)	Andean Latin America	Latin America and Caribbean	0.566	Low-middle SDI
India	South Asia	South Asia	0.566	Low-middle SDI
Congo	Central Sub-Saharan Africa	Sub-Saharan Africa	0.568	Low-middle SDI
El Salvador	Central Latin America	Latin America and Caribbean	0.573	Low-middle SDI
Eswatini	Southern Sub-Saharan Africa	Sub-Saharan Africa	0.577	Low-middle SDI
Micronesia (Federated States of)	Oceania	Southeast Asia, east Asia, and Oceania	0.58	Low-middle SDI
Palestine	North Africa and Middle East	North Africa and Middle East	0.588	Low-middle SDI
Tuvalu	Oceania	Southeast Asia, east Asia, and Oceania	0.589	Low-middle SDI
Dominican Republic	Caribbean	Latin America and Caribbean	0.592	Low-middle SDI
Kyrgyzstan	Central Asia	Central Europe, eastern Europe, and central Asia	0.596	Low-middle SDI
Belize	Caribbean	Latin America and Caribbean	0.603	Low-middle SDI
Mongolia	Central Asia	Central Europe, eastern Europe, and central Asia	0.606	Low-middle SDI
Venezuela (Bolivarian Republic of)	Central Latin America	Latin America and Caribbean	0.607	Low-middle SDI
Namibia	Southern Sub-Saharan Africa	Sub-Saharan Africa	0.612	Middle SDI
Viet Nam	Southeast Asia	Southeast Asia, east Asia, and Oceania	0.617	Middle SDI
Guyana	Caribbean	Latin America and Caribbean	0.618	Middle SDI
Nauru	Oceania	Southeast Asia, east Asia, and Oceania	0.618	Middle SDI
Syrian Arab Republic	North Africa and Middle East	North Africa and Middle East	0.619	Middle SDI
Philippines	Southeast Asia	Southeast Asia, east Asia, and Oceania	0.623	Middle SDI

Location	GBD region	GBD super region	SDI	SDI quitile
Tokelau	Oceania	Southeast Asia, east Asia, and Oceania	0.626	Middle SDI
Saint Vincent and the Grenadines	Caribbean	Latin America and Caribbean	0.627	Middle SDI
Uzbekistan	Central Asia	Central Europe, eastern Europe, and central Asia	0.631	Middle SDI
Colombia	Central Latin America	Latin America and Caribbean	0.633	Middle SDI
Botswana	Southern Sub-Saharan Africa	Sub-Saharan Africa	0.634	Middle SDI
Suriname	Caribbean	Latin America and Caribbean	0.636	Middle SDI
Tonga	Oceania	Southeast Asia, east Asia, and Oceania	0.636	Middle SDI
Paraguay	Tropical Latin America	Latin America and Caribbean	0.638	Middle SDI
Brazil	Tropical Latin America	Latin America and Caribbean	0.64	Middle SDI
Ecuador	Andean Latin America	Latin America and Caribbean	0.64	Middle SDI
Samoa	Oceania	Southeast Asia, east Asia, and Oceania	0.641	Middle SDI
Peru	Andean Latin America	Latin America and Caribbean	0.648	Middle SDI
Mexico	Central Latin America	Latin America and Caribbean	0.649	Middle SDI
Algeria	North Africa and Middle East	North Africa and Middle East	0.652	Middle SDI
Gabon	Central Sub-Saharan Africa	Sub-Saharan Africa	0.656	Middle SDI
Egypt	North Africa and Middle East	North Africa and Middle East	0.658	Middle SDI
Indonesia	Southeast Asia	Southeast Asia, east Asia, and Oceania	0.66	Middle SDI
Fiji	Oceania	Southeast Asia, east Asia, and Oceania	0.664	Middle SDI
Cuba	Caribbean	Latin America and Caribbean	0.668	Middle SDI
Grenada	Caribbean	Latin America and Caribbean	0.669	Middle SDI
Iran (Islamic Republic of)	North Africa and Middle East	North Africa and Middle East	0.67	Middle SDI

Location	GBD region	GBD super region	SDI	SDI quitile
Saint Lucia	Caribbean	Latin America and Caribbean	0.67	Middle SDI
Turkmenistan	Central Asia	Central Europe, eastern Europe, and central Asia	0.67	Middle SDI
Iraq	North Africa and Middle East	North Africa and Middle East	0.671	Middle SDI
Tunisia	North Africa and Middle East	North Africa and Middle East	0.672	Middle SDI
South Africa	Southern Sub-Saharan Africa	Sub-Saharan Africa	0.678	Middle SDI
Costa Rica	Central Latin America	Latin America and Caribbean	0.68	Middle SDI
Albania	Central Europe	Central Europe, eastern Europe, and central Asia	0.681	Middle SDI
Azerbaijan	Central Asia	Central Europe, eastern Europe, and central Asia	0.683	Middle SDI
Jamaica	Caribbean	Latin America and Caribbean	0.684	Middle SDI
Equatorial Guinea	Central Sub-Saharan Africa	Sub-Saharan Africa	0.685	Middle SDI
China	East Asia	Southeast Asia, east Asia, and Oceania	0.686	Middle SDI
Panama	Central Latin America	Latin America and Caribbean	0.686	Middle SDI
Thailand	Southeast Asia	Southeast Asia, east Asia, and Oceania	0.687	Middle SDI
Armenia	Central Asia	Central Europe, eastern Europe, and central Asia	0.689	Middle SDI
Sri Lanka	Southeast Asia	Southeast Asia, east Asia, and Oceania	0.69	High-middle SDI
Republic of Moldova	Eastern Europe	Central Europe, eastern Europe, and central Asia	0.696	High-middle SDI
Uruguay	Southern Latin America	Latin America and Caribbean	0.697	High-middle SDI
Georgia	Central Asia	Central Europe, eastern Europe, and central Asia	0.702	High-middle SDI
Mauritius	Southeast Asia	Southeast Asia, east Asia, and Oceania	0.705	High-middle SDI
Argentina	Southern Latin America	Latin America and Caribbean	0.708	High-middle SDI
Lebanon	North Africa and Middle East	North Africa and Middle East	0.708	High-middle SDI

Location	GBD region	GBD super region	SDI	SDI quitile
Libya	North Africa and Middle East	North Africa and Middle East	0.709	High-middle SDI
Niue	Oceania	Southeast Asia, east Asia, and Oceania	0.711	High-middle SDI
American Samoa	Oceania	Southeast Asia, east Asia, and Oceania	0.712	High-middle SDI
Bosnia and Herzegovina	Central Europe	Central Europe, eastern Europe, and central Asia	0.718	High-middle SDI
Kazakhstan	Central Asia	Central Europe, eastern Europe, and central Asia	0.723	High-middle SDI
Seychelles	Southeast Asia	Southeast Asia, east Asia, and Oceania	0.724	High-middle SDI
Dominica	Caribbean	Latin America and Caribbean	0.729	High-middle SDI
Jordan	North Africa and Middle East	North Africa and Middle East	0.731	High-middle SDI
Ukraine	Eastern Europe	Central Europe, eastern Europe, and central Asia	0.736	High-middle SDI
Malaysia	Southeast Asia	Southeast Asia, east Asia, and Oceania	0.737	High-middle SDI
Palau	Oceania	Southeast Asia, east Asia, and Oceania	0.738	High-middle SDI
Barbados	Caribbean	Latin America and Caribbean	0.742	High-middle SDI
Antigua and Barbuda	Caribbean	Latin America and Caribbean	0.743	High-middle SDI
Portugal	Western Europe	High income	0.743	High-middle SDI
North Macedonia	Central Europe	Central Europe, eastern Europe, and central Asia	0.744	High-middle SDI
Belarus	Eastern Europe	Central Europe, eastern Europe, and central Asia	0.745	High-middle SDI
Saint Kitts and Nevis	Caribbean	Latin America and Caribbean	0.746	High-middle SDI
Turkey	North Africa and Middle East	North Africa and Middle East	0.748	High-middle SDI
Bahrain	North Africa and Middle East	North Africa and Middle East	0.751	High-middle SDI
Trinidad and Tobago	Caribbean	Latin America and Caribbean	0.757	High-middle SDI
Chile	Southern Latin America	Latin America and Caribbean	0.759	High-middle SDI

Location	GBD region	GBD super region	SDI	SDI quitile
Romania	Central Europe	Central Europe, eastern Europe, and central Asia	0.76	High-middle SDI
Greenland	High-income North America	High income	0.761	High-middle SDI
Bulgaria	Central Europe	Central Europe, eastern Europe, and central Asia	0.764	High-middle SDI
Cook Islands	Oceania	Southeast Asia, east Asia, and Oceania	0.764	High-middle SDI
Serbia	Central Europe	Central Europe, eastern Europe, and central Asia	0.767	High-middle SDI
Spain	Western Europe	High income	0.767	High-middle SDI
Northern Mariana Islands	Oceania	Southeast Asia, east Asia, and Oceania	0.771	High-middle SDI
Oman	North Africa and Middle East	North Africa and Middle East	0.783	High-middle SDI
Hungary	Central Europe	Central Europe, eastern Europe, and central Asia	0.791	High-middle SDI
Montenegro	Central Europe	Central Europe, eastern Europe, and central Asia	0.791	High-middle SDI
Croatia	Central Europe	Central Europe, eastern Europe, and central Asia	0.794	High-middle SDI
Greece	Western Europe	High income	0.794	High-middle SDI
Bahamas	Caribbean	Latin America and Caribbean	0.796	High-middle SDI
United States Virgin Islands	Caribbean	Latin America and Caribbean	0.799	High-middle SDI
Italy	Western Europe	High income	0.801	High-middle SDI
Malta	Western Europe	High income	0.801	High-middle SDI
Poland	Central Europe	Central Europe, eastern Europe, and central Asia	0.802	High-middle SDI
Israel	Western Europe	High income	0.803	High-middle SDI
Russian Federation	Eastern Europe	Central Europe, eastern Europe, and central Asia	0.805	High-middle SDI
Saudi Arabia	North Africa and Middle East	North Africa and Middle East	0.805	High-middle SDI
Slovakia	Central Europe	Central Europe, eastern Europe, and central Asia	0.812	High SDI

Location	GBD region	GBD super region	SDI	SDI quitile
Bermuda	Caribbean	Latin America and Caribbean	0.813	High SDI
Guam	Oceania	Southeast Asia, east Asia, and Oceania	0.813	High SDI
Puerto Rico	Caribbean	Latin America and Caribbean	0.814	High SDI
Latvia	Eastern Europe	Central Europe, eastern Europe, and central Asia	0.82	High SDI
Brunei Darussalam	High-income Asia Pacific	High income	0.823	High SDI
Czechia	Central Europe	Central Europe, eastern Europe, and central Asia	0.828	High SDI
Qatar	North Africa and Middle East	North Africa and Middle East	0.83	High SDI
France	Western Europe	High income	0.834	High SDI
Estonia	Eastern Europe	Central Europe, eastern Europe, and central Asia	0.835	High SDI
Australia	Australasia	High income	0.839	High SDI
New Zealand	Australasia	High income	0.84	High SDI
Slovenia	Central Europe	Central Europe, eastern Europe, and central Asia	0.84	High SDI
Cyprus	Western Europe	High income	0.841	High SDI
Lithuania	Eastern Europe	Central Europe, eastern Europe, and central Asia	0.843	High SDI
United Kingdom	Western Europe	High income	0.847	High SDI
Austria	Western Europe	High income	0.849	High SDI
Belgium	Western Europe	High income	0.851	High SDI
Kuwait	North Africa and Middle East	North Africa and Middle East	0.851	High SDI
Finland	Western Europe	High income	0.856	High SDI
United States of America	High-income North America	High income	0.859	High SDI
Singapore	High-income Asia Pacific	High income	0.861	High SDI

**Location**	**GBD region**	**GBD super region**	**SDI**	**SDI quitile**
Ireland	Western Europe	High income	0.867	High SDI
Taiwan (Province of China)	East Asia	Southeast Asia, east Asia, and Oceania	0.868	High SDI
Iceland	Western Europe	High income	0.869	High SDI
Japan	High-income Asia Pacific	High income	0.87	High SDI
Sweden	Western Europe	High income	0.872	High SDI
Canada	High-income North America	High income	0.873	High SDI
Republic of Korea	High-income Asia Pacific	High income	0.878	High SDI
United Arab Emirates	North Africa and Middle East	North Africa and Middle East	0.88	High SDI
Netherlands	Western Europe	High income	0.883	High SDI
San Marino	Western Europe	High income	0.884	High SDI
Denmark	Western Europe	High income	0.89	High SDI
Andorra	Western Europe	High income	0.894	High SDI
Luxembourg	Western Europe	High income	0.895	High SDI
Germany	Western Europe	High income	0.898	High SDI
Monaco	Western Europe	High income	0.902	High SDI
Norway	Western Europe	High income	0.913	High SDI
Switzerland	Western Europe	High income	0.929	High SDI

Table S4. Absolute (the number) and relative contribution (the proportion) associated with disease severity changes for neoplasms by sex at global, SDI regional, GBD regional level between 1990 and 2021.

Location	Cause	Attributable number (Both thousands)	Attributable proportion (Both %)	Attributable number (Male thousands)	Attributable proportion (Male %)	Attributable number (Female thousands)	Attributable proportion (Female %)
Global	Neoplasms	-81963.31	-52.68	-56172.28	-63.72	-28558.95	-42.35
High SDI	Neoplasms	-30317.14	-67.01	-20164.15	-79.3	-11238.66	-56.72
High-middle SDI	Neoplasms	-26445.21	-56.78	-20128.27	-71.19	-8371.11	-45.74
Middle SDI	Neoplasms	-32958.22	-80.36	-19728.93	-83.86	-13198.93	-75.48
Low-middle SDI	Neoplasms	-5869.18	-37.48	-2538.29	-32.93	-2997.64	-37.69
Low SDI	Neoplasms	-2881.96	-41.62	-1135.3	-36.34	-1660.62	-43.69
High-income Asia Pacific	Neoplasms	-5181.41	-67.7	-3437.67	-73.83	-1850.42	-61.73
High-income North America	Neoplasms	-11472.46	-79.29	-6934.5	-89.68	-4747.9	-70.48
Western Europe	Neoplasms	-12062.35	-52.1	-8906.88	-67.71	-3986.78	-39.87
Australasia	Neoplasms	-586.84	-61.94	-382.16	-72.2	-219.17	-52.41
Eastern Europe	Neoplasms	-5618.11	-45.02	-4297.28	-59.94	-1825.37	-34.37
Central Europe	Neoplasms	-2895.55	-42.46	-2259.61	-56.28	-909.01	-32.41
Southern Latin America	Neoplasms	-1129.96	-56.67	-782.85	-71.81	-393.79	-43.57
East Asia	Neoplasms	-45158.85	-99.71	-29142.72	-103.15	-16643.99	-97.68
Central Asia	Neoplasms	-973.85	-48.61	-621.55	-55.58	-354.06	-39.99
North Africa and Middle East	Neoplasms	-4206.66	-90.11	-2636.38	-95.31	-1496.67	-78.69
Andean Latin America	Neoplasms	-657	-91.53	-353.53	-108.99	-316.18	-80.37
Southeast Asia	Neoplasms	-3783.74	-48.69	-2010.74	-52.4	-1873.74	-47.64

Location	Cause	Attributable number (Both thousands)	Attributable proportion (Both %)	Attributable number (Male thousands)	Attributable proportion (Male %)	Attributable number (Female thousands)	Attributable proportion (Female %)
Tropical Latin America	Neoplasms	-2187.18	-69.07	-1310.84	-77.82	-839.53	-56.65
Southern Sub-Saharan Africa	Neoplasms	-37.71	-3.97	-69.71	-14.69	38.82	8.19
Caribbean	Neoplasms	-430.29	-48.45	-279.19	-62.06	-163.5	-37.3
Central Latin America	Neoplasms	-2328.2	-89.73	-1133.45	-96.31	-1187.16	-83.73
South Asia	Neoplasms	-7014.36	-49.34	-2668.47	-37.4	-3857.02	-54.47
Central Sub-Saharan Africa	Neoplasms	-279.77	-38.31	-129.82	-39.8	-142.09	-35.16
Oceania	Neoplasms	-17.46	-19.52	-10.84	-25.94	-6.58	-13.81
Western Sub-Saharan Africa	Neoplasms	-713.14	-35.79	-307.03	-32.44	-345.83	-33.06
Eastern Sub-Saharan Africa	Neoplasms	-1357.88	-45.22	-528.15	-41.23	-817.96	-47.5
Afghanistan	Neoplasms	-78.02	-29.13	-29.89	-27.86	-46.09	-28.71
Albania	Neoplasms	-33.87	-51.25	-25.53	-60.15	-8.56	-36.21
Algeria	Neoplasms	-137.39	-69.33	-73.9	-73.04	-65.35	-67.38
American Samoa	Neoplasms	-0.15	-17.93	-0.14	-28.33	0.02	5
Andorra	Neoplasms	-2.02	-75.72	-1.56	-80.7	-0.45	-60.32
Angola	Neoplasms	-93.13	-63.12	-39.66	-51.96	-42.22	-59.29
Antigua and Barbuda	Neoplasms	-0.99	-57.82	-0.67	-70.25	-0.31	-40.78
Argentina	Neoplasms	-667.52	-48.08	-459.46	-59.52	-221.9	-36.01
Armenia	Neoplasms	-50.35	-40.21	-30.31	-43.84	-22.7	-40.49
Australia	Neoplasms	-499.04	-63.99	-325.49	-73.58	-184.45	-54.66
Austria	Neoplasms	-250.74	-55.11	-176.84	-75.14	-96.27	-43.83

Location	Cause	Attributable number (Both thousands)	Attributable proportion (Both %)	Attributable number (Male thousands)	Attributable proportion (Male %)	Attributable number (Female thousands)	Attributable proportion (Female %)
Azerbaijan	Neoplasms	-96.6	-50.2	-59.13	-53.45	-37.06	-45.29
Bahamas	Neoplasms	-3.67	-51.32	-2.18	-61.55	-1.5	-41.73
Bahrain	Neoplasms	-14.28	-192.41	-9.29	-213.26	-5.3	-172.78
Bangladesh	Neoplasms	-888.33	-71.37	-425.04	-61.16	-384.57	-69.95
Barbados	Neoplasms	-5.09	-49.76	-2.94	-59.96	-2.23	-41.79
Belarus	Neoplasms	-253.83	-47.75	-204.51	-65.26	-87.03	-39.88
Belgium	Neoplasms	-325.39	-48.09	-238.85	-60.64	-107.87	-38.16
Belize	Neoplasms	-0.81	-39.02	-0.54	-54.12	-0.26	-24.39
Benin	Neoplasms	-19.57	-39.1	-12.64	-49.99	-7.63	-30.79
Bermuda	Neoplasms	-3.09	-99	-2.19	-124.77	-1.04	-76.02
Bhutan	Neoplasms	-2.87	-45.95	-1.31	-43.04	-1.55	-48.25
Bolivia (Plurinational State of)	Neoplasms	-105.83	-66.54	-47.79	-74.07	-59.08	-62.5
Bosnia and Herzegovina	Neoplasms	-52.3	-32.29	-45.48	-46.5	-14.87	-23.18
Botswana	Neoplasms	-7.01	-33.75	-4.47	-41.88	-2.81	-27.85
Brazil	Neoplasms	-2167.47	-69.58	-1299.19	-78.21	-829.05	-57.03
Brunei Darussalam	Neoplasms	-3.99	-67.28	-2.03	-66.04	-1.86	-64.99
Bulgaria	Neoplasms	-109.44	-22.1	-96.37	-32.89	-29.2	-14.44
Burkina Faso	Neoplasms	-37.77	-29.19	-22	-35.71	-17.29	-25.52
Burundi	Neoplasms	-40.13	-39.11	-20.25	-48.11	-22.22	-36.71
Cabo Verde	Neoplasms	-1.69	-28.69	-1.54	-56.47	-1.21	-38.65

**Location**	**Cause**	**Attributable number (Both thousands)**	**Attributable proportion (Both %)**	**Attributable number (Male thousands)**	**Attributable proportion (Male %)**	**Attributable number (Female thousands)**	**Attributable proportion (Female %)**
Cambodia	Neoplasms	-95.45	-54.61	-43.14	-53.58	-52.09	-55.24
Cameroon	Neoplasms	-45.01	-34.93	-22.44	-37.96	-25.86	-37.08
Canada	Neoplasms	-656.08	-49.76	-440.37	-60.65	-233.77	-39.46
Central African Republic	Neoplasms	-10.46	-20.78	-5.45	-22.74	-4.63	-17.57
Chad	Neoplasms	2.17	3.65	-3.48	-12.14	2.87	9.36
Chile	Neoplasms	-364.63	-87.45	-237.72	-114.74	-152.94	-72.91
China	Neoplasms	-44306.96	-100.59	-28570.33	-103.82	-16314.08	-98.71
Colombia	Neoplasms	-755.73	-117.63	-390.68	-126.82	-357.9	-107.03
Comoros	Neoplasms	-3.07	-38.77	-1.17	-38.11	-1.89	-38.97
Congo	Neoplasms	-29.15	-60.54	-14.49	-67.35	-14.13	-53.03
Cook Islands	Neoplasms	-0.31	-72.69	-0.22	-86.18	-0.09	-54.78
Costa Rica	Neoplasms	-57.39	-99.2	-36.78	-121.38	-21.97	-79.75
Coted'Ivoire	Neoplasms	-33.65	-40.11	-23.36	-54.95	-9.38	-22.69
Croatia	Neoplasms	-147.76	-49.76	-123.08	-67.8	-42.3	-36.65
Cuba	Neoplasms	-204.21	-56.52	-140.44	-71.44	-73.9	-44.87
Cyprus	Neoplasms	-29.28	-117.57	-20.11	-145.97	-10.97	-98.63
Czechia	Neoplasms	-467.31	-65.28	-354.39	-84.07	-146.96	-49.93
Democratic People's Republic of Korea	Neoplasms	-170.57	-26.68	-124.79	-35.58	-76.03	-26.34
Democratic Republic of the Congo	Neoplasms	-135.95	-29.8	-61.73	-32.46	-73.58	-27.65

Location	Cause	Attributable number (Both thousands)	Attributable proportion (Both %)	Attributable number (Male thousands)	Attributable proportion (Male %)	Attributable number (Female thousands)	Attributable proportion (Female %)
Denmark	Neoplasms	-193.1	-55.75	-138.67	-78.85	-72.34	-42.42
Djibouti	Neoplasms	-2.6	-48.49	-1.2	-49.4	-1.52	-52.04
Dominica	Neoplasms	-0.47	-20.4	-0.37	-32.43	-0.2	-16.93
Dominican Republic	Neoplasms	-19.24	-22.42	-9.56	-23.45	-11.32	-25.1
Ecuador	Neoplasms	-144.04	-91.87	-66.05	-92.07	-77.28	-90.86
Egypt	Neoplasms	-121.35	-20.39	-36.57	-10.93	-54.53	-20.92
El Salvador	Neoplasms	-47.53	-65.9	-20.95	-68.66	-25.21	-60.58
Equatorial Guinea	Neoplasms	-7.06	-101.69	-3.14	-98.72	-3.75	-99.58
Eritrea	Neoplasms	-22.98	-37.85	-8.87	-38.62	-14.02	-37.11
Estonia	Neoplasms	-55.83	-60.45	-45.7	-88.66	-18.79	-46.03
Eswatini	Neoplasms	3.29	26.56	2.15	33.18	1.22	20.7
Ethiopia	Neoplasms	-645.45	-64.69	-254.88	-58.83	-384.16	-68.05
Fiji	Neoplasms	-2.08	-16.53	-0.85	-18.07	-1.22	-15.4
Finland	Neoplasms	-162.4	-66.67	-122.01	-94.59	-56.06	-48.92
France	Neoplasms	-2218.62	-64.25	-1691.01	-77.44	-606.68	-47.79
Gabon	Neoplasms	-9.18	-43.7	-4.87	-44.39	-4.36	-43.39
Gambia	Neoplasms	-0.55	-6.78	0.17	3.54	0.15	4.45
Georgia	Neoplasms	-24.19	-10.86	-21.56	-17.95	-11.04	-10.76
Germany	Neoplasms	-2608.74	-50.92	-1944.56	-71.79	-934.59	-38.7
Ghana	Neoplasms	-75.01	-43.83	-28.14	-38.09	-44.01	-45.24

Location	Cause	Attributable number (Both thousands)	Attributable proportion (Both %)	Attributable number (Male thousands)	Attributable proportion (Male %)	Attributable number (Female thousands)	Attributable proportion (Female %)
Greece	Neoplasms	-229.5	-40.62	-163.74	-48.22	-78.34	-34.74
Greenland	Neoplasms	-1.93	-58.64	-1.13	-61.96	-0.82	-55.93
Grenada	Neoplasms	-1.39	-51.27	-0.9	-68.93	-0.54	-38.9
Guam	Neoplasms	-0.5	-21.81	-0.36	-25.15	-0.13	-14.93
Guatemala	Neoplasms	-77.72	-78.28	-34.72	-84.05	-40.49	-69.85
Guinea	Neoplasms	-19.22	-20.35	-6.78	-16.53	-12.54	-23.46
Guinea-Bissau	Neoplasms	-5.73	-33.92	-3.27	-37.26	-1.88	-23.09
Guyana	Neoplasms	-3.74	-29.41	-2.19	-38.64	-1.61	-22.85
Haiti	Neoplasms	-65.34	-40.02	-26.82	-40.45	-37.57	-38.73
Honduras	Neoplasms	-13.11	-24.63	-5.81	-28.31	-6.44	-19.69
Hungary	Neoplasms	-347.94	-44.08	-267.69	-57.86	-106.04	-32.46
Iceland	Neoplasms	-5.5	-53.15	-3.61	-69.23	-2.19	-42.67
India	Neoplasms	-5780.44	-52.3	-2055.68	-37.83	-3389.91	-60.35
Indonesia	Neoplasms	-523.48	-19.95	-141.53	-12.44	-404.33	-27.2
Iran (Islamic Republic of)	Neoplasms	-680.75	-108.33	-401.25	-110.53	-269.3	-101.48
Iraq	Neoplasms	-167.55	-84.98	-96.16	-88.98	-67.38	-75.62
Ireland	Neoplasms	-131.41	-75.14	-82.47	-88.55	-53.53	-65.49
Israel	Neoplasms	-115.63	-70.91	-67.36	-83.25	-56.66	-68.98
Italy	Neoplasms	-1713.77	-47.28	-1223.52	-56.41	-535.99	-36.81
Jamaica	Neoplasms	-21.47	-43.34	-15.21	-60.69	-6.75	-27.61

Location	Cause	Attributable number (Both thousands)	Attributable proportion (Both %)	Attributable number (Male thousands)	Attributable proportion (Male %)	Attributable number (Female thousands)	Attributable proportion (Female %)
Japan	Neoplasms	-3407.69	-58.2	-2252.5	-63.69	-1220.87	-52.67
Jordan	Neoplasms	-65.29	-179.71	-34.99	-187.16	-31.62	-179.31
Kazakhstan	Neoplasms	-425.48	-57.64	-264.24	-61.92	-156.03	-50.1
Kenya	Neoplasms	-12.17	-6.92	6.25	9.16	-17.17	-15.92
Kiribati	Neoplasms	-0.19	-12.25	-0.05	-7.58	-0.11	-12.54
Kuwait	Neoplasms	-26.42	-182.49	-14.45	-171.07	-11.16	-185.18
Kyrgyzstan	Neoplasms	-67.48	-54.05	-43.54	-62.41	-23.29	-42.29
Lao People's Democratic Republic	Neoplasms	-43.95	-53.64	-21.87	-52.54	-20.81	-51.63
Latvia	Neoplasms	-57.69	-38.48	-47.51	-56.47	-19.83	-30.13
Lebanon	Neoplasms	-93.16	-130.32	-55.99	-140.7	-38	-119.91
Lesotho	Neoplasms	18.22	77.8	7.25	64.6	9.73	79.76
Liberia	Neoplasms	-9.41	-32.98	-5.64	-36.63	-3.08	-23.46
Libya	Neoplasms	-37.49	-60.85	-20.26	-54.93	-14.04	-56.76
Lithuania	Neoplasms	-68.82	-37.25	-62.43	-58.48	-19.75	-25.31
Luxembourg	Neoplasms	-16.87	-68.79	-12.2	-87.16	-5.97	-56.7
Madagascar	Neoplasms	-57.47	-32.49	-21.23	-31.06	-35.08	-32.31
Malawi	Neoplasms	-26.98	-20.66	-4.01	-8.12	-24.04	-29.6
Malaysia	Neoplasms	-212.66	-69.46	-113.68	-74.56	-103.25	-67.18
Maldives	Neoplasms	-3.07	-138.58	-1.93	-161.37	-1.34	-131.12

Location	Cause	Attributable number (Both thousands)	Attributable proportion (Both %)	Attributable number (Male thousands)	Attributable proportion (Male %)	Attributable number (Female thousands)	Attributable proportion (Female %)
Mali	Neoplasms	-42.98	-30.97	-14.26	-22.11	-29.15	-39.22
Malta	Neoplasms	-9.69	-63.62	-6.59	-80.11	-3.67	-52.33
Marshall Islands	Neoplasms	-0.06	-10.4	-0.06	-18.99	0.01	3.32
Mauritania	Neoplasms	-22.83	-69.8	-14.24	-81.19	-7.86	-51.79
Mauritius	Neoplasms	-11.67	-58.8	-7.35	-73.51	-4.76	-48.35
Mexico	Neoplasms	-1031.08	-82.05	-458.39	-82.23	-579.11	-82.82
Micronesia (Federated States of)	Neoplasms	-0.41	-21.05	-0.2	-20.62	-0.18	-18.98
Monaco	Neoplasms	-0.74	-26.41	-0.65	-39.88	-0.14	-12.4
Mongolia	Neoplasms	-19.63	-28.44	-5.85	-15.42	-12.87	-41.41
Montenegro	Neoplasms	-4.41	-17.53	-4.02	-26.65	-1.12	-11.21
Morocco	Neoplasms	-77.7	-36.93	-52.08	-43.87	-22.31	-24.34
Mozambique	Neoplasms	-7.17	-3.9	10.41	14.3	-18.31	-16.46
Myanmar	Neoplasms	-467.79	-55.73	-174.34	-48.86	-274.98	-56.97
Namibia	Neoplasms	-0.83	-5.24	-0.48	-6.58	-0.26	-2.97
Nauru	Neoplasms	-0.05	-19.26	-0.02	-12.49	-0.02	-13.17
Nepal	Neoplasms	-122.79	-55.52	-43.76	-42.96	-73.43	-61.54
Netherlands	Neoplasms	-438.92	-52.17	-352.27	-73.61	-119.17	-32.85
New Zealand	Neoplasms	-88.26	-52.64	-57.44	-66.07	-34.43	-42.65
Nicaragua	Neoplasms	-23.84	-70.34	-9.78	-73.52	-14.25	-69.19
Niger	Neoplasms	-30.64	-44.19	-18.66	-53.95	-9.88	-28.45

Location	Cause	Attributable number (Both thousands)	Attributable proportion (Both %)	Attributable number (Male thousands)	Attributable proportion (Male %)	Attributable number (Female thousands)	Attributable proportion (Female %)
Nigeria	Neoplasms	-286.37	-34.92	-125.93	-32.41	-137.78	-31.93
Niue	Neoplasms	-0.01	-13.47	-0.01	-21.75	0	-9.93
North Macedonia	Neoplasms	-31.95	-39.46	-23.1	-49.05	-11.51	-33.96
Northern Mariana Islands	Neoplasms	-0.36	-41.66	-0.19	-39.72	-0.11	-30.47
Norway	Neoplasms	-109.93	-48.03	-76.96	-62.21	-37.94	-36.07
Oman	Neoplasms	-12.57	-108.13	-8.96	-118	-4.14	-102.68
Pakistan	Neoplasms	-188.88	-11.16	-74.97	-8.32	-27.5	-3.47
Palau	Neoplasms	-0.13	-25.16	-0.09	-38.76	-0.08	-26.56
Palestine	Neoplasms	-26.17	-94.48	-15.21	-103.2	-11.97	-92.39
Panama	Neoplasms	-35.08	-88.24	-21.02	-108.89	-14.2	-69.43
Papua New Guinea	Neoplasms	-10.17	-20.22	-6.26	-27.14	-4.13	-15.16
Paraguay	Neoplasms	-17.04	-33.08	-8.75	-37.68	-10.25	-36.23
Peru	Neoplasms	-391.51	-97.41	-232.81	-123.76	-171.31	-80.13
Philippines	Neoplasms	-250.36	-29.04	-135.33	-30.11	-72.13	-17.48
Poland	Neoplasms	-1000.4	-46.43	-760.65	-59.97	-312.94	-35.32
Portugal	Neoplasms	-347.66	-66.27	-263.56	-88.33	-128.55	-56.83
Puerto Rico	Neoplasms	-78.58	-72.66	-50.75	-81.06	-29.7	-65.22
Qatar	Neoplasms	-15.74	-337.34	-10.86	-364.33	-5.42	-321.9
Republic of Korea	Neoplasms	-2327.73	-136.41	-1673.25	-156.68	-764.5	-119.75
Republic of Moldova	Neoplasms	-77.9	-41.67	-55.77	-53.22	-33.02	-40.2

Location	Cause	Attributable number (Both thousands)	Attributable proportion (Both %)	Attributable number (Male thousands)	Attributable proportion (Male %)	Attributable number (Female thousands)	Attributable proportion (Female %)
Romania	Neoplasms	-184.15	-18.53	-188.91	-33.05	-57.72	-13.68
Russian Federation	Neoplasms	-4101.89	-50.21	-3206.92	-67.91	-1255.68	-36.42
Rwanda	Neoplasms	-93.23	-59.46	-33.44	-57.91	-60.51	-61.08
Saint Kitts and Nevis	Neoplasms	-1.18	-76.84	-0.67	-99.14	-0.58	-67.07
Saint Lucia	Neoplasms	-2.63	-72.26	-1.7	-94.53	-1.08	-58.77
Saint Vincent and the Grenadines	Neoplasms	-1.14	-41.16	-0.7	-55.68	-0.5	-33.32
Samoa	Neoplasms	-0.42	-18.53	-0.31	-27.49	-0.08	-7.25
San Marino	Neoplasms	-0.72	-51.99	-0.49	-55.58	-0.23	-44.7
Sao Tome and Principe	Neoplasms	-0.38	-27.76	-0.18	-28.13	-0.22	-29.89
Saudi Arabia	Neoplasms	-122.45	-112.93	-77.07	-118.63	-42.33	-97.4
Senegal	Neoplasms	-27	-34.64	-17.66	-45.05	-8.51	-21.96
Serbia	Neoplasms	-202.78	-38.71	-143.33	-47.5	-70.88	-31.91
Seychelles	Neoplasms	-1.5	-65.15	-1.15	-86.15	-0.45	-47.18
Sierra Leone	Neoplasms	-11.56	-25.57	-10.99	-43.24	0.68	3.42
Singapore	Neoplasms	-118.16	-136.52	-75.12	-155.79	-45.47	-118.63
Slovakia	Neoplasms	-164.88	-54.67	-127.04	-68.15	-48.44	-42.05
Slovenia	Neoplasms	-70.91	-67.24	-58.57	-94.99	-22.73	-51.9
Solomon Islands	Neoplasms	-0.72	-15.28	-0.54	-19.4	0.13	6.5
Somalia	Neoplasms	-25.39	-20.54	-10.34	-20.35	-14.21	-19.52
South Africa	Neoplasms	-97.78	-13.63	-86	-23.73	-12.78	-3.6

Location	Cause	Attributable number (Both thousands)	Attributable proportion (Both %)	Attributable number (Male thousands)	Attributable proportion (Male %)	Attributable number (Female thousands)	Attributable proportion (Female %)
South Sudan	Neoplasms	-15.25	-17.18	-6.43	-13.46	-6.24	-15.21
Spain	Neoplasms	-1147.8	-56.43	-901.63	-71.31	-329.73	-42.84
Sri Lanka	Neoplasms	-191.58	-76.54	-93.39	-70.92	-90.76	-76.51
Sudan	Neoplasms	-94.59	-43.39	-54.53	-47.19	-40.95	-39.96
Suriname	Neoplasms	-2.56	-32.21	-1.36	-36.28	-1.31	-31.43
Sweden	Neoplasms	-191.82	-41.22	-123.33	-50.86	-74.59	-33.48
Switzerland	Neoplasms	-155.32	-46.77	-109.12	-57.44	-53.01	-37.3
Syrian Arab Republic	Neoplasms	-93.42	-79.41	-51.88	-81.78	-39.39	-72.68
Taiwan (Province of China)	Neoplasms	-589.9	-97.17	-389.06	-101.57	-205.19	-91.59
Tajikistan	Neoplasms	-50.37	-52.1	-29.34	-55.35	-20.57	-47.09
Thailand	Neoplasms	-1300.64	-90.76	-792.72	-101.28	-564.29	-86.76
Timor-Leste	Neoplasms	-2.01	-27.86	-0.8	-24.49	-1.1	-27.98
Togo	Neoplasms	-5.92	-19.43	-1.94	-15.84	-4.21	-23.08
Tokelau	Neoplasms	-0.01	-25.02	-0.01	-32.02	0	-21.42
Tonga	Neoplasms	-0.41	-17.52	-0.22	-18.43	-0.22	-19.51
Trinidad and Tobago	Neoplasms	-16.49	-57.06	-9.52	-68.08	-7.46	-49.98
Tunisia	Neoplasms	-80.11	-75.19	-49.93	-76.76	-29.17	-70.3
Turkey	Neoplasms	-1965.08	-120.05	-1347.73	-122.02	-578.71	-108.72
Turkmenistan	Neoplasms	-32.05	-42.12	-19.4	-47.88	-12.83	-36.05
Tuvalu	Neoplasms	-0.06	-25.17	-0.04	-34.39	-0.04	-25.95

Location	Cause	Attributable number (Both thousands)	Attributable proportion (Both %)	Attributable number (Male thousands)	Attributable proportion (Male %)	Attributable number (Female thousands)	Attributable proportion (Female %)
Uganda	Neoplasms	-94.12	-37	-51.33	-38.96	-36.44	-29.72
Ukraine	Neoplasms	-952.04	-30.09	-644.06	-36.05	-375.92	-27.29
United Arab Emirates	Neoplasms	-55.1	-233.64	-37.91	-269.87	-19.19	-201.34
United Kingdom	Neoplasms	-1752.43	-46.11	-1298.29	-65.22	-598.34	-33.06
United Republic of Tanzania	Neoplasms	-158.94	-39.33	-70.43	-40.11	-85.86	-37.57
United States of America	Neoplasms	-10823.82	-82.34	-6492.65	-92.7	-4522.42	-73.63
United States Virgin Islands	Neoplasms	-1.16	-35.98	-0.54	-34	-0.68	-41.84
Uruguay	Neoplasms	-70.97	-37.62	-53.13	-47.89	-20.79	-26.75
Uzbekistan	Neoplasms	-192.21	-53.65	-120.51	-63.3	-64.88	-38.64
Vanuatu	Neoplasms	-0.2	-10.68	-0.14	-13.2	0.05	5.74
Venezuela (Bolivarian Republic of)	Neoplasms	-292.71	-86.21	-152.86	-97.86	-135.5	-73.92
Viet Nam	Neoplasms	-767.47	-66.39	-568.39	-83.19	-288.41	-61.01
Yemen	Neoplasms	-56.5	-46.22	-38.1	-53.81	-15.26	-29.65
Zambia	Neoplasms	-69.69	-53.11	-30.42	-56.27	-44.35	-57.47
Zimbabwe	Neoplasms	65.28	41.06	21.53	28.11	50.8	61.66

Table S5. Absolute (the number) and relative contribution (the proportion) associated with disease severity changes for thyroid cancer (ThyC) by sex at global, SDI regional, GBD regional level between 1990 and 2021.

Location	Cause	Attributable number (Both thousands)	Attributable proportion (Both %)	Attributable number (Male thousands)	Attributable proportion (Male %)	Attributable number (Female thousands)	Attributable proportion (Female %)
Global	Thyroid cancer	-465.77	-75.28	-197.61	-92.34	-296.71	-73.32
High SDI	Thyroid cancer	-125.72	-77.53	-55.02	-89.65	-76.94	-76.35
High-middle SDI	Thyroid cancer	-142.3	-92.15	-65.53	-120.81	-90.99	-90.82
Middle SDI	Thyroid cancer	-293.35	-186.79	-131.28	-244.08	-182.46	-176.7
Low-middle SDI	Thyroid cancer	-132.34	-137.73	-45.22	-152.45	-90.51	-136.27
Low SDI	Thyroid cancer	-59.28	-123.31	-18.47	-126.53	-41.78	-124.83
High-income Asia Pacific	Thyroid cancer	-25.1	-82.71	-11.54	-113.48	-16.1	-79.82
High-income North America	Thyroid cancer	-17.99	-48.77	-9.46	-58.6	-9.89	-47.67
Western Europe	Thyroid cancer	-64.99	-69.29	-29.11	-82.5	-40.44	-69.12
Australasia	Thyroid cancer	-3.2	-127.15	-1.58	-144.35	-1.85	-130.35
Eastern Europe	Thyroid cancer	-20.56	-55.31	-8.62	-69.13	-14.82	-59.99
Central Europe	Thyroid cancer	-22.75	-66.49	-9.08	-67.4	-13.91	-67.07
Southern Latin America	Thyroid cancer	-8.77	-94.21	-3.67	-104.26	-5.48	-94.63
East Asia	Thyroid cancer	-246	-223.04	-130.53	-344.11	-145.25	-200.74
Central Asia	Thyroid cancer	-4.69	-59.71	-1.65	-56.6	-2.97	-60.04
North Africa and Middle East	Thyroid cancer	-38.81	-189.43	-16.52	-246.82	-24.89	-180.39
Andean Latin America	Thyroid cancer	-13.72	-273.09	-3.97	-288.5	-10.04	-275.39
Southeast Asia	Thyroid cancer	-91.17	-151.23	-24.49	-149.71	-68.44	-155.81

Location	Cause	Attributable number (Both thousands)	Attributable proportion (Both %)	Attributable number (Male thousands)	Attributable proportion (Male %)	Attributable number (Female thousands)	Attributable proportion (Female %)
Tropical Latin America	Thyroid cancer	-17.34	-121.75	-7.27	-132.51	-10.92	-124.87
Southern Sub-Saharan Africa	Thyroid cancer	-1.94	-51.89	-0.75	-64.7	-1.3	-50.57
Caribbean	Thyroid cancer	-3.44	-87.42	-1.43	-109.6	-2.22	-84.63
Central Latin America	Thyroid cancer	-29.75	-171.88	-9.8	-177.98	-20.49	-173.58
South Asia	Thyroid cancer	-174.95	-180.16	-62.6	-191.28	-114.12	-177.27
Central Sub-Saharan Africa	Thyroid cancer	-2.39	-100.48	-0.77	-103.85	-1.69	-103.2
Oceania	Thyroid cancer	-0.23	-52.51	-0.05	-34.43	-0.19	-60.09
Western Sub-Saharan Africa	Thyroid cancer	-2.7	-84.38	-0.6	-74.29	-2.07	-86.47
Eastern Sub-Saharan Africa	Thyroid cancer	-32.42	-115.05	-11.33	-130.26	-22.62	-116.12
Afghanistan	Thyroid cancer	-1.14	-117.84	-0.18	-113.59	-0.92	-113.9
Albania	Thyroid cancer	-0.41	-115.28	-0.22	-117.53	-0.19	-114.88
Algeria	Thyroid cancer	-2.34	-172.88	-0.83	-216.28	-1.63	-168.68
American Samoa	Thyroid cancer	0	-56.1	0	-45.43	0	-59
Andorra	Thyroid cancer	-0.01	-81.69	0	-80.38	0	-84.04
Angola	Thyroid cancer	-0.66	-164.65	-0.21	-156.58	-0.45	-169.29
Antigua and Barbuda	Thyroid cancer	-0.01	-100.22	0	-103.18	-0.01	-100.32
Argentina	Thyroid cancer	-4.64	-72.63	-2.07	-83.79	-2.85	-72.48
Armenia	Thyroid cancer	-0.37	-94.65	-0.16	-129.39	-0.26	-97.43
Australia	Thyroid cancer	-2.8	-139.04	-1.31	-144.61	-1.61	-145.93
Austria	Thyroid cancer	-1.53	-59.28	-0.77	-80.53	-0.97	-59.87

Location	Cause	Attributable number (Both thousands)	Attributable proportion (Both %)	Attributable number (Male thousands)	Attributable proportion (Male %)	Attributable number (Female thousands)	Attributable proportion (Female %)
Azerbaijan	Thyroid cancer	-0.45	-76.24	-0.2	-94.48	-0.29	-78
Bahamas	Thyroid cancer	-0.03	-85.81	-0.01	-90.55	-0.02	-88.24
Bahrain	Thyroid cancer	-0.1	-219.81	-0.03	-379.1	-0.08	-209.4
Bangladesh	Thyroid cancer	-18.05	-208.12	-6.94	-213.87	-11.08	-204.11
Barbados	Thyroid cancer	-0.03	-82.16	-0.01	-79.49	-0.02	-84.06
Belarus	Thyroid cancer	-1.18	-73.65	-0.48	-102.27	-0.86	-75.68
Belgium	Thyroid cancer	-1.65	-61.11	-0.83	-74.48	-0.97	-60.8
Belize	Thyroid cancer	-0.01	-127.62	0	-162.23	-0.01	-132.66
Benin	Thyroid cancer	-0.07	-77.71	-0.02	-75.08	-0.05	-79.3
Bermuda	Thyroid cancer	-0.02	-127.16	-0.01	-166.95	-0.01	-121.63
Bhutan	Thyroid cancer	-0.08	-164.99	-0.03	-191.62	-0.05	-161.68
Bolivia (Plurinational State of)	Thyroid cancer	-2.45	-180.59	-0.58	-209.88	-1.97	-182.3
Bosnia and Herzegovina	Thyroid cancer	-0.47	-72.47	-0.17	-71.94	-0.3	-75.32
Botswana	Thyroid cancer	-0.06	-79.06	-0.02	-99.56	-0.04	-83.43
Brazil	Thyroid cancer	-16.87	-122.01	-7.08	-132.31	-10.61	-125.22
Brunei Darussalam	Thyroid cancer	-0.03	-109.29	-0.01	-135.51	-0.03	-111.59
Bulgaria	Thyroid cancer	-1.24	-54.07	-0.39	-37.01	-0.77	-61.57
Burkina Faso	Thyroid cancer	-0.12	-52.92	-0.03	-46.45	-0.09	-53.39
Burundi	Thyroid cancer	-0.42	-57.85	-0.15	-78.68	-0.33	-60.97
Cabo Verde	Thyroid cancer	0	-221.62	0	-1012	0	-212.26

Location	Cause	Attributable number (Both thousands)	Attributable proportion (Both %)	Attributable number (Male thousands)	Attributable proportion (Male %)	Attributable number (Female thousands)	Attributable proportion (Female %)
Cambodia	Thyroid cancer	-2.8	-204.3	-0.54	-187.24	-2.27	-209.73
Cameroon	Thyroid cancer	-0.22	-76.61	-0.06	-74.85	-0.16	-78.44
Canada	Thyroid cancer	-2.45	-67.73	-1.14	-81.58	-1.51	-67.77
Central African Republic	Thyroid cancer	-0.05	-34.32	-0.01	-34.6	-0.04	-36.84
Chad	Thyroid cancer	-0.05	-47.34	-0.01	-41.39	-0.04	-48.7
Chile	Thyroid cancer	-3.31	-150	-1.29	-160.13	-2.12	-150.95
China	Thyroid cancer	-244.99	-234.06	-137.24	-380.89	-142.12	-207.06
Colombia	Thyroid cancer	-9.63	-190.29	-2.94	-180.46	-6.7	-194.94
Comoros	Thyroid cancer	-0.04	-87.54	-0.01	-92.07	-0.03	-88.99
Congo	Thyroid cancer	-0.18	-120.66	-0.06	-135.4	-0.13	-122.2
Cook Islands	Thyroid cancer	0	-92.16	0	-82.43	0	-127.75
Costa Rica	Thyroid cancer	-0.38	-105.16	-0.14	-132.45	-0.27	-106.14
Coted'Ivoire	Thyroid cancer	-0.48	-105	-0.06	-72.08	-0.39	-104.08
Croatia	Thyroid cancer	-0.74	-52.7	-0.3	-54.27	-0.46	-54.53
Cuba	Thyroid cancer	-1.26	-96.47	-0.59	-124.18	-0.81	-97.51
Cyprus	Thyroid cancer	-0.21	-127.28	-0.1	-165.79	-0.13	-119.51
Czechia	Thyroid cancer	-2.07	-71.36	-0.8	-74.24	-1.32	-72.03
Democratic People's Republic of Korea	Thyroid cancer	-1.92	-81.91	-0.52	-77.32	-1.46	-87.25
Democratic Republic of the Congo	Thyroid cancer	-1.36	-86.25	-0.43	-86.79	-0.97	-89.62

Location	Cause	Attributable number (Both thousands)	Attributable proportion (Both %)	Attributable number (Male thousands)	Attributable proportion (Male %)	Attributable number (Female thousands)	Attributable proportion (Female %)
Denmark	Thyroid cancer	-0.54	-68.54	-0.24	-86.87	-0.34	-65.33
Djibouti	Thyroid cancer	-0.04	-141.74	-0.02	-170.36	-0.03	-145.38
Dominica	Thyroid cancer	0	-34.83	0	-52.43	0	-39.91
Dominican Republic	Thyroid cancer	-0.55	-111.19	-0.22	-124.59	-0.36	-114.14
Ecuador	Thyroid cancer	-3.05	-313.71	-0.97	-344.73	-2.18	-315.83
Egypt	Thyroid cancer	-4.47	-152.39	-2.02	-185.28	-2.76	-149.45
El Salvador	Thyroid cancer	-1.11	-171.58	-0.29	-174.05	-0.83	-172.35
Equatorial Guinea	Thyroid cancer	-0.07	-310.15	-0.03	-435.81	-0.04	-286.63
Eritrea	Thyroid cancer	-0.33	-92.34	-0.1	-98.2	-0.23	-93.86
Estonia	Thyroid cancer	-0.24	-78.86	-0.1	-135.98	-0.19	-80.92
Eswatini	Thyroid cancer	-0.03	-51.21	-0.01	-53.21	-0.02	-55.42
Ethiopia	Thyroid cancer	-19.96	-127.79	-6.92	-148.83	-13.83	-126.09
Fiji	Thyroid cancer	-0.07	-50.77	-0.01	-30.32	-0.06	-55.01
Finland	Thyroid cancer	-0.91	-73.69	-0.4	-91.74	-0.61	-76.49
France	Thyroid cancer	-12.9	-91.48	-5.43	-92	-7.52	-91.74
Gabon	Thyroid cancer	-0.07	-95.51	-0.03	-124.99	-0.05	-96.97
Gambia	Thyroid cancer	-0.02	-91.52	0	-82.76	-0.01	-92.04
Georgia	Thyroid cancer	-0.38	-43.06	-0.11	-34.46	-0.23	-41.44
Germany	Thyroid cancer	-16.21	-67.08	-8.49	-101.11	-10.56	-66.96
Ghana	Thyroid cancer	-0.08	-107.61	-0.06	-100.18	-0.02	-118.07

Location	Cause	Attributable number (Both thousands)	Attributable proportion (Both %)	Attributable number (Male thousands)	Attributable proportion (Male %)	Attributable number (Female thousands)	Attributable proportion (Female %)
Greece	Thyroid cancer	-0.76	-41.54	-0.37	-50.75	-0.49	-44.27
Greenland	Thyroid cancer	-0.01	-82.09	0	-93.86	0	-82.93
Grenada	Thyroid cancer	-0.01	-78.4	0	-99.36	-0.01	-80.39
Guam	Thyroid cancer	0	-52.11	0	-42.41	0	-62.99
Guatemala	Thyroid cancer	-1.49	-193.62	-0.41	-180.87	-1.07	-196.58
Guinea	Thyroid cancer	-0.16	-58.11	-0.07	-58.23	-0.1	-63.12
Guinea-Bissau	Thyroid cancer	-0.02	-61.81	0	-50.28	-0.01	-62.11
Guyana	Thyroid cancer	-0.05	-81.42	-0.02	-81.5	-0.03	-84.32
Haiti	Thyroid cancer	-0.63	-77.49	-0.14	-74.32	-0.5	-79.31
Honduras	Thyroid cancer	-0.27	-164.81	-0.08	-147.89	-0.19	-167.14
Hungary	Thyroid cancer	-2.15	-58.77	-0.84	-57.88	-1.32	-59.39
Iceland	Thyroid cancer	-0.06	-60.81	-0.03	-72.68	-0.03	-60.51
India	Thyroid cancer	-135.95	-188.32	-49.39	-201.74	-88.38	-185.25
Indonesia	Thyroid cancer	-25.39	-120.09	-6.6	-129.73	-19.95	-124.23
Iran (Islamic Republic of)	Thyroid cancer	-2.98	-236.1	-1.41	-316.42	-1.86	-227.31
Iraq	Thyroid cancer	-2.26	-175.96	-1	-194.17	-1.32	-172.2
Ireland	Thyroid cancer	-0.62	-90.93	-0.28	-102.17	-0.37	-89.45
Israel	Thyroid cancer	-1.09	-103.69	-0.48	-122.58	-0.68	-102.17
Italy	Thyroid cancer	-10.48	-58.16	-4.39	-66.89	-6.8	-59.36
Jamaica	Thyroid cancer	-0.16	-74.58	-0.04	-76.34	-0.12	-76.37

Location	Cause	Attributable number (Both thousands)	Attributable proportion (Both %)	Attributable number (Male thousands)	Attributable proportion (Male %)	Attributable number (Female thousands)	Attributable proportion (Female %)
Japan	Thyroid cancer	-16.64	-67.62	-7.9	-96.44	-10.77	-65.57
Jordan	Thyroid cancer	-0.54	-213.56	-0.24	-345.9	-0.38	-205.22
Kazakhstan	Thyroid cancer	-2.44	-56.29	-0.85	-49.38	-1.47	-56.23
Kenya	Thyroid cancer	-0.53	-98.56	-0.19	-113.57	-0.38	-103.55
Kiribati	Thyroid cancer	0	-52.38	0	-44.16	0	-62.22
Kuwait	Thyroid cancer	-0.16	-123.05	-0.08	-143.85	-0.09	-122.33
Kyrgyzstan	Thyroid cancer	-0.44	-64.93	-0.11	-70.99	-0.34	-65.07
Lao People's Democratic Republic	Thyroid cancer	-0.93	-145.97	-0.21	-150.81	-0.74	-148.35
Latvia	Thyroid cancer	-0.27	-58.48	-0.12	-90.9	-0.2	-61.01
Lebanon	Thyroid cancer	-0.46	-146.68	-0.22	-212.67	-0.29	-137.1
Lesotho	Thyroid cancer	-0.02	-20.27	0	-15.41	-0.01	-22.97
Liberia	Thyroid cancer	-0.06	-100.75	-0.02	-86.35	-0.04	-102.73
Libya	Thyroid cancer	-0.37	-108.95	-0.08	-110.65	-0.29	-108.94
Lithuania	Thyroid cancer	-0.21	-37.7	-0.12	-72.79	-0.17	-44.39
Luxembourg	Thyroid cancer	-0.12	-83.2	-0.06	-96.78	-0.06	-81.36
Madagascar	Thyroid cancer	-0.87	-75.79	-0.25	-75.76	-0.63	-77.74
Malawi	Thyroid cancer	-0.82	-95.85	-0.16	-96.46	-0.68	-97.68
Malaysia	Thyroid cancer	-3.66	-156.17	-1.2	-166.28	-2.59	-159.34
Maldives	Thyroid cancer	-0.03	-225.91	-0.02	-293.28	-0.02	-208.16
Mali	Thyroid cancer	-0.56	-76.22	-0.12	-82.6	-0.46	-78.19

Location	Cause	Attributable number (Both thousands)	Attributable proportion (Both %)	Attributable number (Male thousands)	Attributable proportion (Male %)	Attributable number (Female thousands)	Attributable proportion (Female %)
Malta	Thyroid cancer	-0.07	-87.63	-0.02	-96.21	-0.05	-88.26
Marshall Islands	Thyroid cancer	0	-70.46	0	-58.85	0	-76.27
Mauritania	Thyroid cancer	-0.06	-111.2	-0.02	-112.02	-0.04	-111.85
Mauritius	Thyroid cancer	-0.08	-66.36	-0.02	-63.32	-0.06	-70.15
Mexico	Thyroid cancer	-14.32	-170.95	-4.96	-183.18	-9.7	-171.25
Micronesia (Federated States of)	Thyroid cancer	-0.01	-74.89	0	-70.05	0	-79.86
Monaco	Thyroid cancer	0	-54.81	0	-60.87	0	-56.08
Mongolia	Thyroid cancer	-0.28	-128.55	-0.11	-136.34	-0.19	-131.13
Montenegro	Thyroid cancer	-0.04	-38.44	-0.02	-46.32	-0.03	-41.77
Morocco	Thyroid cancer	-1.62	-125.87	-0.6	-149.48	-1.1	-123.43
Mozambique	Thyroid cancer	-0.88	-67.54	-0.33	-78.66	-0.64	-72.81
Myanmar	Thyroid cancer	-8.52	-135.68	-1.87	-145.47	-6.82	-136.65
Namibia	Thyroid cancer	-0.09	-124.29	-0.03	-137.55	-0.06	-126.63
Nauru	Thyroid cancer	0	-56.97	0	-36.08	0	-62.98
Nepal	Thyroid cancer	-2.9	-182.24	-0.95	-189.98	-1.98	-181.65
Netherlands	Thyroid cancer	-2.1	-91.11	-0.62	-81.45	-1.47	-95.29
New Zealand	Thyroid cancer	-0.25	-50.66	-0.22	-120.71	-0.19	-61.13
Nicaragua	Thyroid cancer	-0.37	-185.34	-0.12	-188.88	-0.26	-186.25
Niger	Thyroid cancer	-0.1	-72.48	-0.02	-56.33	-0.07	-72.46
Nigeria	Thyroid cancer	-0.39	-119.36	-0.04	-93.42	-0.34	-119.03

Location	Cause	Attributable number (Both thousands)	Attributable proportion (Both %)	Attributable number (Male thousands)	Attributable proportion (Male %)	Attributable number (Female thousands)	Attributable proportion (Female %)
Niue	Thyroid cancer	0	-52.31	0	-52.14	0	-53.89
North Macedonia	Thyroid cancer	-0.28	-83.03	-0.13	-87.5	-0.16	-84.2
Northern Mariana Islands	Thyroid cancer	0	-65.96	0	-48.87	0	-82.7
Norway	Thyroid cancer	-0.62	-75.82	-0.34	-112.08	-0.37	-71.13
Oman	Thyroid cancer	-0.12	-198.61	-0.07	-295.89	-0.07	-183.48
Pakistan	Thyroid cancer	-15.62	-107	-4.25	-94.96	-11.14	-110.12
Palau	Thyroid cancer	0	-64.06	0	-66.98	0	-72.46
Palestine	Thyroid cancer	-0.2	-121.38	-0.06	-166.37	-0.16	-121.17
Panama	Thyroid cancer	-0.36	-128.35	-0.13	-169.73	-0.25	-124.28
Papua New Guinea	Thyroid cancer	-0.11	-57.61	-0.02	-42.35	-0.09	-66.95
Paraguay	Thyroid cancer	-0.46	-111.42	-0.18	-136.28	-0.31	-114.13
Peru	Thyroid cancer	-7.64	-283.7	-2.35	-286.3	-5.38	-287.01
Philippines	Thyroid cancer	-8.06	-84.63	-1.81	-60.26	-6.06	-92.82
Poland	Thyroid cancer	-9.51	-67.43	-3.61	-65.57	-5.81	-67.57
Portugal	Thyroid cancer	-2.07	-86.53	-1	-112.1	-1.39	-92.28
Puerto Rico	Thyroid cancer	-0.45	-85.3	-0.23	-100.98	-0.26	-85.76
Qatar	Thyroid cancer	-0.12	-339.95	-0.09	-714.34	-0.06	-274.34
Republic of Korea	Thyroid cancer	-16.3	-307.63	-6.29	-344.03	-10.93	-314.91
Republic of Moldova	Thyroid cancer	-0.35	-61.25	-0.18	-82.18	-0.22	-62.57
Romania	Thyroid cancer	-3.69	-80	-1.45	-78.92	-2.31	-83.06

Location	Cause	Attributable number (Both thousands)	Attributable proportion (Both %)	Attributable number (Male thousands)	Attributable proportion (Male %)	Attributable number (Female thousands)	Attributable proportion (Female %)
Russian Federation	Thyroid cancer	-15.51	-61.14	-6.41	-75.69	-11.22	-66.38
Rwanda	Thyroid cancer	-1.21	-106.83	-0.36	-134.2	-0.92	-106.89
Saint Kitts and Nevis	Thyroid cancer	-0.01	-125.65	0	-163.06	0	-120.85
Saint Lucia	Thyroid cancer	-0.02	-112.15	-0.01	-141.23	-0.02	-113.25
Saint Vincent and the Grenadines	Thyroid cancer	-0.01	-63.39	0	-104.06	-0.01	-67.61
Samoa	Thyroid cancer	-0.01	-70.65	0	-49.78	-0.01	-75.38
San Marino	Thyroid cancer	0	-46.98	0	-49.6	0	-48.37
Sao Tome and Principe	Thyroid cancer	0	-127.58	0	-136.75	0	-116.21
Saudi Arabia	Thyroid cancer	-3.96	-363.26	-2.03	-519.5	-2.36	-337.7
Senegal	Thyroid cancer	-0.12	-74.57	-0.03	-65.85	-0.08	-75.29
Serbia	Thyroid cancer	-1.43	-88.48	-0.57	-96.96	-0.91	-88.54
Seychelles	Thyroid cancer	0	-81.36	0	-102.62	0	-83.08
Sierra Leone	Thyroid cancer	-0.05	-65.56	-0.01	-49.63	-0.04	-65.3
Singapore	Thyroid cancer	-0.72	-181.56	-0.28	-190.94	-0.46	-180.48
Slovakia	Thyroid cancer	-0.86	-72.59	-0.32	-77.32	-0.58	-74.92
Slovenia	Thyroid cancer	-0.33	-74.38	-0.13	-78.36	-0.22	-77.82
Solomon Islands	Thyroid cancer	-0.01	-91.81	0	-68.23	-0.01	-100.54
Somalia	Thyroid cancer	-0.34	-48.57	-0.1	-44.3	-0.23	-50.03
South Africa	Thyroid cancer	-1.59	-67.34	-0.62	-79.54	-1.11	-70.23
South Sudan	Thyroid cancer	-0.34	-66.11	-0.12	-53.49	-0.19	-66.35

**Location**	**Cause**	**Attributable number (Both thousands)**	**Attributable proportion (Both %)**	**Attributable number (Male thousands)**	**Attributable proportion (Male %)**	**Attributable number (Female thousands)**	**Attributable proportion (Female %)**
Spain	Thyroid cancer	-5.99	-79.38	-2.91	-93.02	-3.53	-79.9
Sri Lanka	Thyroid cancer	-3.46	-143.68	-1.08	-119.02	-2.3	-153.79
Sudan	Thyroid cancer	-1.19	-145.86	-0.43	-228.18	-0.89	-143.5
Suriname	Thyroid cancer	-0.03	-73.12	-0.01	-86.23	-0.02	-76.48
Sweden	Thyroid cancer	-0.93	-50.44	-0.36	-51.22	-0.6	-53.56
Switzerland	Thyroid cancer	-0.78	-55.84	-0.36	-71.29	-0.48	-54.43
Syrian Arab Republic	Thyroid cancer	-0.76	-205.4	-0.29	-252.79	-0.51	-197.52
Taiwan (Province of China)	Thyroid cancer	-4.05	-123.73	-1.33	-107.97	-2.72	-132.91
Tajikistan	Thyroid cancer	0	-33.64	0	-44.84	0	-36.68
Thailand	Thyroid cancer	-12.43	-165.11	-3.89	-164.59	-8.82	-170.73
Timor-Leste	Thyroid cancer	-0.08	-136.01	-0.02	-108.41	-0.07	-144.29
Togo	Thyroid cancer	-0.05	-77.21	-0.01	-69.11	-0.04	-77.76
Tokelau	Thyroid cancer	0	-79.02	0	-82.55	0	-80.48
Tonga	Thyroid cancer	0	-50.38	0	-48.66	0	-56.52
Trinidad and Tobago	Thyroid cancer	-0.15	-97.34	-0.05	-115.82	-0.1	-99.08
Tunisia	Thyroid cancer	-0.7	-134.36	-0.25	-155.47	-0.48	-131.04
Turkey	Thyroid cancer	-12.22	-182.21	-5.3	-230.19	-7.76	-176
Turkmenistan	Thyroid cancer	-0.26	-63.39	-0.11	-59.25	-0.14	-62.2
Tuvalu	Thyroid cancer	0	-91.05	0	-104.38	0	-93.56
Uganda	Thyroid cancer	-2.36	-140.86	-0.95	-126.14	-1.34	-145.81

Location	Cause	Attributable number (Both thousands)	Attributable proportion (Both %)	Attributable number (Male thousands)	Attributable proportion (Male %)	Attributable number (Female thousands)	Attributable proportion (Female %)
Ukraine	Thyroid cancer	-2.18	-26.34	-0.9	-30.59	-1.54	-28.75
United Arab Emirates	Thyroid cancer	-0.43	-238.8	-0.23	-339.96	-0.28	-246.55
United Kingdom	Thyroid cancer	-5.34	-54.98	-2.33	-62.67	-3.36	-55.98
United Republic of Tanzania	Thyroid cancer	-2.26	-84.17	-0.79	-87.47	-1.53	-86.39
United States of America	Thyroid cancer	-15.78	-47.45	-8.4	-56.95	-8.57	-46.29
United States Virgin Islands	Thyroid cancer	0	-46.9	0	-56.83	0	-52.06
Uruguay	Thyroid cancer	-0.51	-72.41	-0.22	-88.35	-0.33	-72.64
Uzbekistan	Thyroid cancer	-0.32	-92.51	-0.12	-109.49	-0.23	-96.31
Vanuatu	Thyroid cancer	0	-58.48	0	-37.1	0	-69.3
Venezuela (Bolivarian Republic of)	Thyroid cancer	-2.3	-158.73	-0.8	-170	-1.56	-160.1
Viet Nam	Thyroid cancer	-21.48	-244.95	-6.41	-257.66	-15.55	-247.48
Yemen	Thyroid cancer	-0.52	-148.43	-0.15	-160.77	-0.38	-149.44
Zambia	Thyroid cancer	-1.03	-125.04	-0.56	-228.74	-0.74	-127.19
Zimbabwe	Thyroid cancer	-0.09	-8.35	0	0.78	-0.04	-4.94

Table S6. Absolute (the number) and relative contribution (the proportion) associated with disease severity changes for testicular cancer (TesC), by sex at global, SDI regional, GBD regional level between 1990 and 2021.

Location	Cause	Attributable number (Male thousands)	Attributable proportion (Male %)
Global	Testicular cancer	-292.03	-80.81
High SDI	Testicular cancer	-69.07	-71.73
High-middle SDI	Testicular cancer	-164.95	-170.17
Middle SDI	Testicular cancer	-436.32	-495.02
Low-middle SDI	Testicular cancer	-222.63	-375.94
Low SDI	Testicular cancer	-66.5	-328.78
High-income Asia Pacific	Testicular cancer	-6.34	-69.87
High-income North America	Testicular cancer	-14.2	-52.81
Western Europe	Testicular cancer	-41.44	-76.67
Australasia	Testicular cancer	-2.24	-83.17
Eastern Europe	Testicular cancer	-28.35	-109.11
Central Europe	Testicular cancer	-43.45	-155.68
Southern Latin America	Testicular cancer	-32.88	-270.89
East Asia	Testicular cancer	-233.39	-517.77
Central Asia	Testicular cancer	-6.44	-131.08
North Africa and Middle East	Testicular cancer	-34.78	-303.77
Andean Latin America	Testicular cancer	-40.66	-1198.45
Southeast Asia	Testicular cancer	-61.53	-375.76
Tropical Latin America	Testicular cancer	-52.51	-491.42
Southern Sub-Saharan Africa	Testicular cancer	-3.93	-162.64
Caribbean	Testicular cancer	-2.65	-609.95

Location	Cause	Attributable number (Male thousands)	Attributable proportion (Male %)
Central Latin America	Testicular cancer	-165.01	-893.96
South Asia	Testicular cancer	-310.74	-406.34
Central Sub-Saharan Africa	Testicular cancer	-6.59	-370.12
Oceania	Testicular cancer	-0.12	-77.05
Western Sub-Saharan Africa	Testicular cancer	-7.84	-223.61
Eastern Sub-Saharan Africa	Testicular cancer	-29.8	-396.84
Afghanistan	Testicular cancer	-0.22	-488.12
Albania	Testicular cancer	-0.99	-240.73
Algeria	Testicular cancer	-0.7	-206.28
American Samoa	Testicular cancer	0	-105.02
Andorra	Testicular cancer	0	-76.65
Angola	Testicular cancer	-2.16	-566.29
Antigua and Barbuda	Testicular cancer	0	-621.82
Argentina	Testicular cancer	-14.61	-202.62
Armenia	Testicular cancer	-0.41	-224.42
Australia	Testicular cancer	-1.9	-84.91
Austria	Testicular cancer	-0.82	-69.98
Azerbaijan	Testicular cancer	-0.74	-268.87
Bahamas	Testicular cancer	0	-656.88
Bahrain	Testicular cancer	-0.04	-294.3
Bangladesh	Testicular cancer	-49.53	-813.96
Barbados	Testicular cancer	-0.01	-331.72
Belarus	Testicular cancer	-1.41	-199.94

Location	Cause	Attributable number (Male thousands)	Attributable proportion (Male %)
Belgium	Testicular cancer	-0.66	-73.49
Belize	Testicular cancer	-0.01	-1507.14
Benin	Testicular cancer	-0.27	-203.73
Bermuda	Testicular cancer	0	-911.85
Bhutan	Testicular cancer	-0.2	-669.05
Bolivia (Plurinational State of)	Testicular cancer	-4.8	-864.93
Bosnia and Herzegovina	Testicular cancer	-0.74	-145.26
Botswana	Testicular cancer	-0.17	-276.24
Brazil	Testicular cancer	-51.48	-493.29
Brunei Darussalam	Testicular cancer	-0.05	-187.32
Bulgaria	Testicular cancer	-4.5	-153.32
Burkina Faso	Testicular cancer	-0.29	-131.25
Burundi	Testicular cancer	-0.45	-198.84
Cabo Verde	Testicular cancer	-0.01	-370.94
Cambodia	Testicular cancer	-1.71	-682.32
Cameroon	Testicular cancer	-0.77	-222.49
Canada	Testicular cancer	-1.83	-79.72
Central African Republic	Testicular cancer	-0.05	-55.46
Chad	Testicular cancer	-0.15	-105.4
Chile	Testicular cancer	-17.8	-410.32
China	Testicular cancer	-258.38	-591.81
Colombia	Testicular cancer	-39.88	-1215.84
Comoros	Testicular cancer	-0.06	-276.83

Location	Cause	Attributable number (Male thousands)	Attributable proportion (Male %)
Congo	Testicular cancer	-0.46	-456.07
Cook Islands	Testicular cancer	0	-200.24
Costa Rica	Testicular cancer	-1.34	-548.78
Coted'Ivoire	Testicular cancer	-0.82	-289.06
Croatia	Testicular cancer	-0.75	-86.8
Cuba	Testicular cancer	-0.97	-577.88
Cyprus	Testicular cancer	-0.16	-247.85
Czechia	Testicular cancer	-3.44	-129.35
Democratic People's Republic of Korea	Testicular cancer	-1	-156.48
Democratic Republic of the Congo	Testicular cancer	-3.34	-291.36
Denmark	Testicular cancer	-0.69	-86.71
Djibouti	Testicular cancer	-0.12	-444.13
Dominica	Testicular cancer	0	-222.91
Dominican Republic	Testicular cancer	-0.15	-327.55
Ecuador	Testicular cancer	-7.31	-3580.83
Egypt	Testicular cancer	-2.23	-347.16
El Salvador	Testicular cancer	-3.31	-1215.3
Equatorial Guinea	Testicular cancer	-0.47	-3479.73
Eritrea	Testicular cancer	-0.34	-234.02
Estonia	Testicular cancer	-0.27	-130.06
Eswatini	Testicular cancer	-0.03	-123.31
Ethiopia	Testicular cancer	-15.16	-718.43
Fiji	Testicular cancer	-0.06	-78.95

Location	Cause	Attributable number (Male thousands)	Attributable proportion (Male %)
Finland	Testicular cancer	-0.35	-85.12
France	Testicular cancer	-7.86	-95.82
Gabon	Testicular cancer	-0.19	-410.39
Gambia	Testicular cancer	-0.09	-222.19
Georgia	Testicular cancer	-0.7	-54.9
Germany	Testicular cancer	-14.86	-85.32
Ghana	Testicular cancer	-1.15	-246.86
Greece	Testicular cancer	-0.58	-42.62
Greenland	Testicular cancer	-0.02	-81.83
Grenada	Testicular cancer	-0.01	-1138.99
Guam	Testicular cancer	0	-59.6
Guatemala	Testicular cancer	-3.59	-706.7
Guinea	Testicular cancer	-0.57	-179.08
Guinea-Bissau	Testicular cancer	-0.04	-134.99
Guyana	Testicular cancer	-0.05	-646.82
Haiti	Testicular cancer	-0.11	-129.07
Honduras	Testicular cancer	-0.35	-234.97
Hungary	Testicular cancer	-4.65	-143.71
Iceland	Testicular cancer	-0.01	-62.83
India	Testicular cancer	-233.27	-405.82
Indonesia	Testicular cancer	-21.37	-356.26
Iran (Islamic Republic of)	Testicular cancer	-2.45	-327.86
Iraq	Testicular cancer	-1.17	-307.88

Location	Cause	Attributable number (Male thousands)	Attributable proportion (Male %)
Ireland	Testicular cancer	-0.45	-118.7
Israel	Testicular cancer	-0.36	-151.05
Italy	Testicular cancer	-6.01	-97.41
Jamaica	Testicular cancer	-0.06	-460.53
Japan	Testicular cancer	-4.65	-60.22
Jordan	Testicular cancer	-0.59	-532.82
Kazakhstan	Testicular cancer	-3.31	-217.68
Kenya	Testicular cancer	-0.28	-166.83
Kiribati	Testicular cancer	0	-101.53
Kuwait	Testicular cancer	-0.04	-105.52
Kyrgyzstan	Testicular cancer	-0.77	-175.96
Lao People's Democratic Republic	Testicular cancer	-0.45	-434.16
Latvia	Testicular cancer	-0.53	-143.3
Lebanon	Testicular cancer	-0.29	-417.2
Lesotho	Testicular cancer	-0.02	-48.52
Liberia	Testicular cancer	-0.24	-303.43
Libya	Testicular cancer	-0.06	-134.23
Lithuania	Testicular cancer	-0.21	-77.56
Luxembourg	Testicular cancer	-0.06	-92.76
Madagascar	Testicular cancer	-1.08	-232.59
Malawi	Testicular cancer	-2.46	-341.06
Malaysia	Testicular cancer	-4.26	-413.86
Maldives	Testicular cancer	-0.08	-2644.44

Location	Cause	Attributable number (Male thousands)	Attributable proportion (Male %)
Mali	Testicular cancer	-0.99	-254.05
Malta	Testicular cancer	-0.05	-106.66
Marshall Islands	Testicular cancer	0	-109.88
Mauritania	Testicular cancer	-0.36	-405.34
Mauritius	Testicular cancer	-0.1	-222.68
Mexico	Testicular cancer	-110.21	-835.18
Micronesia (Federated States of)	Testicular cancer	0	-163.73
Monaco	Testicular cancer	-0.01	-57.43
Mongolia	Testicular cancer	-0.65	-573.89
Montenegro	Testicular cancer	-0.07	-70.18
Morocco	Testicular cancer	-0.23	-163.17
Mozambique	Testicular cancer	-1.19	-236.03
Myanmar	Testicular cancer	-4.1	-389.36
Namibia	Testicular cancer	-0.38	-398.37
Nauru	Testicular cancer	0	-92.17
Nepal	Testicular cancer	-4.28	-543.19
Netherlands	Testicular cancer	-1.68	-98.7
New Zealand	Testicular cancer	-0.37	-79.02
Nicaragua	Testicular cancer	-1.68	-1067.78
Niger	Testicular cancer	-0.25	-135.5
Nigeria	Testicular cancer	-0.8	-289.76
Niue	Testicular cancer	0	-96.57
North Macedonia	Testicular cancer	-1.08	-204.73

Location	Cause	Attributable number (Male thousands)	Attributable proportion (Male %)
Northern Mariana Islands	Testicular cancer	0	-68.84
Norway	Testicular cancer	-0.28	-65.09
Oman	Testicular cancer	-0.05	-371.39
Pakistan	Testicular cancer	-31.91	-263.92
Palau	Testicular cancer	0	-106.48
Palestine	Testicular cancer	-0.11	-240.72
Panama	Testicular cancer	-1.07	-1079.27
Papua New Guinea	Testicular cancer	-0.04	-101.04
Paraguay	Testicular cancer	-1.08	-430.02
Peru	Testicular cancer	-27.96	-1061.35
Philippines	Testicular cancer	-3.16	-119.02
Poland	Testicular cancer	-17.89	-203.16
Portugal	Testicular cancer	-1.55	-133.61
Puerto Rico	Testicular cancer	-0.65	-940.35
Qatar	Testicular cancer	-0.04	-1518.48
Republic of Korea	Testicular cancer	-3.91	-331.29
Republic of Moldova	Testicular cancer	-0.76	-144.04
Romania	Testicular cancer	-8.68	-210.66
Russian Federation	Testicular cancer	-18.97	-110.88
Rwanda	Testicular cancer	-1.63	-481.4
Saint Kitts and Nevis	Testicular cancer	-0.01	-2122.36
Saint Lucia	Testicular cancer	-0.03	-1421.86
Saint Vincent and the Grenadines	Testicular cancer	-0.01	-609.74

Location	Cause	Attributable number (Male thousands)	Attributable proportion (Male %)
Samoa	Testicular cancer	-0.03	-141.78
San Marino	Testicular cancer	0	-43.92
Sao Tome and Principe	Testicular cancer	-0.01	-391.88
Saudi Arabia	Testicular cancer	-2.43	-898.45
Senegal	Testicular cancer	-0.58	-215.94
Serbia	Testicular cancer	-2.96	-163.74
Seychelles	Testicular cancer	-0.01	-189.57
Sierra Leone	Testicular cancer	-0.2	-172.51
Singapore	Testicular cancer	-0.32	-215.51
Slovakia	Testicular cancer	-1.53	-130.96
Slovenia	Testicular cancer	-0.38	-119.7
Solomon Islands	Testicular cancer	-0.01	-164.57
Somalia	Testicular cancer	-0.23	-79.25
South Africa	Testicular cancer	-3.42	-173.53
South Sudan	Testicular cancer	-0.26	-112.96
Spain	Testicular cancer	-2.95	-87.35
Sri Lanka	Testicular cancer	-1.87	-336.57
Sudan	Testicular cancer	-0.85	-596.34
Suriname	Testicular cancer	-0.03	-391.73
Sweden	Testicular cancer	-0.36	-52.56
Switzerland	Testicular cancer	-0.51	-52.34
Syrian Arab Republic	Testicular cancer	-0.22	-172.56
Taiwan (Province of China)	Testicular cancer	-1.1	-141.7

Location	Cause	Attributable number (Male thousands)	Attributable proportion (Male %)
Tajikistan	Testicular cancer	-0.02	-114.94
Thailand	Testicular cancer	-10.1	-438.4
Timor-Leste	Testicular cancer	-0.05	-269.32
Togo	Testicular cancer	-0.2	-176.57
Tokelau	Testicular cancer	0	-217.31
Tonga	Testicular cancer	0	-106.13
Trinidad and Tobago	Testicular cancer	-0.11	-875.41
Tunisia	Testicular cancer	-0.22	-172.49
Turkey	Testicular cancer	-22.39	-281.73
Turkmenistan	Testicular cancer	-1	-391.07
Tuvalu	Testicular cancer	0	-292.02
Uganda	Testicular cancer	-2.13	-315.29
Ukraine	Testicular cancer	-4.61	-67.81
United Arab Emirates	Testicular cancer	-0.49	-411.25
United Kingdom	Testicular cancer	-3.72	-44.12
United Republic of Tanzania	Testicular cancer	-3.27	-265.95
United States of America	Testicular cancer	-12.08	-49.16
United States Virgin Islands	Testicular cancer	0	-262.77
Uruguay	Testicular cancer	-1.01	-171.18
Uzbekistan	Testicular cancer	-1.89	-226.36
Vanuatu	Testicular cancer	0	-71.15
Venezuela (Bolivarian Republic of)	Testicular cancer	-5.31	-965.5
Viet Nam	Testicular cancer	-11.83	-507.38

**Location**	**Cause**	**Attributable number (Male thousands)**	**Attributable proportion (Male %)**
Yemen	Testicular cancer	-0.26	-378.41
Zambia	Testicular cancer	-4.63	-1337.98
Zimbabwe	Testicular cancer	0.02	8.76

Table S7. Absolute (the number) and relative contribution (the proportion) associated with disease severity changes for mesothelioma (Meso), by sex at global, SDI regional, GBD regional level between 1990 and 2021.

Location	Cause	Attributable number (Both thousands)	Attributable proportion (Both %)	Attributable number (Male thousands)	Attributable proportion (Male %)	Attributable number (Female thousands)	Attributable proportion (Female %)
Global	Mesothelioma	1.86	0.48	7.31	2.6	-4.79	-4.55
High SDI	Mesothelioma	-8.71	-4.58	-4.06	-2.7	-3.53	-8.85
High-middle SDI	Mesothelioma	-1.19	-1.25	-0.95	-1.53	-0.03	-0.1
Middle SDI	Mesothelioma	-3.66	-6.32	-0.44	-1.14	-3.2	-16.81
Low-middle SDI	Mesothelioma	-1.47	-4.84	0.04	0.19	-1.48	-16.31
Low SDI	Mesothelioma	-0.73	-5.49	-0.03	-0.36	-0.71	-15.6
High-income Asia Pacific	Mesothelioma	-1.71	-11.38	-0.84	-7.83	-0.64	-15.13
High-income North America	Mesothelioma	-2.16	-4.05	-0.79	-1.85	-1.15	-10.58
Western Europe	Mesothelioma	-12.87	-8.93	-8.32	-7.43	-3.13	-9.75
Australasia	Mesothelioma	1.28	13.1	1.45	17.02	0.02	1.82
Eastern Europe	Mesothelioma	0.11	0.59	0.15	1.4	0.09	0.96
Central Europe	Mesothelioma	-0.17	-2.31	0.36	8.09	-0.35	-11.65
Southern Latin America	Mesothelioma	-0.3	-8.47	-0.06	-2.35	-0.23	-23.35
East Asia	Mesothelioma	-0.73	-1.87	-0.15	-0.6	-0.78	-5.42
Central Asia	Mesothelioma	0.05	2.08	0.05	4.47	0.05	4.67
North Africa and Middle East	Mesothelioma	-1.41	-8.17	-0.53	-4.13	-0.89	-19.96
Andean Latin America	Mesothelioma	-0.12	-7.27	-0.02	-2.07	-0.1	-17.09
Southeast Asia	Mesothelioma	-0.34	-2.59	0.24	2.59	-0.54	-15.2

Location	Cause	Attributable number (Both thousands)	Attributable proportion (Both %)	Attributable number (Male thousands)	Attributable proportion (Male %)	Attributable number (Female thousands)	Attributable proportion (Female %)
Tropical Latin America	Mesothelioma	-0.76	-6.82	0.06	0.84	-0.73	-16.34
Southern Sub-Saharan Africa	Mesothelioma	0.12	3.12	0.17	5.98	-0.06	-6.91
Caribbean	Mesothelioma	-0.03	-2.25	0.03	2.88	-0.06	-10.6
Central Latin America	Mesothelioma	-0.38	-7.02	0	0	-0.34	-16.96
South Asia	Mesothelioma	-1.67	-6.6	-0.3	-1.59	-1.34	-20.86
Central Sub-Saharan Africa	Mesothelioma	-0.04	-3.02	0.01	0.77	-0.05	-15.65
Oceania	Mesothelioma	0	2.01	0	3.91	0	-11.45
Western Sub-Saharan Africa	Mesothelioma	-0.33	-4.47	0.09	2.13	-0.47	-14.36
Eastern Sub-Saharan Africa	Mesothelioma	-0.38	-7.17	-0.06	-1.82	-0.33	-16.7
Afghanistan	Mesothelioma	0	-1.61	0	0.91	0	-15
Albania	Mesothelioma	0	-1.83	0	-2.78	0	-0.18
Algeria	Mesothelioma	-0.01	-6.69	0	-2.73	-0.01	-24.47
American Samoa	Mesothelioma	0	0.04	0	2.36	0	-13.38
Andorra	Mesothelioma	0	-14.75	0	-13.73	0	-21.4
Angola	Mesothelioma	-0.02	-6.22	0	-1.71	-0.01	-22.9
Antigua and Barbuda	Mesothelioma	0	-3.46	0	1.13	0	-9.44
Argentina	Mesothelioma	-0.19	-7.79	-0.02	-1.39	-0.16	-22.13
Armenia	Mesothelioma	-0.01	-4.39	0	1.4	0	0.63
Australia	Mesothelioma	1.31	15.33	1.38	18.43	0.05	4.98
Austria	Mesothelioma	-0.27	-17.61	-0.17	-16.04	-0.08	-17.14

Location	Cause	Attributable number (Both thousands)	Attributable proportion (Both %)	Attributable number (Male thousands)	Attributable proportion (Male %)	Attributable number (Female thousands)	Attributable proportion (Female %)
Azerbaijan	Mesothelioma	0.01	7.15	0	3.04	0	5.4
Bahamas	Mesothelioma	0	-0.71	0	3.44	0	-13.08
Bahrain	Mesothelioma	0	-14.81	0	-11.88	0	-89.39
Bangladesh	Mesothelioma	-0.23	-10.06	-0.1	-5.49	-0.13	-25.54
Barbados	Mesothelioma	0	-3.08	0	1.35	0	-8.04
Belarus	Mesothelioma	0	-0.39	0	0.86	0	0.06
Belgium	Mesothelioma	-0.56	-16.33	-0.42	-15.32	-0.12	-17.43
Belize	Mesothelioma	0	1.18	0	6.71	0	-16.3
Benin	Mesothelioma	-0.01	-4.6	0	2.05	-0.01	-13.95
Bermuda	Mesothelioma	0	-6.31	0	-4.27	0	-11.02
Bhutan	Mesothelioma	0	-8.49	0	-2.78	0	-22.97
Bolivia (Plurinational State of)	Mesothelioma	-0.02	-7.08	0	-0.85	-0.02	-17.95
Bosnia and Herzegovina	Mesothelioma	0	-1.2	0	-1.1	0	-0.29
Botswana	Mesothelioma	0	0.5	0	3.71	0	-10.04
Brazil	Mesothelioma	-0.75	-6.85	0.05	0.79	-0.72	-16.36
Brunei Darussalam	Mesothelioma	0	-9.06	0	-3.15	0	-21.63
Bulgaria	Mesothelioma	0	1.46	0	3.11	0	1.95
Burkina Faso	Mesothelioma	-0.01	-3.8	0	2.02	-0.02	-10.97
Burundi	Mesothelioma	-0.01	-6.84	0	-2.87	-0.01	-13.1
Cabo Verde	Mesothelioma	0	-4.54	0	4.09	0	-13.91

Location	Cause	Attributable number (Both thousands)	Attributable proportion (Both %)	Attributable number (Male thousands)	Attributable proportion (Male %)	Attributable number (Female thousands)	Attributable proportion (Female %)
Cambodia	Mesothelioma	-0.01	-5.38	0	1.23	-0.01	-20.1
Cameroon	Mesothelioma	-0.02	-3.24	0.01	4.69	-0.03	-13.6
Canada	Mesothelioma	-0.47	-6.66	-0.25	-4.21	-0.17	-15.29
Central African Republic	Mesothelioma	0	0.61	0	3.43	0	-9.47
Chad	Mesothelioma	0	-2.23	0	4.62	-0.01	-10.25
Chile	Mesothelioma	-0.1	-11.52	-0.04	-5.39	-0.06	-29.61
China	Mesothelioma	-0.95	-2.54	-0.31	-1.35	-0.83	-5.91
Colombia	Mesothelioma	-0.13	-10.83	-0.03	-3.99	-0.09	-19.86
Comoros	Mesothelioma	0	-5.46	0	-0.2	0	-14.61
Congo	Mesothelioma	0	-5.12	0	-1.01	0	-16.96
Cook Islands	Mesothelioma	0	-4.49	0	-3.16	0	-15.59
Costa Rica	Mesothelioma	0	-5.44	0	2.23	0	-17.45
Coted'Ivoire	Mesothelioma	-0.02	-3.95	0.01	2.72	-0.03	-14.72
Croatia	Mesothelioma	-0.08	-11.04	-0.03	-4.72	0	-2.09
Cuba	Mesothelioma	-0.02	-4.8	0	1.57	-0.03	-11.24
Cyprus	Mesothelioma	-0.04	-20.91	-0.03	-19.69	-0.01	-24.61
Czechia	Mesothelioma	-0.16	-15.68	-0.04	-6.15	-0.02	-3.51
Democratic People's Republic of Korea	Mesothelioma	-0.01	-1.93	0.02	3.76	-0.03	-12.2
Democratic Republic of the Congo	Mesothelioma	-0.02	-2.04	0.01	1.7	-0.03	-14.28
Denmark	Mesothelioma	-0.27	-17.23	-0.21	-16.31	-0.05	-19.24

Location	Cause	Attributable number (Both thousands)	Attributable proportion (Both %)	Attributable number (Male thousands)	Attributable proportion (Male %)	Attributable number (Female thousands)	Attributable proportion (Female %)
Djibouti	Mesothelioma	0	-4.18	0	5.21	0	-20.36
Dominica	Mesothelioma	0	3.38	0	3.6	0	-7.76
Dominican Republic	Mesothelioma	0	-1.8	0	4.77	-0.01	-11.32
Ecuador	Mesothelioma	-0.02	-7.69	0	-0.46	-0.02	-18.77
Egypt	Mesothelioma	-0.08	-3.13	-0.01	-0.68	-0.06	-16.97
El Salvador	Mesothelioma	0	-7.61	0	-0.64	0	-17.52
Equatorial Guinea	Mesothelioma	0	-11.38	0	-6.49	0	-29.52
Eritrea	Mesothelioma	-0.01	-10.6	0	-4.56	-0.01	-20.1
Estonia	Mesothelioma	0	-8.42	0	-6.98	0	-7.37
Eswatini	Mesothelioma	0	3.77	0	6.12	0	-6.73
Ethiopia	Mesothelioma	-0.2	-11.87	-0.08	-7.54	-0.12	-20.24
Fiji	Mesothelioma	0	1.3	0	3.85	0	-7.51
Finland	Mesothelioma	-0.31	-20.4	-0.21	-19.19	-0.08	-17.94
France	Mesothelioma	-3.67	-19.13	-2.8	-18.5	-0.79	-19.56
Gabon	Mesothelioma	0	-2.62	0	0.37	0	-12.71
Gambia	Mesothelioma	0	-2.78	0	4.5	0	-14.02
Georgia	Mesothelioma	-0.01	-12.84	0.01	20.8	0	4.07
Germany	Mesothelioma	-0.36	-1.6	1.05	6.4	-0.03	-0.5
Ghana	Mesothelioma	-0.05	-5.09	0.01	2.99	-0.07	-13.53
Greece	Mesothelioma	-0.12	-10.61	-0.08	-9.11	-0.03	-13.37

Location	Cause	Attributable number (Both thousands)	Attributable proportion (Both %)	Attributable number (Male thousands)	Attributable proportion (Male %)	Attributable number (Female thousands)	Attributable proportion (Female %)
Greenland	Mesothelioma	0	-26.36	0	-15.56	0	-20.48
Grenada	Mesothelioma	0	-3.96	0	1.57	0	-9.06
Guam	Mesothelioma	0	3.98	0	4.16	0	-14.89
Guatemala	Mesothelioma	-0.01	-7.75	0	-1.27	-0.01	-17.55
Guinea	Mesothelioma	-0.01	-2.29	0.01	4.21	-0.01	-10.72
Guinea-Bissau	Mesothelioma	0	-3.45	0	1.61	0	-10.83
Guyana	Mesothelioma	0	-4.2	0	2.35	0	-11
Haiti	Mesothelioma	-0.01	-3.09	0	2.38	-0.01	-13.56
Honduras	Mesothelioma	0	-3.43	0	5.16	-0.01	-17.67
Hungary	Mesothelioma	-0.05	-6.68	-0.02	-4.17	0	-1.44
Iceland	Mesothelioma	-0.01	-18.69	-0.01	-17.53	0	-20.87
India	Mesothelioma	-1.42	-6.95	-0.27	-1.8	-1.1	-21.29
Indonesia	Mesothelioma	-0.07	-1.84	0.13	4.61	-0.19	-15.25
Iran (Islamic Republic of)	Mesothelioma	-0.03	-7.02	-0.02	-4.2	-0.01	-22.22
Iraq	Mesothelioma	-0.04	-6.12	0	-0.23	-0.04	-23.33
Ireland	Mesothelioma	-0.14	-17.71	-0.12	-17.28	-0.02	-18.9
Israel	Mesothelioma	-0.1	-21.99	-0.07	-20.08	-0.03	-22.63
Italy	Mesothelioma	-1.97	-6.54	-1.26	-5.94	-0.83	-9.3
Jamaica	Mesothelioma	0	-2.07	0	3.53	0	-8.15
Japan	Mesothelioma	-1.39	-10.45	-0.66	-6.95	-0.52	-13.71

Location	Cause	Attributable number (Both thousands)	Attributable proportion (Both %)	Attributable number (Male thousands)	Attributable proportion (Male %)	Attributable number (Female thousands)	Attributable proportion (Female %)
Jordan	Mesothelioma	-0.01	-8.37	0	-3.35	0	-46.09
Kazakhstan	Mesothelioma	0.01	0.98	0.01	1.04	0.01	1.73
Kenya	Mesothelioma	-0.01	-3.03	0.02	6.48	-0.03	-15.7
Kiribati	Mesothelioma	0	0.66	0	2.31	0	-11.19
Kuwait	Mesothelioma	0	-3.85	0	1.46	0	-151.27
Kyrgyzstan	Mesothelioma	0	12.67	0	5.49	0	12.06
Lao People's Democratic Republic	Mesothelioma	0	-5.34	0	0.14	0	-18.86
Latvia	Mesothelioma	0	0.43	0	1.34	0	-0.56
Lebanon	Mesothelioma	-0.02	-8.63	-0.01	-6.04	-0.01	-23.77
Lesotho	Mesothelioma	0.01	11.19	0.01	14.62	0	-0.3
Liberia	Mesothelioma	-0.01	-5.64	0	0.94	-0.01	-16.04
Libya	Mesothelioma	0	-0.39	0	3.46	0	-20.93
Lithuania	Mesothelioma	0	2.11	0	2.83	0	1.57
Luxembourg	Mesothelioma	-0.02	-21.07	-0.02	-20.61	0	-19.91
Madagascar	Mesothelioma	-0.01	-3.09	0	2.56	-0.01	-12.52
Malawi	Mesothelioma	-0.01	-4.46	0	1.25	-0.01	-13.83
Malaysia	Mesothelioma	-0.01	-5.34	0	2.75	-0.01	-22.93
Maldives	Mesothelioma	0	-8.32	0	-4.19	0	-27.71
Mali	Mesothelioma	-0.02	-4.7	0	2.08	-0.02	-12.74
Malta	Mesothelioma	-0.02	-24.33	-0.01	-24.14	0	-20.77

Location	Cause	Attributable number (Both thousands)	Attributable proportion (Both %)	Attributable number (Male thousands)	Attributable proportion (Male %)	Attributable number (Female thousands)	Attributable proportion (Female %)
Marshall Islands	Mesothelioma	0	0.28	0	1.32	0	-10.34
Mauritania	Mesothelioma	-0.01	-8.4	0	-1.35	-0.01	-16.81
Mauritius	Mesothelioma	0	-6.95	0	4.45	0	-30.99
Mexico	Mesothelioma	-0.21	-6.14	0.02	0.9	-0.2	-16.67
Micronesia (Federated States of)	Mesothelioma	0	-0.36	0	0.74	0	-10.7
Monaco	Mesothelioma	0	-10.72	0	-10.51	0	-12.82
Mongolia	Mesothelioma	0	2.94	0	2.34	0	2.02
Montenegro	Mesothelioma	0	2.8	0	4.34	0	3.28
Morocco	Mesothelioma	-0.01	-3.57	0	0.07	-0.01	-19.39
Mozambique	Mesothelioma	0	0.97	0.02	6.72	-0.01	-9.13
Myanmar	Mesothelioma	-0.05	-5.12	0	0.05	-0.05	-13.99
Namibia	Mesothelioma	0	-1.43	0	1.4	0	-12.19
Nauru	Mesothelioma	0	0.77	0	1.68	0	-8.31
Nepal	Mesothelioma	-0.03	-8.25	0	-1.95	-0.02	-21.57
Netherlands	Mesothelioma	-0.78	-10.51	-0.45	-6.97	-0.27	-27.35
New Zealand	Mesothelioma	-0.08	-6.65	-0.01	-0.81	-0.03	-18.09
Nicaragua	Mesothelioma	0	-6.22	0	2.03	0	-18.54
Niger	Mesothelioma	-0.01	-4.05	0	2.59	-0.01	-14.46
Nigeria	Mesothelioma	-0.16	-5.04	0.02	1.07	-0.2	-16
Niue	Mesothelioma	0	-0.14	0	0.98	0	-7.53

Location	Cause	Attributable number (Both thousands)	Attributable proportion (Both %)	Attributable number (Male thousands)	Attributable proportion (Male %)	Attributable number (Female thousands)	Attributable proportion (Female %)
North Macedonia	Mesothelioma	0	0.71	0	-0.13	0	0.54
Northern Mariana Islands	Mesothelioma	0	-0.82	0	2.96	0	-28.48
Norway	Mesothelioma	-0.26	-14.83	-0.2	-14.26	-0.05	-15.35
Oman	Mesothelioma	0	-10.09	0	-7.6	0	-34.95
Pakistan	Mesothelioma	-0.02	-0.68	0.06	3.66	-0.08	-13.51
Palau	Mesothelioma	0	0.59	0	1.84	0	-12.17
Palestine	Mesothelioma	0	-5.08	0	-0.83	0	-21.01
Panama	Mesothelioma	0	-8.11	0	-0.08	0	-17.26
Papua New Guinea	Mesothelioma	0	2.14	0	4.2	0	-14.34
Paraguay	Mesothelioma	0	-3.31	0	5.99	-0.01	-15.12
Peru	Mesothelioma	-0.1	-8.11	-0.03	-3.38	-0.07	-17.28
Philippines	Mesothelioma	0.02	1.41	0.07	6.62	-0.05	-10.19
Poland	Mesothelioma	0.15	8.64	0.04	4.11	0.04	6.35
Portugal	Mesothelioma	-0.17	-21.72	-0.12	-20.51	-0.04	-22.02
Puerto Rico	Mesothelioma	-0.01	-5.01	0	-1.52	-0.01	-19.61
Qatar	Mesothelioma	0	-19.45	0	-14.9	0	-139.36
Republic of Korea	Mesothelioma	-0.33	-23.52	-0.18	-18.73	-0.13	-31.55
Republic of Moldova	Mesothelioma	0	3.35	0	2.56	0	0.47
Romania	Mesothelioma	-0.04	-3.88	-0.01	-2.14	-0.01	-1.09
Russian Federation	Mesothelioma	0	0	0.03	0.4	0.03	0.46

Location	Cause	Attributable number (Both thousands)	Attributable proportion (Both %)	Attributable number (Male thousands)	Attributable proportion (Male %)	Attributable number (Female thousands)	Attributable proportion (Female %)
Rwanda	Mesothelioma	-0.03	-10.6	-0.01	-5.44	-0.02	-18.93
Saint Kitts and Nevis	Mesothelioma	0	-2.3	0	-0.98	0	-12.77
Saint Lucia	Mesothelioma	0	-6.31	0	0.58	0	-10.1
Saint Vincent and the Grenadines	Mesothelioma	0	-4.31	0	3.45	0	-9.04
Samoa	Mesothelioma	0	-0.29	0	0.9	0	-10.05
San Marino	Mesothelioma	0	-12.13	0	-11.23	0	-12.42
Sao Tome and Principe	Mesothelioma	0	-5.63	0	2.35	0	-14.65
Saudi Arabia	Mesothelioma	-0.01	-15.46	0	-6.05	-0.01	-39.53
Senegal	Mesothelioma	-0.01	-4.02	0	2.41	-0.02	-14
Serbia	Mesothelioma	-0.02	-2.82	-0.01	-1.82	0	-0.68
Seychelles	Mesothelioma	0	-1.51	0	2.73	0	-11.17
Sierra Leone	Mesothelioma	0	-3.12	0	1.7	-0.01	-10.73
Singapore	Mesothelioma	-0.05	-14.16	-0.03	-11.86	-0.02	-21.31
Slovakia	Mesothelioma	-0.01	-2.63	0	-2.47	0	-0.54
Slovenia	Mesothelioma	-0.11	-26.53	-0.06	-27.04	-0.01	-3.47
Solomon Islands	Mesothelioma	0	0.41	0	1.83	0	-15.33
Somalia	Mesothelioma	0	-2.01	0	3.36	-0.01	-12.63
South Africa	Mesothelioma	0.1	3.18	0.15	5.93	-0.05	-6.95
South Sudan	Mesothelioma	0	-0.98	0	2.25	0	-9.82
Spain	Mesothelioma	-1.18	-19.3	-0.77	-17.15	-0.34	-21.13

Location	Cause	Attributable number (Both thousands)	Attributable proportion (Both %)	Attributable number (Male thousands)	Attributable proportion (Male %)	Attributable number (Female thousands)	Attributable proportion (Female %)
Sri Lanka	Mesothelioma	-0.04	-7.91	-0.01	-1.61	-0.03	-18.25
Sudan	Mesothelioma	-0.01	-4.19	0	-1.11	0	-19.58
Suriname	Mesothelioma	0	-5.3	0	4.57	0	-12.68
Sweden	Mesothelioma	-0.37	-13.35	-0.29	-12.6	-0.07	-14.4
Switzerland	Mesothelioma	-0.61	-26.93	-0.41	-23.23	-0.08	-15.8
Syrian Arab Republic	Mesothelioma	-0.01	-7.23	0	-2.92	0	-22.4
Taiwan (Province of China)	Mesothelioma	-0.05	-5.53	-0.01	-1.09	-0.04	-15.88
Tajikistan	Mesothelioma	0.01	6.82	0.01	6.82	0.01	7.49
Thailand	Mesothelioma	-0.1	-2.68	0	-0.1	-0.09	-14.98
Timor-Leste	Mesothelioma	0	-1.91	0	4.28	0	-17.06
Togo	Mesothelioma	0	-3.27	0	6.09	-0.01	-14.7
Tokelau	Mesothelioma	0	-1.69	0	-0.82	0	-10.13
Tonga	Mesothelioma	0	0.71	0	1.91	0	-8.67
Trinidad and Tobago	Mesothelioma	0	-3.74	0	0.57	0	-12.73
Tunisia	Mesothelioma	0	-4.3	0	-1.05	0	-21.31
Turkey	Mesothelioma	-1.21	-10.14	-0.49	-5.96	-0.71	-19.52
Turkmenistan	Mesothelioma	0	5.03	0	5.07	0	5.48
Tuvalu	Mesothelioma	0	-2.71	0	-1.59	0	-11.11
Uganda	Mesothelioma	-0.03	-8.56	0	-1.6	-0.03	-21.34
Ukraine	Mesothelioma	0.11	1.8	0.12	3.67	0.03	0.99

Location	Cause	Attributable number (Both thousands)	Attributable proportion (Both %)	Attributable number (Male thousands)	Attributable proportion (Male %)	Attributable number (Female thousands)	Attributable proportion (Female %)
United Arab Emirates	Mesothelioma	0	-1.78	0.01	3.69	-0.01	-39.43
United Kingdom	Mesothelioma	-3.1	-7.77	-2.04	-6.05	-0.73	-11.74
United Republic of Tanzania	Mesothelioma	-0.04	-4.92	0	0.45	-0.04	-14.08
United States of America	Mesothelioma	-1.61	-3.49	-0.48	-1.32	-0.96	-9.89
United States Virgin Islands	Mesothelioma	0	-0.53	0	3.33	0	-8.45
Uruguay	Mesothelioma	-0.01	-5.98	0	-1.74	-0.01	-18.49
Uzbekistan	Mesothelioma	0.03	11.75	0.02	18.85	0.02	15.03
Vanuatu	Mesothelioma	0	2.83	0	4.36	0	-14.22
Venezuela (Bolivarian Republic of)	Mesothelioma	-0.02	-5.29	0	1.94	-0.03	-13.62
Viet Nam	Mesothelioma	-0.09	-5.43	0.01	1.26	-0.11	-20
Yemen	Mesothelioma	0	-2.06	0	1.57	0	-21.63
Zambia	Mesothelioma	-0.01	-4.61	0	2.13	-0.01	-17.06
Zimbabwe	Mesothelioma	0.01	5.29	0.01	9.78	0	-2.83

Table S8. Absolute (the number) and relative contribution (the proportion) associated with disease severity changes for nasopharynx cancer (NPC) by sex at global, SDI regional, GBD regional level between 1990 and 2021.

Location	Cause	Attributable number (Both thousands)	Attributable proportion (Both %)	Attributable number (Male thousands)	Attributable proportion (Male %)	Attributable number (Female thousands)	Attributable proportion (Female %)
Global	Nasopharynx cancer	-1665.44	-75.11	-1233.38	-81.77	-432.56	-61.01
High SDI	Nasopharynx cancer	-95.58	-48.88	-74.88	-50.88	-21.32	-44.05
High-middle SDI	Nasopharynx cancer	-780.4	-115.66	-606.32	-123.86	-179.87	-97.12
Middle SDI	Nasopharynx cancer	-717.58	-76.37	-514.61	-83.71	-204.95	-63.09
Low-middle SDI	Nasopharynx cancer	-54.97	-18.76	-32.88	-17.53	-22.53	-21.39
Low SDI	Nasopharynx cancer	-16.5	-14.52	-9.22	-13.42	-7.41	-16.5
High-income Asia Pacific	Nasopharynx cancer	-10.61	-45.61	-8.44	-46.36	-2.23	-44.09
High-income North America	Nasopharynx cancer	-11.97	-34.92	-9.3	-38.87	-2.85	-27.6
Western Europe	Nasopharynx cancer	-35.11	-45.74	-27.33	-45.32	-7.89	-47.88
Australasia	Nasopharynx cancer	-1.29	-37.04	-0.95	-34.81	-0.35	-46.3
Eastern Europe	Nasopharynx cancer	-3.44	-12.24	-1.29	-5.89	-1.48	-23.89
Central Europe	Nasopharynx cancer	-5.64	-31.58	-4.32	-32.41	-1.35	-29.67
Southern Latin America	Nasopharynx cancer	-1.17	-24.75	-0.83	-23.3	-0.33	-28.78
East Asia	Nasopharynx cancer	-1451.19	-108.04	-1085.85	-117.86	-374.3	-88.73
Central Asia	Nasopharynx cancer	-0.91	-15.12	-0.58	-14.84	-0.36	-16.64
North Africa and Middle East	Nasopharynx cancer	-27.84	-53.55	-18.34	-51.73	-9.56	-57.81
Andean Latin America	Nasopharynx cancer	-0.44	-42.03	-0.24	-40.88	-0.2	-44.4
Southeast Asia	Nasopharynx cancer	-88.21	-45.67	-58.85	-47.88	-30.87	-43.94

Location	Cause	Attributable number (Both thousands)	Attributable proportion (Both %)	Attributable number (Male thousands)	Attributable proportion (Male %)	Attributable number (Female thousands)	Attributable proportion (Female %)
Tropical Latin America	Nasopharynx cancer	-2.81	-40.09	-1.89	-36.97	-0.91	-47.5
Southern Sub-Saharan Africa	Nasopharynx cancer	-0.23	-4.19	-0.18	-5.09	-0.05	-2.44
Caribbean	Nasopharynx cancer	-0.69	-20.13	-0.51	-22.81	-0.18	-15.09
Central Latin America	Nasopharynx cancer	-2.24	-35.01	-1.4	-32.72	-0.84	-40.11
South Asia	Nasopharynx cancer	-70.26	-21.13	-42.05	-19.33	-28.48	-24.76
Central Sub-Saharan Africa	Nasopharynx cancer	-0.42	-11.75	-0.26	-11.66	-0.17	-11.95
Oceania	Nasopharynx cancer	-0.07	-5.9	-0.05	-5.5	-0.02	-6.49
Western Sub-Saharan Africa	Nasopharynx cancer	-3.09	-14.02	-1.56	-11.53	-1.49	-17.59
Eastern Sub-Saharan Africa	Nasopharynx cancer	-8.32	-16.01	-4.61	-14.93	-3.78	-17.94
Afghanistan	Nasopharynx cancer	-0.2	-10.32	-0.07	-9.07	-0.13	-11.07
Albania	Nasopharynx cancer	-0.11	-34.68	-0.07	-33.17	-0.04	-38.87
Algeria	Nasopharynx cancer	-6.53	-56.82	-3.9	-52.86	-2.65	-64.14
American Samoa	Nasopharynx cancer	0	-12.89	0	-10.75	0	-15.64
Andorra	Nasopharynx cancer	0	-53.55	0	-53.2	0	-63.2
Angola	Nasopharynx cancer	-0.13	-17.88	-0.08	-16.77	-0.05	-19.64
Antigua and Barbuda	Nasopharynx cancer	0	-22.53	0	-22.76	0	-23.64
Argentina	Nasopharynx cancer	-0.72	-20.37	-0.52	-19.03	-0.2	-24.19
Armenia	Nasopharynx cancer	-0.04	-22.57	-0.03	-24.74	-0.01	-22.04
Australia	Nasopharynx cancer	-1.12	-36.56	-0.81	-34.19	-0.31	-45.73
Austria	Nasopharynx cancer	-0.48	-44.39	-0.32	-43.05	-0.15	-45.26

Location	Cause	Attributable number (Both thousands)	Attributable proportion (Both %)	Attributable number (Male thousands)	Attributable proportion (Male %)	Attributable number (Female thousands)	Attributable proportion (Female %)
Azerbaijan	Nasopharynx cancer	-0.05	-17.32	-0.04	-17.87	-0.02	-17.96
Bahamas	Nasopharynx cancer	-0.01	-22.14	0	-22.76	0	-23.52
Bahrain	Nasopharynx cancer	-0.07	-103.75	-0.06	-106.04	-0.01	-99.17
Bangladesh	Nasopharynx cancer	-9.12	-29.03	-5.15	-25.34	-3.96	-35.75
Barbados	Nasopharynx cancer	-0.01	-20.11	0	-21.15	0	-19.42
Belarus	Nasopharynx cancer	-0.26	-16.78	-0.19	-14.81	-0.06	-24.08
Belgium	Nasopharynx cancer	-0.75	-42.35	-0.55	-41.64	-0.2	-44.62
Belize	Nasopharynx cancer	0	-27.46	0	-28.82	0	-31.01
Benin	Nasopharynx cancer	-0.02	-16.41	-0.01	-16.33	-0.01	-17.54
Bermuda	Nasopharynx cancer	-0.01	-57.37	0	-60.8	0	-48.81
Bhutan	Nasopharynx cancer	-0.04	-22.44	-0.02	-21.16	-0.01	-25.27
Bolivia (Plurinational State of)	Nasopharynx cancer	-0.06	-21.3	-0.03	-20.82	-0.03	-22.42
Bosnia and Herzegovina	Nasopharynx cancer	-0.05	-26.03	-0.03	-25.4	-0.02	-28.15
Botswana	Nasopharynx cancer	-0.01	-9.01	-0.01	-9.27	0	-9.46
Brazil	Nasopharynx cancer	-2.78	-40.1	-1.87	-36.95	-0.9	-47.54
Brunei Darussalam	Nasopharynx cancer	-0.05	-30.63	-0.03	-30.89	-0.01	-30.83
Bulgaria	Nasopharynx cancer	-0.35	-39.34	-0.29	-43.79	-0.04	-18.26
Burkina Faso	Nasopharynx cancer	-0.03	-10	-0.02	-10.24	-0.01	-10.74
Burundi	Nasopharynx cancer	-0.2	-10.64	-0.13	-11.49	-0.07	-9.83
Cabo Verde	Nasopharynx cancer	0	-179.15	0	-196.35	0	-159.66

Location	Cause	Attributable number (Both thousands)	Attributable proportion (Both %)	Attributable number (Male thousands)	Attributable proportion (Male %)	Attributable number (Female thousands)	Attributable proportion (Female %)
Cambodia	Nasopharynx cancer	-1.28	-34.09	-0.75	-35.14	-0.57	-35.12
Cameroon	Nasopharynx cancer	-0.05	-16.92	-0.04	-16.82	-0.02	-19.13
Canada	Nasopharynx cancer	-1.71	-40.61	-1.25	-40.73	-0.46	-40.79
Central African Republic	Nasopharynx cancer	-0.01	-3.01	-0.01	-3.65	0	-2.05
Chad	Nasopharynx cancer	-0.01	-6.11	-0.01	-6.72	0	-6.01
Chile	Nasopharynx cancer	-0.29	-49.5	-0.2	-48.25	-0.09	-52.29
China	Nasopharynx cancer	-1438.03	-110.64	-1075.68	-120.87	-371.27	-90.62
Colombia	Nasopharynx cancer	-0.86	-44.1	-0.53	-41.54	-0.33	-48.3
Comoros	Nasopharynx cancer	-0.01	-11.4	-0.01	-10.7	-0.01	-12.4
Congo	Nasopharynx cancer	-0.04	-17.57	-0.02	-18.35	-0.01	-16.72
Cook Islands	Nasopharynx cancer	0	-43.77	0	-41.11	0	-53
Costa Rica	Nasopharynx cancer	-0.1	-34.39	-0.07	-34.13	-0.03	-37.33
Coted'Ivoire	Nasopharynx cancer	-0.11	-16.47	-0.08	-15.44	-0.03	-19.87
Croatia	Nasopharynx cancer	-0.15	-19.8	-0.1	-16.52	-0.04	-31.02
Cuba	Nasopharynx cancer	-0.35	-31.45	-0.27	-34.11	-0.1	-29.5
Cyprus	Nasopharynx cancer	-0.05	-91.33	-0.04	-97.06	-0.01	-80.26
Czechia	Nasopharynx cancer	-0.95	-51.8	-0.79	-58.45	-0.14	-28.82
Democratic People's Republic of Korea	Nasopharynx cancer	-3.55	-27.91	-2.33	-28.3	-1.25	-27.91
Democratic Republic of the Congo	Nasopharynx cancer	-0.22	-9.62	-0.13	-9.44	-0.09	-9.96

Location	Cause	Attributable number (Both thousands)	Attributable proportion (Both %)	Attributable number (Male thousands)	Attributable proportion (Male %)	Attributable number (Female thousands)	Attributable proportion (Female %)
Denmark	Nasopharynx cancer	-0.26	-56.82	-0.19	-61.35	-0.06	-42.04
Djibouti	Nasopharynx cancer	-0.02	-16.19	-0.01	-15.27	-0.01	-18.63
Dominica	Nasopharynx cancer	0	-7.29	0	-8.39	0	-7.37
Dominican Republic	Nasopharynx cancer	-0.1	-19.24	-0.05	-16.34	-0.05	-23.93
Ecuador	Nasopharynx cancer	-0.09	-34.88	-0.05	-38.13	-0.04	-33.46
Egypt	Nasopharynx cancer	-0.35	-32.36	-0.22	-33.41	-0.13	-32.29
El Salvador	Nasopharynx cancer	-0.08	-40.46	-0.05	-37.69	-0.03	-46.08
Equatorial Guinea	Nasopharynx cancer	-0.01	-38.83	-0.01	-40.94	0	-37.66
Eritrea	Nasopharynx cancer	-0.14	-13.82	-0.09	-15.35	-0.05	-12.16
Estonia	Nasopharynx cancer	-0.07	-16.62	-0.05	-15.32	-0.02	-20.37
Eswatini	Nasopharynx cancer	0	-2.21	0	-1.91	0	-2.76
Ethiopia	Nasopharynx cancer	-3.44	-20.23	-1.83	-20.38	-1.65	-20.58
Fiji	Nasopharynx cancer	0	-8.46	0	-8.22	0	-9.3
Finland	Nasopharynx cancer	-0.17	-46.95	-0.11	-46.83	-0.06	-47.43
France	Nasopharynx cancer	-12.25	-56.23	-10.13	-53.24	-1.94	-70.14
Gabon	Nasopharynx cancer	-0.01	-14.6	-0.01	-14.58	0	-15.09
Gambia	Nasopharynx cancer	0	-14.44	0	-11.94	0	-17.4
Georgia	Nasopharynx cancer	-0.05	-8.11	-0.04	-7.59	-0.01	-9.23
Germany	Nasopharynx cancer	-4.73	-37.11	-3.74	-37.78	-0.92	-32.09
Ghana	Nasopharynx cancer	-0.03	-9.73	-0.02	-8.5	0	-20.44

Location	Cause	Attributable number (Both thousands)	Attributable proportion (Both %)	Attributable number (Male thousands)	Attributable proportion (Male %)	Attributable number (Female thousands)	Attributable proportion (Female %)
Greece	Nasopharynx cancer	-0.48	-25.32	-0.35	-24.69	-0.13	-27.31
Greenland	Nasopharynx cancer	-0.04	-22.89	-0.02	-21.54	-0.01	-25.25
Grenada	Nasopharynx cancer	0	-17.89	0	-18.41	0	-18.44
Guam	Nasopharynx cancer	-0.01	-9.6	-0.01	-9.49	0	-10.14
Guatemala	Nasopharynx cancer	-0.1	-29.24	-0.06	-26.5	-0.05	-34.15
Guinea	Nasopharynx cancer	-0.02	-5.92	-0.01	-4.94	-0.01	-8.27
Guinea-Bissau	Nasopharynx cancer	0	-9.9	0	-9.77	0	-10.57
Guyana	Nasopharynx cancer	0	-13.27	0	-13.55	0	-14.22
Haiti	Nasopharynx cancer	-0.07	-8.58	-0.04	-7.6	-0.03	-10.12
Honduras	Nasopharynx cancer	-0.03	-16.24	-0.01	-12.11	-0.01	-23.02
Hungary	Nasopharynx cancer	-0.65	-29.84	-0.48	-29.08	-0.18	-32.27
Iceland	Nasopharynx cancer	-0.01	-54.66	-0.01	-55.43	0	-53.67
India	Nasopharynx cancer	-57.58	-21.57	-34.85	-20.01	-23.09	-24.9
Indonesia	Nasopharynx cancer	-14.69	-25.23	-8.59	-25.22	-6.5	-26.89
Iran (Islamic Republic of)	Nasopharynx cancer	-1.16	-62.64	-0.73	-64.94	-0.45	-61
Iraq	Nasopharynx cancer	-0.83	-52.15	-0.51	-49.9	-0.32	-57.05
Ireland	Nasopharynx cancer	-0.27	-64.28	-0.19	-63.26	-0.08	-66.91
Israel	Nasopharynx cancer	-0.31	-62.21	-0.22	-62.78	-0.09	-61.31
Italy	Nasopharynx cancer	-4.33	-36.44	-3.47	-38.62	-0.89	-30.52
Jamaica	Nasopharynx cancer	-0.02	-16.48	-0.01	-16.65	-0.01	-18.83

Location	Cause	Attributable number (Both thousands)	Attributable proportion (Both %)	Attributable number (Male thousands)	Attributable proportion (Male %)	Attributable number (Female thousands)	Attributable proportion (Female %)
Japan	Nasopharynx cancer	-3.95	-32.25	-3.29	-33.63	-0.7	-28.4
Jordan	Nasopharynx cancer	-0.49	-92.83	-0.36	-101.43	-0.14	-82.99
Kazakhstan	Nasopharynx cancer	-0.39	-22.26	-0.25	-22.08	-0.15	-23.62
Kenya	Nasopharynx cancer	-0.61	-10.61	-0.28	-7.88	-0.34	-15.24
Kiribati	Nasopharynx cancer	0	-7.31	0	-7.14	0	-10.02
Kuwait	Nasopharynx cancer	-0.14	-64.42	-0.09	-68.29	-0.05	-66.79
Kyrgyzstan	Nasopharynx cancer	-0.1	-25.22	-0.07	-22.02	-0.03	-32.35
Lao People's Democratic Republic	Nasopharynx cancer	-0.44	-23.88	-0.26	-23.4	-0.18	-25.79
Latvia	Nasopharynx cancer	-0.07	-9.28	-0.05	-8.79	-0.02	-11.77
Lebanon	Nasopharynx cancer	-0.24	-61.05	-0.17	-62.26	-0.07	-61.51
Lesotho	Nasopharynx cancer	0.01	8.27	0.01	9.24	0	7.2
Liberia	Nasopharynx cancer	-0.02	-25.48	-0.01	-23.33	-0.01	-30.43
Libya	Nasopharynx cancer	-0.88	-44.35	-0.51	-38.47	-0.35	-53.75
Lithuania	Nasopharynx cancer	-0.08	-8.93	-0.06	-7.72	-0.02	-11.58
Luxembourg	Nasopharynx cancer	-0.06	-56.24	-0.05	-55.8	-0.01	-59.02
Madagascar	Nasopharynx cancer	-0.24	-8.69	-0.12	-7.35	-0.12	-10.35
Malawi	Nasopharynx cancer	-0.06	-11.25	-0.03	-10.51	-0.03	-13.2
Malaysia	Nasopharynx cancer	-14.65	-57.35	-10.59	-56.67	-4.15	-60.51
Maldives	Nasopharynx cancer	-0.02	-99.71	-0.01	-124.43	-0.01	-85.28
Mali	Nasopharynx cancer	-0.03	-10.37	-0.02	-9.49	-0.01	-12.39

Location	Cause	Attributable number (Both thousands)	Attributable proportion (Both %)	Attributable number (Male thousands)	Attributable proportion (Male %)	Attributable number (Female thousands)	Attributable proportion (Female %)
Malta	Nasopharynx cancer	-0.08	-53.88	-0.06	-53.34	-0.02	-56.36
Marshall Islands	Nasopharynx cancer	0	-11.07	0	-9.91	0	-12.98
Mauritania	Nasopharynx cancer	-0.02	-30.22	-0.01	-28.74	-0.01	-33.11
Mauritius	Nasopharynx cancer	-0.07	-35.37	-0.05	-35.62	-0.02	-35.44
Mexico	Nasopharynx cancer	-0.79	-32.16	-0.51	-29.66	-0.29	-38.66
Micronesia (Federated States of)	Nasopharynx cancer	0	-12.3	0	-10.41	0	-17.13
Monaco	Nasopharynx cancer	0	-35.55	0	-34.3	0	-36.28
Mongolia	Nasopharynx cancer	-0.04	-22.16	-0.02	-22.74	-0.02	-24.02
Montenegro	Nasopharynx cancer	0	-18.47	0	-18.45	0	-20.33
Morocco	Nasopharynx cancer	-1.85	-25.43	-1.33	-23.05	-0.51	-33.45
Mozambique	Nasopharynx cancer	0	-0.98	0	-0.22	0	-3.44
Myanmar	Nasopharynx cancer	-4.16	-23.75	-2.2	-23.82	-2.11	-25.51
Namibia	Nasopharynx cancer	-0.02	-15.64	-0.01	-15.53	-0.01	-16.76
Nauru	Nasopharynx cancer	0	-9.14	0	-7.09	0	-13.45
Nepal	Nasopharynx cancer	-1.07	-20.74	-0.56	-18.33	-0.52	-24.64
Netherlands	Nasopharynx cancer	-1.22	-56.19	-0.83	-56.88	-0.39	-53.94
New Zealand	Nasopharynx cancer	-0.17	-40.43	-0.13	-37.71	-0.04	-57.63
Nicaragua	Nasopharynx cancer	-0.04	-37.28	-0.02	-34.77	-0.02	-43.88
Niger	Nasopharynx cancer	-0.02	-10.85	-0.01	-10.1	-0.01	-12.85
Nigeria	Nasopharynx cancer	-2.67	-14.18	-1.28	-11.41	-1.36	-17.9

Location	Cause	Attributable number (Both thousands)	Attributable proportion (Both %)	Attributable number (Male thousands)	Attributable proportion (Male %)	Attributable number (Female thousands)	Attributable proportion (Female %)
Niue	Nasopharynx cancer	0	-12.72	0	-11.53	0	-16.4
North Macedonia	Nasopharynx cancer	-0.05	-25.79	-0.03	-24.75	-0.02	-29.58
Northern Mariana Islands	Nasopharynx cancer	0	-16.34	0	-18.74	0	-15.55
Norway	Nasopharynx cancer	-0.14	-44.4	-0.11	-45.21	-0.03	-42.06
Oman	Nasopharynx cancer	-0.13	-82.67	-0.09	-84.89	-0.03	-78.66
Pakistan	Nasopharynx cancer	-3.56	-12.32	-1.99	-10.01	-1.51	-16.57
Palau	Nasopharynx cancer	0	-20.21	0	-24.58	0	-20.17
Palestine	Nasopharynx cancer	-0.05	-49.29	-0.03	-50.65	-0.02	-48.84
Panama	Nasopharynx cancer	-0.05	-40.09	-0.03	-37.04	-0.02	-47.68
Papua New Guinea	Nasopharynx cancer	-0.05	-7.81	-0.04	-7.15	-0.02	-9.01
Paraguay	Nasopharynx cancer	-0.03	-38.16	-0.02	-36.51	-0.01	-44.51
Peru	Nasopharynx cancer	-0.28	-54.58	-0.15	-50.97	-0.13	-59.61
Philippines	Nasopharynx cancer	-3.24	-16.78	-2.18	-16.57	-1.13	-18.25
Poland	Nasopharynx cancer	-1.57	-31.71	-1.14	-31.28	-0.44	-33.84
Portugal	Nasopharynx cancer	-1.17	-49.92	-0.89	-47.3	-0.28	-58.96
Puerto Rico	Nasopharynx cancer	-0.16	-36.92	-0.13	-37.13	-0.03	-37.03
Qatar	Nasopharynx cancer	-0.08	-285.79	-0.07	-337.44	-0.01	-186.13
Republic of Korea	Nasopharynx cancer	-4.18	-77.17	-3.28	-78.88	-0.94	-74.82
Republic of Moldova	Nasopharynx cancer	-0.13	-13.12	-0.08	-11.46	-0.05	-18.6
Romania	Nasopharynx cancer	-1.15	-36.45	-0.84	-37.57	-0.33	-36.09

Location	Cause	Attributable number (Both thousands)	Attributable proportion (Both %)	Attributable number (Male thousands)	Attributable proportion (Male %)	Attributable number (Female thousands)	Attributable proportion (Female %)
Russian Federation	Nasopharynx cancer	-2.57	-14.63	-0.57	-4.18	-1.15	-29.03
Rwanda	Nasopharynx cancer	-0.48	-17.24	-0.28	-17.65	-0.21	-17.33
Saint Kitts and Nevis	Nasopharynx cancer	0	-24.52	0	-27.09	0	-24.35
Saint Lucia	Nasopharynx cancer	-0.01	-29.31	0	-32.62	0	-28.18
Saint Vincent and the Grenadines	Nasopharynx cancer	0	-14.29	0	-15.31	0	-15.63
Samoa	Nasopharynx cancer	-0.01	-19.76	-0.01	-16.93	0	-25.48
San Marino	Nasopharynx cancer	0	-28.52	0	-26.65	0	-31.84
Sao Tome and Principe	Nasopharynx cancer	0	-34.02	0	-30.6	0	-35.13
Saudi Arabia	Nasopharynx cancer	-4.6	-114.22	-3.12	-103.39	-1.46	-144
Senegal	Nasopharynx cancer	-0.03	-17.18	-0.02	-16.97	-0.01	-18.65
Serbia	Nasopharynx cancer	-0.46	-30.54	-0.39	-35.59	-0.04	-9.4
Seychelles	Nasopharynx cancer	-0.01	-36.65	-0.01	-37.22	0	-35.9
Sierra Leone	Nasopharynx cancer	-0.01	-12.59	-0.01	-11.5	0	-14.64
Singapore	Nasopharynx cancer	-3.27	-60.1	-2.54	-60.93	-0.77	-60.21
Slovakia	Nasopharynx cancer	-0.2	-15.49	-0.14	-12.49	-0.05	-26.94
Slovenia	Nasopharynx cancer	-0.1	-28.94	-0.08	-28.66	-0.02	-31.15
Solomon Islands	Nasopharynx cancer	-0.01	-12.5	-0.01	-10.2	0	-17.98
Somalia	Nasopharynx cancer	-0.08	-3.99	-0.05	-3.85	-0.03	-3.86
South Africa	Nasopharynx cancer	-0.34	-8.45	-0.23	-8.34	-0.13	-9.56
South Sudan	Nasopharynx cancer	-0.07	-5.64	-0.04	-4.24	-0.03	-7.86

Location	Cause	Attributable number (Both thousands)	Attributable proportion (Both %)	Attributable number (Male thousands)	Attributable proportion (Male %)	Attributable number (Female thousands)	Attributable proportion (Female %)
Spain	Nasopharynx cancer	-4.16	-43.38	-3.1	-39.89	-1.06	-58.39
Sri Lanka	Nasopharynx cancer	-2.04	-52.75	-1.38	-53.2	-0.69	-53.43
Sudan	Nasopharynx cancer	-0.44	-24.13	-0.23	-22.03	-0.21	-27.24
Suriname	Nasopharynx cancer	-0.01	-13.18	0	-12.39	0	-15.06
Sweden	Nasopharynx cancer	-0.16	-30.01	-0.11	-29.11	-0.05	-35.9
Switzerland	Nasopharynx cancer	-0.37	-23.74	-0.26	-22.65	-0.12	-27.37
Syrian Arab Republic	Nasopharynx cancer	-0.14	-42.73	-0.07	-37.02	-0.06	-51
Taiwan (Province of China)	Nasopharynx cancer	-16.77	-54.56	-13.29	-57.57	-3.8	-49.62
Tajikistan	Nasopharynx cancer	-0.04	-6.41	-0.02	-6.88	-0.02	-7.55
Thailand	Nasopharynx cancer	-15.6	-66.01	-11.58	-70.33	-4.4	-61.48
Timor-Leste	Nasopharynx cancer	-0.04	-20.49	-0.02	-18.91	-0.02	-23.22
Togo	Nasopharynx cancer	-0.01	-16.43	-0.01	-16.03	-0.01	-18.89
Tokelau	Nasopharynx cancer	0	-16.17	0	-13.15	0	-24.88
Tonga	Nasopharynx cancer	0	-10.73	0	-9.64	0	-14.3
Trinidad and Tobago	Nasopharynx cancer	-0.02	-25.99	-0.01	-26.44	-0.01	-27.29
Tunisia	Nasopharynx cancer	-2.3	-51.31	-1.48	-48.51	-0.84	-57.85
Turkey	Nasopharynx cancer	-6.27	-54.77	-4.46	-53.62	-1.83	-58.61
Turkmenistan	Nasopharynx cancer	-0.06	-18.49	-0.04	-19.04	-0.02	-20.01
Tuvalu	Nasopharynx cancer	0	-15.36	0	-14.25	0	-18.63
Uganda	Nasopharynx cancer	-1.33	-17.53	-0.83	-15.98	-0.49	-20.18

Location	Cause	Attributable number (Both thousands)	Attributable proportion (Both %)	Attributable number (Male thousands)	Attributable proportion (Male %)	Attributable number (Female thousands)	Attributable proportion (Female %)
Ukraine	Nasopharynx cancer	-0.43	-7.22	-0.31	-6.44	-0.15	-12.63
United Arab Emirates	Nasopharynx cancer	-0.29	-119.6	-0.25	-119.93	-0.04	-120.47
United Kingdom	Nasopharynx cancer	-2.93	-42.21	-1.94	-42.87	-0.87	-36
United Republic of Tanzania	Nasopharynx cancer	-0.8	-11.75	-0.48	-11.02	-0.33	-13.22
United States of America	Nasopharynx cancer	-10.1	-33.77	-7.88	-37.96	-2.39	-26.18
United States Virgin Islands	Nasopharynx cancer	0	-12.58	0	-13.14	0	-14.29
Uruguay	Nasopharynx cancer	-0.12	-20.35	-0.09	-19.62	-0.03	-22.57
Uzbekistan	Nasopharynx cancer	-0.21	-12.79	-0.13	-12.9	-0.1	-14.56
Vanuatu	Nasopharynx cancer	0	-7.33	0	-6.16	0	-8.8
Venezuela (Bolivarian Republic of)	Nasopharynx cancer	-0.26	-33.71	-0.16	-33.28	-0.1	-37.35
Viet Nam	Nasopharynx cancer	-26.25	-67.78	-18.33	-73.34	-8.7	-63.32
Yemen	Nasopharynx cancer	-0.17	-17.4	-0.09	-16.35	-0.07	-18.96
Zambia	Nasopharynx cancer	-0.38	-19.26	-0.24	-21.05	-0.17	-19.39
Zimbabwe	Nasopharynx cancer	0.06	7.41	0.03	6.61	0.03	9.4

Table S9. Absolute (the number) and relative contribution (the proportion) associated with disease severity changes for tracheal, bronchus, and lung cancer (TBLC), by sex at global, SDI regional, GBD regional level between 1990 and 2021.

Location	Cause	Attributable number (Both thousands)	Attributable proportion (Both %)	Attributable number (Male thousands)	Attributable proportion (Male %)	Attributable number (Female thousands)	Attributable proportion (Female %)
Global	Tracheal, bronchus, and lung cancer	-12228.72	-43.09	-8035.21	-37.84	-3805.45	-53.24
High SDI	Tracheal, bronchus, and lung cancer	-5694.7	-54.8	-3745.09	-50.15	-1791.51	-61.27
High-middle SDI	Tracheal, bronchus, and lung cancer	-5367.75	-54.4	-3662.53	-46.52	-1651.03	-82.75
Middle SDI	Tracheal, bronchus, and lung cancer	-3452.78	-55.35	-2265.86	-50.91	-1172.35	-65.58
Low-middle SDI	Tracheal, bronchus, and lung cancer	-128.24	-8.77	-82.41	-7.42	-44.19	-12.55
Low SDI	Tracheal, bronchus, and lung cancer	-20.88	-5.47	-15.32	-5.11	-5.63	-6.86
High-income Asia Pacific	Tracheal, bronchus, and lung cancer	-1021.3	-86.26	-749.63	-86	-276.31	-88.46
High-income North America	Tracheal, bronchus, and lung cancer	-1508.83	-36.25	-775.19	-28.84	-642.68	-43.59
Western Europe	Tracheal, bronchus, and lung cancer	-2940.94	-59.71	-1966.85	-49.89	-915.53	-93.11
Australasia	Tracheal, bronchus, and lung cancer	-151.02	-80.8	-89.99	-67.13	-56.47	-106.83
Eastern Europe	Tracheal, bronchus, and lung cancer	-528.82	-18.16	-430.17	-17.3	-95.06	-22.37
Central Europe	Tracheal, bronchus, and lung cancer	-360.02	-21.84	-254.12	-18.4	-96.67	-36.2
Southern Latin America	Tracheal, bronchus, and lung cancer	-59.43	-16.76	-39.64	-13.56	-17.26	-27.74
East Asia	Tracheal, bronchus, and lung cancer	-7394.16	-93	-4767.62	-86.17	-2603.97	-107.69
Central Asia	Tracheal, bronchus, and lung cancer	-20.76	-5.18	-16.57	-5.04	-4.21	-5.84

Location	Cause	Attributable number (Both thousands)	Attributable proportion (Both %)	Attributable number (Male thousands)	Attributable proportion (Male %)	Attributable number (Female thousands)	Attributable proportion (Female %)
North Africa and Middle East	Tracheal, bronchus, and lung cancer	-97.93	-11.11	-81.45	-10.71	-16.89	-13.98
Andean Latin America	Tracheal, bronchus, and lung cancer	-14.5	-20.26	-8.09	-17.45	-6.4	-25.42
Southeast Asia	Tracheal, bronchus, and lung cancer	-222.38	-16.11	-145.4	-14.74	-76.68	-19.46
Tropical Latin America	Tracheal, bronchus, and lung cancer	-68.92	-17.06	-42.3	-14.34	-26.05	-23.86
Southern Sub-Saharan Africa	Tracheal, bronchus, and lung cancer	-4.04	-3.01	-2.37	-2.38	-1.55	-4.43
Caribbean	Tracheal, bronchus, and lung cancer	-42.15	-30.1	-34.03	-33.83	-9.49	-24.06
Central Latin America	Tracheal, bronchus, and lung cancer	-70.18	-22.8	-29.93	-14.75	-36.19	-34.5
South Asia	Tracheal, bronchus, and lung cancer	-106.42	-10.39	-75.8	-9.04	-30.91	-16.66
Central Sub-Saharan Africa	Tracheal, bronchus, and lung cancer	-3.45	-5.3	-2.61	-5.15	-0.81	-5.64
Oceania	Tracheal, bronchus, and lung cancer	-0.63	-4.65	-0.42	-4.12	-0.22	-6.5
Western Sub-Saharan Africa	Tracheal, bronchus, and lung cancer	-3.46	-4.32	-2.4	-3.85	-1.09	-6.13
Eastern Sub-Saharan Africa	Tracheal, bronchus, and lung cancer	-10.44	-6.79	-7.87	-6.44	-2.46	-7.79
Afghanistan	Tracheal, bronchus, and lung cancer	-0.51	-2.94	-0.4	-3.37	-0.17	-3.18
Albania	Tracheal, bronchus, and lung cancer	-3.98	-25.37	-3.04	-23.11	-0.92	-35.92
Algeria	Tracheal, bronchus, and lung cancer	-2.18	-8.55	-1.8	-8.05	-0.4	-12.62

Location	Cause	Attributable number (Both thousands)	Attributable proportion (Both %)	Attributable number (Male thousands)	Attributable proportion (Male %)	Attributable number (Female thousands)	Attributable proportion (Female %)
American Samoa	Tracheal, bronchus, and lung cancer	-0.01	-5.84	-0.01	-5.38	0	-7.36
Andorra	Tracheal, bronchus, and lung cancer	-0.26	-41.96	-0.23	-39.6	-0.03	-63.87
Angola	Tracheal, bronchus, and lung cancer	-1.18	-8.13	-0.93	-7.67	-0.23	-9.42
Antigua and Barbuda	Tracheal, bronchus, and lung cancer	-0.03	-22.09	-0.03	-25.21	-0.01	-18.68
Argentina	Tracheal, bronchus, and lung cancer	-31.34	-11.79	-21.5	-9.74	-8.41	-18.62
Armenia	Tracheal, bronchus, and lung cancer	-2.92	-9.34	-2.36	-8.95	-0.56	-11.57
Australia	Tracheal, bronchus, and lung cancer	-126.01	-82.2	-76.1	-68	-45.14	-109.07
Austria	Tracheal, bronchus, and lung cancer	-55.81	-68.51	-32.13	-51.25	-21.05	-112.14
Azerbaijan	Tracheal, bronchus, and lung cancer	-2.51	-6.82	-1.99	-6.47	-0.51	-8.5
Bahamas	Tracheal, bronchus, and lung cancer	-0.12	-16.93	-0.11	-19.27	-0.02	-12.94
Bahrain	Tracheal, bronchus, and lung cancer	-0.33	-20.67	-0.25	-19.86	-0.08	-23.89
Bangladesh	Tracheal, bronchus, and lung cancer	-12.52	-11.99	-9.63	-10.56	-2.77	-20.85
Barbados	Tracheal, bronchus, and lung cancer	-0.15	-22.85	-0.13	-26.29	-0.03	-18.15
Belarus	Tracheal, bronchus, and lung cancer	-29.25	-24.31	-24.65	-23.35	-4.6	-31.18
Belgium	Tracheal, bronchus, and lung cancer	-73.02	-40.31	-51.32	-33.05	-19.15	-73.91
Belize	Tracheal, bronchus, and lung cancer	-0.04	-18.13	-0.04	-20.61	-0.01	-15.27
Benin	Tracheal, bronchus, and lung cancer	-0.14	-4.45	-0.1	-4.16	-0.04	-5.52
Bermuda	Tracheal, bronchus, and lung cancer	-0.48	-80.95	-0.39	-88.97	-0.09	-55.85
Bhutan	Tracheal, bronchus, and lung cancer	-0.04	-10.67	-0.03	-9.09	-0.01	-17.2

Location	Cause	Attributable number (Both thousands)	Attributable proportion (Both %)	Attributable number (Male thousands)	Attributable proportion (Male %)	Attributable number (Female thousands)	Attributable proportion (Female %)
Bolivia (Plurinational State of)	Tracheal, bronchus, and lung cancer	-1.22	-9.32	-0.73	-8.35	-0.51	-11.8
Bosnia and Herzegovina	Tracheal, bronchus, and lung cancer	-7.74	-17.6	-5.87	-15.77	-1.83	-26.9
Botswana	Tracheal, bronchus, and lung cancer	-0.09	-3.49	-0.06	-2.91	-0.03	-4.52
Brazil	Tracheal, bronchus, and lung cancer	-68.05	-17.08	-41.66	-14.34	-25.81	-23.92
Brunei Darussalam	Tracheal, bronchus, and lung cancer	-0.31	-32.88	-0.16	-27.02	-0.14	-40.58
Bulgaria	Tracheal, bronchus, and lung cancer	-15.18	-14.42	-13.43	-14.79	-2.22	-15.37
Burkina Faso	Tracheal, bronchus, and lung cancer	-0.24	-3.91	-0.17	-3.74	-0.06	-4.53
Burundi	Tracheal, bronchus, and lung cancer	-0.27	-5.39	-0.22	-5.83	-0.05	-4.28
Cabo Verde	Tracheal, bronchus, and lung cancer	-0.07	-11.13	-0.04	-10.15	-0.03	-12.96
Cambodia	Tracheal, bronchus, and lung cancer	-3.3	-10.97	-2.34	-10.23	-0.96	-13.42
Cameroon	Tracheal, bronchus, and lung cancer	-0.34	-3.91	-0.22	-3.41	-0.12	-5.41
Canada	Tracheal, bronchus, and lung cancer	-234.46	-62.77	-121.79	-48.65	-95.87	-77.82
Central African Republic	Tracheal, bronchus, and lung cancer	-0.08	-1.95	-0.07	-1.96	-0.01	-1.44
Chad	Tracheal, bronchus, and lung cancer	-0.07	-2.01	-0.05	-1.88	-0.02	-2.25
Chile	Tracheal, bronchus, and lung cancer	-18.7	-39.41	-11.2	-32.18	-7.12	-56.28
China	Tracheal, bronchus, and lung cancer	-7299.91	-94.52	-4713.55	-87.63	-2563.61	-109.35
Colombia	Tracheal, bronchus, and lung cancer	-27	-36.52	-11.21	-23.86	-14.45	-53.66
Comoros	Tracheal, bronchus, and lung cancer	-0.02	-5.47	-0.01	-5.3	-0.01	-5.95

Location	Cause	Attributable number (Both thousands)	Attributable proportion (Both %)	Attributable number (Male thousands)	Attributable proportion (Male %)	Attributable number (Female thousands)	Attributable proportion (Female %)
Congo	Tracheal, bronchus, and lung cancer	-0.38	-8.05	-0.29	-8.29	-0.09	-7.54
Cook Islands	Tracheal, bronchus, and lung cancer	-0.02	-21.63	-0.02	-20.26	0	-28.06
Costa Rica	Tracheal, bronchus, and lung cancer	-1.67	-34.2	-0.75	-21.74	-0.76	-53.33
Coted'Ivoire	Tracheal, bronchus, and lung cancer	-0.25	-5.43	-0.18	-5.13	-0.06	-6.37
Croatia	Tracheal, bronchus, and lung cancer	-36.13	-49.4	-24.65	-39.45	-9.55	-89.55
Cuba	Tracheal, bronchus, and lung cancer	-30.46	-37.57	-24.43	-42.19	-7.07	-30.52
Cyprus	Tracheal, bronchus, and lung cancer	-3.94	-93.23	-2.88	-83.22	-1.02	-134.28
Czechia	Tracheal, bronchus, and lung cancer	-68.5	-40.15	-46.94	-32.09	-18.46	-75.84
Democratic People's Republic of Korea	Tracheal, bronchus, and lung cancer	-12.43	-10.43	-7.77	-10.05	-4.72	-11.31
Democratic Republic of the Congo	Tracheal, bronchus, and lung cancer	-1.5	-3.88	-1.08	-3.7	-0.41	-4.35
Denmark	Tracheal, bronchus, and lung cancer	-45.64	-47.78	-25.24	-40.73	-19.89	-59.33
Djibouti	Tracheal, bronchus, and lung cancer	-0.02	-7.44	-0.02	-7.24	-0.01	-7.78
Dominica	Tracheal, bronchus, and lung cancer	-0.02	-7.82	-0.02	-9	0	-4.89
Dominican Republic	Tracheal, bronchus, and lung cancer	-1.9	-17.4	-1.45	-20.24	-0.45	-12.11
Ecuador	Tracheal, bronchus, and lung cancer	-2.01	-15.87	-1.13	-13.67	-0.88	-20.14
Egypt	Tracheal, bronchus, and lung cancer	-4.51	-8.63	-3.32	-8.28	-1.21	-9.96
El Salvador	Tracheal, bronchus, and lung cancer	-1.83	-27.51	-0.63	-16.97	-1.15	-39.01
Equatorial Guinea	Tracheal, bronchus, and lung cancer	-0.13	-19.44	-0.1	-18.17	-0.03	-26.18

Location	Cause	Attributable number (Both thousands)	Attributable proportion (Both %)	Attributable number (Male thousands)	Attributable proportion (Male %)	Attributable number (Female thousands)	Attributable proportion (Female %)
Eritrea	Tracheal, bronchus, and lung cancer	-0.25	-8.7	-0.19	-8.83	-0.06	-8.14
Estonia	Tracheal, bronchus, and lung cancer	-4.14	-18.31	-2.88	-15.21	-1.15	-31.19
Eswatini	Tracheal, bronchus, and lung cancer	0	-0.14	0	0.29	0	-0.5
Ethiopia	Tracheal, bronchus, and lung cancer	-5.7	-8.31	-4.83	-7.96	-0.88	-11.15
Fiji	Tracheal, bronchus, and lung cancer	-0.05	-4.47	-0.03	-3.92	-0.02	-5.75
Finland	Tracheal, bronchus, and lung cancer	-28.09	-57.46	-18.48	-46.42	-8.38	-92.24
France	Tracheal, bronchus, and lung cancer	-737.68	-114.86	-548.4	-97.64	-179.14	-222.25
Gabon	Tracheal, bronchus, and lung cancer	-0.17	-6.98	-0.12	-6.66	-0.04	-7.81
Gambia	Tracheal, bronchus, and lung cancer	-0.01	-3.45	-0.01	-3.09	0	-5.43
Georgia	Tracheal, bronchus, and lung cancer	-1.24	-2.56	-1.01	-2.49	-0.28	-3.44
Germany	Tracheal, bronchus, and lung cancer	-575.18	-56.22	-346.26	-42.07	-211.92	-106
Ghana	Tracheal, bronchus, and lung cancer	-0.44	-5.32	-0.31	-4.54	-0.13	-8.97
Greece	Tracheal, bronchus, and lung cancer	-37.09	-24.96	-27.04	-21.62	-8.75	-37.22
Greenland	Tracheal, bronchus, and lung cancer	-0.16	-15.33	-0.08	-12.96	-0.07	-19.04
Grenada	Tracheal, bronchus, and lung cancer	-0.03	-14.44	-0.03	-16.65	-0.01	-10.18
Guam	Tracheal, bronchus, and lung cancer	-0.07	-9.95	-0.04	-8.1	-0.03	-15.48
Guatemala	Tracheal, bronchus, and lung cancer	-1.15	-13.92	-0.44	-9.72	-0.68	-18.4
Guinea	Tracheal, bronchus, and lung cancer	-0.08	-1.37	-0.04	-0.92	-0.03	-3.05
Guinea-Bissau	Tracheal, bronchus, and lung cancer	-0.03	-3.62	-0.03	-3.58	-0.01	-3.49

Location	Cause	Attributable number (Both thousands)	Attributable proportion (Both %)	Attributable number (Male thousands)	Attributable proportion (Male %)	Attributable number (Female thousands)	Attributable proportion (Female %)
Guyana	Tracheal, bronchus, and lung cancer	-0.06	-7.56	-0.05	-8.52	-0.02	-6.84
Haiti	Tracheal, bronchus, and lung cancer	-0.55	-4.29	-0.48	-4.94	-0.12	-3.74
Honduras	Tracheal, bronchus, and lung cancer	-0.84	-11.67	-0.25	-6.25	-0.56	-17.19
Hungary	Tracheal, bronchus, and lung cancer	-46.34	-24.05	-29.22	-19	-15.62	-40.2
Iceland	Tracheal, bronchus, and lung cancer	-1.63	-75.82	-0.72	-60.3	-0.92	-96.84
India	Tracheal, bronchus, and lung cancer	-87.53	-12.14	-61.45	-10.71	-26.51	-18
Indonesia	Tracheal, bronchus, and lung cancer	-42.78	-9.49	-26.28	-8.59	-16.53	-11.41
Iran (Islamic Republic of)	Tracheal, bronchus, and lung cancer	-8.76	-11.76	-6.57	-11.53	-2.25	-12.89
Iraq	Tracheal, bronchus, and lung cancer	-2.98	-9.36	-2.11	-8.25	-0.86	-13.88
Ireland	Tracheal, bronchus, and lung cancer	-27.48	-72.04	-14.97	-58.06	-11.99	-96.98
Israel	Tracheal, bronchus, and lung cancer	-16.13	-57.99	-10.48	-51.84	-5.52	-72.7
Italy	Tracheal, bronchus, and lung cancer	-400.58	-50.4	-257.57	-38.27	-115.72	-95.11
Jamaica	Tracheal, bronchus, and lung cancer	-1.16	-15.64	-1.05	-17.6	-0.17	-11.61
Japan	Tracheal, bronchus, and lung cancer	-717.09	-75.81	-534.26	-76.84	-188.75	-75.31
Jordan	Tracheal, bronchus, and lung cancer	-0.97	-18.66	-0.75	-17.54	-0.22	-24.84
Kazakhstan	Tracheal, bronchus, and lung cancer	-11.83	-7.07	-9.55	-6.79	-2.27	-8.48
Kenya	Tracheal, bronchus, and lung cancer	-0.27	-5.79	-0.12	-3.97	-0.15	-9.13
Kiribati	Tracheal, bronchus, and lung cancer	-0.01	-3.44	0	-3.36	0	-3.61
Kuwait	Tracheal, bronchus, and lung cancer	-0.52	-22.42	-0.4	-20.93	-0.12	-28.05

Location	Cause	Attributable number (Both thousands)	Attributable proportion (Both %)	Attributable number (Male thousands)	Attributable proportion (Male %)	Attributable number (Female thousands)	Attributable proportion (Female %)
Kyrgyzstan	Tracheal, bronchus, and lung cancer	-1.42	-5.84	-1.09	-5.48	-0.32	-7.15
Lao People's Democratic Republic	Tracheal, bronchus, and lung cancer	-1.09	-7.67	-0.82	-7.34	-0.28	-9.11
Latvia	Tracheal, bronchus, and lung cancer	-7.98	-22.52	-6.58	-22.01	-1.45	-26.3
Lebanon	Tracheal, bronchus, and lung cancer	-2.6	-18.35	-1.82	-16.5	-0.76	-24.1
Lesotho	Tracheal, bronchus, and lung cancer	0.16	6.16	0.13	6.77	0.03	4.53
Liberia	Tracheal, bronchus, and lung cancer	-0.09	-5.19	-0.06	-4.85	-0.03	-6.55
Libya	Tracheal, bronchus, and lung cancer	-0.76	-6.58	-0.66	-6.28	-0.1	-9.74
Lithuania	Tracheal, bronchus, and lung cancer	-8.45	-20.1	-6.62	-18.32	-1.69	-28.87
Luxembourg	Tracheal, bronchus, and lung cancer	-3.44	-61.32	-2.37	-51.27	-1	-102.82
Madagascar	Tracheal, bronchus, and lung cancer	-0.24	-2.78	-0.16	-2.7	-0.08	-3.07
Malawi	Tracheal, bronchus, and lung cancer	-0.1	-4.2	-0.07	-3.87	-0.02	-5.59
Malaysia	Tracheal, bronchus, and lung cancer	-9.11	-23.03	-6.32	-20.87	-2.8	-30.18
Maldives	Tracheal, bronchus, and lung cancer	-0.1	-30.19	-0.08	-29.07	-0.03	-40.64
Mali	Tracheal, bronchus, and lung cancer	-0.21	-4.37	-0.13	-4.05	-0.08	-5.34
Malta	Tracheal, bronchus, and lung cancer	-1.4	-49.11	-0.94	-37.78	-0.36	-103.17
Marshall Islands	Tracheal, bronchus, and lung cancer	-0.01	-4.91	0	-5.23	0	-3.77
Mauritania	Tracheal, bronchus, and lung cancer	-0.16	-8.75	-0.1	-7.94	-0.06	-10.92
Mauritius	Tracheal, bronchus, and lung cancer	-0.41	-15.18	-0.29	-14.33	-0.12	-17.46
Mexico	Tracheal, bronchus, and lung cancer	-24.66	-16.53	-11.18	-10.91	-11.78	-25.23

Location	Cause	Attributable number (Both thousands)	Attributable proportion (Both %)	Attributable number (Male thousands)	Attributable proportion (Male %)	Attributable number (Female thousands)	Attributable proportion (Female %)
Micronesia (Federated States of)	Tracheal, bronchus, and lung cancer	-0.02	-4.54	-0.01	-4.22	0	-5.41
Monaco	Tracheal, bronchus, and lung cancer	-0.35	-46.49	-0.21	-36.12	-0.13	-71.76
Mongolia	Tracheal, bronchus, and lung cancer	-0.65	-7.15	-0.46	-6.69	-0.19	-8.61
Montenegro	Tracheal, bronchus, and lung cancer	-0.88	-11.5	-0.56	-9.34	-0.3	-18.27
Morocco	Tracheal, bronchus, and lung cancer	-2.06	-4.3	-1.9	-4.2	-0.19	-6.82
Mozambique	Tracheal, bronchus, and lung cancer	-0.03	-0.33	0.03	0.49	-0.05	-2.12
Myanmar	Tracheal, bronchus, and lung cancer	-13.06	-8.95	-8.82	-8.49	-4.2	-10
Namibia	Tracheal, bronchus, and lung cancer	-0.06	-5.86	-0.03	-5.13	-0.02	-7.09
Nauru	Tracheal, bronchus, and lung cancer	0	-3.41	0	-3.02	0	-4.38
Nepal	Tracheal, bronchus, and lung cancer	-1.32	-8.44	-0.85	-7.02	-0.48	-13.54
Netherlands	Tracheal, bronchus, and lung cancer	-105.87	-48.7	-65.04	-36.15	-38.42	-102.44
New Zealand	Tracheal, bronchus, and lung cancer	-24.44	-72.73	-13.41	-60.58	-10.93	-95.28
Nicaragua	Tracheal, bronchus, and lung cancer	-0.58	-25.11	-0.23	-15.06	-0.32	-38.76
Niger	Tracheal, bronchus, and lung cancer	-0.13	-4.05	-0.1	-4.06	-0.03	-4.57
Nigeria	Tracheal, bronchus, and lung cancer	-0.62	-4	-0.43	-3.49	-0.2	-6.5
Niue	Tracheal, bronchus, and lung cancer	0	-6.43	0	-5.98	0	-7.3
North Macedonia	Tracheal, bronchus, and lung cancer	-3	-18.32	-2.33	-16.68	-0.66	-26.98
Northern Mariana Islands	Tracheal, bronchus, and lung cancer	-0.03	-11.96	-0.02	-10.4	-0.01	-19.56

**Location**	**Cause**	**Attributable number (Both thousands)**	**Attributable proportion (Both %)**	**Attributable number (Male thousands)**	**Attributable proportion (Male %)**	**Attributable number (Female thousands)**	**Attributable proportion (Female %)**
Norway	Tracheal, bronchus, and lung cancer	-31	-83.19	-17.28	-65.76	-13.42	-122.16
Oman	Tracheal, bronchus, and lung cancer	-0.17	-15.03	-0.14	-13.89	-0.03	-21.61
Pakistan	Tracheal, bronchus, and lung cancer	-4.56	-2.5	-3.78	-2.35	-0.97	-4.56
Palau	Tracheal, bronchus, and lung cancer	-0.01	-10.21	-0.01	-9.25	-0.01	-11.37
Palestine	Tracheal, bronchus, and lung cancer	-0.42	-9.29	-0.33	-8.86	-0.09	-11.23
Panama	Tracheal, bronchus, and lung cancer	-1.4	-27.98	-0.65	-17.48	-0.58	-45.62
Papua New Guinea	Tracheal, bronchus, and lung cancer	-0.35	-4.45	-0.23	-3.99	-0.12	-6.06
Paraguay	Tracheal, bronchus, and lung cancer	-0.85	-15.03	-0.61	-13.9	-0.24	-18.83
Peru	Tracheal, bronchus, and lung cancer	-11.35	-24.76	-6.31	-21.5	-5.04	-30.56
Philippines	Tracheal, bronchus, and lung cancer	-5.96	-4.14	-3.77	-3.59	-2.2	-5.64
Poland	Tracheal, bronchus, and lung cancer	-56.4	-10.25	-31.1	-6.73	-18.69	-21.18
Portugal	Tracheal, bronchus, and lung cancer	-22.45	-31.77	-16.29	-28	-5.85	-46.78
Puerto Rico	Tracheal, bronchus, and lung cancer	-6.47	-43.65	-5.08	-48.71	-1.48	-33.67
Qatar	Tracheal, bronchus, and lung cancer	-0.34	-45.51	-0.29	-43.9	-0.05	-55.23
Republic of Korea	Tracheal, bronchus, and lung cancer	-483.19	-220.36	-344.8	-211.75	-137.97	-244.44
Republic of Moldova	Tracheal, bronchus, and lung cancer	-5.5	-13.9	-4.25	-12.94	-1.21	-18.05
Romania	Tracheal, bronchus, and lung cancer	-50.96	-23.26	-39.62	-21.41	-11.07	-32.59
Russian Federation	Tracheal, bronchus, and lung cancer	-419.81	-22.29	-337.93	-20.82	-76.59	-29.45
Rwanda	Tracheal, bronchus, and lung cancer	-0.62	-8.42	-0.43	-8.46	-0.19	-8.19

Location	Cause	Attributable number (Both thousands)	Attributable proportion (Both %)	Attributable number (Male thousands)	Attributable proportion (Male %)	Attributable number (Female thousands)	Attributable proportion (Female %)
Saint Kitts and Nevis	Tracheal, bronchus, and lung cancer	-0.02	-22.48	-0.02	-26.13	-0.01	-15.37
Saint Lucia	Tracheal, bronchus, and lung cancer	-0.07	-22.67	-0.06	-26.15	-0.02	-15.79
Saint Vincent and the Grenadines	Tracheal, bronchus, and lung cancer	-0.02	-11.22	-0.02	-13.11	0	-8.04
Samoa	Tracheal, bronchus, and lung cancer	-0.01	-6.78	-0.01	-6.39	0	-8.94
San Marino	Tracheal, bronchus, and lung cancer	-0.1	-34.28	-0.07	-29.7	-0.03	-48.42
Sao Tome and Principe	Tracheal, bronchus, and lung cancer	-0.01	-6.94	-0.01	-6.32	0	-9.23
Saudi Arabia	Tracheal, bronchus, and lung cancer	-1.49	-14.54	-1.12	-13.23	-0.38	-21.79
Senegal	Tracheal, bronchus, and lung cancer	-0.33	-5.34	-0.24	-4.97	-0.09	-7.09
Serbia	Tracheal, bronchus, and lung cancer	-38.54	-28.45	-30.12	-27.63	-8.5	-32.12
Seychelles	Tracheal, bronchus, and lung cancer	-0.03	-11.53	-0.03	-10.96	-0.01	-13.95
Sierra Leone	Tracheal, bronchus, and lung cancer	-0.11	-3.53	-0.09	-3.53	-0.02	-3.74
Singapore	Tracheal, bronchus, and lung cancer	-27.19	-152.1	-17.61	-136.12	-9.35	-189.39
Slovakia	Tracheal, bronchus, and lung cancer	-17.51	-25.88	-14.57	-24.57	-3.28	-39.32
Slovenia	Tracheal, bronchus, and lung cancer	-12.79	-53.11	-9	-44.96	-3.7	-91.02
Solomon Islands	Tracheal, bronchus, and lung cancer	-0.05	-5.62	-0.03	-5.19	-0.01	-7.24
Somalia	Tracheal, bronchus, and lung cancer	-0.12	-2.83	-0.1	-2.85	-0.02	-2.57
South Africa	Tracheal, bronchus, and lung cancer	-4.26	-3.74	-2.51	-2.97	-1.66	-5.64
South Sudan	Tracheal, bronchus, and lung cancer	-0.1	-1.92	-0.08	-1.81	-0.02	-2.33

Location	Cause	Attributable number (Both thousands)	Attributable proportion (Both %)	Attributable number (Male thousands)	Attributable proportion (Male %)	Attributable number (Female thousands)	Attributable proportion (Female %)
Spain	Tracheal, bronchus, and lung cancer	-224.95	-54.43	-188.77	-50.68	-46.64	-114.26
Sri Lanka	Tracheal, bronchus, and lung cancer	-6.93	-26.02	-4.85	-23.35	-2	-34.09
Sudan	Tracheal, bronchus, and lung cancer	-0.97	-4.9	-0.77	-4.95	-0.22	-5.13
Suriname	Tracheal, bronchus, and lung cancer	-0.1	-10.69	-0.08	-12.11	-0.02	-8.05
Sweden	Tracheal, bronchus, and lung cancer	-21.44	-32	-9.82	-22.08	-10.13	-44.97
Switzerland	Tracheal, bronchus, and lung cancer	-38.92	-57.26	-23.99	-46.89	-13.53	-80.49
Syrian Arab Republic	Tracheal, bronchus, and lung cancer	-1.79	-9.17	-1.27	-8.69	-0.51	-10.39
Taiwan (Province of China)	Tracheal, bronchus, and lung cancer	-64.43	-59.32	-37.68	-48.98	-26.43	-83.43
Tajikistan	Tracheal, bronchus, and lung cancer	-0.2	-1.37	-0.14	-1.34	-0.06	-1.61
Thailand	Tracheal, bronchus, and lung cancer	-85.67	-27.87	-53.99	-25.64	-31.47	-32.51
Timor-Leste	Tracheal, bronchus, and lung cancer	-0.06	-6.34	-0.04	-5.34	-0.03	-9.16
Togo	Tracheal, bronchus, and lung cancer	-0.09	-4.02	-0.05	-3.19	-0.03	-6.31
Tokelau	Tracheal, bronchus, and lung cancer	0	-6.97	0	-6.27	0	-8.66
Tonga	Tracheal, bronchus, and lung cancer	-0.02	-5.47	-0.02	-4.94	-0.01	-7.21
Trinidad and Tobago	Tracheal, bronchus, and lung cancer	-0.57	-22.41	-0.49	-25.01	-0.08	-14.21
Tunisia	Tracheal, bronchus, and lung cancer	-2.54	-10.08	-2.24	-9.56	-0.28	-15.33
Turkey	Tracheal, bronchus, and lung cancer	-67.12	-13.43	-57.36	-12.8	-9.29	-18.04
Turkmenistan	Tracheal, bronchus, and lung cancer	-0.41	-4.09	-0.3	-3.78	-0.11	-5.27
Tuvalu	Tracheal, bronchus, and lung cancer	0	-7.07	0	-6.93	0	-7.5

Location	Cause	Attributable number (Both thousands)	Attributable proportion (Both %)	Attributable number (Male thousands)	Attributable proportion (Male %)	Attributable number (Female thousands)	Attributable proportion (Female %)
Uganda	Tracheal, bronchus, and lung cancer	-0.79	-7.77	-0.45	-6.38	-0.31	-9.91
Ukraine	Tracheal, bronchus, and lung cancer	-90.2	-11.74	-74.67	-11.67	-16.95	-13.21
United Arab Emirates	Tracheal, bronchus, and lung cancer	-0.4	-14.81	-0.32	-14.2	-0.09	-18.82
United Kingdom	Tracheal, bronchus, and lung cancer	-450.92	-47.47	-258.42	-40.07	-184.61	-60.51
United Republic of Tanzania	Tracheal, bronchus, and lung cancer	-1.04	-5.21	-0.71	-4.99	-0.33	-5.81
United States of America	Tracheal, bronchus, and lung cancer	-1288.41	-34.02	-660.61	-27.11	-552.6	-40.91
United States Virgin Islands	Tracheal, bronchus, and lung cancer	-0.07	-17.26	-0.05	-19.36	-0.02	-12.19
Uruguay	Tracheal, bronchus, and lung cancer	-5.99	-14.54	-4.52	-12.27	-1.2	-27.48
Uzbekistan	Tracheal, bronchus, and lung cancer	-1.19	-2.01	-0.72	-1.6	-0.44	-3.11
Vanuatu	Tracheal, bronchus, and lung cancer	-0.01	-3.92	-0.01	-3.6	0	-5.38
Venezuela (Bolivarian Republic of)	Tracheal, bronchus, and lung cancer	-11.2	-22.28	-4.77	-14.7	-5.99	-33.56
Viet Nam	Tracheal, bronchus, and lung cancer	-55.95	-25.96	-39.21	-22.91	-16.42	-37.03
Yemen	Tracheal, bronchus, and lung cancer	-0.57	-4.31	-0.48	-4.29	-0.11	-5.15
Zambia	Tracheal, bronchus, and lung cancer	-0.36	-5.71	-0.2	-4.6	-0.15	-7.92
Zimbabwe	Tracheal, bronchus, and lung cancer	0.33	2.57	0.23	2.46	0.12	3.25

Table S10. Absolute (the number) and relative contribution (the proportion) associated with disease severity changes for stomach cancer (SC), by sex at global, SDI regional, GBD regional level between 1990 and 2021.

Location	Cause	Attributable number (Both thousands)	Attributable proportion (Both %)	Attributable number (Male thousands)	Attributable proportion (Male %)	Attributable number (Female thousands)	Attributable proportion (Female %)
Global	Stomach cancer	-8504.91	-36.72	-6288.22	-41.81	-2069.64	-25.49
High SDI	Stomach cancer	-1244.94	-30.41	-788.78	-30.66	-450.41	-29.6
High-middle SDI	Stomach cancer	-4518.51	-54.96	-3359.53	-61.53	-1129.75	-40.91
Middle SDI	Stomach cancer	-4846.39	-59.31	-3517.9	-65.21	-1244.44	-44.82
Low-middle SDI	Stomach cancer	-290.25	-15.57	-193.1	-16.74	-96.28	-13.55
Low SDI	Stomach cancer	-68.84	-8.7	-42.61	-9.49	-26.37	-7.7
High-income Asia Pacific	Stomach cancer	-534.28	-29.21	-333.4	-28.61	-206.44	-31.11
High-income North America	Stomach cancer	-161.91	-36.95	-108.45	-39.62	-53.75	-32.68
Western Europe	Stomach cancer	-657.79	-37.2	-415.07	-39.19	-247.62	-34.91
Australasia	Stomach cancer	-16.07	-40.37	-10.55	-41.86	-5.58	-38.14
Eastern Europe	Stomach cancer	-437.67	-19.36	-264.28	-19.39	-177.18	-19.72
Central Europe	Stomach cancer	-133.22	-20.01	-89.35	-20.53	-44.32	-19.22
Southern Latin America	Stomach cancer	-44.98	-22.39	-31.14	-23.12	-13.9	-21
East Asia	Stomach cancer	-8140.99	-74.37	-5990.58	-79.71	-2053.19	-59.83
Central Asia	Stomach cancer	-24.66	-6.89	-15.91	-7.02	-9.03	-6.89
North Africa and Middle East	Stomach cancer	-244.03	-34.77	-207.67	-49.38	-16.86	-6
Andean Latin America	Stomach cancer	-50.95	-30.47	-30.27	-32.08	-19.59	-26.9

**Location**	**Cause**	**Attributable number (Both thousands)**	**Attributable proportion (Both %)**	**Attributable number (Male thousands)**	**Attributable proportion (Male %)**	**Attributable number (Female thousands)**	**Attributable proportion (Female %)**
Southeast Asia	Stomach cancer	-260.05	-31.95	-163.21	-34.43	-92.15	-27.12
Tropical Latin America	Stomach cancer	-87.44	-19.53	-58.53	-19.35	-28.88	-19.89
Southern Sub-Saharan Africa	Stomach cancer	-1.19	-1.9	-0.93	-2.49	-0.22	-0.88
Caribbean	Stomach cancer	-9.62	-12.22	-6.12	-12.79	-3.52	-11.41
Central Latin America	Stomach cancer	-119.04	-28.06	-63.86	-26.43	-54.96	-30.09
South Asia	Stomach cancer	-191.41	-13.41	-122.95	-13.24	-68.84	-13.81
Central Sub-Saharan Africa	Stomach cancer	-6.16	-8.01	-3.75	-8.2	-2.36	-7.54
Oceania	Stomach cancer	-1.69	-10.67	-1.12	-10.71	-0.57	-10.76
Western Sub-Saharan Africa	Stomach cancer	-12.02	-6.47	-6.4	-6.02	-5.59	-7.04
Eastern Sub-Saharan Africa	Stomach cancer	-22.98	-9.17	-11.93	-8.87	-10.82	-9.32
Afghanistan	Stomach cancer	-4.76	-5.82	-4.36	-12.47	-1.45	-3.09
Albania	Stomach cancer	-2.31	-26.52	-1.61	-27.84	-0.71	-24.28
Algeria	Stomach cancer	-5.82	-31.9	-4.69	-45.34	-0.58	-7.33
American Samoa	Stomach cancer	-0.02	-13.85	-0.01	-14.03	-0.01	-15.54
Andorra	Stomach cancer	-0.05	-26.96	-0.03	-26.31	-0.02	-27.94
Angola	Stomach cancer	-1.89	-11.63	-1.21	-11.47	-0.65	-11.46
Antigua and Barbuda	Stomach cancer	-0.03	-13.81	-0.02	-14.07	-0.01	-13.52
Argentina	Stomach cancer	-14.6	-13.65	-10.02	-13.94	-4.61	-13.11
Armenia	Stomach cancer	-2.01	-11.69	-1.26	-11.5	-0.76	-12.13

Location	Cause	Attributable number (Both thousands)	Attributable proportion (Both %)	Attributable number (Male thousands)	Attributable proportion (Male %)	Attributable number (Female thousands)	Attributable proportion (Female %)
Australia	Stomach cancer	-12.32	-39.19	-8.19	-40.82	-4.14	-36.45
Austria	Stomach cancer	-15.78	-39.05	-9.57	-42.77	-6.31	-35
Azerbaijan	Stomach cancer	-3.78	-9.97	-2.55	-10.17	-1.25	-9.7
Bahamas	Stomach cancer	-0.07	-12.17	-0.04	-12.47	-0.03	-11.69
Bahrain	Stomach cancer	-0.42	-74.48	-0.34	-96.25	-0.03	-13.93
Bangladesh	Stomach cancer	-26.63	-18.45	-17.67	-18.04	-8.95	-19.32
Barbados	Stomach cancer	-0.15	-13.95	-0.1	-14.25	-0.06	-13.68
Belarus	Stomach cancer	-25.17	-23.13	-13.29	-19.99	-12.09	-28.52
Belgium	Stomach cancer	-8.35	-22.71	-5.33	-24.83	-3.1	-20.25
Belize	Stomach cancer	-0.04	-12.8	-0.02	-12.19	-0.01	-13.96
Benin	Stomach cancer	-0.45	-7.15	-0.26	-7.04	-0.19	-7.19
Bermuda	Stomach cancer	-0.08	-41.8	-0.06	-42.68	-0.02	-40.3
Bhutan	Stomach cancer	-0.1	-14.94	-0.06	-14.71	-0.04	-15.4
Bolivia (Plurinational State of)	Stomach cancer	-6.27	-15.33	-3.35	-16.1	-2.86	-14.26
Bosnia and Herzegovina	Stomach cancer	-2.67	-18.15	-1.75	-18.5	-0.93	-17.68
Botswana	Stomach cancer	-0.07	-4.58	-0.04	-4.57	-0.03	-4.54
Brazil	Stomach cancer	-86.52	-19.57	-57.94	-19.37	-28.55	-19.96
Brunei Darussalam	Stomach cancer	-0.29	-40.71	-0.16	-41.66	-0.12	-37.87
Bulgaria	Stomach cancer	-10.76	-16.26	-7.27	-17.74	-3.33	-13.21

Location	Cause	Attributable number (Both thousands)	Attributable proportion (Both %)	Attributable number (Male thousands)	Attributable proportion (Male %)	Attributable number (Female thousands)	Attributable proportion (Female %)
Burkina Faso	Stomach cancer	-0.87	-5.51	-0.49	-5.43	-0.37	-5.43
Burundi	Stomach cancer	-0.63	-7.37	-0.41	-8.55	-0.22	-5.95
Cabo Verde	Stomach cancer	-0.19	-11.64	-0.12	-12.67	-0.08	-10.41
Cambodia	Stomach cancer	-5.43	-23.91	-3.24	-25.58	-2.07	-20.69
Cameroon	Stomach cancer	-0.96	-6.45	-0.49	-5.91	-0.44	-6.78
Canada	Stomach cancer	-27.08	-48.99	-19.27	-55.98	-5.19	-24.9
Central African Republic	Stomach cancer	-0.14	-2.25	-0.1	-2.63	-0.03	-1.45
Chad	Stomach cancer	-0.25	-2.9	-0.13	-2.77	-0.1	-2.78
Chile	Stomach cancer	-27.93	-35.11	-19.29	-36.08	-8.69	-33.33
China	Stomach cancer	-8071.16	-75.18	-5945.77	-80.6	-2029.72	-60.43
Colombia	Stomach cancer	-51.96	-37.69	-29.22	-35.21	-22.78	-41.49
Comoros	Stomach cancer	-0.04	-7.23	-0.02	-7.36	-0.02	-7.1
Congo	Stomach cancer	-0.48	-10.55	-0.31	-11.71	-0.18	-9.37
Cook Islands	Stomach cancer	-0.01	-36.73	-0.01	-37.47	0	-33.61
Costa Rica	Stomach cancer	-4.17	-27.93	-2.58	-26.03	-1.53	-30.62
Coted'Ivoire	Stomach cancer	-0.41	-8.02	-0.23	-7.93	-0.18	-8.15
Croatia	Stomach cancer	-9.63	-29.15	-5.82	-27.43	-3.91	-33.15
Cuba	Stomach cancer	-4.15	-22.42	-2.6	-22.46	-1.54	-22.3
Cyprus	Stomach cancer	-1	-58.99	-0.61	-60.67	-0.39	-57.08

Location	Cause	Attributable number (Both thousands)	Attributable proportion (Both %)	Attributable number (Male thousands)	Attributable proportion (Male %)	Attributable number (Female thousands)	Attributable proportion (Female %)
Czechia	Stomach cancer	-19.02	-35.94	-11.66	-36.46	-7.43	-35.45
Democratic People's Republic of Korea	Stomach cancer	-37.37	-28.27	-24.69	-30.07	-12.15	-24.28
Democratic Republic of the Congo	Stomach cancer	-3.29	-6.97	-1.87	-6.94	-1.37	-6.75
Denmark	Stomach cancer	-5.87	-36.52	-3.94	-41.89	-1.52	-22.84
Djibouti	Stomach cancer	-0.04	-9.81	-0.02	-9.86	-0.01	-9.65
Dominica	Stomach cancer	-0.02	-5.1	-0.01	-5.38	-0.01	-4.7
Dominican Republic	Stomach cancer	-1.11	-13.84	-0.65	-13.67	-0.45	-13.84
Ecuador	Stomach cancer	-9.28	-23.2	-5.41	-23.2	-3.66	-21.94
Egypt	Stomach cancer	-12.76	-34.76	-11.62	-50.75	-0.91	-6.58
El Salvador	Stomach cancer	-5.15	-33.52	-2.55	-31.31	-2.62	-36.29
Equatorial Guinea	Stomach cancer	-0.18	-20.51	-0.12	-21.72	-0.06	-19.16
Eritrea	Stomach cancer	-0.51	-9.32	-0.3	-9.82	-0.21	-8.44
Estonia	Stomach cancer	-3.99	-29.74	-2.02	-25.82	-1.97	-35.28
Eswatini	Stomach cancer	0	-0.26	0	0.19	0	-0.34
Ethiopia	Stomach cancer	-12.15	-11.79	-6.19	-12.17	-5.92	-11.34
Fiji	Stomach cancer	-0.13	-11.16	-0.08	-11.25	-0.05	-10.81
Finland	Stomach cancer	-6.57	-31.94	-3.93	-34.58	-2.74	-29.76
France	Stomach cancer	-109.41	-59.98	-68.87	-59.41	-41.07	-61.77
Gabon	Stomach cancer	-0.17	-9.37	-0.12	-9.84	-0.06	-8.62

Location	Cause	Attributable number (Both thousands)	Attributable proportion (Both %)	Attributable number (Male thousands)	Attributable proportion (Male %)	Attributable number (Female thousands)	Attributable proportion (Female %)
Gambia	Stomach cancer	-0.02	-5.08	-0.01	-4.68	-0.01	-5.92
Georgia	Stomach cancer	-1.25	-4.18	-0.76	-4.08	-0.53	-4.61
Germany	Stomach cancer	-142.64	-36.34	-85.93	-40	-58.45	-32.89
Ghana	Stomach cancer	-1.34	-8.36	-0.53	-6.77	-0.81	-9.76
Greece	Stomach cancer	-5.36	-10.35	-3.39	-10.56	-1.99	-10.1
Greenland	Stomach cancer	-0.03	-16.69	-0.03	-17.9	-0.01	-14.26
Grenada	Stomach cancer	-0.03	-10.5	-0.01	-10.77	-0.01	-9.95
Guam	Stomach cancer	-0.04	-21.17	-0.02	-20.13	-0.01	-23.22
Guatemala	Stomach cancer	-7.49	-27.51	-3.22	-26.16	-4.29	-28.73
Guinea	Stomach cancer	-0.32	-3.29	-0.13	-2.42	-0.18	-3.99
Guinea-Bissau	Stomach cancer	-0.12	-5.26	-0.07	-5.39	-0.04	-4.66
Guyana	Stomach cancer	-0.1	-7.13	-0.06	-7.06	-0.04	-7.33
Haiti	Stomach cancer	-1.48	-5.8	-0.83	-5.86	-0.65	-5.76
Honduras	Stomach cancer	-1.53	-15.26	-0.61	-12.61	-0.9	-17.28
Hungary	Stomach cancer	-12.63	-18.46	-8.04	-18.57	-4.61	-18.33
Iceland	Stomach cancer	-0.21	-24.95	-0.13	-25.73	-0.08	-23.89
India	Stomach cancer	-159.37	-13.59	-102.11	-13.39	-57.36	-13.99
Indonesia	Stomach cancer	-59.55	-20.84	-34.43	-22.3	-23.52	-17.92
Iran (Islamic Republic of)	Stomach cancer	-67.53	-43.05	-55.27	-55.12	-4.52	-7.99

Location	Cause	Attributable number (Both thousands)	Attributable proportion (Both %)	Attributable number (Male thousands)	Attributable proportion (Male %)	Attributable number (Female thousands)	Attributable proportion (Female %)
Iraq	Stomach cancer	-5.4	-33.98	-4.36	-50.59	-0.51	-6.96
Ireland	Stomach cancer	-4.38	-37.79	-2.77	-39.42	-1.63	-35.69
Israel	Stomach cancer	-4.02	-36.22	-2.54	-37.32	-1.48	-34.48
Italy	Stomach cancer	-152.87	-41.9	-93.6	-42.57	-60.26	-41.56
Jamaica	Stomach cancer	-0.55	-9.08	-0.37	-9.08	-0.19	-9.12
Japan	Stomach cancer	-238.34	-17.9	-142.14	-16.69	-100.27	-20.89
Jordan	Stomach cancer	-1.94	-67.8	-1.36	-90.06	-0.17	-12.73
Kazakhstan	Stomach cancer	-12.17	-9.91	-7.82	-10.13	-4.53	-9.94
Kenya	Stomach cancer	-1.32	-7.15	-0.56	-5.84	-0.75	-8.42
Kiribati	Stomach cancer	-0.03	-8.9	-0.02	-9.02	-0.01	-8.12
Kuwait	Stomach cancer	-0.63	-88.01	-0.4	-97.49	-0.04	-14.23
Kyrgyzstan	Stomach cancer	-2.78	-9.87	-1.88	-10.08	-0.92	-9.62
Lao People's Democratic Republic	Stomach cancer	-1.73	-15.69	-1.1	-16.71	-0.62	-13.92
Latvia	Stomach cancer	-3.78	-16.84	-1.77	-13.62	-2.13	-22.64
Lebanon	Stomach cancer	-2.6	-44.39	-1.87	-61.26	-0.28	-10.09
Lesotho	Stomach cancer	0.13	6.71	0.09	7.4	0.05	6.11
Liberia	Stomach cancer	-0.33	-10.1	-0.18	-9.78	-0.15	-10.67
Libya	Stomach cancer	-1.07	-26.7	-0.89	-39.61	-0.1	-5.67
Lithuania	Stomach cancer	-4.36	-15.5	-2.07	-11.82	-2.24	-21.04

Location	Cause	Attributable number (Both thousands)	Attributable proportion (Both %)	Attributable number (Male thousands)	Attributable proportion (Male %)	Attributable number (Female thousands)	Attributable proportion (Female %)
Luxembourg	Stomach cancer	-0.52	-33.24	-0.34	-35.34	-0.18	-30.77
Madagascar	Stomach cancer	-0.58	-4.37	-0.29	-4.17	-0.3	-4.68
Malawi	Stomach cancer	-0.31	-6.86	-0.16	-6.34	-0.14	-7.13
Malaysia	Stomach cancer	-9.6	-43.91	-5.86	-46.68	-3.65	-39.24
Maldives	Stomach cancer	-0.14	-57.03	-0.1	-63.89	-0.04	-44
Mali	Stomach cancer	-1.62	-6.37	-0.8	-6.09	-0.79	-6.49
Malta	Stomach cancer	-0.37	-29.86	-0.25	-30.68	-0.12	-28.83
Marshall Islands	Stomach cancer	-0.01	-10.64	-0.01	-11.65	0	-9.33
Mauritania	Stomach cancer	-0.42	-13.4	-0.22	-13.28	-0.21	-13.55
Mauritius	Stomach cancer	-0.99	-31.89	-0.64	-34.39	-0.33	-26.92
Mexico	Stomach cancer	-40.17	-25.11	-20.47	-23.27	-19.64	-27.28
Micronesia (Federated States of)	Stomach cancer	-0.04	-11.6	-0.03	-11.94	-0.01	-10.76
Monaco	Stomach cancer	-0.03	-15.04	-0.02	-16.51	-0.01	-13.06
Mongolia	Stomach cancer	-2.14	-13.08	-1.29	-13.42	-0.86	-12.77
Montenegro	Stomach cancer	-0.15	-11.08	-0.1	-10.88	-0.05	-11.38
Morocco	Stomach cancer	-2.24	-18.08	-1.96	-28.41	-0.23	-4.23
Mozambique	Stomach cancer	-0.17	-1.43	-0.03	-0.47	-0.12	-2.59
Myanmar	Stomach cancer	-19.64	-17.78	-11.57	-18.92	-7.67	-15.57
Namibia	Stomach cancer	-0.06	-8.99	-0.03	-9.05	-0.02	-8.72

Location	Cause	Attributable number (Both thousands)	Attributable proportion (Both %)	Attributable number (Male thousands)	Attributable proportion (Male %)	Attributable number (Female thousands)	Attributable proportion (Female %)
Nauru	Stomach cancer	0	-9.29	0	-9.12	0	-9.99
Nepal	Stomach cancer	-3.45	-13.41	-2	-12.74	-1.46	-14.61
Netherlands	Stomach cancer	0.37	0.69	-0.56	-1.66	0.85	4.19
New Zealand	Stomach cancer	-3.78	-45.16	-2.43	-47.39	-1.45	-44.44
Nicaragua	Stomach cancer	-1.67	-29.34	-0.88	-27.92	-0.79	-31.14
Niger	Stomach cancer	-0.62	-6.23	-0.37	-6.18	-0.25	-6.36
Nigeria	Stomach cancer	-2.51	-5.91	-1.45	-5.42	-1.1	-6.98
Niue	Stomach cancer	0	-13.82	0	-14.57	0	-12.99
North Macedonia	Stomach cancer	-1.86	-18.34	-1.23	-18.44	-0.63	-18.16
Northern Mariana Islands	Stomach cancer	-0.02	-23.51	-0.01	-22.98	-0.01	-24.59
Norway	Stomach cancer	-5.39	-34.53	-3.57	-37.78	-1.84	-29.89
Oman	Stomach cancer	-1.08	-50.26	-0.9	-61.79	-0.06	-9.28
Pakistan	Stomach cancer	-3.81	-4.54	-2.16	-4.17	-1.79	-5.61
Palau	Stomach cancer	-0.02	-22.11	-0.01	-22.66	0	-19.45
Palestine	Stomach cancer	-0.77	-31.83	-0.6	-43.12	-0.06	-5.92
Panama	Stomach cancer	-1.77	-34.9	-1.05	-31.87	-0.68	-38.89
Papua New Guinea	Stomach cancer	-1.1	-10.66	-0.71	-10.61	-0.39	-10.85
Paraguay	Stomach cancer	-0.91	-16.15	-0.56	-16.33	-0.34	-15.46
Peru	Stomach cancer	-33.8	-39.16	-20.6	-41.02	-12.45	-34.51

**Location**	**Cause**	**Attributable number (Both thousands)**	**Attributable proportion (Both %)**	**Attributable number (Male thousands)**	**Attributable proportion (Male %)**	**Attributable number (Female thousands)**	**Attributable proportion (Female %)**
Philippines	Stomach cancer	-6.84	-13.65	-4.56	-13.67	-2.35	-14
Poland	Stomach cancer	-21.76	-10.11	-13.16	-9.1	-8.46	-11.95
Portugal	Stomach cancer	-31.21	-34.42	-18.91	-34.02	-12.49	-35.61
Puerto Rico	Stomach cancer	-2.51	-26.4	-1.66	-25.76	-0.85	-27.79
Qatar	Stomach cancer	-0.66	-153.16	-0.54	-178.31	-0.03	-25.21
Republic of Korea	Stomach cancer	-447	-91.7	-300.16	-97.49	-152.43	-84.9
Republic of Moldova	Stomach cancer	-4.23	-16.86	-2.36	-14.85	-1.98	-21.47
Romania	Stomach cancer	-27.56	-24.88	-20.14	-26.48	-7.21	-20.75
Russian Federation	Stomach cancer	-354.89	-22.9	-217.8	-23.59	-139.52	-22.26
Rwanda	Stomach cancer	-1.3	-10.91	-0.68	-11.43	-0.6	-10.2
Saint Kitts and Nevis	Stomach cancer	-0.03	-17.93	-0.02	-18.13	-0.01	-17.57
Saint Lucia	Stomach cancer	-0.08	-18.03	-0.05	-18.79	-0.03	-16.8
Saint Vincent and the Grenadines	Stomach cancer	-0.02	-8.26	-0.02	-8.31	-0.01	-8.12
Samoa	Stomach cancer	-0.05	-16.92	-0.03	-16.96	-0.02	-17.59
San Marino	Stomach cancer	-0.03	-16.35	-0.02	-17.24	-0.01	-15
Sao Tome and Principe	Stomach cancer	-0.03	-10.27	-0.01	-10.22	-0.01	-10.37
Saudi Arabia	Stomach cancer	-5.59	-54.36	-4.73	-71.68	-0.38	-10.33
Senegal	Stomach cancer	-0.82	-7.84	-0.49	-7.71	-0.33	-8.04
Serbia	Stomach cancer	-8.05	-22.15	-5.86	-25.07	-2.08	-16.07

Location	Cause	Attributable number (Both thousands)	Attributable proportion (Both %)	Attributable number (Male thousands)	Attributable proportion (Male %)	Attributable number (Female thousands)	Attributable proportion (Female %)
Seychelles	Stomach cancer	-0.04	-25.71	-0.03	-27.25	-0.01	-22.48
Sierra Leone	Stomach cancer	-0.33	-5.63	-0.22	-5.89	-0.11	-5.42
Singapore	Stomach cancer	-7.97	-86.67	-4.92	-89.03	-3.09	-84.13
Slovakia	Stomach cancer	-6.33	-24.28	-4.14	-24.45	-2.19	-24
Slovenia	Stomach cancer	-5.07	-45.3	-3.42	-48.36	-1.74	-42.2
Solomon Islands	Stomach cancer	-0.14	-13.48	-0.1	-13.4	-0.04	-14.73
Somalia	Stomach cancer	-0.38	-3.25	-0.22	-3.26	-0.14	-2.83
South Africa	Stomach cancer	-2.08	-4.59	-1.28	-4.56	-0.73	-4.25
South Sudan	Stomach cancer	-0.23	-3.04	-0.15	-2.9	-0.08	-3.37
Spain	Stomach cancer	-77.95	-40.51	-49.25	-40.88	-29.5	-41
Sri Lanka	Stomach cancer	-9.88	-39.1	-5.94	-40.4	-3.83	-36.17
Sudan	Stomach cancer	-9.2	-16.71	-7.6	-25.01	-0.94	-3.82
Suriname	Stomach cancer	-0.06	-8.42	-0.04	-8.25	-0.02	-8.75
Sweden	Stomach cancer	-7.7	-26.67	-4.99	-28.91	-2.78	-23.92
Switzerland	Stomach cancer	-6.25	-26.54	-4.06	-27.57	-2.18	-24.66
Syrian Arab Republic	Stomach cancer	-3.9	-33.89	-3.24	-48.67	-0.34	-6.9
Taiwan (Province of China)	Stomach cancer	-43.05	-54.27	-29.76	-53.09	-13.1	-56.28
Tajikistan	Stomach cancer	-0.67	-2.9	-0.43	-2.91	-0.24	-2.82
Thailand	Stomach cancer	-65.66	-56.42	-43.06	-59.2	-21.86	-50.09

Location	Cause	Attributable number (Both thousands)	Attributable proportion (Both %)	Attributable number (Male thousands)	Attributable proportion (Male %)	Attributable number (Female thousands)	Attributable proportion (Female %)
Timor-Leste	Stomach cancer	-0.15	-16.43	-0.08	-16.43	-0.07	-16.01
Togo	Stomach cancer	-0.29	-7.03	-0.13	-5.83	-0.15	-8.11
Tokelau	Stomach cancer	0	-15.5	0	-15.9	0	-15.02
Tonga	Stomach cancer	-0.04	-14.2	-0.03	-14.03	-0.01	-13.55
Trinidad and Tobago	Stomach cancer	-0.32	-14.24	-0.2	-14.39	-0.12	-14
Tunisia	Stomach cancer	-2.92	-35.41	-2.24	-48.32	-0.25	-6.98
Turkey	Stomach cancer	-108.46	-45.5	-89.93	-58.1	-7.08	-8.48
Turkmenistan	Stomach cancer	-0.86	-6.97	-0.54	-7.06	-0.32	-6.9
Tuvalu	Stomach cancer	-0.01	-15.37	0	-16.39	0	-13.04
Uganda	Stomach cancer	-1.43	-9.02	-0.88	-8.59	-0.53	-9.4
Ukraine	Stomach cancer	-40.72	-7.93	-25.82	-8.1	-15.77	-8.11
United Arab Emirates	Stomach cancer	-1.39	-70.58	-1.16	-89.92	-0.08	-12.07
United Kingdom	Stomach cancer	-57.82	-25.39	-41	-28.82	-16.19	-18.94
United Republic of Tanzania	Stomach cancer	-1.86	-6.53	-1.03	-6.45	-0.82	-6.58
United States of America	Stomach cancer	-135.9	-35.51	-88.53	-37.02	-48.29	-33.63
United States Virgin Islands	Stomach cancer	-0.03	-10.26	-0.02	-9.84	-0.01	-11.16
Uruguay	Stomach cancer	-2.08	-14.47	-1.39	-14.8	-0.69	-13.91
Uzbekistan	Stomach cancer	-2.55	-3.64	-1.53	-3.44	-1.05	-4.06
Vanuatu	Stomach cancer	-0.04	-9.65	-0.03	-9.44	-0.01	-10.74

Location	Cause	Attributable number (Both thousands)	Attributable proportion (Both %)	Attributable number (Male thousands)	Attributable proportion (Male %)	Attributable number (Female thousands)	Attributable proportion (Female %)
Venezuela (Bolivarian Republic of)	Stomach cancer	-10.91	-22.7	-6.22	-21.48	-4.78	-25.03
Viet Nam	Stomach cancer	-67.56	-41.02	-44.81	-43.72	-22.14	-35.59
Yemen	Stomach cancer	-4.73	-13.51	-4.41	-20.73	-0.53	-3.83
Zambia	Stomach cancer	-0.72	-8.17	-0.35	-7.45	-0.34	-8.43
Zimbabwe	Stomach cancer	0.7	5.73	0.35	5.67	0.36	6.04

Table S11. Absolute (the number) and relative contribution (the proportion) associated with disease severity changes for cervical cancer (CC), by sex at global, SDI regional, GBD regional level between 1990 and 2021.

Location	Cause	Attributable number (Female thousands)	Attributable proportion (Female %)
Global	Cervical cancer	-3546.54	-48.16
High SDI	Cervical cancer	-251.12	-28.32
High-middle SDI	Cervical cancer	-824.81	-67.78
Middle SDI	Cervical cancer	-1812.33	-84.32
Low-middle SDI	Cervical cancer	-1055.88	-55.37
Low SDI	Cervical cancer	-500.82	-41.9
High-income Asia Pacific	Cervical cancer	-55.45	-38.41
High-income North America	Cervical cancer	-6.76	-3.03
Western Europe	Cervical cancer	-142.85	-36.51
Australasia	Cervical cancer	-8.66	-41.94
Eastern Europe	Cervical cancer	-130.1	-34.45
Central Europe	Cervical cancer	-113.52	-43.14
Southern Latin America	Cervical cancer	-50.82	-49.47
East Asia	Cervical cancer	-1558.29	-133.07
Central Asia	Cervical cancer	-26.1	-29.25
North Africa and Middle East	Cervical cancer	-90.39	-66.69
Andean Latin America	Cervical cancer	-81.54	-98.4
Southeast Asia	Cervical cancer	-372.5	-61.46
Tropical Latin America	Cervical cancer	-144.07	-59.63
Southern Sub-Saharan Africa	Cervical cancer	-29.22	-26.67
Caribbean	Cervical cancer	-25.85	-31.53

Location	Cause	Attributable number (Female thousands)	Attributable proportion (Female %)
Central Latin America	Cervical cancer	-222.04	-67.56
South Asia	Cervical cancer	-1094.32	-56.09
Central Sub-Saharan Africa	Cervical cancer	-72.37	-48.2
Oceania	Cervical cancer	-2.16	-16.87
Western Sub-Saharan Africa	Cervical cancer	-140.65	-43.49
Eastern Sub-Saharan Africa	Cervical cancer	-248.41	-44.55
Afghanistan	Cervical cancer	-6.36	-41.22
Albania	Cervical cancer	-1.05	-63.54
Algeria	Cervical cancer	-11.72	-66.5
American Samoa	Cervical cancer	-0.01	-21.82
Andorra	Cervical cancer	-0.01	-42.86
Angola	Cervical cancer	-20.39	-74.78
Antigua and Barbuda	Cervical cancer	-0.05	-41.28
Argentina	Cervical cancer	-30.49	-47.86
Armenia	Cervical cancer	-1.64	-29.83
Australia	Cervical cancer	-7.15	-43.02
Austria	Cervical cancer	-3.37	-32.26
Azerbaijan	Cervical cancer	-3.03	-41.07
Bahamas	Cervical cancer	-0.2	-37.46
Bahrain	Cervical cancer	-0.17	-104.34
Bangladesh	Cervical cancer	-123.45	-77.13
Barbados	Cervical cancer	-0.27	-34.82
Belarus	Cervical cancer	-6.78	-36.66

Location	Cause	Attributable number (Female thousands)	Attributable proportion (Female %)
Belgium	Cervical cancer	-3.29	-34.02
Belize	Cervical cancer	-0.19	-59.29
Benin	Cervical cancer	-3.68	-45.05
Bermuda	Cervical cancer	-0.04	-52.36
Bhutan	Cervical cancer	-0.56	-59.15
Bolivia (Plurinational State of)	Cervical cancer	-16.11	-69.78
Bosnia and Herzegovina	Cervical cancer	-2.34	-46.28
Botswana	Cervical cancer	-1.06	-37.9
Brazil	Cervical cancer	-139.47	-59.74
Brunei Darussalam	Cervical cancer	-0.16	-45.51
Bulgaria	Cervical cancer	-4.81	-27.71
Burkina Faso	Cervical cancer	-6.19	-28.03
Burundi	Cervical cancer	-5.91	-27.96
Cabo Verde	Cervical cancer	-0.32	-46.4
Cambodia	Cervical cancer	-11.56	-66.63
Cameroon	Cervical cancer	-10.26	-44.88
Canada	Cervical cancer	-7.13	-42.12
Central African Republic	Cervical cancer	-1.4	-13.08
Chad	Cervical cancer	-2.72	-24.09
Chile	Cervical cancer	-19.63	-60.31
China	Cervical cancer	-1609.16	-145.12
Colombia	Cervical cancer	-50.06	-93.62
Comoros	Cervical cancer	-0.54	-34.69

Location	Cause	Attributable number (Female thousands)	Attributable proportion (Female %)
Congo	Cervical cancer	-5.38	-59.61
Cook Islands	Cervical cancer	-0.01	-49.56
Costa Rica	Cervical cancer	-2.21	-54.14
Coted'Ivoire	Cervical cancer	-4.78	-46.59
Croatia	Cervical cancer	-2.55	-35.73
Cuba	Cervical cancer	-7.45	-38.52
Cyprus	Cervical cancer	-0.41	-77.86
Czechia	Cervical cancer	-7.59	-39.91
Democratic People's Republic of Korea	Cervical cancer	-13.35	-46.77
Democratic Republic of the Congo	Cervical cancer	-41.57	-41.98
Denmark	Cervical cancer	-2.56	-25.74
Djibouti	Cervical cancer	-0.55	-61.81
Dominica	Cervical cancer	-0.03	-17.92
Dominican Republic	Cervical cancer	-5.49	-58.3
Ecuador	Cervical cancer	-14.11	-79.43
Egypt	Cervical cancer	-7.85	-87.9
El Salvador	Cervical cancer	-10.85	-100.78
Equatorial Guinea	Cervical cancer	-1.75	-120.65
Eritrea	Cervical cancer	-5.09	-34.99
Estonia	Cervical cancer	-1.43	-42.3
Eswatini	Cervical cancer	-0.36	-23.15
Ethiopia	Cervical cancer	-94.61	-51.64
Fiji	Cervical cancer	-0.4	-17.12

Location	Cause	Attributable number (Female thousands)	Attributable proportion (Female %)
Finland	Cervical cancer	-1.45	-48.42
France	Cervical cancer	-23.93	-43.27
Gabon	Cervical cancer	-1.4	-52.4
Gambia	Cervical cancer	-0.52	-42.77
Georgia	Cervical cancer	-1.23	-11.4
Germany	Cervical cancer	-39.82	-36.03
Ghana	Cervical cancer	-15.21	-49.2
Greece	Cervical cancer	-1.99	-20.09
Greenland	Cervical cancer	-0.06	-38.61
Grenada	Cervical cancer	-0.11	-41.02
Guam	Cervical cancer	-0.02	-15.79
Guatemala	Cervical cancer	-13.77	-121.85
Guinea	Cervical cancer	-6.08	-27.44
Guinea-Bissau	Cervical cancer	-1.14	-35.92
Guyana	Cervical cancer	-0.65	-32.17
Haiti	Cervical cancer	-12.29	-37.39
Honduras	Cervical cancer	-5.48	-79.07
Hungary	Cervical cancer	-8.52	-41.39
Iceland	Cervical cancer	-0.06	-34.72
India	Cervical cancer	-931.66	-54.75
Indonesia	Cervical cancer	-116.08	-52.4
Iran (Islamic Republic of)	Cervical cancer	-8.35	-66.58
Iraq	Cervical cancer	-3.35	-85.54

Location	Cause	Attributable number (Female thousands)	Attributable proportion (Female %)
Ireland	Cervical cancer	-1.52	-53.66
Israel	Cervical cancer	-1.51	-58.2
Italy	Cervical cancer	-21.57	-94.4
Jamaica	Cervical cancer	-1.51	-29.1
Japan	Cervical cancer	-22.58	-23.9
Jordan	Cervical cancer	-1.02	-93.7
Kazakhstan	Cervical cancer	-10.81	-37.38
Kenya	Cervical cancer	-11.21	-50.95
Kiribati	Cervical cancer	-0.1	-25.58
Kuwait	Cervical cancer	-0.33	-62.09
Kyrgyzstan	Cervical cancer	-3.1	-40.44
Lao People's Democratic Republic	Cervical cancer	-4.38	-53.98
Latvia	Cervical cancer	-1.25	-25.14
Lebanon	Cervical cancer	-1.05	-59.19
Lesotho	Cervical cancer	-0.01	-0.27
Liberia	Cervical cancer	-2.94	-68.54
Libya	Cervical cancer	-1.73	-69.14
Lithuania	Cervical cancer	-1.85	-26.09
Luxembourg	Cervical cancer	-0.16	-45
Madagascar	Cervical cancer	-11.85	-33.68
Malawi	Cervical cancer	-12.4	-45.93
Malaysia	Cervical cancer	-17.37	-77.1
Maldives	Cervical cancer	-0.18	-84.39

Location	Cause	Attributable number (Female thousands)	Attributable proportion (Female %)
Mali	Cervical cancer	-8.23	-36.33
Malta	Cervical cancer	-0.11	-42.73
Marshall Islands	Cervical cancer	-0.02	-27.68
Mauritania	Cervical cancer	-3	-68.66
Mauritius	Cervical cancer	-0.52	-38.25
Mexico	Cervical cancer	-103.73	-55.36
Micronesia (Federated States of)	Cervical cancer	-0.07	-34.6
Monaco	Cervical cancer	-0.01	-26.28
Mongolia	Cervical cancer	-2.14	-73.98
Montenegro	Cervical cancer	-0.14	-22.86
Morocco	Cervical cancer	-12.29	-61.09
Mozambique	Cervical cancer	-12.74	-28.46
Myanmar	Cervical cancer	-41.14	-50.24
Namibia	Cervical cancer	-1.16	-65.92
Nauru	Cervical cancer	-0.01	-23.35
Nepal	Cervical cancer	-21.9	-61.59
Netherlands	Cervical cancer	-3.94	-37.86
New Zealand	Cervical cancer	-1.04	-25.77
Nicaragua	Cervical cancer	-5.53	-74.63
Niger	Cervical cancer	-5.34	-38.25
Nigeria	Cervical cancer	-56.85	-48.06
Niue	Cervical cancer	0	-24.97
North Macedonia	Cervical cancer	-1.39	-51.84

Location	Cause	Attributable number (Female thousands)	Attributable proportion (Female %)
Northern Mariana Islands	Cervical cancer	-0.02	-23.98
Norway	Cervical cancer	-1.37	-29.43
Oman	Cervical cancer	-0.39	-75.38
Pakistan	Cervical cancer	-23.16	-43.96
Palau	Cervical cancer	-0.02	-30.31
Palestine	Cervical cancer	-0.16	-58.91
Panama	Cervical cancer	-3.26	-58.79
Papua New Guinea	Cervical cancer	-1.48	-20.16
Paraguay	Cervical cancer	-4.54	-55.82
Peru	Cervical cancer	-47.82	-113.82
Philippines	Cervical cancer	-16.9	-34.98
Poland	Cervical cancer	-35.21	-41.38
Portugal	Cervical cancer	-7.36	-55.33
Puerto Rico	Cervical cancer	-1.55	-48.36
Qatar	Cervical cancer	-0.15	-194.42
Republic of Korea	Cervical cancer	-35.83	-76.89
Republic of Moldova	Cervical cancer	-3.15	-32.44
Romania	Cervical cancer	-34.24	-51.46
Russian Federation	Cervical cancer	-97.36	-46.84
Rwanda	Cervical cancer	-16.06	-46.73
Saint Kitts and Nevis	Cervical cancer	-0.09	-53.88
Saint Lucia	Cervical cancer	-0.19	-48.83
Saint Vincent and the Grenadines	Cervical cancer	-0.1	-27.86

Location	Cause	Attributable number (Female thousands)	Attributable proportion (Female %)
Samoa	Cervical cancer	-0.07	-36.49
San Marino	Cervical cancer	0	-25.44
Sao Tome and Principe	Cervical cancer	-0.17	-58.09
Saudi Arabia	Cervical cancer	-2.93	-154.87
Senegal	Cervical cancer	-6.13	-45.33
Serbia	Cervical cancer	-10.43	-46.1
Seychelles	Cervical cancer	-0.09	-42.46
Sierra Leone	Cervical cancer	-2.65	-40.79
Singapore	Cervical cancer	-1.89	-63.99
Slovakia	Cervical cancer	-2.75	-32.12
Slovenia	Cervical cancer	-1.1	-53.8
Solomon Islands	Cervical cancer	-0.22	-39.14
Somalia	Cervical cancer	-5.13	-17.34
South Africa	Cervical cancer	-28.06	-35.89
South Sudan	Cervical cancer	-3.71	-28.63
Spain	Cervical cancer	-11.87	-37.6
Sri Lanka	Cervical cancer	-8.76	-71.65
Sudan	Cervical cancer	-4.91	-57.87
Suriname	Cervical cancer	-0.4	-37.95
Sweden	Cervical cancer	-1.25	-17.4
Switzerland	Cervical cancer	-1.82	-28.16
Syrian Arab Republic	Cervical cancer	-1.6	-66
Taiwan (Province of China)	Cervical cancer	-13.5	-40.09

Location	Cause	Attributable number (Female thousands)	Attributable proportion (Female %)
Tajikistan	Cervical cancer	-0.73	-19.48
Thailand	Cervical cancer	-78.8	-69.93
Timor-Leste	Cervical cancer	-0.38	-48.79
Togo	Cervical cancer	-3.1	-46.38
Tokelau	Cervical cancer	0	-36.97
Tonga	Cervical cancer	-0.06	-23.06
Trinidad and Tobago	Cervical cancer	-1.24	-46.44
Tunisia	Cervical cancer	-2.4	-60.14
Turkey	Cervical cancer	-19.55	-70.67
Turkmenistan	Cervical cancer	-1.49	-40.25
Tuvalu	Cervical cancer	-0.01	-36.58
Uganda	Cervical cancer	-15.91	-45.28
Ukraine	Cervical cancer	-15.48	-12.27
United Arab Emirates	Cervical cancer	-1.04	-119.07
United Kingdom	Cervical cancer	-3.81	-4.83
United Republic of Tanzania	Cervical cancer	-24.51	-35.7
United States of America	Cervical cancer	1.16	0.57
United States Virgin Islands	Cervical cancer	-0.05	-22.85
Uruguay	Cervical cancer	-2.47	-38.22
Uzbekistan	Cervical cancer	-6.41	-34.37
Vanuatu	Cervical cancer	-0.04	-19.34
Venezuela (Bolivarian Republic of)	Cervical cancer	-31.49	-75.33
Viet Nam	Cervical cancer	-69.35	-88.84

Location	Cause	Attributable number (Female thousands)	Attributable proportion (Female %)
Yemen	Cervical cancer	-2.39	-51.27
Zambia	Cervical cancer	-13.56	-51.76
Zimbabwe	Cervical cancer	2.18	9.94

Table S12. Absolute (the number) and relative contribution (the proportion) associated with disease severity changes for prostate cancer (ProC), by sex at global, SDI regional, GBD regional level between 1990 and 2021.

Location	Cause	Attributable number (Male thousands)	Attributable proportion (Male %)
Global	Prostate cancer	-2512.85	-60.6
High SDI	Prostate cancer	-1332.3	-66.71
High-middle SDI	Prostate cancer	-878.1	-101.69
Middle SDI	Prostate cancer	-963.51	-153.31
Low-middle SDI	Prostate cancer	-532.78	-135.58
Low SDI	Prostate cancer	-194.33	-75.39
High-income Asia Pacific	Prostate cancer	-158.71	-175.4
High-income North America	Prostate cancer	-373.32	-43.94
Western Europe	Prostate cancer	-1089.78	-94.18
Australasia	Prostate cancer	-66.81	-94.62
Eastern Europe	Prostate cancer	-212.98	-98.94
Central Europe	Prostate cancer	-226.21	-123.32
Southern Latin America	Prostate cancer	-94.55	-107.2
East Asia	Prostate cancer	-674.88	-286.31
Central Asia	Prostate cancer	-17.06	-58.99
North Africa and Middle East	Prostate cancer	-253.57	-248.72
Andean Latin America	Prostate cancer	-66.47	-203.48
Southeast Asia	Prostate cancer	-221.78	-153.64
Tropical Latin America	Prostate cancer	-183.17	-133.36
Southern Sub-Saharan Africa	Prostate cancer	-32.75	-56.02
Caribbean	Prostate cancer	-50.57	-73.09

**Location**	**Cause**	**Attributable number (Male thousands)**	**Attributable proportion (Male %)**
Central Latin America	Prostate cancer	-191.15	-160.58
South Asia	Prostate cancer	-338.58	-146.71
Central Sub-Saharan Africa	Prostate cancer	-25.25	-81.51
Oceania	Prostate cancer	-1.6	-46.21
Western Sub-Saharan Africa	Prostate cancer	-149.09	-78.63
Eastern Sub-Saharan Africa	Prostate cancer	-87.63	-80.08
Afghanistan	Prostate cancer	-2.64	-66.16
Albania	Prostate cancer	-3.44	-160.36
Algeria	Prostate cancer	-3.93	-175.05
American Samoa	Prostate cancer	-0.04	-57.48
Andorra	Prostate cancer	-0.13	-101.5
Angola	Prostate cancer	-6.74	-122.77
Antigua and Barbuda	Prostate cancer	-0.19	-64.84
Argentina	Prostate cancer	-50.37	-83.42
Armenia	Prostate cancer	-2.15	-91.94
Australia	Prostate cancer	-61.95	-102.25
Austria	Prostate cancer	-17.27	-79.55
Azerbaijan	Prostate cancer	-3.06	-81.38
Bahamas	Prostate cancer	-0.43	-69.9
Bahrain	Prostate cancer	-0.78	-398.39
Bangladesh	Prostate cancer	-44.37	-176.46
Barbados	Prostate cancer	-0.63	-57.28
Belarus	Prostate cancer	-10.97	-106.53

Location	Cause	Attributable number (Male thousands)	Attributable proportion (Male %)
Belgium	Prostate cancer	-26.66	-74.51
Belize	Prostate cancer	-0.13	-79.19
Benin	Prostate cancer	-2.29	-84.54
Bermuda	Prostate cancer	-0.38	-167.48
Bhutan	Prostate cancer	-0.14	-173.44
Bolivia (Plurinational State of)	Prostate cancer	-9.08	-149.4
Bosnia and Herzegovina	Prostate cancer	-3.93	-120.27
Botswana	Prostate cancer	-1.11	-83.57
Brazil	Prostate cancer	-180.1	-133.5
Brunei Darussalam	Prostate cancer	-0.15	-154.37
Bulgaria	Prostate cancer	-12.14	-83.94
Burkina Faso	Prostate cancer	-2.97	-57.98
Burundi	Prostate cancer	-2.78	-64.1
Cabo Verde	Prostate cancer	-0.48	-123.5
Cambodia	Prostate cancer	-4.84	-163.97
Cameroon	Prostate cancer	-5.99	-96.97
Canada	Prostate cancer	-49.63	-62.47
Central African Republic	Prostate cancer	-0.43	-27.43
Chad	Prostate cancer	-1.22	-40.22
Chile	Prostate cancer	-32.77	-188.56
China	Prostate cancer	-645.59	-287.82
Colombia	Prostate cancer	-58.04	-208.77
Comoros	Prostate cancer	-0.29	-77.54

Location	Cause	Attributable number (Male thousands)	Attributable proportion (Male %)
Congo	Prostate cancer	-2.03	-113.44
Cook Islands	Prostate cancer	-0.09	-146.26
Costa Rica	Prostate cancer	-3.22	-134.91
Coted'Ivoire	Prostate cancer	-10.62	-97
Croatia	Prostate cancer	-10.63	-108.26
Cuba	Prostate cancer	-20.85	-79.88
Cyprus	Prostate cancer	-3.75	-216.84
Czechia	Prostate cancer	-29.08	-135.34
Democratic People's Republic of Korea	Prostate cancer	-3.98	-103.87
Democratic Republic of the Congo	Prostate cancer	-14.17	-68.46
Denmark	Prostate cancer	-23.6	-103.67
Djibouti	Prostate cancer	-0.37	-145.23
Dominica	Prostate cancer	-0.07	-30.74
Dominican Republic	Prostate cancer	-6.1	-81.68
Ecuador	Prostate cancer	-13.64	-164.07
Egypt	Prostate cancer	-19.06	-222.03
El Salvador	Prostate cancer	-7.02	-187.75
Equatorial Guinea	Prostate cancer	-0.78	-274.84
Eritrea	Prostate cancer	-1.5	-94.63
Estonia	Prostate cancer	-4.3	-156.39
Eswatini	Prostate cancer	-0.28	-43.45
Ethiopia	Prostate cancer	-17.25	-121.91
Fiji	Prostate cancer	-0.18	-41.71

Location	Cause	Attributable number (Male thousands)	Attributable proportion (Male %)
Finland	Prostate cancer	-19.4	-148.88
France	Prostate cancer	-218.75	-107.98
Gabon	Prostate cancer	-1.03	-90.74
Gambia	Prostate cancer	-0.09	-66.67
Georgia	Prostate cancer	-2	-33.91
Germany	Prostate cancer	-252.38	-112.96
Ghana	Prostate cancer	-11.67	-82.28
Greece	Prostate cancer	-12.42	-47.09
Greenland	Prostate cancer	-0.04	-100.77
Grenada	Prostate cancer	-0.23	-79.2
Guam	Prostate cancer	-0.06	-60.64
Guatemala	Prostate cancer	-9.06	-277.53
Guinea	Prostate cancer	-1.61	-42.29
Guinea-Bissau	Prostate cancer	-0.37	-62.94
Guyana	Prostate cancer	-0.75	-59.55
Haiti	Prostate cancer	-5.6	-55.3
Honduras	Prostate cancer	-2.26	-110.01
Hungary	Prostate cancer	-21.52	-90.71
Iceland	Prostate cancer	-0.49	-68.05
India	Prostate cancer	-255.99	-161.24
Indonesia	Prostate cancer	-66.01	-122.1
Iran (Islamic Republic of)	Prostate cancer	-40.63	-241.51
Iraq	Prostate cancer	-5.94	-193.91

Location	Cause	Attributable number (Male thousands)	Attributable proportion (Male %)
Ireland	Prostate cancer	-10.35	-106.77
Israel	Prostate cancer	-6.94	-105.71
Italy	Prostate cancer	-101.65	-75.67
Jamaica	Prostate cancer	-2.36	-48.94
Japan	Prostate cancer	-124.65	-153.89
Jordan	Prostate cancer	-2.51	-370.19
Kazakhstan	Prostate cancer	-6.52	-72.71
Kenya	Prostate cancer	-4.3	-65.48
Kiribati	Prostate cancer	-0.01	-40.71
Kuwait	Prostate cancer	-0.76	-339.11
Kyrgyzstan	Prostate cancer	-1.25	-64.51
Lao People's Democratic Republic	Prostate cancer	-1.68	-123.92
Latvia	Prostate cancer	-3.38	-78.7
Lebanon	Prostate cancer	-7.02	-325.03
Lesotho	Prostate cancer	-0.09	-6
Liberia	Prostate cancer	-1.51	-95.67
Libya	Prostate cancer	-2.1	-119.34
Lithuania	Prostate cancer	-4.17	-78.68
Luxembourg	Prostate cancer	-1.18	-107.14
Madagascar	Prostate cancer	-4.5	-49.04
Malawi	Prostate cancer	-3.27	-74.13
Malaysia	Prostate cancer	-11.69	-192.4
Maldives	Prostate cancer	-0.16	-315.34

Location	Cause	Attributable number (Male thousands)	Attributable proportion (Male %)
Mali	Prostate cancer	-1.97	-66.58
Malta	Prostate cancer	-0.66	-115.54
Marshall Islands	Prostate cancer	-0.01	-60.78
Mauritania	Prostate cancer	-2.24	-169.68
Mauritius	Prostate cancer	-0.87	-167.99
Mexico	Prostate cancer	-79.94	-135.92
Micronesia (Federated States of)	Prostate cancer	-0.05	-59.23
Monaco	Prostate cancer	-0.08	-55.09
Mongolia	Prostate cancer	-0.29	-110.65
Montenegro	Prostate cancer	-0.38	-39.77
Morocco	Prostate cancer	-7.03	-137.65
Mozambique	Prostate cancer	-1.19	-34.9
Myanmar	Prostate cancer	-18.72	-132.55
Namibia	Prostate cancer	-1.22	-115.19
Nauru	Prostate cancer	0	-36.39
Nepal	Prostate cancer	-4.54	-140.88
Netherlands	Prostate cancer	-35.79	-83.61
New Zealand	Prostate cancer	-5.89	-58.79
Nicaragua	Prostate cancer	-2.56	-165.34
Niger	Prostate cancer	-2.36	-83.71
Nigeria	Prostate cancer	-95.57	-76.42
Niue	Prostate cancer	0	-49.08
North Macedonia	Prostate cancer	-2.14	-109.79

Location	Cause	Attributable number (Male thousands)	Attributable proportion (Male %)
Northern Mariana Islands	Prostate cancer	-0.02	-81.56
Norway	Prostate cancer	-15.07	-78.77
Oman	Prostate cancer	-0.47	-224.35
Pakistan	Prostate cancer	-29.22	-67.07
Palau	Prostate cancer	-0.02	-70.02
Palestine	Prostate cancer	-1.73	-160.55
Panama	Prostate cancer	-3.61	-153.92
Papua New Guinea	Prostate cancer	-0.89	-46.93
Paraguay	Prostate cancer	-2.91	-118.8
Peru	Prostate cancer	-42.4	-231.97
Philippines	Prostate cancer	-17.9	-61.99
Poland	Prostate cancer	-87.68	-168.95
Portugal	Prostate cancer	-34.8	-126.36
Puerto Rico	Prostate cancer	-8.35	-93.23
Qatar	Prostate cancer	-0.94	-653.17
Republic of Korea	Prostate cancer	-57.47	-693.77
Republic of Moldova	Prostate cancer	-3.24	-93.68
Romania	Prostate cancer	-35.98	-149.27
Russian Federation	Prostate cancer	-139.56	-122.79
Rwanda	Prostate cancer	-6.26	-110.12
Saint Kitts and Nevis	Prostate cancer	-0.22	-120.13
Saint Lucia	Prostate cancer	-0.49	-117.34
Saint Vincent and the Grenadines	Prostate cancer	-0.18	-62.58

Location	Cause	Attributable number (Male thousands)	Attributable proportion (Male %)
Samoa	Prostate cancer	-0.05	-61.77
San Marino	Prostate cancer	-0.05	-66.79
Sao Tome and Principe	Prostate cancer	-0.06	-89.93
Saudi Arabia	Prostate cancer	-5.84	-279.12
Senegal	Prostate cancer	-4.36	-96.77
Serbia	Prostate cancer	-15.93	-110.32
Seychelles	Prostate cancer	-0.19	-126.43
Sierra Leone	Prostate cancer	-1.69	-62.4
Singapore	Prostate cancer	-3.98	-358.79
Slovakia	Prostate cancer	-8.88	-102.18
Slovenia	Prostate cancer	-5.64	-158.89
Solomon Islands	Prostate cancer	-0.12	-60.48
Somalia	Prostate cancer	-1.25	-36.02
South Africa	Prostate cancer	-26.27	-63.19
South Sudan	Prostate cancer	-2.16	-36.79
Spain	Prostate cancer	-110.33	-113.55
Sri Lanka	Prostate cancer	-9.37	-184.13
Sudan	Prostate cancer	-6.96	-145.24
Suriname	Prostate cancer	-0.34	-57.74
Sweden	Prostate cancer	-22.67	-54.37
Switzerland	Prostate cancer	-19.93	-66.43
Syrian Arab Republic	Prostate cancer	-6.9	-198.35
Taiwan (Province of China)	Prostate cancer	-20.59	-271.61

Location	Cause	Attributable number (Male thousands)	Attributable proportion (Male %)
Tajikistan	Prostate cancer	-0.48	-36.76
Thailand	Prostate cancer	-59.24	-249.44
Timor-Leste	Prostate cancer	-0.16	-130.11
Togo	Prostate cancer	-1.38	-93.7
Tokelau	Prostate cancer	0	-69.91
Tonga	Prostate cancer	-0.06	-47.3
Trinidad and Tobago	Prostate cancer	-2.95	-86.38
Tunisia	Prostate cancer	-4.33	-171.49
Turkey	Prostate cancer	-115.64	-287.95
Turkmenistan	Prostate cancer	-0.49	-71.06
Tuvalu	Prostate cancer	-0.01	-96.76
Uganda	Prostate cancer	-18.37	-82.05
Ukraine	Prostate cancer	-19.8	-26.23
United Arab Emirates	Prostate cancer	-1.84	-415.65
United Kingdom	Prostate cancer	-167.14	-84.78
United Republic of Tanzania	Prostate cancer	-15.53	-70.16
United States of America	Prostate cancer	-332.62	-43.19
United States Virgin Islands	Prostate cancer	-0.14	-48.4
Uruguay	Prostate cancer	-7.44	-71.17
Uzbekistan	Prostate cancer	-1.24	-32.62
Vanuatu	Prostate cancer	-0.03	-41.13
Venezuela (Bolivarian Republic of)	Prostate cancer	-26.54	-155.38
Viet Nam	Prostate cancer	-13.05	-186.18

Location	Cause	Attributable number (Male thousands)	Attributable proportion (Male %)
Yemen	Prostate cancer	-2.93	-136.38
Zambia	Prostate cancer	-7.15	-129
Zimbabwe	Prostate cancer	0.39	3.18

Table S13. Absolute (the number) and relative contribution (the proportion) associated with disease severity changes for breast cancer (BreC), by sex at global, SDI regional, GBD regional level between 1990 and 2021.

Location	Cause	Attributable number (Female thousands)	Attributable proportion (Female %)
Global	Breast cancer	-3866.49	-35.11
High SDI	Breast cancer	-1734.58	-43
High-middle SDI	Breast cancer	-1751.81	-59.95
Middle SDI	Breast cancer	-2154.68	-93.92
Low-middle SDI	Breast cancer	-768.43	-62.72
Low SDI	Breast cancer	-227.01	-43.6
High-income Asia Pacific	Breast cancer	-93.18	-32.98
High-income North America	Breast cancer	-506.25	-32.73
Western Europe	Breast cancer	-1152.58	-47.55
Australasia	Breast cancer	-57.01	-60.33
Eastern Europe	Breast cancer	-275.47	-29.55
Central Europe	Breast cancer	-215.56	-41.74
Southern Latin America	Breast cancer	-101.3	-49.94
East Asia	Breast cancer	-1977.52	-129.65
Central Asia	Breast cancer	-49.81	-28.92
North Africa and Middle East	Breast cancer	-320.4	-127.58
Andean Latin America	Breast cancer	-52.3	-112.89
Southeast Asia	Breast cancer	-522.55	-71.55
Tropical Latin America	Breast cancer	-215.36	-76.35
Southern Sub-Saharan Africa	Breast cancer	-18.81	-20.98
Caribbean	Breast cancer	-24.9	-30.22

Location	Cause	Attributable number (Female thousands)	Attributable proportion (Female %)
Central Latin America	Breast cancer	-200.52	-103.38
South Asia	Breast cancer	-809.86	-73.74
Central Sub-Saharan Africa	Breast cancer	-28.58	-44.5
Oceania	Breast cancer	0.7	5.79
Western Sub-Saharan Africa	Breast cancer	-120.71	-49.91
Eastern Sub-Saharan Africa	Breast cancer	-115.4	-51.85
Afghanistan	Breast cancer	-4.32	-37.17
Albania	Breast cancer	-1.77	-52.82
Algeria	Breast cancer	-14.12	-92.58
American Samoa	Breast cancer	0	-2.37
Andorra	Breast cancer	-0.13	-64.67
Angola	Breast cancer	-7.9	-74.47
Antigua and Barbuda	Breast cancer	-0.09	-43.73
Argentina	Breast cancer	-67.88	-44.4
Armenia	Breast cancer	-5.91	-38.37
Australia	Breast cancer	-47.21	-63.09
Austria	Breast cancer	-17.26	-36.33
Azerbaijan	Breast cancer	-8.01	-45.9
Bahamas	Breast cancer	-0.5	-46.13
Bahrain	Breast cancer	-1.71	-209.32
Bangladesh	Breast cancer	-84.95	-127.95
Barbados	Breast cancer	-0.52	-39.46
Belarus	Breast cancer	-14.33	-36.23

Location	Cause	Attributable number (Female thousands)	Attributable proportion (Female %)
Belgium	Breast cancer	-31.94	-41.77
Belize	Breast cancer	-0.06	-40.58
Benin	Breast cancer	-1.68	-41.96
Bermuda	Breast cancer	-0.25	-71.17
Bhutan	Breast cancer	-0.28	-72.7
Bolivia (Plurinational State of)	Breast cancer	-7.99	-83.68
Bosnia and Herzegovina	Breast cancer	-3.62	-34.59
Botswana	Breast cancer	-0.71	-40.35
Brazil	Breast cancer	-212.13	-76.57
Brunei Darussalam	Breast cancer	-0.27	-63.36
Bulgaria	Breast cancer	-9.75	-23.54
Burkina Faso	Breast cancer	-5.3	-34.27
Burundi	Breast cancer	-2.13	-29.34
Cabo Verde	Breast cancer	-0.3	-57.15
Cambodia	Breast cancer	-10.43	-77.06
Cameroon	Breast cancer	-5.84	-47.35
Canada	Breast cancer	-54.91	-40.63
Central African Republic	Breast cancer	-0.37	-10.01
Chad	Breast cancer	-0.53	-11.69
Chile	Breast cancer	-22.61	-76.94
China	Breast cancer	-1921.54	-131.5
Colombia	Breast cancer	-64.3	-133.41
Comoros	Breast cancer	-0.26	-40.79

Location	Cause	Attributable number (Female thousands)	Attributable proportion (Female %)
Congo	Breast cancer	-3.18	-69.07
Cook Islands	Breast cancer	-0.04	-53
Costa Rica	Breast cancer	-3.5	-89.47
Coted'Ivoire	Breast cancer	-6.92	-55.38
Croatia	Breast cancer	-9.07	-39.67
Cuba	Breast cancer	-13	-42.92
Cyprus	Breast cancer	-2.62	-90.97
Czechia	Breast cancer	-25.29	-49.56
Democratic People's Republic of Korea	Breast cancer	-13.79	-39.6
Democratic Republic of the Congo	Breast cancer	-14.68	-34.37
Denmark	Breast cancer	-21.77	-48.88
Djibouti	Breast cancer	-0.33	-65.08
Dominica	Breast cancer	-0.04	-16.36
Dominican Republic	Breast cancer	-3.82	-46.93
Ecuador	Breast cancer	-9.5	-105.79
Egypt	Breast cancer	-42.42	-78.75
El Salvador	Breast cancer	-5.53	-123.95
Equatorial Guinea	Breast cancer	-0.97	-170.22
Eritrea	Breast cancer	-1.93	-40.36
Estonia	Breast cancer	-2.95	-40.19
Eswatini	Breast cancer	-0.13	-13.62
Ethiopia	Breast cancer	-44.7	-68.67
Fiji	Breast cancer	-0.01	-0.36

Location	Cause	Attributable number (Female thousands)	Attributable proportion (Female %)
Finland	Breast cancer	-12.3	-48.81
France	Breast cancer	-199.12	-60.63
Gabon	Breast cancer	-1.15	-57.26
Gambia	Breast cancer	-0.09	-24.16
Georgia	Breast cancer	-4.93	-15.92
Germany	Breast cancer	-256.32	-47.57
Ghana	Breast cancer	-12.47	-55.64
Greece	Breast cancer	-11.63	-20.69
Greenland	Breast cancer	-0.09	-45.87
Grenada	Breast cancer	-0.12	-43.02
Guam	Breast cancer	-0.04	-19.26
Guatemala	Breast cancer	-4.75	-118.38
Guinea	Breast cancer	-1.8	-25.55
Guinea-Bissau	Breast cancer	-0.43	-34.23
Guyana	Breast cancer	-0.47	-31.72
Haiti	Breast cancer	-4	-29.18
Honduras	Breast cancer	-2.08	-64.74
Hungary	Breast cancer	-25.49	-42.56
Iceland	Breast cancer	-0.49	-44.14
India	Breast cancer	-627.41	-75.96
Indonesia	Breast cancer	-158.82	-54.56
Iran (Islamic Republic of)	Breast cancer	-48.87	-136.46
Iraq	Breast cancer	-27.56	-145.63

Location	Cause	Attributable number (Female thousands)	Attributable proportion (Female %)
Ireland	Breast cancer	-12.89	-65.87
Israel	Breast cancer	-13.73	-62.36
Italy	Breast cancer	-144.55	-39.89
Jamaica	Breast cancer	-1.72	-32.87
Japan	Breast cancer	-53.87	-22.93
Jordan	Breast cancer	-8.23	-201.85
Kazakhstan	Breast cancer	-20.45	-38.16
Kenya	Breast cancer	-7.86	-43.87
Kiribati	Breast cancer	0	0.41
Kuwait	Breast cancer	-2.13	-165.43
Kyrgyzstan	Breast cancer	-3.83	-38.19
Lao People's Democratic Republic	Breast cancer	-3.7	-64.03
Latvia	Breast cancer	-3	-23.9
Lebanon	Breast cancer	-9.91	-144.58
Lesotho	Breast cancer	0.4	19.94
Liberia	Breast cancer	-1.28	-61.41
Libya	Breast cancer	-2.56	-90.65
Lithuania	Breast cancer	-3.24	-22.47
Luxembourg	Breast cancer	-1.5	-56.64
Madagascar	Breast cancer	-5.45	-35.13
Malawi	Breast cancer	-3.31	-35.75
Malaysia	Breast cancer	-39.05	-102.46
Maldives	Breast cancer	-0.25	-162.1

Location	Cause	Attributable number (Female thousands)	Attributable proportion (Female %)
Mali	Breast cancer	-4.16	-38.46
Malta	Breast cancer	-1.28	-56.02
Marshall Islands	Breast cancer	0	1.88
Mauritania	Breast cancer	-1.84	-76.51
Mauritius	Breast cancer	-0.97	-60.64
Mexico	Breast cancer	-89.68	-91.92
Micronesia (Federated States of)	Breast cancer	-0.04	-16.67
Monaco	Breast cancer	-0.1	-33.57
Mongolia	Breast cancer	-0.53	-46.77
Montenegro	Breast cancer	-0.6	-24.74
Morocco	Breast cancer	-11.66	-73.1
Mozambique	Breast cancer	-2.62	-17.47
Myanmar	Breast cancer	-57.15	-62.48
Namibia	Breast cancer	-1.32	-60.55
Nauru	Breast cancer	0	-11.09
Nepal	Breast cancer	-10.26	-71.16
Netherlands	Breast cancer	-53.17	-53.85
New Zealand	Breast cancer	-9.73	-49.5
Nicaragua	Breast cancer	-2.1	-103.06
Niger	Breast cancer	-1.28	-27.91
Nigeria	Breast cancer	-70.86	-55.42
Niue	Breast cancer	0	-10.67
North Macedonia	Breast cancer	-3.25	-42.02

Location	Cause	Attributable number (Female thousands)	Attributable proportion (Female %)
Northern Mariana Islands	Breast cancer	-0.01	-14.93
Norway	Breast cancer	-10.51	-49.42
Oman	Breast cancer	-0.53	-116.89
Pakistan	Breast cancer	-83.4	-43.64
Palau	Breast cancer	-0.01	-19.92
Palestine	Breast cancer	-3.36	-113.71
Panama	Breast cancer	-3.29	-111.77
Papua New Guinea	Breast cancer	0.28	4.24
Paraguay	Breast cancer	-3.16	-63.2
Peru	Breast cancer	-34.27	-123.23
Philippines	Breast cancer	-38.74	-34.39
Poland	Breast cancer	-75.05	-50.98
Portugal	Breast cancer	-30.3	-58.16
Puerto Rico	Breast cancer	-6.41	-56.93
Qatar	Breast cancer	-1.83	-364.28
Republic of Korea	Breast cancer	-50.75	-126.71
Republic of Moldova	Breast cancer	-6.06	-34.32
Romania	Breast cancer	-29.94	-35.78
Russian Federation	Breast cancer	-178.66	-32.95
Rwanda	Breast cancer	-7.96	-60.54
Saint Kitts and Nevis	Breast cancer	-0.13	-60.97
Saint Lucia	Breast cancer	-0.24	-57.78
Saint Vincent and the Grenadines	Breast cancer	-0.11	-32.38

Location	Cause	Attributable number (Female thousands)	Attributable proportion (Female %)
Samoa	Breast cancer	-0.02	-7.29
San Marino	Breast cancer	-0.04	-41.33
Sao Tome and Principe	Breast cancer	-0.07	-59.13
Saudi Arabia	Breast cancer	-18.25	-229.72
Senegal	Breast cancer	-2.91	-43.76
Serbia	Breast cancer	-22.58	-46.21
Seychelles	Breast cancer	-0.09	-62.96
Sierra Leone	Breast cancer	-0.97	-28.56
Singapore	Breast cancer	-7.37	-103.08
Slovakia	Breast cancer	-9.1	-44.48
Slovenia	Breast cancer	-4.49	-51.05
Solomon Islands	Breast cancer	0.03	8.96
Somalia	Breast cancer	-0.76	-10.63
South Africa	Breast cancer	-19.04	-26.7
South Sudan	Breast cancer	-1.14	-22.41
Spain	Breast cancer	-101.09	-53.12
Sri Lanka	Breast cancer	-19.21	-89.59
Sudan	Breast cancer	-7.62	-75.21
Suriname	Breast cancer	-0.25	-32.99
Sweden	Breast cancer	-14.68	-33.59
Switzerland	Breast cancer	-16.38	-44.21
Syrian Arab Republic	Breast cancer	-9.64	-99.66
Taiwan (Province of China)	Breast cancer	-30.68	-104.87

Location	Cause	Attributable number (Female thousands)	Attributable proportion (Female %)
Tajikistan	Breast cancer	-1.44	-19.06
Thailand	Breast cancer	-97.91	-112.87
Timor-Leste	Breast cancer	-0.24	-40.2
Togo	Breast cancer	-1.63	-46.79
Tokelau	Breast cancer	0	-22.98
Tonga	Breast cancer	-0.05	-14.61
Trinidad and Tobago	Breast cancer	-1.96	-53.54
Tunisia	Breast cancer	-8.2	-101.79
Turkey	Breast cancer	-74.75	-198.4
Turkmenistan	Breast cancer	-2.19	-40
Tuvalu	Breast cancer	-0.01	-18.4
Uganda	Breast cancer	-10.02	-52.29
Ukraine	Breast cancer	-33.47	-11.22
United Arab Emirates	Breast cancer	-3.83	-228.79
United Kingdom	Breast cancer	-195.29	-43.58
United Republic of Tanzania	Breast cancer	-13.56	-41.8
United States of America	Breast cancer	-454.63	-32.21
United States Virgin Islands	Breast cancer	-0.17	-36.28
Uruguay	Breast cancer	-8.1	-39.39
Uzbekistan	Breast cancer	-9.18	-29.97
Vanuatu	Breast cancer	0.04	23.77
Venezuela (Bolivarian Republic of)	Breast cancer	-26.62	-96.31
Viet Nam	Breast cancer	-62.56	-94.84

Location	Cause	Attributable number (Female thousands)	Attributable proportion (Female %)
Yemen	Breast cancer	-1.89	-40.79
Zambia	Breast cancer	-6.65	-70.32
Zimbabwe	Breast cancer	1.67	14.6

Table S14. Absolute (the number) and relative contribution (the proportion) associated with disease severity changes for liver cancer (LivC), by sex at global, SDI regional, GBD regional level between 1990 and 2021.

Location	Cause	Attributable number (Both thousands)	Attributable proportion (Both %)	Attributable number (Male thousands)	Attributable proportion (Male %)	Attributable number (Female thousands)	Attributable proportion (Female %)
Global	Liver cancer	-2561.62	-36.45	-1915.22	-37.87	-633.95	-32.2
High SDI	Liver cancer	-905.55	-61.15	-688.53	-62.53	-211.21	-55.64
High-middle SDI	Liver cancer	-731.74	-39.94	-564.09	-41.31	-167.89	-35.98
Middle SDI	Liver cancer	-901.8	-35.87	-660.82	-36.26	-239.39	-34.6
Low-middle SDI	Liver cancer	-41.04	-5.64	-26.7	-5.71	-14.42	-5.54
Low SDI	Liver cancer	-13.15	-2.81	-7.72	-2.59	-5.6	-3.31
High-income Asia Pacific	Liver cancer	-521.38	-59.96	-395.23	-58.16	-128.71	-67.75
High-income North America	Liver cancer	-119.66	-71.88	-87.91	-81.11	-28.21	-48.56
Western Europe	Liver cancer	-327.97	-70.53	-250.37	-78.14	-75.52	-52.22
Australasia	Liver cancer	-15.22	-136.93	-13.04	-159.78	-2.05	-69.2
Eastern Europe	Liver cancer	-8.57	-6.46	-5.47	-6.83	-3.16	-6.01
Central Europe	Liver cancer	-11.51	-9.69	-7.73	-10.54	-3.73	-8.2
Southern Latin America	Liver cancer	-2.48	-25.22	-1.71	-26.92	-0.78	-22.35
East Asia	Liver cancer	-1486.34	-46.23	-1113.83	-45.74	-373.05	-47.83
Central Asia	Liver cancer	-2.1	-1.98	-1.44	-2.12	-0.69	-1.79
North Africa and Middle East	Liver cancer	-30.58	-12.97	-19.28	-13.91	-11.06	-11.38
Andean Latin America	Liver cancer	-1.58	-10.39	-0.78	-10.75	-0.8	-10.01
Southeast Asia	Liver cancer	-95.82	-16.47	-63.3	-14.9	-32.85	-20.94

Location	Cause	Attributable number (Both thousands)	Attributable proportion (Both %)	Attributable number (Male thousands)	Attributable proportion (Male %)	Attributable number (Female thousands)	Attributable proportion (Female %)
Tropical Latin America	Liver cancer	-4.05	-9.29	-2.54	-10.35	-1.51	-7.89
Southern Sub-Saharan Africa	Liver cancer	0.29	0.56	0.03	0.11	0.25	1.11
Caribbean	Liver cancer	-1.05	-7.55	-0.69	-9.2	-0.34	-5.33
Central Latin America	Liver cancer	-5.67	-8.85	-2.94	-9.47	-2.69	-8.13
South Asia	Liver cancer	-27.35	-7.09	-19.82	-7.22	-7.9	-7.1
Central Sub-Saharan Africa	Liver cancer	-1.69	-3.17	-1.19	-3.62	-0.49	-2.39
Oceania	Liver cancer	-0.22	-4.87	-0.13	-4.32	-0.08	-6.04
Western Sub-Saharan Africa	Liver cancer	-7.87	-2.29	-5.19	-2.23	-2.94	-2.64
Eastern Sub-Saharan Africa	Liver cancer	-4.52	-3.24	-1.94	-2.62	-2.5	-3.82
Afghanistan	Liver cancer	-0.39	-3.21	-0.19	-3.52	-0.23	-3.39
Albania	Liver cancer	-0.55	-8.58	-0.39	-9.56	-0.16	-6.94
Algeria	Liver cancer	-0.8	-16.96	-0.46	-17.35	-0.33	-16.15
American Samoa	Liver cancer	0	-8.47	0	-7.02	0	-10.98
Andorra	Liver cancer	-0.05	-33.09	-0.03	-35.36	-0.02	-30.25
Angola	Liver cancer	-0.73	-4	-0.58	-4.21	-0.15	-3.31
Antigua and Barbuda	Liver cancer	0	-5.97	0	-6.74	0	-4.5
Argentina	Liver cancer	-0.61	-12.62	-0.45	-14.33	-0.17	-9.86
Armenia	Liver cancer	-0.26	-4.69	-0.15	-5.1	-0.11	-4.09
Australia	Liver cancer	-13.45	-148.58	-11.7	-174.89	-1.57	-66.54
Austria	Liver cancer	-6.41	-85.6	-4.72	-91.03	-1.64	-71.13

Location	Cause	Attributable number (Both thousands)	Attributable proportion (Both %)	Attributable number (Male thousands)	Attributable proportion (Male %)	Attributable number (Female thousands)	Attributable proportion (Female %)
Azerbaijan	Liver cancer	-0.29	-3.07	-0.21	-3.93	-0.08	-2.07
Bahamas	Liver cancer	-0.01	-5	-0.01	-5.86	0	-3.69
Bahrain	Liver cancer	-0.11	-30.39	-0.08	-32.57	-0.03	-26.13
Bangladesh	Liver cancer	-2.89	-9	-2.1	-8.72	-0.8	-9.97
Barbados	Liver cancer	-0.01	-6.61	-0.01	-7.43	0	-5.62
Belarus	Liver cancer	-0.61	-7.08	-0.38	-7.22	-0.2	-6.08
Belgium	Liver cancer	-3.53	-36.22	-2.46	-45.3	-1.16	-26.98
Belize	Liver cancer	0	-4.73	0	-5.31	0	-4.34
Benin	Liver cancer	-0.3	-2.29	-0.21	-2.23	-0.09	-2.37
Bermuda	Liver cancer	-0.01	-22.28	-0.01	-24.32	0	-19.46
Bhutan	Liver cancer	-0.02	-7.07	-0.01	-6.79	-0.01	-8.38
Bolivia (Plurinational State of)	Liver cancer	-0.16	-5.41	-0.07	-5.76	-0.09	-5.08
Bosnia and Herzegovina	Liver cancer	-0.52	-7.15	-0.35	-7.29	-0.17	-6.83
Botswana	Liver cancer	-0.01	-0.95	-0.01	-1.27	0	-0.98
Brazil	Liver cancer	-3.97	-9.36	-2.49	-10.41	-1.47	-7.96
Brunei Darussalam	Liver cancer	-0.1	-22.47	-0.07	-20.89	-0.03	-26.2
Bulgaria	Liver cancer	-0.76	-3.17	-0.49	-3.27	-0.26	-2.85
Burkina Faso	Liver cancer	-0.8	-2.14	-0.62	-2.16	-0.19	-2.22
Burundi	Liver cancer	-0.13	-3.44	-0.06	-4.23	-0.07	-2.93
Cabo Verde	Liver cancer	-0.04	-6.26	-0.02	-7.31	-0.02	-5.19

Location	Cause	Attributable number (Both thousands)	Attributable proportion (Both %)	Attributable number (Male thousands)	Attributable proportion (Male %)	Attributable number (Female thousands)	Attributable proportion (Female %)
Cambodia	Liver cancer	-1.1	-8.97	-0.46	-7.25	-0.65	-10.98
Cameroon	Liver cancer	-0.54	-1.92	-0.33	-1.75	-0.19	-2.08
Canada	Liver cancer	-11.76	-75.7	-9.06	-86.34	-2.75	-54.39
Central African Republic	Liver cancer	-0.06	-1.23	-0.05	-1.49	0	-0.21
Chad	Liver cancer	-0.14	-0.96	-0.11	-1.01	-0.04	-1.06
Chile	Liver cancer	-1.64	-39.68	-1.08	-42.17	-0.56	-35.54
China	Liver cancer	-1440.06	-46.25	-1086.61	-46.08	-353.63	-46.8
Colombia	Liver cancer	-1.75	-13.61	-0.85	-14.06	-0.88	-12.92
Comoros	Liver cancer	-0.01	-3.26	0	-3.44	-0.01	-3.16
Congo	Liver cancer	-0.2	-4.34	-0.14	-5.23	-0.06	-3.3
Cook Islands	Liver cancer	-0.01	-24.64	-0.01	-22.59	0	-30.58
Costa Rica	Liver cancer	-0.32	-15.1	-0.19	-16	-0.13	-13.86
Coted'Ivoire	Liver cancer	-0.28	-2.79	-0.22	-2.84	-0.06	-2.82
Croatia	Liver cancer	-1.17	-29.88	-0.79	-34.6	-0.36	-21.99
Cuba	Liver cancer	-0.49	-8.94	-0.28	-9.64	-0.21	-8.21
Cyprus	Liver cancer	-0.35	-59.85	-0.25	-62.16	-0.11	-55.78
Czechia	Liver cancer	-2.02	-12.43	-1.43	-13.64	-0.59	-10.25
Democratic People's Republic of Korea	Liver cancer	-5.44	-9.15	-3.07	-7.15	-2.3	-13.98
Democratic Republic of the Congo	Liver cancer	-0.55	-2.35	-0.29	-2.53	-0.25	-2.06
Denmark	Liver cancer	0.28	10.56	0.02	1.19	0.24	23.52

Location	Cause	Attributable number (Both thousands)	Attributable proportion (Both %)	Attributable number (Male thousands)	Attributable proportion (Male %)	Attributable number (Female thousands)	Attributable proportion (Female %)
Djibouti	Liver cancer	-0.01	-4.32	0	-4.96	0	-3.46
Dominica	Liver cancer	0	-2.32	0	-2.59	0	-1.73
Dominican Republic	Liver cancer	-0.06	-4.1	-0.03	-3.8	-0.03	-4.27
Ecuador	Liver cancer	-0.38	-6.63	-0.18	-6.46	-0.2	-6.76
Egypt	Liver cancer	-9.96	-8.67	-6.33	-9.69	-3.41	-6.89
El Salvador	Liver cancer	-0.11	-7.64	-0.05	-7.7	-0.06	-7.56
Equatorial Guinea	Liver cancer	-0.03	-13.55	-0.02	-22.39	-0.01	-8.49
Eritrea	Liver cancer	-0.1	-6	-0.05	-6.54	-0.05	-5.45
Estonia	Liver cancer	-0.23	-13.37	-0.14	-13.62	-0.08	-12.12
Eswatini	Liver cancer	0.03	2.95	0.02	3.63	0	0.79
Ethiopia	Liver cancer	-1.63	-6.84	-0.59	-7.56	-1.03	-6.44
Fiji	Liver cancer	-0.03	-5.26	-0.02	-4.54	-0.01	-6.67
Finland	Liver cancer	-4.11	-84.29	-2.92	-103.91	-1.34	-64.88
France	Liver cancer	-73.07	-79.5	-60.94	-81.99	-11.7	-66.56
Gabon	Liver cancer	-0.07	-3.85	-0.05	-4.54	-0.02	-2.68
Gambia	Liver cancer	-0.09	-2.3	-0.07	-2.34	-0.02	-2.5
Georgia	Liver cancer	-0.13	-1.65	-0.09	-1.67	-0.05	-1.62
Germany	Liver cancer	-76.02	-95.72	-52.45	-104.76	-21.91	-74.65
Ghana	Liver cancer	-0.76	-2.86	-0.47	-2.54	-0.27	-3.24
Greece	Liver cancer	-1.63	-23.29	-1.32	-32.06	-0.41	-14.04

Location	Cause	Attributable number (Both thousands)	Attributable proportion (Both %)	Attributable number (Male thousands)	Attributable proportion (Male %)	Attributable number (Female thousands)	Attributable proportion (Female %)
Greenland	Liver cancer	-0.01	-11.55	-0.01	-11.85	0	-10.74
Grenada	Liver cancer	0	-5.03	0	-6.15	0	-4.23
Guam	Liver cancer	-0.01	-15.92	-0.01	-15.55	0	-21.01
Guatemala	Liver cancer	-0.42	-6.11	-0.22	-6.3	-0.21	-5.87
Guinea	Liver cancer	-0.23	-0.92	-0.08	-0.52	-0.15	-1.65
Guinea-Bissau	Liver cancer	-0.08	-1.76	-0.07	-1.72	-0.02	-1.67
Guyana	Liver cancer	-0.01	-2.81	-0.01	-3.1	0	-2.53
Haiti	Liver cancer	-0.04	-2.41	-0.02	-2.4	-0.02	-2.5
Honduras	Liver cancer	-0.03	-2.3	-0.01	-2.27	-0.02	-2.3
Hungary	Liver cancer	-0.91	-8.57	-0.6	-9.4	-0.31	-7.39
Iceland	Liver cancer	-0.1	-63.36	-0.08	-74.08	-0.02	-45.15
India	Liver cancer	-23.35	-7.86	-17.03	-7.85	-6.61	-8.26
Indonesia	Liver cancer	-9.34	-8.75	-4.63	-7.09	-4.74	-11.42
Iran (Islamic Republic of)	Liver cancer	-3.53	-23.45	-1.99	-24.57	-1.56	-22.47
Iraq	Liver cancer	-1.31	-13.94	-0.74	-13.62	-0.56	-14.18
Ireland	Liver cancer	-1.39	-80.56	-0.9	-93.11	-0.51	-66.55
Israel	Liver cancer	-1	-41.82	-0.66	-47.7	-0.35	-34.52
Italy	Liver cancer	-63.6	-47.45	-47.34	-50.41	-16.57	-41.32
Jamaica	Liver cancer	-0.02	-4.53	-0.01	-5.17	-0.01	-3.93
Japan	Liver cancer	-257.53	-48.74	-196.98	-47.45	-62.48	-55.14

Location	Cause	Attributable number (Both thousands)	Attributable proportion (Both %)	Attributable number (Male thousands)	Attributable proportion (Male %)	Attributable number (Female thousands)	Attributable proportion (Female %)
Jordan	Liver cancer	-0.22	-26.68	-0.13	-26.75	-0.09	-26.58
Kazakhstan	Liver cancer	-1.21	-3.01	-0.87	-3.09	-0.36	-3
Kenya	Liver cancer	-0.23	-3.37	-0.08	-2.71	-0.13	-3.55
Kiribati	Liver cancer	0	-3.06	0	-2.49	0	-3.48
Kuwait	Liver cancer	-0.21	-26.26	-0.15	-23.86	-0.05	-30.3
Kyrgyzstan	Liver cancer	-0.16	-2.79	-0.11	-3.04	-0.05	-2.32
Lao People's Democratic Republic	Liver cancer	-0.42	-6.15	-0.28	-5.69	-0.14	-7.5
Latvia	Liver cancer	-0.22	-8.82	-0.17	-10.69	-0.05	-5.24
Lebanon	Liver cancer	-0.43	-22.06	-0.26	-23.03	-0.17	-21.11
Lesotho	Liver cancer	0.13	8.21	0.09	10.14	0.03	4.82
Liberia	Liver cancer	-0.27	-3.23	-0.21	-3.15	-0.07	-3.8
Libya	Liver cancer	-0.39	-12.93	-0.22	-13.9	-0.16	-11.8
Lithuania	Liver cancer	-0.52	-20.78	-0.37	-24.96	-0.05	-5.23
Luxembourg	Liver cancer	-0.22	-52.37	-0.15	-58.15	-0.07	-43.9
Madagascar	Liver cancer	-0.08	-1.43	-0.03	-1.38	-0.05	-1.6
Malawi	Liver cancer	-0.16	-3.55	-0.09	-3.41	-0.07	-3.43
Malaysia	Liver cancer	-2.89	-24.14	-1.88	-21.93	-1.06	-31.1
Maldives	Liver cancer	-0.05	-28.9	-0.05	-29.24	-0.01	-40.32
Mali	Liver cancer	-1.04	-2.89	-0.7	-2.71	-0.32	-3.12
Malta	Liver cancer	-0.09	-49.04	-0.07	-57.07	-0.02	-36.21

Location	Cause	Attributable number (Both thousands)	Attributable proportion (Both %)	Attributable number (Male thousands)	Attributable proportion (Male %)	Attributable number (Female thousands)	Attributable proportion (Female %)
Marshall Islands	Liver cancer	0	-4.02	0	-4.09	0	-4.13
Mauritania	Liver cancer	-0.51	-4.06	-0.4	-4.1	-0.12	-4.44
Mauritius	Liver cancer	-0.08	-9.11	-0.04	-8.12	-0.04	-11.42
Mexico	Liver cancer	-2.16	-9.41	-1.11	-10.18	-1.04	-8.6
Micronesia (Federated States of)	Liver cancer	0	-4.08	0	-3.28	0	-5.57
Monaco	Liver cancer	-0.02	-34.12	-0.01	-40.59	0	-22.53
Mongolia	Liver cancer	-0.84	-4.57	-0.42	-3.54	-0.38	-5.65
Montenegro	Liver cancer	-0.05	-6.06	-0.03	-5.9	-0.02	-6.18
Morocco	Liver cancer	-0.12	-6.99	-0.08	-6.97	-0.04	-6.93
Mozambique	Liver cancer	0.19	0.62	0.27	1.47	-0.07	-0.63
Myanmar	Liver cancer	-2.36	-8.97	-1.19	-7.86	-1.19	-10.56
Namibia	Liver cancer	-0.01	-3.4	-0.01	-4.38	-0.01	-2.91
Nauru	Liver cancer	0	-3.03	0	-2.36	0	-4.78
Nepal	Liver cancer	-0.42	-8.62	-0.27	-8.45	-0.14	-8.9
Netherlands	Liver cancer	-1.1	-18.84	-0.94	-25.45	-0.17	-8.21
New Zealand	Liver cancer	-2.37	-115.09	-1.72	-116.75	-0.66	-111.77
Nicaragua	Liver cancer	-0.1	-7.84	-0.05	-8.15	-0.05	-7.55
Niger	Liver cancer	-0.31	-1.68	-0.26	-1.79	-0.1	-2.35
Nigeria	Liver cancer	-1.84	-2.52	-0.91	-2.58	-1.12	-2.97
Niue	Liver cancer	0	-6.45	0	-6.01	0	-7.94

Location	Cause	Attributable number (Both thousands)	Attributable proportion (Both %)	Attributable number (Male thousands)	Attributable proportion (Male %)	Attributable number (Female thousands)	Attributable proportion (Female %)
North Macedonia	Liver cancer	-0.3	-6.49	-0.19	-6.93	-0.11	-5.54
Northern Mariana Islands	Liver cancer	-0.01	-16.65	0	-13.42	0	-23.2
Norway	Liver cancer	-1.84	-78.91	-1.23	-89.33	-0.55	-57.51
Oman	Liver cancer	-0.21	-31.43	-0.16	-33.65	-0.04	-25.62
Pakistan	Liver cancer	-0.85	-1.65	-0.52	-1.72	-0.35	-1.64
Palau	Liver cancer	0	-9.86	0	-9.87	0	-12.89
Palestine	Liver cancer	-0.19	-12.01	-0.11	-12.99	-0.08	-10.62
Panama	Liver cancer	-0.14	-10.41	-0.08	-10.64	-0.07	-10.29
Papua New Guinea	Liver cancer	-0.08	-3.39	-0.04	-2.75	-0.04	-4.65
Paraguay	Liver cancer	-0.08	-6.52	-0.04	-7.23	-0.03	-5.67
Peru	Liver cancer	-1	-15.45	-0.51	-15.99	-0.49	-14.91
Philippines	Liver cancer	-2.94	-3.84	-1.89	-3.07	-0.96	-6.47
Poland	Liver cancer	-0.94	-14.83	-0.61	-21.14	-0.38	-10.9
Portugal	Liver cancer	-2.63	-38.46	-1.97	-43.27	-0.71	-30.96
Puerto Rico	Liver cancer	-0.47	-18.02	-0.33	-21.15	-0.14	-13.4
Qatar	Liver cancer	-0.41	-112.04	-0.33	-122.37	-0.09	-87.13
Republic of Korea	Liver cancer	-335.79	-100.14	-256.87	-98.8	-81.7	-108.45
Republic of Moldova	Liver cancer	-0.16	-4.3	-0.1	-4.18	-0.06	-4.45
Romania	Liver cancer	-1.29	-13.24	-0.87	-13.45	-0.41	-12.38
Russian Federation	Liver cancer	-6.23	-7.73	-3.85	-7.88	-2.53	-7.95

Location	Cause	Attributable number (Both thousands)	Attributable proportion (Both %)	Attributable number (Male thousands)	Attributable proportion (Male %)	Attributable number (Female thousands)	Attributable proportion (Female %)
Rwanda	Liver cancer	-0.32	-5.19	-0.14	-5.25	-0.18	-5.12
Saint Kitts and Nevis	Liver cancer	0	-6.53	0	-7.34	0	-5.64
Saint Lucia	Liver cancer	0	-6.74	0	-7.76	0	-5.55
Saint Vincent and the Grenadines	Liver cancer	0	-3.12	0	-3.37	0	-2.67
Samoa	Liver cancer	-0.01	-6.28	-0.01	-5.39	0	-9.09
San Marino	Liver cancer	0	-31.59	0	-34.6	0	-23.66
Sao Tome and Principe	Liver cancer	0	-3.46	0	-3.49	0	-3.59
Saudi Arabia	Liver cancer	-2.44	-19.88	-1.74	-18.63	-0.68	-23.3
Senegal	Liver cancer	-0.37	-2.44	-0.28	-2.51	-0.1	-2.66
Serbia	Liver cancer	-1.52	-9.72	-0.98	-10.26	-0.53	-8.74
Seychelles	Liver cancer	-0.01	-9.3	-0.01	-9.47	0	-10.79
Sierra Leone	Liver cancer	-0.19	-1.57	-0.18	-1.82	-0.03	-1.23
Singapore	Liver cancer	-7.02	-131.23	-5.16	-124.76	-1.88	-154.3
Slovakia	Liver cancer	-0.82	-9.64	-0.57	-10.93	-0.23	-7.18
Slovenia	Liver cancer	-0.72	-26.46	-0.47	-27.11	-0.26	-26.53
Solomon Islands	Liver cancer	-0.01	-4.73	-0.01	-4.1	0	-6.83
Somalia	Liver cancer	-0.17	-2.16	-0.1	-2.19	-0.07	-2.07
South Africa	Liver cancer	-0.06	-0.19	-0.06	-0.36	0	-0.03
South Sudan	Liver cancer	-0.05	-1.19	-0.03	-1.48	-0.02	-0.97
Spain	Liver cancer	-34.99	-68.11	-27.57	-78.5	-6.82	-41.97

Location	Cause	Attributable number (Both thousands)	Attributable proportion (Both %)	Attributable number (Male thousands)	Attributable proportion (Male %)	Attributable number (Female thousands)	Attributable proportion (Female %)
Sri Lanka	Liver cancer	-1.3	-21.56	-0.82	-19.21	-0.5	-28.47
Sudan	Liver cancer	-0.55	-6.17	-0.34	-6.45	-0.21	-5.69
Suriname	Liver cancer	-0.01	-3.61	0	-3.71	0	-3.24
Sweden	Liver cancer	-1.54	-18.65	-0.9	-19.06	-0.69	-19.62
Switzerland	Liver cancer	-4.48	-47.25	-3.76	-51.21	-0.7	-32.8
Syrian Arab Republic	Liver cancer	-1.21	-12.03	-0.68	-12.08	-0.52	-11.89
Taiwan (Province of China)	Liver cancer	-32.54	-77.47	-19.73	-58.09	-11.5	-142.99
Tajikistan	Liver cancer	0	0.05	-0.01	-0.4	0.01	0.4
Thailand	Liver cancer	-41.23	-25.02	-27.7	-22.63	-14.3	-33.71
Timor-Leste	Liver cancer	-0.02	-5.29	-0.01	-3.32	-0.01	-8.18
Togo	Liver cancer	-0.1	-2.35	-0.04	-1.76	-0.05	-3.13
Tokelau	Liver cancer	0	-8.59	0	-8.07	0	-9.24
Tonga	Liver cancer	-0.02	-5.3	-0.01	-4.64	-0.01	-7.65
Trinidad and Tobago	Liver cancer	-0.03	-6.51	-0.02	-7.04	-0.01	-5.87
Tunisia	Liver cancer	-0.38	-17.16	-0.25	-18.24	-0.13	-15.35
Turkey	Liver cancer	-6.28	-21.77	-4.43	-22.74	-1.87	-19.91
Turkmenistan	Liver cancer	-0.06	-2.05	-0.05	-2.18	-0.02	-2.34
Tuvalu	Liver cancer	0	-6.51	0	-6.2	0	-7.27
Uganda	Liver cancer	-0.87	-5.4	-0.57	-5.06	-0.29	-5.74
Ukraine	Liver cancer	-1.29	-3.88	-0.85	-4.33	-0.43	-3.13

Location	Cause	Attributable number (Both thousands)	Attributable proportion (Both %)	Attributable number (Male thousands)	Attributable proportion (Male %)	Attributable number (Female thousands)	Attributable proportion (Female %)
United Arab Emirates	Liver cancer	-0.51	-38.25	-0.38	-42.72	-0.12	-26.97
United Kingdom	Liver cancer	-40.5	-107.32	-28.45	-126.48	-9.98	-65.47
United Republic of Tanzania	Liver cancer	-0.56	-2.8	-0.33	-2.94	-0.24	-2.75
United States of America	Liver cancer	-108.12	-71.66	-79.12	-80.87	-25.31	-47.72
United States Virgin Islands	Liver cancer	0	-5.38	0	-6.25	0	-4.96
Uruguay	Liver cancer	-0.15	-16.63	-0.13	-18.85	-0.03	-11.77
Uzbekistan	Liver cancer	0.04	0.32	0.02	0.32	0.02	0.34
Vanuatu	Liver cancer	0	-3.64	0	-2.97	0	-5.16
Venezuela (Bolivarian Republic of)	Liver cancer	-0.73	-5.21	-0.38	-5.42	-0.35	-5.03
Viet Nam	Liver cancer	-35.05	-20.88	-24.59	-18.26	-10.38	-31.27
Yemen	Liver cancer	-0.19	-4.15	-0.12	-4.26	-0.07	-4.45
Zambia	Liver cancer	-0.17	-1.91	-0.07	-1.42	-0.09	-2.41
Zimbabwe	Liver cancer	0.33	2.29	0.13	1.53	0.2	3.32

Table S15. Absolute (the number) and relative contribution (the proportion) associated with disease severity changes for pancreatic cancer (PanC), by sex at global, SDI regional, GBD regional level between 1990 and 2021.

Location	Cause	Attributable number (Both thousands)	Attributable proportion (Both %)	Attributable number (Male thousands)	Attributable proportion (Male %)	Attributable number (Female thousands)	Attributable proportion (Female %)
Global	Pancreatic cancer	-945.76	-18.19	-485.55	-16.46	-465.58	-20.7
High SDI	Pancreatic cancer	-686.31	-31.96	-333.9	-28.75	-354	-35.91
High-middle SDI	Pancreatic cancer	-243.9	-13.7	-137.01	-13.11	-108.76	-14.79
Middle SDI	Pancreatic cancer	-123.06	-13.09	-71.04	-12.89	-52.36	-13.46
Low-middle SDI	Pancreatic cancer	-17.86	-7.42	-9.69	-6.88	-8.15	-8.16
Low SDI	Pancreatic cancer	-3.6	-4.28	-1.87	-3.9	-1.71	-4.74
High-income Asia Pacific	Pancreatic cancer	-131.18	-32.25	-66.46	-27.67	-65.59	-39.37
High-income North America	Pancreatic cancer	-135.14	-19.53	-60.88	-16.83	-74.78	-22.63
Western Europe	Pancreatic cancer	-395.75	-37.05	-197.18	-34.88	-200.06	-39.79
Australasia	Pancreatic cancer	-16.56	-43	-8.87	-41.6	-7.75	-45.13
Eastern Europe	Pancreatic cancer	-30.18	-5.47	-16.47	-5.25	-14.02	-5.87
Central Europe	Pancreatic cancer	-26.63	-8.33	-14.14	-7.55	-12.56	-9.5
Southern Latin America	Pancreatic cancer	-8.5	-8.37	-4.2	-7.92	-4.35	-8.96
East Asia	Pancreatic cancer	-228.37	-19.85	-140.81	-19.78	-88.06	-20.07
Central Asia	Pancreatic cancer	-1.34	-3.3	-0.75	-3.36	-0.64	-3.5
North Africa and Middle East	Pancreatic cancer	-23.64	-17.83	-14.44	-17.64	-9.19	-18.12
Andean Latin America	Pancreatic cancer	-2.65	-11.4	-1.15	-10.43	-1.51	-12.29
Southeast Asia	Pancreatic cancer	-19.2	-11.69	-9.77	-10.79	-9.44	-12.79
Tropical Latin America	Pancreatic cancer	-10.54	-9.18	-5.3	-8.42	-5.22	-10.06

Location	Cause	Attributable number (Both thousands)	Attributable proportion (Both %)	Attributable number (Male thousands)	Attributable proportion (Male %)	Attributable number (Female thousands)	Attributable proportion (Female %)
Southern Sub-Saharan Africa	Pancreatic cancer	-0.4	-1.41	-0.21	-1.35	-0.19	-1.5
Caribbean	Pancreatic cancer	-1.81	-6.17	-0.91	-5.72	-0.89	-6.65
Central Latin America	Pancreatic cancer	-8.54	-8.96	-3.76	-8.11	-4.76	-9.73
South Asia	Pancreatic cancer	-12.73	-7.59	-7.61	-7	-5.14	-8.7
Central Sub-Saharan Africa	Pancreatic cancer	-0.53	-3.78	-0.32	-3.81	-0.21	-3.59
Oceania	Pancreatic cancer	-0.06	-4.22	-0.03	-3.45	-0.03	-5.53
Western Sub-Saharan Africa	Pancreatic cancer	-1.06	-4.48	-0.47	-3.55	-0.59	-5.73
Eastern Sub-Saharan Africa	Pancreatic cancer	-1.69	-4.9	-0.8	-4.48	-0.87	-5.22
Afghanistan	Pancreatic cancer	-0.13	-3.44	-0.05	-3.15	-0.08	-3.89
Albania	Pancreatic cancer	-0.21	-10.32	-0.14	-9.75	-0.08	-11.53
Algeria	Pancreatic cancer	-0.55	-15.07	-0.32	-14.14	-0.23	-16.64
American Samoa	Pancreatic cancer	0	-4.96	0	-4.43	0	-6.41
Andorra	Pancreatic cancer	-0.03	-13.59	-0.02	-12.26	-0.01	-16.5
Angola	Pancreatic cancer	-0.16	-6.28	-0.1	-6.04	-0.06	-6.5
Antigua and Barbuda	Pancreatic cancer	0	-5.55	0	-5.51	0	-5.69
Argentina	Pancreatic cancer	-4.8	-6.1	-2.47	-5.86	-2.37	-6.49
Armenia	Pancreatic cancer	-0.27	-5.12	-0.15	-5.01	-0.13	-5.46
Australia	Pancreatic cancer	-14.49	-44.94	-7.82	-44.14	-6.81	-46.85
Austria	Pancreatic cancer	-6.77	-26.98	-3.01	-24.28	-3.92	-30.91
Azerbaijan	Pancreatic cancer	-0.16	-4.04	-0.1	-4.06	-0.06	-4.25
Bahamas	Pancreatic cancer	-0.01	-5.23	-0.01	-5.27	0	-5.37

Location	Cause	Attributable number (Both thousands)	Attributable proportion (Both %)	Attributable number (Male thousands)	Attributable proportion (Male %)	Attributable number (Female thousands)	Attributable proportion (Female %)
Bahrain	Pancreatic cancer	-0.08	-28.81	-0.05	-29.37	-0.03	-27.93
Bangladesh	Pancreatic cancer	-1.29	-9.47	-0.8	-8.49	-0.48	-11.63
Barbados	Pancreatic cancer	-0.02	-5.23	-0.01	-5.18	-0.01	-5.31
Belarus	Pancreatic cancer	-2.15	-11.42	-0.84	-7.46	-1.29	-17.03
Belgium	Pancreatic cancer	-4.08	-13.88	-2.08	-13.46	-2.05	-14.75
Belize	Pancreatic cancer	0	-5.85	0	-5.35	0	-6.42
Benin	Pancreatic cancer	-0.04	-4.76	-0.02	-3.72	-0.02	-5.92
Bermuda	Pancreatic cancer	-0.03	-16.35	-0.01	-16.69	-0.01	-16.74
Bhutan	Pancreatic cancer	-0.01	-9.41	0	-8.43	0	-11.12
Bolivia (Plurinational State of)	Pancreatic cancer	-0.3	-6.64	-0.12	-6.14	-0.18	-7.05
Bosnia and Herzegovina	Pancreatic cancer	-0.49	-6.61	-0.27	-6.25	-0.23	-7.4
Botswana	Pancreatic cancer	-0.01	-2.58	-0.01	-2.02	-0.01	-3.08
Brazil	Pancreatic cancer	-10.42	-9.18	-5.24	-8.42	-5.16	-10.07
Brunei Darussalam	Pancreatic cancer	-0.02	-16.12	-0.01	-14.96	-0.01	-17.13
Bulgaria	Pancreatic cancer	-1.38	-6	-0.75	-5.16	-0.62	-7.38
Burkina Faso	Pancreatic cancer	-0.06	-4.22	-0.03	-3.32	-0.03	-5.28
Burundi	Pancreatic cancer	-0.05	-3.26	-0.03	-3.76	-0.02	-2.77
Cabo Verde	Pancreatic cancer	-0.01	-39.43	-0.01	-30.44	-0.01	-45.08
Cambodia	Pancreatic cancer	-0.32	-10.12	-0.15	-9.09	-0.17	-11.21
Cameroon	Pancreatic cancer	-0.1	-4.03	-0.05	-3.26	-0.05	-4.95
Canada	Pancreatic cancer	-26.11	-41.49	-10.94	-32.43	-14.79	-50.62

Location	Cause	Attributable number (Both thousands)	Attributable proportion (Both %)	Attributable number (Male thousands)	Attributable proportion (Male %)	Attributable number (Female thousands)	Attributable proportion (Female %)
Central African Republic	Pancreatic cancer	-0.01	-1.45	-0.01	-1.6	0	-1.1
Chad	Pancreatic cancer	-0.01	-2.01	-0.01	-1.73	-0.01	-2.26
Chile	Pancreatic cancer	-2.62	-19.4	-1.18	-19.55	-1.46	-19.57
China	Pancreatic cancer	-219.76	-19.7	-136.47	-19.77	-83.7	-19.68
Colombia	Pancreatic cancer	-2.89	-12.14	-1.28	-10.94	-1.6	-13.27
Comoros	Pancreatic cancer	0	-3.47	0	-3.18	0	-3.66
Congo	Pancreatic cancer	-0.06	-5.89	-0.04	-6.57	-0.02	-5.04
Cook Islands	Pancreatic cancer	0	-11.97	0	-11.33	0	-14.9
Costa Rica	Pancreatic cancer	-0.22	-12.52	-0.1	-10.91	-0.12	-14.14
Coted'Ivoire	Pancreatic cancer	-0.07	-4.55	-0.04	-4.29	-0.03	-4.74
Croatia	Pancreatic cancer	-3.11	-24.95	-1.49	-21.07	-1.67	-30.84
Cuba	Pancreatic cancer	-0.94	-6.89	-0.47	-6	-0.46	-7.93
Cyprus	Pancreatic cancer	-0.32	-31.63	-0.17	-29.89	-0.14	-34.45
Czechia	Pancreatic cancer	-6.6	-16.83	-3.3	-14.74	-3.33	-19.82
Democratic People's Republic of Korea	Pancreatic cancer	-1.13	-6.8	-0.6	-6.43	-0.55	-7.57
Democratic Republic of the Congo	Pancreatic cancer	-0.25	-2.75	-0.14	-2.66	-0.1	-2.66
Denmark	Pancreatic cancer	-2.91	-23.8	-1.63	-28.31	-1.33	-20.5
Djibouti	Pancreatic cancer	-0.01	-5.82	0	-5.84	0	-5.78
Dominica	Pancreatic cancer	0	-2.02	0	-2.18	0	-1.98
Dominican Republic	Pancreatic cancer	-0.19	-6.3	-0.09	-5.38	-0.11	-7.18

Location	Cause	Attributable number (Both thousands)	Attributable proportion (Both %)	Attributable number (Male thousands)	Attributable proportion (Male %)	Attributable number (Female thousands)	Attributable proportion (Female %)
Ecuador	Pancreatic cancer	-0.45	-9.99	-0.19	-9.26	-0.26	-10.67
Egypt	Pancreatic cancer	-2.3	-13.92	-1.42	-12.8	-0.87	-15.98
El Salvador	Pancreatic cancer	-0.24	-12.27	-0.1	-10.78	-0.14	-13.47
Equatorial Guinea	Pancreatic cancer	-0.02	-15.79	-0.01	-16.27	-0.01	-15.56
Eritrea	Pancreatic cancer	-0.04	-5.94	-0.02	-6.4	-0.02	-5.38
Estonia	Pancreatic cancer	-1.02	-23.45	-0.52	-22.4	-0.51	-24.98
Eswatini	Pancreatic cancer	0	0.04	0	0.51	0	-0.37
Ethiopia	Pancreatic cancer	-0.46	-7.7	-0.22	-7.44	-0.23	-7.95
Fiji	Pancreatic cancer	-0.01	-3.89	-0.01	-3.29	0	-4.98
Finland	Pancreatic cancer	-4.66	-28.15	-2.21	-28.28	-2.56	-29.36
France	Pancreatic cancer	-108.01	-80.29	-59.68	-76.32	-48.23	-85.63
Gabon	Pancreatic cancer	-0.03	-4.89	-0.02	-5.19	-0.01	-4.53
Gambia	Pancreatic cancer	0	-2.61	0	-2.47	0	-2.93
Georgia	Pancreatic cancer	-0.09	-2.61	-0.05	-2.26	-0.04	-3.44
Germany	Pancreatic cancer	-135.79	-55.92	-65.07	-53.55	-71.34	-58.8
Ghana	Pancreatic cancer	-0.18	-7.57	-0.07	-4.95	-0.11	-11.02
Greece	Pancreatic cancer	-2.21	-7.09	-1.17	-6.38	-1.04	-8.13
Greenland	Pancreatic cancer	-0.02	-8.42	-0.01	-8.42	-0.01	-8.72
Grenada	Pancreatic cancer	-0.01	-4.65	0	-4.68	0	-4.58
Guam	Pancreatic cancer	0	-6.04	0	-4.69	0	-8.38
Guatemala	Pancreatic cancer	-0.2	-10.67	-0.09	-9.78	-0.11	-11.62

Location	Cause	Attributable number (Both thousands)	Attributable proportion (Both %)	Attributable number (Male thousands)	Attributable proportion (Male %)	Attributable number (Female thousands)	Attributable proportion (Female %)
Guinea	Pancreatic cancer	-0.01	-1.48	-0.01	-1.16	0	-2.15
Guinea-Bissau	Pancreatic cancer	-0.01	-3.67	0	-3.02	0	-4.13
Guyana	Pancreatic cancer	-0.01	-3.76	-0.01	-3.6	-0.01	-3.89
Haiti	Pancreatic cancer	-0.09	-2.54	-0.05	-2.61	-0.04	-2.54
Honduras	Pancreatic cancer	-0.08	-5.27	-0.03	-4.28	-0.05	-6.21
Hungary	Pancreatic cancer	-2.81	-7.73	-1.46	-7.03	-1.34	-8.62
Iceland	Pancreatic cancer	-0.11	-18.76	-0.05	-17.48	-0.05	-20.32
India	Pancreatic cancer	-10.96	-7.94	-6.56	-7.33	-4.42	-9.1
Indonesia	Pancreatic cancer	-5.41	-8.41	-2.38	-7.26	-3.04	-9.62
Iran (Islamic Republic of)	Pancreatic cancer	-3.1	-22.97	-1.94	-23.07	-1.16	-22.77
Iraq	Pancreatic cancer	-1.03	-15.76	-0.6	-15.27	-0.43	-16.5
Ireland	Pancreatic cancer	-1.84	-20.86	-0.96	-19.97	-0.9	-22.25
Israel	Pancreatic cancer	-1.91	-19.16	-0.97	-19.04	-0.95	-19.58
Italy	Pancreatic cancer	-49.52	-27.39	-17.41	-17.66	-30.5	-37.12
Jamaica	Pancreatic cancer	-0.05	-3.57	-0.02	-3.61	-0.03	-3.7
Japan	Pancreatic cancer	-100.48	-29.68	-50.05	-25.21	-51.2	-36.58
Jordan	Pancreatic cancer	-0.27	-29.86	-0.15	-30.38	-0.11	-29.45
Kazakhstan	Pancreatic cancer	-0.72	-4.03	-0.39	-4.27	-0.36	-4.11
Kenya	Pancreatic cancer	-0.16	-4.76	-0.05	-3.44	-0.11	-5.57
Kiribati	Pancreatic cancer	0	-3.34	0	-2.91	0	-3.53
Kuwait	Pancreatic cancer	-0.18	-35.73	-0.11	-36.17	-0.07	-35.8

Location	Cause	Attributable number (Both thousands)	Attributable proportion (Both %)	Attributable number (Male thousands)	Attributable proportion (Male %)	Attributable number (Female thousands)	Attributable proportion (Female %)
Kyrgyzstan	Pancreatic cancer	-0.14	-4.33	-0.08	-3.94	-0.06	-4.76
Lao People's Democratic Republic	Pancreatic cancer	-0.12	-7.89	-0.06	-7.31	-0.06	-8.52
Latvia	Pancreatic cancer	-0.59	-7.91	-0.2	-4.8	-0.38	-11.28
Lebanon	Pancreatic cancer	-0.41	-21.03	-0.22	-20.97	-0.19	-21.41
Lesotho	Pancreatic cancer	0.02	3.68	0.01	4.65	0.01	2.93
Liberia	Pancreatic cancer	-0.02	-5.16	-0.01	-4.34	-0.01	-6.45
Libya	Pancreatic cancer	-0.29	-11.35	-0.17	-10.66	-0.12	-12.48
Lithuania	Pancreatic cancer	-0.71	-7.38	-0.26	-4.56	-0.42	-10.92
Luxembourg	Pancreatic cancer	-0.23	-19.8	-0.13	-19.28	-0.11	-21.03
Madagascar	Pancreatic cancer	-0.06	-2.19	-0.03	-2.07	-0.03	-2.31
Malawi	Pancreatic cancer	-0.03	-3.85	-0.01	-3.03	-0.02	-4.22
Malaysia	Pancreatic cancer	-0.67	-15.6	-0.37	-14.33	-0.3	-17.82
Maldives	Pancreatic cancer	-0.01	-24.36	-0.01	-23.75	-0.01	-25.49
Mali	Pancreatic cancer	-0.06	-2.93	-0.04	-2.63	-0.02	-3.75
Malta	Pancreatic cancer	-0.16	-18.75	-0.09	-17.12	-0.08	-20.76
Marshall Islands	Pancreatic cancer	0	-4.42	0	-4.45	0	-4.43
Mauritania	Pancreatic cancer	-0.04	-7.97	-0.02	-6.69	-0.02	-9.81
Mauritius	Pancreatic cancer	-0.09	-9.86	-0.05	-9.82	-0.04	-10.39
Mexico	Pancreatic cancer	-4.23	-7.15	-1.87	-6.52	-2.35	-7.7
Micronesia (Federated States of)	Pancreatic cancer	0	-4.41	0	-4	0	-4.99

Location	Cause	Attributable number (Both thousands)	Attributable proportion (Both %)	Attributable number (Male thousands)	Attributable proportion (Male %)	Attributable number (Female thousands)	Attributable proportion (Female %)
Monaco	Pancreatic cancer	-0.02	-9.66	-0.01	-9.22	-0.01	-10.21
Mongolia	Pancreatic cancer	-0.06	-14.66	-0.03	-12.78	-0.03	-16.86
Montenegro	Pancreatic cancer	-0.04	-3.5	-0.02	-3.22	-0.02	-4.13
Morocco	Pancreatic cancer	-0.35	-9.19	-0.21	-8.61	-0.14	-10.25
Mozambique	Pancreatic cancer	0	-0.45	0	0.3	0	-1.2
Myanmar	Pancreatic cancer	-1.34	-8.51	-0.61	-7.7	-0.73	-9.37
Namibia	Pancreatic cancer	-0.01	-4.17	0	-3.98	0	-4.45
Nauru	Pancreatic cancer	0	-3.05	0	-2.33	0	-4.17
Nepal	Pancreatic cancer	-0.17	-7.66	-0.09	-6.86	-0.08	-8.95
Netherlands	Pancreatic cancer	-1.73	-4.47	-0.94	-4.71	-0.82	-4.39
New Zealand	Pancreatic cancer	-1.98	-31.67	-1.08	-29.77	-0.9	-34.29
Nicaragua	Pancreatic cancer	-0.09	-11.22	-0.04	-10.08	-0.05	-12.25
Niger	Pancreatic cancer	-0.03	-3.33	-0.01	-2.62	-0.01	-4.87
Nigeria	Pancreatic cancer	-0.23	-3.43	-0.08	-2.93	-0.17	-4.22
Niue	Pancreatic cancer	0	-4.74	0	-4.38	0	-5.26
North Macedonia	Pancreatic cancer	-0.21	-6.5	-0.12	-6.03	-0.09	-7.31
Northern Mariana Islands	Pancreatic cancer	0	-11.34	0	-7.86	0	-19.96
Norway	Pancreatic cancer	-2.59	-20.42	-1.3	-19.77	-1.34	-21.93
Oman	Pancreatic cancer	-0.06	-24.28	-0.04	-24.7	-0.02	-23.22
Pakistan	Pancreatic cancer	-0.35	-2.52	-0.18	-2.1	-0.17	-3.09
Palau	Pancreatic cancer	0	-6.67	0	-6.4	0	-6.8

Location	Cause	Attributable number (Both thousands)	Attributable proportion (Both %)	Attributable number (Male thousands)	Attributable proportion (Male %)	Attributable number (Female thousands)	Attributable proportion (Female %)
Palestine	Pancreatic cancer	-0.12	-12.54	-0.07	-12.79	-0.05	-12.3
Panama	Pancreatic cancer	-0.11	-13.41	-0.05	-11.91	-0.06	-15.13
Papua New Guinea	Pancreatic cancer	-0.03	-4.38	-0.02	-3.47	-0.02	-5.86
Paraguay	Pancreatic cancer	-0.11	-8.55	-0.05	-7.7	-0.06	-9.37
Peru	Pancreatic cancer	-1.92	-13.44	-0.85	-12.14	-1.08	-14.68
Philippines	Pancreatic cancer	-0.94	-4.63	-0.45	-3.66	-0.48	-5.88
Poland	Pancreatic cancer	-4	-3.95	-2.32	-4.09	-1.69	-3.82
Portugal	Pancreatic cancer	-1.76	-8	-1	-8.08	-0.78	-8.04
Puerto Rico	Pancreatic cancer	-0.53	-14.88	-0.28	-15.02	-0.26	-15.21
Qatar	Pancreatic cancer	-0.12	-64.4	-0.08	-64.54	-0.03	-65.01
Republic of Korea	Pancreatic cancer	-42.37	-64.97	-22.97	-57.67	-19.67	-77.53
Republic of Moldova	Pancreatic cancer	-0.62	-6.88	-0.26	-4.87	-0.36	-10.19
Romania	Pancreatic cancer	-3.67	-7.8	-2.09	-6.93	-1.55	-9.19
Russian Federation	Pancreatic cancer	-20.84	-5.19	-11.48	-5.04	-9.6	-5.52
Rwanda	Pancreatic cancer	-0.13	-5.36	-0.06	-5.12	-0.07	-5.47
Saint Kitts and Nevis	Pancreatic cancer	0	-6.69	0	-6.57	0	-6.92
Saint Lucia	Pancreatic cancer	-0.01	-7.71	0	-7.91	0	-7.75
Saint Vincent and the Grenadines	Pancreatic cancer	0	-3.25	0	-3.57	0	-3.21
Samoa	Pancreatic cancer	0	-4.94	0	-4.18	0	-5.99
San Marino	Pancreatic cancer	-0.01	-9.39	0	-9.05	0	-10.13
Sao Tome and Principe	Pancreatic cancer	0	-5.44	0	-5.23	0	-5.75

Location	Cause	Attributable number (Both thousands)	Attributable proportion (Both %)	Attributable number (Male thousands)	Attributable proportion (Male %)	Attributable number (Female thousands)	Attributable proportion (Female %)
Saudi Arabia	Pancreatic cancer	-0.81	-31.11	-0.5	-30.06	-0.31	-33.35
Senegal	Pancreatic cancer	-0.07	-5.07	-0.04	-4.29	-0.03	-6.22
Serbia	Pancreatic cancer	-1.16	-5.24	-0.61	-4.73	-0.55	-5.98
Seychelles	Pancreatic cancer	-0.01	-8.08	0	-7.62	0	-8.99
Sierra Leone	Pancreatic cancer	-0.02	-3.33	-0.01	-2.8	-0.01	-3.97
Singapore	Pancreatic cancer	-1.91	-66.72	-1	-59.87	-0.91	-76.63
Slovakia	Pancreatic cancer	-0.97	-6.71	-0.66	-7.66	-0.31	-5.37
Slovenia	Pancreatic cancer	-1.16	-23.69	-0.56	-21.75	-0.64	-27.35
Solomon Islands	Pancreatic cancer	0	-5.63	0	-4.79	0	-7.2
Somalia	Pancreatic cancer	-0.02	-1.43	-0.01	-1.39	-0.01	-1.39
South Africa	Pancreatic cancer	-0.47	-2.12	-0.24	-1.91	-0.23	-2.5
South Sudan	Pancreatic cancer	-0.01	-0.85	-0.01	-0.64	-0.01	-1.13
Spain	Pancreatic cancer	-18.58	-21.76	-9.76	-20.09	-9.04	-24.56
Sri Lanka	Pancreatic cancer	-0.61	-15.26	-0.3	-13.2	-0.3	-17.71
Sudan	Pancreatic cancer	-0.31	-8.99	-0.18	-9.23	-0.12	-8.66
Suriname	Pancreatic cancer	-0.01	-3.81	-0.01	-3.36	-0.01	-4.27
Sweden	Pancreatic cancer	-0.72	-2.26	-0.36	-2.31	-0.39	-2.37
Switzerland	Pancreatic cancer	-3.4	-24.36	-1.97	-28.57	-1.71	-24.15
Syrian Arab Republic	Pancreatic cancer	-0.53	-15.56	-0.3	-15.28	-0.23	-15.77
Taiwan (Province of China)	Pancreatic cancer	-5.9	-31.84	-3.12	-25.31	-2.72	-43.93
Tajikistan	Pancreatic cancer	-0.01	-0.56	-0.01	-0.84	0	-0.46

Location	Cause	Attributable number (Both thousands)	Attributable proportion (Both %)	Attributable number (Male thousands)	Attributable proportion (Male %)	Attributable number (Female thousands)	Attributable proportion (Female %)
Thailand	Pancreatic cancer	-7.06	-20.14	-3.89	-18.64	-3.2	-22.6
Timor-Leste	Pancreatic cancer	-0.01	-6.77	0	-5.41	-0.01	-8.05
Togo	Pancreatic cancer	-0.02	-5.12	-0.01	-3.29	-0.01	-7.19
Tokelau	Pancreatic cancer	0	-5.93	0	-5.16	0	-6.86
Tonga	Pancreatic cancer	0	-4.26	0	-3.91	0	-5
Trinidad and Tobago	Pancreatic cancer	-0.06	-5.79	-0.03	-5.87	-0.03	-5.83
Tunisia	Pancreatic cancer	-0.37	-15.06	-0.22	-14.26	-0.15	-16.49
Turkey	Pancreatic cancer	-12.16	-19.49	-7.32	-19.05	-4.86	-20.29
Turkmenistan	Pancreatic cancer	-0.02	-21.24	-0.01	-22.55	-0.01	-22.73
Tuvalu	Pancreatic cancer	0	-6.3	0	-6.01	0	-6.73
Uganda	Pancreatic cancer	-0.25	-5.92	-0.15	-5.49	-0.1	-6.14
Ukraine	Pancreatic cancer	-4.21	-4.16	-2.62	-4.59	-1.59	-3.57
United Arab Emirates	Pancreatic cancer	-0.33	-29.19	-0.23	-30.02	-0.1	-27.19
United Kingdom	Pancreatic cancer	-17.58	-10.5	-8.8	-10.39	-8.94	-10.81
United Republic of Tanzania	Pancreatic cancer	-0.23	-3.39	-0.11	-3.18	-0.12	-3.57
United States of America	Pancreatic cancer	-101.4	-16.12	-46.33	-14.13	-55.71	-18.51
United States Virgin Islands	Pancreatic cancer	0	-4.56	0	-4.37	0	-5.45
Uruguay	Pancreatic cancer	-0.7	-7.44	-0.34	-6.96	-0.36	-7.88
Uzbekistan	Pancreatic cancer	-0.05	-1.03	-0.02	-0.95	-0.03	-1.42
Vanuatu	Pancreatic cancer	0	-3.86	0	-3.17	0	-5.16

Location	Cause	Attributable number (Both thousands)	Attributable proportion (Both %)	Attributable number (Male thousands)	Attributable proportion (Male %)	Attributable number (Female thousands)	Attributable proportion (Female %)
Venezuela (Bolivarian Republic of)	Pancreatic cancer	-0.63	-16.89	-0.28	-15.47	-0.36	-18.45
Viet Nam	Pancreatic cancer	-2.42	-16.68	-1.31	-15.11	-1.1	-19
Yemen	Pancreatic cancer	-0.12	-6.88	-0.07	-6.99	-0.05	-6.9
Zambia	Pancreatic cancer	-0.1	-4.48	-0.04	-3.5	-0.05	-5.17
Zimbabwe	Pancreatic cancer	0.08	1.73	0.04	1.8	0.05	1.9

Table S16. Absolute (the number) and relative contribution (the proportion) associated with disease severity changes for kidney cancer (KC), by sex at global, SDI regional, GBD regional level between 1990 and 2021.

Location	Cause	Attributable number (Both thousands)	Attributable proportion (Both %)	Attributable number (Male thousands)	Attributable proportion (Male %)	Attributable number (Female thousands)	Attributable proportion (Female %)
Global	Kidney cancer	-1195.23	-62.4	-843.58	-67.61	-387.95	-58.1
High SDI	Kidney cancer	-565.07	-64.47	-388.73	-68.84	-189.75	-60.84
High-middle SDI	Kidney cancer	-528.86	-83.82	-375.99	-91.31	-172.44	-78.68
Middle SDI	Kidney cancer	-384.5	-144	-265.99	-150.75	-123.1	-135.93
Low-middle SDI	Kidney cancer	-94.35	-97.26	-62.07	-94.93	-31.98	-101.16
Low SDI	Kidney cancer	-32.05	-78.17	-19.59	-71.24	-12.23	-90.64
High-income Asia Pacific	Kidney cancer	-81.28	-98.64	-56.77	-97.96	-25.32	-103.57
High-income North America	Kidney cancer	-133.95	-44.6	-93.03	-48.34	-44.56	-41.29
Western Europe	Kidney cancer	-390.26	-79.93	-266.14	-85.02	-133.16	-76
Australasia	Kidney cancer	-20.28	-110.91	-12.9	-113.32	-7.02	-101.77
Eastern Europe	Kidney cancer	-126.13	-48.31	-92.65	-56.58	-48.54	-49.85
Central Europe	Kidney cancer	-109.29	-80.34	-72.93	-81.11	-38.32	-83.1
Southern Latin America	Kidney cancer	-46.72	-92.85	-28.32	-84.51	-19.12	-113.74
East Asia	Kidney cancer	-523.36	-219.98	-375.44	-230.96	-154.58	-205.14
Central Asia	Kidney cancer	-10.11	-42.83	-7.66	-56.82	-3.84	-38
North Africa and Middle East	Kidney cancer	-71.97	-160.84	-46.86	-154.3	-25.88	-180.06
Andean Latin America	Kidney cancer	-16.27	-149.73	-9.37	-178.29	-7.45	-132.89
Southeast Asia	Kidney cancer	-39.04	-69.8	-26.42	-70.77	-12.83	-68.98

Location	Cause	Attributable number (Both thousands)	Attributable proportion (Both %)	Attributable number (Male thousands)	Attributable proportion (Male %)	Attributable number (Female thousands)	Attributable proportion (Female %)
Tropical Latin America	Kidney cancer	-46.43	-132.22	-28.47	-131.72	-18.21	-134.8
Southern Sub-Saharan Africa	Kidney cancer	-4.47	-58.4	-2.86	-58.4	-1.62	-58.73
Caribbean	Kidney cancer	-7.31	-69.65	-4.57	-73.36	-2.83	-66.62
Central Latin America	Kidney cancer	-58.79	-139.86	-35.47	-150.56	-24.84	-134.41
South Asia	Kidney cancer	-102.45	-134.74	-70.55	-122.85	-30.34	-163.02
Central Sub-Saharan Africa	Kidney cancer	-3.11	-76.41	-2.41	-77.74	-0.74	-77.64
Oceania	Kidney cancer	0	-1.58	0	-0.31	0	-15.38
Western Sub-Saharan Africa	Kidney cancer	-7.67	-81.2	-5.13	-72.95	-2.37	-97.74
Eastern Sub-Saharan Africa	Kidney cancer	-17.47	-84.74	-10.16	-80.17	-7.34	-92.33
Afghanistan	Kidney cancer	-1.35	-70.78	-0.53	-51.4	-0.71	-81.01
Albania	Kidney cancer	-0.99	-142.54	-0.75	-137.49	-0.22	-150.43
Algeria	Kidney cancer	-1.83	-188.42	-1.03	-159.03	-0.72	-222.99
American Samoa	Kidney cancer	0	-8.71	0	-8.78	0	-10.49
Andorra	Kidney cancer	-0.04	-84.69	-0.03	-80.68	-0.01	-91.53
Angola	Kidney cancer	-0.87	-117.98	-0.67	-113.28	-0.19	-132.22
Antigua and Barbuda	Kidney cancer	-0.02	-69.97	-0.01	-64.08	-0.01	-75.76
Argentina	Kidney cancer	-28.64	-83.98	-18.27	-76.88	-11.08	-107.25
Armenia	Kidney cancer	-0.92	-61.93	-0.63	-66.21	-0.32	-59.69
Australia	Kidney cancer	-18.51	-120.78	-11.72	-122.07	-6.43	-112.38
Austria	Kidney cancer	-7.97	-63.57	-4.93	-67.26	-3.22	-61.91

Location	Cause	Attributable number (Both thousands)	Attributable proportion (Both %)	Attributable number (Male thousands)	Attributable proportion (Male %)	Attributable number (Female thousands)	Attributable proportion (Female %)
Azerbaijan	Kidney cancer	-2.36	-62.55	-1.76	-70.24	-0.72	-56.31
Bahamas	Kidney cancer	-0.07	-68.53	-0.04	-73.66	-0.03	-68.37
Bahrain	Kidney cancer	-0.25	-215.26	-0.19	-233.45	-0.08	-223.94
Bangladesh	Kidney cancer	-10.05	-145.31	-7.41	-134.52	-2.49	-176.65
Barbados	Kidney cancer	-0.1	-66.45	-0.06	-62.17	-0.04	-71.61
Belarus	Kidney cancer	-6.4	-108.4	-4.5	-118.12	-2.08	-98.99
Belgium	Kidney cancer	-8.31	-66.49	-5.46	-73.16	-3.13	-62.38
Belize	Kidney cancer	-0.03	-102.08	-0.02	-102.43	-0.02	-107.94
Benin	Kidney cancer	-0.15	-100.92	-0.11	-100.91	-0.05	-107.11
Bermuda	Kidney cancer	-0.05	-90.86	-0.04	-97.7	-0.01	-83.11
Bhutan	Kidney cancer	-0.05	-138.25	-0.03	-125.27	-0.02	-164.05
Bolivia (Plurinational State of)	Kidney cancer	-2.2	-130.04	-1.11	-133.66	-1.13	-130.42
Bosnia and Herzegovina	Kidney cancer	-1.98	-97.25	-1.39	-106.43	-0.69	-94.5
Botswana	Kidney cancer	-0.11	-84.13	-0.07	-77.97	-0.04	-94.58
Brazil	Kidney cancer	-45.68	-132.66	-28	-131.98	-17.9	-135.38
Brunei Darussalam	Kidney cancer	-0.09	-124.48	-0.06	-128.41	-0.04	-122.84
Bulgaria	Kidney cancer	-2.84	-81.9	-2.05	-77.37	-0.62	-76.9
Burkina Faso	Kidney cancer	-0.2	-67.54	-0.14	-65.83	-0.07	-74.67
Burundi	Kidney cancer	-0.29	-49.67	-0.2	-56.25	-0.11	-46.95
Cabo Verde	Kidney cancer	-0.04	-1103.93	-0.03	-1857.87	-0.01	-636.97

Location	Cause	Attributable number (Both thousands)	Attributable proportion (Both %)	Attributable number (Male thousands)	Attributable proportion (Male %)	Attributable number (Female thousands)	Attributable proportion (Female %)
Cambodia	Kidney cancer	-0.93	-92.69	-0.6	-94.18	-0.35	-93.82
Cameroon	Kidney cancer	-0.48	-94.19	-0.34	-102.13	-0.17	-92.72
Canada	Kidney cancer	-13.94	-61.65	-8.63	-60.41	-4.31	-51.65
Central African Republic	Kidney cancer	-0.06	-27.09	-0.05	-28	-0.02	-29.13
Chad	Kidney cancer	-0.09	-57.33	-0.06	-57.98	-0.03	-62.41
Chile	Kidney cancer	-17.15	-147.47	-11.14	-165.86	-6.39	-130.08
China	Kidney cancer	-515.22	-224.79	-372.51	-237.35	-148.93	-206.14
Colombia	Kidney cancer	-10.42	-168.64	-6.08	-168.45	-4.39	-170.95
Comoros	Kidney cancer	-0.04	-68.64	-0.02	-65.26	-0.02	-73.92
Congo	Kidney cancer	-0.26	-102.75	-0.19	-103.42	-0.07	-102.68
Cook Islands	Kidney cancer	0	-55.28	0	-56.66	0	-54.02
Costa Rica	Kidney cancer	-1.03	-158.97	-0.61	-161.97	-0.43	-159.89
Coted'Ivoire	Kidney cancer	-0.23	-80.25	-0.17	-76.5	-0.06	-90.01
Croatia	Kidney cancer	-3.9	-66.38	-2.61	-64.27	-1.37	-75.25
Cuba	Kidney cancer	-3.2	-70.89	-2.02	-76.74	-1.26	-67.57
Cyprus	Kidney cancer	-0.58	-188.25	-0.41	-187.98	-0.17	-191.06
Czechia	Kidney cancer	-17.39	-89.04	-11.81	-86.31	-5.52	-94.46
Democratic People's Republic of Korea	Kidney cancer	-1.38	-34	-0.95	-38.5	-0.55	-34.62
Democratic Republic of the Congo	Kidney cancer	-1.66	-61.93	-1.31	-64.72	-0.4	-61.3
Denmark	Kidney cancer	-5	-101.45	-3.23	-111.9	-1.79	-87.59

Location	Cause	Attributable number (Both thousands)	Attributable proportion (Both %)	Attributable number (Male thousands)	Attributable proportion (Male %)	Attributable number (Female thousands)	Attributable proportion (Female %)
Djibouti	Kidney cancer	-0.05	-131.77	-0.03	-125.91	-0.02	-145.21
Dominica	Kidney cancer	-0.01	-38.15	-0.01	-40.08	0	-39.43
Dominican Republic	Kidney cancer	-0.85	-98.34	-0.46	-92.28	-0.39	-106.61
Ecuador	Kidney cancer	-2.91	-130.78	-1.62	-169.3	-1.45	-114.71
Egypt	Kidney cancer	-6.31	-159.59	-3.46	-133.74	-2.54	-185.91
El Salvador	Kidney cancer	-1.21	-147.41	-0.64	-145.96	-0.58	-151
Equatorial Guinea	Kidney cancer	-0.1	-290.01	-0.08	-295.79	-0.03	-304.16
Eritrea	Kidney cancer	-0.2	-73.38	-0.12	-69.17	-0.09	-78.59
Estonia	Kidney cancer	-1.57	-100.2	-1.01	-109.35	-0.58	-89.21
Eswatini	Kidney cancer	-0.04	-45.13	-0.03	-41.8	-0.02	-50.35
Ethiopia	Kidney cancer	-7.29	-95.84	-4.25	-95.32	-3.1	-98.48
Fiji	Kidney cancer	0	-10.29	0	-7	0	-14.98
Finland	Kidney cancer	-5.2	-70.7	-3.34	-75.7	-2.03	-69.1
France	Kidney cancer	-82.63	-117.02	-57.97	-122.17	-25.62	-110.59
Gabon	Kidney cancer	-0.13	-100.74	-0.1	-97.51	-0.04	-107.61
Gambia	Kidney cancer	-0.02	-77.71	-0.01	-65.13	-0.01	-89.7
Georgia	Kidney cancer	-0.35	-12.13	-0.48	-33.77	-0.21	-14.71
Germany	Kidney cancer	-83.04	-60.28	-56.23	-65.73	-30.98	-59.33
Ghana	Kidney cancer	-0.64	-145.35	-0.38	-128.54	-0.26	-172.48
Greece	Kidney cancer	-5.12	-51.38	-3.45	-51.62	-1.75	-53.23

Location	Cause	Attributable number (Both thousands)	Attributable proportion (Both %)	Attributable number (Male thousands)	Attributable proportion (Male %)	Attributable number (Female thousands)	Attributable proportion (Female %)
Greenland	Kidney cancer	-0.05	-101.73	-0.04	-108.48	-0.02	-85.5
Grenada	Kidney cancer	-0.02	-81.31	-0.01	-80.35	-0.01	-83.97
Guam	Kidney cancer	0	-1.63	0	-0.66	0	-8.31
Guatemala	Kidney cancer	-1.73	-135.25	-0.86	-140.79	-0.9	-134.81
Guinea	Kidney cancer	-0.13	-41.38	-0.1	-39.7	-0.03	-49.39
Guinea-Bissau	Kidney cancer	-0.02	-66.75	-0.02	-64.07	-0.01	-74.27
Guyana	Kidney cancer	-0.1	-58.52	-0.06	-57.67	-0.05	-61.31
Haiti	Kidney cancer	-0.66	-50.57	-0.35	-47.24	-0.31	-55.82
Honduras	Kidney cancer	-0.45	-118.85	-0.21	-96.84	-0.22	-138.44
Hungary	Kidney cancer	-10.6	-66.04	-6.99	-65.39	-3.56	-66.45
Iceland	Kidney cancer	-0.28	-86.39	-0.17	-87.09	-0.11	-87.23
India	Kidney cancer	-83.35	-142.88	-56.84	-131.04	-25.42	-169.93
Indonesia	Kidney cancer	-12.16	-65.37	-7.56	-63.13	-4.6	-69.44
Iran (Islamic Republic of)	Kidney cancer	-10.13	-162.66	-6.02	-148.02	-4.06	-187.47
Iraq	Kidney cancer	-4.21	-146.97	-2.74	-130	-1.32	-175.5
Ireland	Kidney cancer	-3.5	-118.34	-2.36	-122.9	-1.18	-114.62
Israel	Kidney cancer	-3.5	-114.18	-2.37	-127.15	-1.23	-102.6
Italy	Kidney cancer	-54.9	-74.43	-38.55	-77.3	-17.62	-73.75
Jamaica	Kidney cancer	-0.3	-56.8	-0.17	-54	-0.14	-61.75
Japan	Kidney cancer	-60.22	-84.81	-41.69	-83.35	-19.16	-91.24

Location	Cause	Attributable number (Both thousands)	Attributable proportion (Both %)	Attributable number (Male thousands)	Attributable proportion (Male %)	Attributable number (Female thousands)	Attributable proportion (Female %)
Jordan	Kidney cancer	-0.84	-243.23	-0.64	-250.91	-0.25	-276.5
Kazakhstan	Kidney cancer	-4.62	-48.67	-3.34	-62.84	-1.8	-43.07
Kenya	Kidney cancer	-0.91	-96.39	-0.4	-85.63	-0.51	-106.16
Kiribati	Kidney cancer	0	-16.1	0	-16.19	0	-17.82
Kuwait	Kidney cancer	-0.35	-214.31	-0.24	-185.07	-0.07	-232.52
Kyrgyzstan	Kidney cancer	-0.77	-122.35	-0.49	-117.49	-0.26	-121.3
Lao People's Democratic Republic	Kidney cancer	-0.36	-74.54	-0.22	-72.64	-0.13	-78.78
Latvia	Kidney cancer	-1.62	-90.06	-1	-83.37	-0.59	-99.21
Lebanon	Kidney cancer	-1.11	-168.33	-0.85	-176.59	-0.31	-176.51
Lesotho	Kidney cancer	-0.03	-19.7	-0.01	-11.78	-0.01	-26.71
Liberia	Kidney cancer	-0.11	-106.24	-0.08	-112.86	-0.03	-101.11
Libya	Kidney cancer	-1.07	-102.16	-0.74	-87.72	-0.26	-126.44
Lithuania	Kidney cancer	-1.71	-60.59	-1.18	-64.14	-0.57	-57.82
Luxembourg	Kidney cancer	-0.27	-105.58	-0.18	-110.03	-0.1	-102.27
Madagascar	Kidney cancer	-0.57	-54.41	-0.31	-48.52	-0.25	-61.9
Malawi	Kidney cancer	-1.11	-65.28	-0.69	-62.53	-0.42	-71.55
Malaysia	Kidney cancer	-2.53	-93.14	-1.74	-91.98	-0.8	-97.35
Maldives	Kidney cancer	-0.02	-177.07	-0.02	-198.22	0	-144.85
Mali	Kidney cancer	-0.32	-65.88	-0.18	-60.83	-0.14	-73.81
Malta	Kidney cancer	-0.33	-107.6	-0.21	-106.02	-0.13	-110.64

**Location**	**Cause**	**Attributable number (Both thousands)**	**Attributable proportion (Both %)**	**Attributable number (Male thousands)**	**Attributable proportion (Male %)**	**Attributable number (Female thousands)**	**Attributable proportion (Female %)**
Marshall Islands	Kidney cancer	0	-17.92	0	-18.59	0	-15.98
Mauritania	Kidney cancer	-0.14	-150.62	-0.09	-157.98	-0.05	-143.8
Mauritius	Kidney cancer	-0.07	-43.7	-0.05	-51.81	-0.02	-40.2
Mexico	Kidney cancer	-35.6	-147.15	-21.93	-151.65	-14.27	-146.69
Micronesia (Federated States of)	Kidney cancer	0	-29.76	0	-30.35	0	-28.64
Monaco	Kidney cancer	-0.03	-71.07	-0.02	-68.65	-0.01	-73.59
Mongolia	Kidney cancer	-0.38	-160.16	-0.28	-1784.44	-0.27	-121.69
Montenegro	Kidney cancer	-0.13	-31.34	-0.1	-34.61	-0.04	-29.72
Morocco	Kidney cancer	-0.86	-137.39	-0.46	-108.45	-0.34	-165.54
Mozambique	Kidney cancer	-0.32	-47.74	-0.19	-42.31	-0.13	-56.32
Myanmar	Kidney cancer	-3.6	-74.93	-2.09	-71.93	-1.48	-77.94
Namibia	Kidney cancer	-0.19	-89.38	-0.11	-87.03	-0.08	-93.57
Nauru	Kidney cancer	0	-12.6	0	-11.79	0	-16.56
Nepal	Kidney cancer	-1.44	-129.25	-0.94	-120.7	-0.49	-146.46
Netherlands	Kidney cancer	-14.02	-74.41	-9.74	-79.33	-4.93	-75.16
New Zealand	Kidney cancer	-2.03	-68.56	-1.31	-73.43	-0.71	-59.86
Nicaragua	Kidney cancer	-0.59	-148.86	-0.34	-158.27	-0.27	-144.95
Niger	Kidney cancer	-0.14	-77.02	-0.1	-73.89	-0.04	-86.12
Nigeria	Kidney cancer	-4.11	-69.87	-2.76	-60.37	-1.19	-90.7
Niue	Kidney cancer	0	-21.97	0	-22.49	0	-21.55

Location	Cause	Attributable number (Both thousands)	Attributable proportion (Both %)	Attributable number (Male thousands)	Attributable proportion (Male %)	Attributable number (Female thousands)	Attributable proportion (Female %)
North Macedonia	Kidney cancer	-0.54	-104.41	-0.39	-103.54	-0.15	-105.88
Northern Mariana Islands	Kidney cancer	0	-13.41	0	-12.33	0	-34.51
Norway	Kidney cancer	-4.46	-74.44	-3.15	-82.56	-1.43	-65.92
Oman	Kidney cancer	-0.21	-197.66	-0.17	-195.46	-0.05	-229.22
Pakistan	Kidney cancer	-6.36	-66.03	-4.46	-57.68	-1.71	-89.87
Palau	Kidney cancer	0	-26.91	0	-27.14	0	-27.7
Palestine	Kidney cancer	-0.29	-110.04	-0.2	-118.23	-0.12	-124.86
Panama	Kidney cancer	-0.93	-126.21	-0.53	-141.8	-0.43	-118.94
Papua New Guinea	Kidney cancer	-0.01	-4.19	-0.01	-4.3	0	-9.63
Paraguay	Kidney cancer	-0.76	-110.65	-0.47	-117.6	-0.31	-109.29
Peru	Kidney cancer	-11.18	-160.94	-6.58	-189.6	-4.95	-142.57
Philippines	Kidney cancer	-1.59	-20.73	-0.85	-16.17	-0.64	-26.23
Poland	Kidney cancer	-40.29	-69.91	-27.31	-73.82	-14.67	-71.09
Portugal	Kidney cancer	-8.21	-131.37	-5.43	-136.85	-2.74	-119.97
Puerto Rico	Kidney cancer	-1.54	-95.14	-1.01	-93.72	-0.53	-98.51
Qatar	Kidney cancer	-0.25	-311.5	-0.23	-374.02	-0.05	-306.02
Republic of Korea	Kidney cancer	-33.37	-321.97	-24.61	-339.95	-9.12	-291.82
Republic of Moldova	Kidney cancer	-1.47	-58.41	-1.07	-65.62	-0.46	-51.89
Romania	Kidney cancer	-12.94	-107.87	-8.93	-122.85	-4.73	-100.1
Russian Federation	Kidney cancer	-100.73	-53.46	-70.62	-59.18	-38.81	-56.17

Location	Cause	Attributable number (Both thousands)	Attributable proportion (Both %)	Attributable number (Male thousands)	Attributable proportion (Male %)	Attributable number (Female thousands)	Attributable proportion (Female %)
Rwanda	Kidney cancer	-0.82	-89.41	-0.45	-89.8	-0.38	-91.05
Saint Kitts and Nevis	Kidney cancer	-0.02	-97.2	-0.01	-113.31	-0.01	-87.73
Saint Lucia	Kidney cancer	-0.04	-94.9	-0.02	-98.4	-0.01	-94.64
Saint Vincent and the Grenadines	Kidney cancer	-0.02	-52.12	-0.01	-59.21	-0.01	-52.37
Samoa	Kidney cancer	0	-25.87	0	-23.15	0	-28.32
San Marino	Kidney cancer	-0.01	-49.9	-0.01	-50.15	0	-50.66
Sao Tome and Principe	Kidney cancer	0	-95.33	0	-95.11	0	-104.43
Saudi Arabia	Kidney cancer	-4.06	-333.11	-3.08	-305.85	-0.8	-378.34
Senegal	Kidney cancer	-0.26	-96.91	-0.18	-95.6	-0.08	-103.99
Serbia	Kidney cancer	-6.42	-74.16	-4.26	-74.13	-2.2	-75.88
Seychelles	Kidney cancer	-0.01	-44.28	-0.01	-48.72	0	-41.56
Sierra Leone	Kidney cancer	-0.1	-71.19	-0.07	-67.67	-0.03	-79.34
Singapore	Kidney cancer	-2.07	-218.45	-1.41	-215.32	-0.66	-225.86
Slovakia	Kidney cancer	-5.17	-95.39	-3.66	-96.35	-1.54	-95.34
Slovenia	Kidney cancer	-1.84	-116.21	-1.27	-119.66	-0.6	-114.45
Solomon Islands	Kidney cancer	0	-22.91	0	-22.12	0	-30.01
Somalia	Kidney cancer	-0.2	-34.13	-0.13	-33.55	-0.07	-37.57
South Africa	Kidney cancer	-3.95	-62.96	-2.53	-63.42	-1.45	-63.86
South Sudan	Kidney cancer	-0.3	-42.54	-0.18	-35.55	-0.11	-51.86
Spain	Kidney cancer	-31.89	-104.93	-20.81	-101.52	-10.97	-110.82

Location	Cause	Attributable number (Both thousands)	Attributable proportion (Both %)	Attributable number (Male thousands)	Attributable proportion (Male %)	Attributable number (Female thousands)	Attributable proportion (Female %)
Sri Lanka	Kidney cancer	-4.07	-44	-2.76	-43.23	-1.3	-45.16
Sudan	Kidney cancer	-2.39	-126.79	-1.42	-107.81	-0.86	-151.09
Suriname	Kidney cancer	-0.06	-64.53	-0.04	-60.95	-0.03	-70.15
Sweden	Kidney cancer	-5.34	-33.12	-3.36	-35.82	-2.07	-30.75
Switzerland	Kidney cancer	-3.42	-85.27	-2.39	-89.98	-1.09	-80.55
Syrian Arab Republic	Kidney cancer	-1.84	-130.17	-1.24	-125.7	-0.62	-143.7
Taiwan (Province of China)	Kidney cancer	-6.91	-148.38	-4.38	-139.44	-2.53	-166.83
Tajikistan	Kidney cancer	-0.29	-22.66	-0.23	-25.22	-0.09	-21.74
Thailand	Kidney cancer	-10.06	-125.48	-7.32	-128.49	-2.89	-124.82
Timor-Leste	Kidney cancer	-0.03	-50.56	-0.02	-47.96	-0.01	-56.68
Togo	Kidney cancer	-0.1	-105.67	-0.07	-102.28	-0.03	-117.49
Tokelau	Kidney cancer	0	-40.86	0	-41.31	0	-40.79
Tonga	Kidney cancer	0	-16.09	0	-16.35	0	-18.67
Trinidad and Tobago	Kidney cancer	-0.36	-80.77	-0.19	-78.45	-0.17	-84.4
Tunisia	Kidney cancer	-1.62	-131	-1.06	-114.05	-0.51	-163.47
Turkey	Kidney cancer	-29.37	-159.99	-19.5	-159.07	-10.55	-173.09
Turkmenistan	Kidney cancer	-0.36	-56.12	-0.46	-284.12	-0.24	-48.98
Tuvalu	Kidney cancer	0	-49.12	0	-50.85	0	-44.25
Uganda	Kidney cancer	-1.47	-90.83	-0.92	-77.92	-0.49	-110.76
Ukraine	Kidney cancer	-9.27	-15.96	-8.95	-25.57	-5.24	-22.72

**Location**	**Cause**	**Attributable number (Both thousands)**	**Attributable proportion (Both %)**	**Attributable number (Male thousands)**	**Attributable proportion (Male %)**	**Attributable number (Female thousands)**	**Attributable proportion (Female %)**
United Arab Emirates	Kidney cancer	-0.79	-269.51	-0.59	-296.09	-0.28	-300.22
United Kingdom	Kidney cancer	-52.17	-75.05	-34.97	-79.61	-18.02	-70.4
United Republic of Tanzania	Kidney cancer	-2.11	-69.69	-1.19	-62.51	-0.88	-77.95
United States of America	Kidney cancer	-122.11	-43.98	-84.4	-47.38	-41.56	-41.75
United States Virgin Islands	Kidney cancer	-0.03	-45.85	-0.02	-45.43	-0.01	-49.47
Uruguay	Kidney cancer	-3.85	-84.1	-2.52	-83.37	-1.36	-87.03
Uzbekistan	Kidney cancer	-1.37	-43.01	-0.88	-48.66	-0.57	-41.72
Vanuatu	Kidney cancer	0	-2.68	0	-2.18	0	-8.09
Venezuela (Bolivarian Republic of)	Kidney cancer	-7.03	-94.93	-4.13	-126.96	-3.48	-83.78
Viet Nam	Kidney cancer	-4.34	-142.42	-3.08	-147.65	-1.33	-138.59
Yemen	Kidney cancer	-0.93	-94.66	-0.54	-77.59	-0.32	-110.61
Zambia	Kidney cancer	-1.03	-125.68	-0.73	-149.76	-0.37	-113.02
Zimbabwe	Kidney cancer	-0.13	-16.36	-0.03	-6.39	-0.05	-18.85

Table S17. Absolute (the number) and relative contribution (the proportion) associated with disease severity changes for colon and rectum cancer (CRC), by sex at global, SDI regional, GBD regional level between 1990 and 2021.

Location	Cause	Attributable number (Both thousands)	Attributable proportion (Both %)	Attributable number (Male thousands)	Attributable proportion (Male %)	Attributable number (Female thousands)	Attributable proportion (Female %)
Global	Colon and rectum cancer	-9334.1	-65.27	-5533.41	-73.24	-3865.96	-57.3
High SDI	Colon and rectum cancer	-2852.15	-53.45	-1612.66	-57.61	-1265.24	-49.87
High-middle SDI	Colon and rectum cancer	-4455.46	-101.5	-2719.56	-116.63	-1794.48	-87.19
Middle SDI	Colon and rectum cancer	-4297.12	-138.73	-2567.23	-152.2	-1742	-123.48
Low-middle SDI	Colon and rectum cancer	-622.18	-61.6	-308.52	-63.4	-315.1	-60.19
Low SDI	Colon and rectum cancer	-165.18	-36.82	-86.47	-35.93	-78.03	-37.53
High-income Asia Pacific	Colon and rectum cancer	-552.41	-63.35	-323.11	-66.64	-230.21	-59.47
High-income North America	Colon and rectum cancer	-582.01	-36.12	-326.6	-38.71	-261.46	-34.07
Western Europe	Colon and rectum cancer	-1707.55	-60.7	-960.79	-66.58	-767.6	-56.04
Australasia	Colon and rectum cancer	-94.34	-70.7	-53.79	-74.94	-41.5	-67.3
Eastern Europe	Colon and rectum cancer	-579.23	-45.31	-287.63	-49.64	-312.25	-44.66
Central Europe	Colon and rectum cancer	-533.52	-69.65	-313.35	-74.97	-225.1	-64.69
Southern Latin America	Colon and rectum cancer	-139.92	-68.06	-77.22	-69.36	-63.06	-66.89
East Asia	Colon and rectum cancer	-6673.35	-183.41	-4152.38	-199.62	-2552.66	-163.81
Central Asia	Colon and rectum cancer	-41.54	-29.86	-22.03	-31.39	-20.13	-29.19

Location	Cause	Attributable number (Both thousands)	Attributable proportion (Both %)	Attributable number (Male thousands)	Attributable proportion (Male %)	Attributable number (Female thousands)	Attributable proportion (Female %)
North Africa and Middle East	Colon and rectum cancer	-533.9	-131.86	-272.32	-127.78	-259.36	-135.22
Andean Latin America	Colon and rectum cancer	-60.97	-140.21	-28.76	-141.71	-32.17	-138.72
Southeast Asia	Colon and rectum cancer	-683.27	-93.96	-381.29	-102.22	-303.65	-85.74
Tropical Latin America	Colon and rectum cancer	-224.92	-103.77	-116.37	-110.74	-109.06	-97.67
Southern Sub-Saharan Africa	Colon and rectum cancer	-14.09	-23.28	-8.01	-25.47	-6.02	-20.7
Caribbean	Colon and rectum cancer	-65.99	-79.38	-31.35	-78.66	-34.66	-80.08
Central Latin America	Colon and rectum cancer	-218.32	-148.36	-118.82	-169.82	-91.18	-118.13
South Asia	Colon and rectum cancer	-448.51	-57.54	-227.37	-59.42	-221.44	-55.81
Central Sub-Saharan Africa	Colon and rectum cancer	-14.53	-33.36	-8.04	-34.33	-6.49	-32.19
Oceania	Colon and rectum cancer	-1.14	-22.47	-0.51	-20.56	-0.64	-24.42
Western Sub-Saharan Africa	Colon and rectum cancer	-31.65	-28.87	-16.67	-27.52	-14.68	-29.91
Eastern Sub-Saharan Africa	Colon and rectum cancer	-84.93	-37.98	-52.33	-39.95	-32.53	-35.12
Afghanistan	Colon and rectum cancer	-10.15	-43.99	-2.79	-34.1	-7.16	-48.09
Albania	Colon and rectum cancer	-3.01	-85.73	-1.8	-90.28	-1.21	-80.43
Algeria	Colon and rectum cancer	-15.38	-112.28	-7.91	-108.26	-7.4	-115.67
American Samoa	Colon and rectum cancer	-0.03	-29.28	-0.01	-28.21	-0.01	-30.1
Andorra	Colon and rectum cancer	-0.22	-69.22	-0.17	-69.95	-0.05	-66.55

Location	Cause	Attributable number (Both thousands)	Attributable proportion (Both %)	Attributable number (Male thousands)	Attributable proportion (Male %)	Attributable number (Female thousands)	Attributable proportion (Female %)
Angola	Colon and rectum cancer	-4.36	-51.26	-2.53	-49.84	-1.79	-52.09
Antigua and Barbuda	Colon and rectum cancer	-0.11	-74.51	-0.05	-71.67	-0.06	-75.39
Argentina	Colon and rectum cancer	-82.41	-52.97	-47.02	-54.2	-35.59	-51.69
Armenia	Colon and rectum cancer	-4.42	-42.45	-2.28	-45.38	-2.2	-40.85
Australia	Colon and rectum cancer	-77.22	-72.6	-44.15	-76.27	-33.75	-69.6
Austria	Colon and rectum cancer	-32.73	-52.81	-18.44	-58.81	-14.78	-48.26
Azerbaijan	Colon and rectum cancer	-5.56	-41.32	-3.14	-43.01	-2.47	-40.04
Bahamas	Colon and rectum cancer	-0.46	-68.57	-0.25	-69.57	-0.2	-67.71
Bahrain	Colon and rectum cancer	-1.2	-218.3	-0.73	-219.72	-0.49	-222.47
Bangladesh	Colon and rectum cancer	-48.82	-77.78	-26.46	-74.97	-22.12	-80.52
Barbados	Colon and rectum cancer	-0.85	-72.06	-0.43	-74.9	-0.43	-69.85
Belarus	Colon and rectum cancer	-33.13	-62.64	-16.35	-67	-17.39	-61.04
Belgium	Colon and rectum cancer	-42.01	-52.8	-23.07	-57.58	-19.42	-49.16
Belize	Colon and rectum cancer	-0.13	-88.38	-0.06	-86.77	-0.06	-88.68
Benin	Colon and rectum cancer	-0.71	-30.23	-0.39	-30.24	-0.31	-30.16
Bermuda	Colon and rectum cancer	-0.43	-111	-0.24	-115.28	-0.19	-108.18
Bhutan	Colon and rectum cancer	-0.22	-66.01	-0.11	-67.93	-0.11	-64.55

Location	Cause	Attributable number (Both thousands)	Attributable proportion (Both %)	Attributable number (Male thousands)	Attributable proportion (Male %)	Attributable number (Female thousands)	Attributable proportion (Female %)
Bolivia (Plurinational State of)	Colon and rectum cancer	-8.66	-83.05	-3.56	-85.97	-5.09	-80.95
Bosnia and Herzegovina	Colon and rectum cancer	-9.93	-71.06	-5.86	-82.08	-4.23	-61.82
Botswana	Colon and rectum cancer	-0.41	-30.07	-0.23	-30.74	-0.19	-30.05
Brazil	Colon and rectum cancer	-221.91	-103.98	-114.8	-110.81	-107.62	-97.99
Brunei Darussalam	Colon and rectum cancer	-0.55	-69.52	-0.32	-70.52	-0.23	-67.29
Bulgaria	Colon and rectum cancer	-33.55	-52.77	-19.43	-54.59	-14.57	-52.05
Burkina Faso	Colon and rectum cancer	-1.04	-20.2	-0.53	-20.26	-0.52	-20.49
Burundi	Colon and rectum cancer	-1.31	-22	-0.78	-26.5	-0.58	-19.25
Cabo Verde	Colon and rectum cancer	-0.12	-69.52	-0.07	-85.1	-0.06	-60.27
Cambodia	Colon and rectum cancer	-12.55	-74.6	-6.1	-78.99	-6.51	-71.44
Cameroon	Colon and rectum cancer	-2.17	-31.18	-1.19	-31.45	-1	-31.4
Canada	Colon and rectum cancer	-84.8	-56.28	-48.7	-61.4	-36.73	-51.46
Central African Republic	Colon and rectum cancer	-0.26	-8.51	-0.16	-9.54	-0.1	-7.15
Chad	Colon and rectum cancer	-0.29	-10.21	-0.16	-11.06	-0.15	-10.26
Chile	Colon and rectum cancer	-39.62	-142.57	-20.46	-150.59	-19.34	-136.13
China	Colon and rectum cancer	-6534.57	-186	-4077.47	-203.06	-2490.02	-165.43
Colombia	Colon and rectum cancer	-77.34	-188.04	-39.79	-217.62	-34.62	-151.53

Location	Cause	Attributable number (Both thousands)	Attributable proportion (Both %)	Attributable number (Male thousands)	Attributable proportion (Male %)	Attributable number (Female thousands)	Attributable proportion (Female %)
Comoros	Colon and rectum cancer	-0.14	-28.49	-0.07	-29.79	-0.07	-27.59
Congo	Colon and rectum cancer	-1.35	-44.25	-0.78	-46.39	-0.58	-41.99
Cook Islands	Colon and rectum cancer	-0.02	-76.76	-0.01	-76.64	-0.01	-77.14
Costa Rica	Colon and rectum cancer	-6.03	-153.18	-3.01	-179.58	-2.44	-107.88
Coted'Ivoire	Colon and rectum cancer	-1.4	-33.72	-0.75	-33.04	-0.65	-34
Croatia	Colon and rectum cancer	-23.05	-72.93	-13.9	-81.56	-9.99	-68.62
Cuba	Colon and rectum cancer	-31.41	-86.28	-13.78	-83.1	-17.63	-88.88
Cyprus	Colon and rectum cancer	-4.06	-146.8	-2.29	-155.92	-1.8	-139.18
Czechia	Colon and rectum cancer	-75.91	-70.33	-47.08	-76.09	-29.52	-64.09
Democratic People's Republic of Korea	Colon and rectum cancer	-27.69	-52.03	-15.03	-54.54	-12.94	-50.45
Democratic Republic of the Congo	Colon and rectum cancer	-7.17	-27.03	-3.67	-27.14	-3.49	-26.82
Denmark	Colon and rectum cancer	-29.18	-70.92	-16.07	-79.21	-13.4	-64.24
Djibouti	Colon and rectum cancer	-0.18	-50.57	-0.12	-56.51	-0.07	-45.8
Dominica	Colon and rectum cancer	-0.05	-28.95	-0.02	-33.03	-0.02	-27.23
Dominican Republic	Colon and rectum cancer	-5.84	-86.55	-2.95	-90.94	-2.92	-83.16
Ecuador	Colon and rectum cancer	-11.31	-129.93	-5.24	-131.64	-6.07	-128.39
Egypt	Colon and rectum cancer	-54.18	-117.6	-27.5	-106.02	-24.85	-123.4

Location	Cause	Attributable number (Both thousands)	Attributable proportion (Both %)	Attributable number (Male thousands)	Attributable proportion (Male %)	Attributable number (Female thousands)	Attributable proportion (Female %)
El Salvador	Colon and rectum cancer	-6.14	-150.6	-3.23	-173.72	-2.81	-126.67
Equatorial Guinea	Colon and rectum cancer	-0.53	-117.86	-0.33	-129.9	-0.21	-107.56
Eritrea	Colon and rectum cancer	-1.05	-29.9	-0.58	-34	-0.49	-26.91
Estonia	Colon and rectum cancer	-6.86	-75.63	-3.62	-87.24	-3.49	-71.05
Eswatini	Colon and rectum cancer	-0.1	-11.78	-0.06	-12.41	-0.04	-11.63
Ethiopia	Colon and rectum cancer	-49.39	-43.99	-33.86	-44.9	-15.61	-42.34
Fiji	Colon and rectum cancer	-0.19	-21.03	-0.09	-18.74	-0.1	-23.53
Finland	Colon and rectum cancer	-17.03	-73.74	-9.24	-84	-8.02	-66.34
France	Colon and rectum cancer	-319.71	-78.04	-177.34	-78.55	-143.93	-78.26
Gabon	Colon and rectum cancer	-0.82	-42.27	-0.53	-44.43	-0.3	-39.81
Gambia	Colon and rectum cancer	-0.06	-24.99	-0.03	-22.95	-0.03	-26.18
Georgia	Colon and rectum cancer	-2.17	-12.46	-1.33	-14.44	-0.98	-12.01
Germany	Colon and rectum cancer	-367.91	-51.74	-199.05	-59.95	-176.09	-46.45
Ghana	Colon and rectum cancer	-3.17	-39.72	-1.25	-38.08	-1.94	-41.2
Greece	Colon and rectum cancer	-15.74	-32.24	-8.72	-34.82	-7.35	-30.88
Greenland	Colon and rectum cancer	-0.2	-53.99	-0.11	-57.39	-0.09	-50.96
Grenada	Colon and rectum cancer	-0.15	-65.58	-0.07	-71.73	-0.08	-62.77

Location	Cause	Attributable number (Both thousands)	Attributable proportion (Both %)	Attributable number (Male thousands)	Attributable proportion (Male %)	Attributable number (Female thousands)	Attributable proportion (Female %)
Guam	Colon and rectum cancer	-0.11	-36.61	-0.06	-32.4	-0.04	-44.87
Guatemala	Colon and rectum cancer	-5.55	-131.06	-2.74	-152.06	-2.66	-109.41
Guinea	Colon and rectum cancer	-0.47	-14.31	-0.23	-13	-0.24	-16.25
Guinea-Bissau	Colon and rectum cancer	-0.16	-20.51	-0.09	-20.03	-0.06	-19.43
Guyana	Colon and rectum cancer	-0.53	-48.44	-0.29	-47.94	-0.24	-48.96
Haiti	Colon and rectum cancer	-4.17	-34.12	-1.66	-32.51	-2.49	-34.97
Honduras	Colon and rectum cancer	-1.56	-63.09	-0.71	-70.98	-0.85	-58.45
Hungary	Colon and rectum cancer	-66.3	-65.44	-40.17	-73.13	-27	-58.19
Iceland	Colon and rectum cancer	-0.57	-58.03	-0.31	-63.08	-0.27	-54.75
India	Colon and rectum cancer	-372.8	-59.11	-187.24	-61.66	-185.9	-56.86
Indonesia	Colon and rectum cancer	-149.92	-56.36	-74.08	-58.75	-76.52	-54.68
Iran (Islamic Republic of)	Colon and rectum cancer	-64.08	-127.71	-28.83	-107.13	-35.44	-152.32
Iraq	Colon and rectum cancer	-16.65	-134.45	-8.69	-125.09	-7.84	-144.32
Ireland	Colon and rectum cancer	-19.02	-84.32	-11.62	-91.37	-7.57	-76.97
Israel	Colon and rectum cancer	-18.03	-83.79	-9.69	-88.42	-8.52	-80.65
Italy	Colon and rectum cancer	-245.63	-64.23	-140.47	-68.86	-107.28	-60.14
Jamaica	Colon and rectum cancer	-2.46	-57.89	-1.26	-59.99	-1.22	-56.39

Location	Cause	Attributable number (Both thousands)	Attributable proportion (Both %)	Attributable number (Male thousands)	Attributable proportion (Male %)	Attributable number (Female thousands)	Attributable proportion (Female %)
Japan	Colon and rectum cancer	-398.67	-53.08	-236.4	-56.07	-163.82	-49.73
Jordan	Colon and rectum cancer	-8.33	-197.27	-4.49	-198.72	-4.01	-204.39
Kazakhstan	Colon and rectum cancer	-21.02	-38.48	-10.97	-40.72	-10.31	-37.23
Kenya	Colon and rectum cancer	-3.31	-30.37	-1.15	-26.92	-2.18	-33.09
Kiribati	Colon and rectum cancer	-0.02	-18.1	-0.01	-18.07	-0.01	-17.46
Kuwait	Colon and rectum cancer	-2.23	-226.95	-1.31	-220.5	-0.9	-232.39
Kyrgyzstan	Colon and rectum cancer	-3.22	-34.91	-1.62	-36.92	-1.63	-33.7
Lao People's Democratic Republic	Colon and rectum cancer	-4.14	-51.28	-2.12	-52.98	-2.03	-50.04
Latvia	Colon and rectum cancer	-6.4	-41.8	-3.18	-46.1	-3.45	-40.87
Lebanon	Colon and rectum cancer	-9.41	-137.69	-5.22	-135.21	-4.13	-138.92
Lesotho	Colon and rectum cancer	0.17	12.85	0.08	13.28	0.09	11.95
Liberia	Colon and rectum cancer	-0.52	-38.56	-0.28	-35.95	-0.24	-41.6
Libya	Colon and rectum cancer	-6.12	-94.38	-2.9	-81.08	-3.08	-105.94
Lithuania	Colon and rectum cancer	-6.78	-38.01	-3.55	-41.15	-3.3	-35.86
Luxembourg	Colon and rectum cancer	-2.42	-78	-1.38	-85.23	-1.07	-72.06
Madagascar	Colon and rectum cancer	-2.28	-20.29	-1.07	-20.15	-1.21	-20.56
Malawi	Colon and rectum cancer	-1.06	-25.57	-0.48	-25.88	-0.59	-26

Location	Cause	Attributable number (Both thousands)	Attributable proportion (Both %)	Attributable number (Male thousands)	Attributable proportion (Male %)	Attributable number (Female thousands)	Attributable proportion (Female %)
Malaysia	Colon and rectum cancer	-43.8	-111.05	-24.31	-120.01	-19.81	-103.3
Maldives	Colon and rectum cancer	-0.27	-150.35	-0.16	-171.12	-0.11	-129.67
Mali	Colon and rectum cancer	-1.81	-25.93	-1.03	-26.23	-0.79	-25.82
Malta	Colon and rectum cancer	-1.43	-82.08	-0.72	-81.03	-0.72	-83.58
Marshall Islands	Colon and rectum cancer	-0.01	-24.84	-0.01	-26.07	-0.01	-23.22
Mauritania	Colon and rectum cancer	-0.86	-57.37	-0.42	-56.34	-0.44	-58.22
Mauritius	Colon and rectum cancer	-1.44	-92.23	-0.81	-105.7	-0.66	-82.79
Mexico	Colon and rectum cancer	-90.11	-140.46	-52.03	-161.65	-34.71	-108.57
Micronesia (Federated States of)	Colon and rectum cancer	-0.05	-30	-0.02	-29.45	-0.02	-30.55
Monaco	Colon and rectum cancer	-0.15	-42.31	-0.09	-46.27	-0.06	-38.62
Mongolia	Colon and rectum cancer	-1.4	-59.63	-0.68	-61.3	-0.73	-58.75
Montenegro	Colon and rectum cancer	-0.52	-24.9	-0.35	-27.29	-0.18	-22.18
Morocco	Colon and rectum cancer	-23.22	-102.3	-8.92	-87.84	-13.7	-109.32
Mozambique	Colon and rectum cancer	-0.31	-7.96	-0.14	-6.82	-0.19	-10.08
Myanmar	Colon and rectum cancer	-45.21	-57.99	-21.79	-61.78	-23.65	-55.42
Namibia	Colon and rectum cancer	-0.4	-41.09	-0.21	-42.53	-0.19	-40.44
Nauru	Colon and rectum cancer	-0.01	-21.6	0	-19.13	0	-23.78

Location	Cause	Attributable number (Both thousands)	Attributable proportion (Both %)	Attributable number (Male thousands)	Attributable proportion (Male %)	Attributable number (Female thousands)	Attributable proportion (Female %)
Nepal	Colon and rectum cancer	-7.04	-61.68	-3.18	-60.45	-3.87	-62.88
Netherlands	Colon and rectum cancer	-28.39	-27.08	-15.58	-29.76	-13.23	-25.22
New Zealand	Colon and rectum cancer	-16.89	-62.43	-9.59	-69.02	-7.56	-57.42
Nicaragua	Colon and rectum cancer	-2.4	-125.63	-1.23	-147.4	-1.1	-102.34
Niger	Colon and rectum cancer	-0.75	-24.02	-0.38	-22.46	-0.35	-24.62
Nigeria	Colon and rectum cancer	-14.52	-26.52	-7.83	-24.65	-6.43	-28
Niue	Colon and rectum cancer	0	-30.12	0	-32.08	0	-28.82
North Macedonia	Colon and rectum cancer	-4.57	-64.02	-2.72	-67.92	-1.88	-59.95
Northern Mariana Islands	Colon and rectum cancer	-0.04	-44.66	-0.02	-42.21	-0.01	-50.33
Norway	Colon and rectum cancer	-21.93	-63.74	-12.77	-72.06	-9.48	-56.82
Oman	Colon and rectum cancer	-1.07	-139.77	-0.7	-139.21	-0.38	-143.47
Pakistan	Colon and rectum cancer	-17.35	-23.37	-8.75	-22.89	-8.61	-23.9
Palau	Colon and rectum cancer	-0.01	-42.8	0	-42.76	-0.01	-43.12
Palestine	Colon and rectum cancer	-4.02	-104.76	-1.91	-104.29	-2.2	-109.65
Panama	Colon and rectum cancer	-4.88	-155.61	-2.9	-176.82	-1.69	-112.99
Papua New Guinea	Colon and rectum cancer	-0.43	-19.86	-0.16	-17.78	-0.28	-21.38
Paraguay	Colon and rectum cancer	-2.98	-89.66	-1.53	-102.91	-1.44	-78.86

Location	Cause	Attributable number (Both thousands)	Attributable proportion (Both %)	Attributable number (Male thousands)	Attributable proportion (Male %)	Attributable number (Female thousands)	Attributable proportion (Female %)
Peru	Colon and rectum cancer	-38.67	-158.81	-19.18	-157.49	-19.55	-160.64
Philippines	Colon and rectum cancer	-29.61	-32.37	-17.36	-31.82	-12.52	-33.9
Poland	Colon and rectum cancer	-164.51	-75.13	-96.58	-85.75	-69.87	-65.7
Portugal	Colon and rectum cancer	-66.74	-99.37	-39.54	-108.42	-27.58	-89.86
Puerto Rico	Colon and rectum cancer	-13.4	-111.9	-7.91	-112.89	-5.52	-111.03
Qatar	Colon and rectum cancer	-1.65	-393.89	-0.99	-421.19	-0.72	-393.6
Republic of Korea	Colon and rectum cancer	-259.88	-240.46	-156.43	-277.08	-103.55	-200.6
Republic of Moldova	Colon and rectum cancer	-11.11	-54.18	-6.29	-60.9	-5.13	-50.44
Romania	Colon and rectum cancer	-88.46	-97.11	-51.12	-101.26	-38.75	-95.41
Russian Federation	Colon and rectum cancer	-440.99	-55.87	-217.85	-62.13	-239.06	-54.5
Rwanda	Colon and rectum cancer	-3.51	-39.88	-1.77	-43.22	-1.76	-37.63
Saint Kitts and Nevis	Colon and rectum cancer	-0.15	-104.76	-0.08	-121.89	-0.07	-91.42
Saint Lucia	Colon and rectum cancer	-0.22	-95.46	-0.12	-104.18	-0.11	-88.8
Saint Vincent and the Grenadines	Colon and rectum cancer	-0.09	-50.62	-0.05	-54.94	-0.05	-48.35
Samoa	Colon and rectum cancer	-0.07	-37.06	-0.03	-34.24	-0.04	-38.74
San Marino	Colon and rectum cancer	-0.07	-41.11	-0.04	-43.86	-0.03	-38.05
Sao Tome and Principe	Colon and rectum cancer	-0.05	-46.01	-0.04	-48.84	-0.01	-40.68

Location	Cause	Attributable number (Both thousands)	Attributable proportion (Both %)	Attributable number (Male thousands)	Attributable proportion (Male %)	Attributable number (Female thousands)	Attributable proportion (Female %)
Saudi Arabia	Colon and rectum cancer	-25.22	-251.84	-14.95	-231.72	-9.93	-278.78
Senegal	Colon and rectum cancer	-1.44	-33.64	-0.8	-32.73	-0.62	-34.08
Serbia	Colon and rectum cancer	-40.4	-66	-24.34	-69.64	-15.43	-58.75
Seychelles	Colon and rectum cancer	-0.16	-70.64	-0.09	-76.94	-0.07	-65.26
Sierra Leone	Colon and rectum cancer	-0.48	-22.57	-0.29	-22.36	-0.18	-21.32
Singapore	Colon and rectum cancer	-18.02	-149.3	-9.83	-154.86	-8.16	-142.54
Slovakia	Colon and rectum cancer	-27.62	-70.61	-16.89	-73.63	-11.02	-68.11
Slovenia	Colon and rectum cancer	-10.1	-83.22	-6.24	-96.34	-4.03	-71.15
Solomon Islands	Colon and rectum cancer	-0.11	-30.61	-0.06	-27.93	-0.05	-32.98
Somalia	Colon and rectum cancer	-0.77	-10.87	-0.38	-11.24	-0.38	-10.21
South Africa	Colon and rectum cancer	-13.15	-28.53	-7.29	-30.28	-5.86	-26.6
South Sudan	Colon and rectum cancer	-0.95	-15.1	-0.56	-14.3	-0.37	-15.53
Spain	Colon and rectum cancer	-180.13	-76.88	-107.29	-84	-75.49	-70.84
Sri Lanka	Colon and rectum cancer	-15.3	-112.19	-7.79	-106.27	-7.48	-118.6
Sudan	Colon and rectum cancer	-13.84	-82.63	-5.84	-75.22	-7.8	-86.76
Suriname	Colon and rectum cancer	-0.45	-57.32	-0.24	-58.4	-0.22	-57.22
Sweden	Colon and rectum cancer	-26.27	-44.44	-14.75	-48.79	-11.64	-40.3

Location	Cause	Attributable number (Both thousands)	Attributable proportion (Both %)	Attributable number (Male thousands)	Attributable proportion (Male %)	Attributable number (Female thousands)	Attributable proportion (Female %)
Switzerland	Colon and rectum cancer	-16.52	-48.94	-9.89	-55.82	-7.11	-44.38
Syrian Arab Republic	Colon and rectum cancer	-10.82	-107.34	-5.08	-99.05	-5.52	-111.43
Taiwan (Province of China)	Colon and rectum cancer	-102.08	-141.71	-63.14	-141.54	-39.4	-143.64
Tajikistan	Colon and rectum cancer	-0.91	-14.3	-0.52	-15.02	-0.4	-13.9
Thailand	Colon and rectum cancer	-190.24	-154.76	-114.91	-170.44	-76.94	-138.62
Timor-Leste	Colon and rectum cancer	-0.33	-46.68	-0.13	-42.29	-0.2	-50.13
Togo	Colon and rectum cancer	-0.47	-32.4	-0.22	-30.32	-0.25	-34.26
Tokelau	Colon and rectum cancer	0	-39.42	0	-38.84	0	-39.97
Tonga	Colon and rectum cancer	-0.03	-27.34	-0.01	-26.89	-0.02	-27.73
Trinidad and Tobago	Colon and rectum cancer	-2.59	-88.06	-1.39	-90.54	-1.22	-86.71
Tunisia	Colon and rectum cancer	-10.27	-111.78	-5.21	-105.66	-5.14	-120.84
Turkey	Colon and rectum cancer	-222.33	-143.77	-122.58	-145.93	-100.32	-142
Turkmenistan	Colon and rectum cancer	-1.16	-30.98	-0.62	-31.79	-0.54	-30.5
Tuvalu	Colon and rectum cancer	-0.01	-39.13	0	-42.23	0	-37.23
Uganda	Colon and rectum cancer	-5.3	-32.6	-3	-32.29	-2.23	-32.04
Ukraine	Colon and rectum cancer	-62.22	-16.66	-31.08	-17.83	-35.89	-18.01
United Arab Emirates	Colon and rectum cancer	-4.97	-192.92	-2.76	-183.81	-2.17	-202.78

Location	Cause	Attributable number (Both thousands)	Attributable proportion (Both %)	Attributable number (Male thousands)	Attributable proportion (Male %)	Attributable number (Female thousands)	Attributable proportion (Female %)
United Kingdom	Colon and rectum cancer	-249.88	-53.64	-141.04	-58.39	-110.91	-49.46
United Republic of Tanzania	Colon and rectum cancer	-7	-28.55	-3.72	-30.41	-3.35	-27.27
United States of America	Colon and rectum cancer	-498.53	-34.14	-277.4	-36.3	-226.22	-32.51
United States Virgin Islands	Colon and rectum cancer	-0.21	-49.34	-0.11	-50.41	-0.1	-49.16
Uruguay	Colon and rectum cancer	-11.37	-51.22	-5.92	-53.8	-5.55	-49.56
Uzbekistan	Colon and rectum cancer	-4.58	-21.26	-2.32	-21.55	-2.34	-21.72
Vanuatu	Colon and rectum cancer	-0.03	-19.02	-0.02	-17.34	-0.01	-20.19
Venezuela (Bolivarian Republic of)	Colon and rectum cancer	-23	-103.98	-12.25	-114.67	-10.25	-89.59
Viet Nam	Colon and rectum cancer	-129.43	-148.59	-77.75	-160.23	-52.22	-135.37
Yemen	Colon and rectum cancer	-6.32	-68.35	-2.66	-57.96	-3.37	-72.5
Zambia	Colon and rectum cancer	-3.15	-40.49	-1.73	-43.68	-1.47	-38.49
Zimbabwe	Colon and rectum cancer	1.04	10.46	0.52	10.34	0.6	12.32

Table S18. Absolute (the number) and relative contribution (the proportion) associated with disease severity changes for oesophageal cancer (EC), by sex at global, SDI regional, GBD regional level between 1990 and 2021.

Location	Cause	Attributable number (Both thousands)	Attributable proportion (Both %)	Attributable number (Male thousands)	Attributable proportion (Male %)	Attributable number (Female thousands)	Attributable proportion (Female %)
Global	Esophageal cancer	-3498.15	-35.87	-2426.03	-34.49	-1111.37	-40.88
High SDI	Esophageal cancer	-663.23	-50.02	-537.13	-51.23	-134.49	-48.48
High-middle SDI	Esophageal cancer	-1371.81	-44.02	-955.67	-40.33	-444.29	-59.49
Middle SDI	Esophageal cancer	-1397.01	-34.21	-883.47	-30.31	-544.35	-46.55
Low-middle SDI	Esophageal cancer	-57.74	-7.57	-31.36	-7.1	-27.54	-8.57
Low SDI	Esophageal cancer	-25.56	-5.55	-13.77	-5.35	-12.05	-5.94
High-income Asia Pacific	Esophageal cancer	-179.6	-70.23	-163.88	-75.09	-20.8	-55.52
High-income North America	Esophageal cancer	-79.93	-25.58	-62.92	-26.07	-22.97	-32.28
Western Europe	Esophageal cancer	-328.62	-50.47	-252.26	-49.53	-77.19	-54.4
Australasia	Esophageal cancer	-7.85	-34.04	-7.16	-44.38	-2.14	-30.87
Eastern Europe	Esophageal cancer	-44.21	-12.97	-35.48	-13.13	-9.48	-13.39
Central Europe	Esophageal cancer	-15.96	-13.04	-12	-12.06	-4.32	-18.8
Southern Latin America	Esophageal cancer	-10.79	-13.1	-6.95	-11.55	-3.64	-16.42
East Asia	Esophageal cancer	-2687	-45.22	-1798.66	-40.85	-964.75	-62.69
Central Asia	Esophageal cancer	-3.21	-1.92	-1.73	-1.77	-1.65	-2.36
North Africa and Middle East	Esophageal cancer	-46.97	-37.61	-22.52	-33.55	-25.32	-43.83
Andean Latin America	Esophageal cancer	-1.43	-13.95	-0.88	-12.52	-0.55	-17.45

Location	Cause	Attributable number (Both thousands)	Attributable proportion (Both %)	Attributable number (Male thousands)	Attributable proportion (Male %)	Attributable number (Female thousands)	Attributable proportion (Female %)
Southeast Asia	Esophageal cancer	-50.9	-24.64	-33.25	-26.25	-18.96	-23.72
Tropical Latin America	Esophageal cancer	-20.41	-11.48	-15.22	-10.9	-5.41	-14.2
Southern Sub-Saharan Africa	Esophageal cancer	0.73	0.78	0.58	0.94	0.16	0.5
Caribbean	Esophageal cancer	-2.94	-11.21	-2.37	-12.53	-0.71	-9.65
Central Latin America	Esophageal cancer	-6.2	-11.35	-3.85	-10.59	-2.68	-14.69
South Asia	Esophageal cancer	-62.02	-8.68	-31.67	-7.92	-31.09	-9.87
Central Sub-Saharan Africa	Esophageal cancer	-3.88	-5.31	-2.4	-5.2	-1.41	-5.23
Oceania	Esophageal cancer	-0.11	-5.77	-0.08	-5.9	-0.03	-5.86
Western Sub-Saharan Africa	Esophageal cancer	-3.64	-5.49	-2.55	-5.86	-1.31	-5.76
Eastern Sub-Saharan Africa	Esophageal cancer	-17.88	-5.87	-9.31	-5.46	-8.39	-6.24
Afghanistan	Esophageal cancer	-1.39	-7.15	-0.41	-5.54	-0.91	-7.67
Albania	Esophageal cancer	-0.13	-11.95	-0.09	-12.21	-0.04	-12.55
Algeria	Esophageal cancer	-0.91	-40.49	-0.45	-32.64	-0.48	-54.08
American Samoa	Esophageal cancer	0	-8.42	0	-8.81	0	-8.51
Andorra	Esophageal cancer	-0.01	-37.58	-0.01	-36.05	0	-49.19
Angola	Esophageal cancer	-1.12	-7.65	-0.71	-7.18	-0.37	-8
Antigua and Barbuda	Esophageal cancer	0	-8.67	0	-9.06	0	-8.91
Argentina	Esophageal cancer	-5.56	-9.78	-3.77	-8.79	-1.62	-11.57
Armenia	Esophageal cancer	-0.1	-4.64	-0.06	-4.52	-0.04	-5.73

Location	Cause	Attributable number (Both thousands)	Attributable proportion (Both %)	Attributable number (Male thousands)	Attributable proportion (Male %)	Attributable number (Female thousands)	Attributable proportion (Female %)
Australia	Esophageal cancer	-5.58	-29.49	-5.81	-43.51	-1.21	-21.71
Austria	Esophageal cancer	-3.26	-45.41	-2.47	-42.06	-0.8	-60.89
Azerbaijan	Esophageal cancer	-0.39	-3.25	-0.25	-3.43	-0.17	-3.68
Bahamas	Esophageal cancer	-0.02	-6.72	-0.02	-7.47	0	-6.87
Bahrain	Esophageal cancer	-0.12	-72.51	-0.07	-63.54	-0.05	-90.64
Bangladesh	Esophageal cancer	-8.83	-11.83	-4.41	-10.26	-4.38	-13.86
Barbados	Esophageal cancer	-0.03	-8.76	-0.03	-9.14	-0.01	-9.47
Belarus	Esophageal cancer	-2.21	-20.71	-1.71	-18.35	-0.58	-42.38
Belgium	Esophageal cancer	-6.04	-41.98	-4.51	-41.02	-1.58	-46.68
Belize	Esophageal cancer	0	-5.68	0	-6.46	0	-7.6
Benin	Esophageal cancer	-0.11	-6.17	-0.08	-7.1	-0.04	-6.23
Bermuda	Esophageal cancer	-0.03	-30.76	-0.03	-31.29	-0.01	-35.53
Bhutan	Esophageal cancer	-0.03	-8.63	-0.02	-7.54	-0.02	-10.29
Bolivia (Plurinational State of)	Esophageal cancer	-0.19	-7.91	-0.11	-7.51	-0.08	-9.08
Bosnia and Herzegovina	Esophageal cancer	-0.24	-8.45	-0.18	-8.8	-0.08	-9.27
Botswana	Esophageal cancer	-0.03	-1.67	-0.03	-1.96	-0.01	-1.99
Brazil	Esophageal cancer	-20.25	-11.52	-15.08	-10.91	-5.37	-14.24
Brunei Darussalam	Esophageal cancer	-0.03	-41.39	-0.02	-41.34	-0.01	-41.54
Bulgaria	Esophageal cancer	-0.49	-5.67	-0.4	-6.13	-0.1	-5.01

Location	Cause	Attributable number (Both thousands)	Attributable proportion (Both %)	Attributable number (Male thousands)	Attributable proportion (Male %)	Attributable number (Female thousands)	Attributable proportion (Female %)
Burkina Faso	Esophageal cancer	-0.21	-5.3	-0.15	-6.51	-0.09	-5.49
Burundi	Esophageal cancer	-0.74	-5.55	-0.46	-6.32	-0.29	-4.78
Cabo Verde	Esophageal cancer	-0.03	-5.82	-0.03	-10.07	-0.01	-7.53
Cambodia	Esophageal cancer	-0.76	-12.72	-0.39	-11.55	-0.39	-14.87
Cameroon	Esophageal cancer	-0.19	-4.09	-0.16	-5.17	-0.07	-4.81
Canada	Esophageal cancer	-13.17	-49.39	-11.02	-56.12	-3.23	-45.97
Central African Republic	Esophageal cancer	-0.11	-2.22	-0.09	-2.56	-0.02	-1.1
Chad	Esophageal cancer	-0.05	-2.31	-0.04	-3.59	-0.02	-2.04
Chile	Esophageal cancer	-4.36	-24.94	-2.54	-22.39	-1.85	-30.08
China	Esophageal cancer	-2643.38	-45.17	-1759.42	-40.63	-958.33	-62.96
Colombia	Esophageal cancer	-3.01	-16.33	-1.73	-14.48	-1.36	-20.78
Comoros	Esophageal cancer	-0.05	-5.19	-0.03	-4.95	-0.03	-5.28
Congo	Esophageal cancer	-0.38	-7.4	-0.28	-8.31	-0.11	-5.92
Cook Islands	Esophageal cancer	0	-25.81	0	-26.61	0	-27.54
Costa Rica	Esophageal cancer	-0.16	-15.1	-0.11	-14.45	-0.05	-19.02
Coted'Ivoire	Esophageal cancer	-0.08	-5.34	-0.06	-5.67	-0.03	-5.1
Croatia	Esophageal cancer	-1.09	-16.88	-0.78	-14.15	-0.34	-36.4
Cuba	Esophageal cancer	-1.53	-16.3	-1.3	-18.7	-0.44	-17.7
Cyprus	Esophageal cancer	-0.2	-83.92	-0.15	-84.36	-0.06	-89.31

Location	Cause	Attributable number (Both thousands)	Attributable proportion (Both %)	Attributable number (Male thousands)	Attributable proportion (Male %)	Attributable number (Female thousands)	Attributable proportion (Female %)
Czechia	Esophageal cancer	-3.82	-37.01	-2.57	-30.2	-1.19	-66.25
Democratic People's Republic of	Esophageal cancer	-7.62	-15.14	-4.77	-13.09	-3.21	-23.21
Democratic Republic of the Congo	Esophageal cancer	-2.03	-4.46	-1.13	-4.08	-0.85	-4.73
Denmark	Esophageal cancer	-3.9	-43.47	-2.6	-38.6	-1.26	-56.37
Djibouti	Esophageal cancer	-0.03	-4.96	-0.02	-5.49	-0.01	-5.16
Dominica	Esophageal cancer	0	-2.32	0	-3.61	0	-2.42
Dominican Republic	Esophageal cancer	-0.1	-5.11	-0.06	-5.02	-0.05	-7.72
Ecuador	Esophageal cancer	-0.27	-9.91	-0.17	-8.6	-0.1	-13.36
Egypt	Esophageal cancer	-1.87	-19.86	-0.92	-15.86	-0.97	-26.82
El Salvador	Esophageal cancer	-0.18	-12.44	-0.11	-11.34	-0.08	-15.91
Equatorial Guinea	Esophageal cancer	-0.11	-13.18	-0.08	-14.18	-0.03	-12.97
Eritrea	Esophageal cancer	-0.63	-7.76	-0.35	-8.14	-0.26	-6.98
Estonia	Esophageal cancer	-0.48	-25.62	-0.36	-23.33	-0.15	-46.59
Eswatini	Esophageal cancer	0.02	1.32	0.01	1.23	0	0.97
Ethiopia	Esophageal cancer	-5.45	-9.38	-2.94	-9.25	-2.45	-9.31
Fiji	Esophageal cancer	-0.01	-6.01	-0.01	-6.09	0	-6.49
Finland	Esophageal cancer	-2.92	-53.49	-1.99	-59.66	-1.21	-57.32
France	Esophageal cancer	-110.33	-69.02	-91.16	-64.61	-17.44	-92.95
Gabon	Esophageal cancer	-0.11	-5.44	-0.08	-6.05	-0.03	-5.39

Location	Cause	Attributable number (Both thousands)	Attributable proportion (Both %)	Attributable number (Male thousands)	Attributable proportion (Male %)	Attributable number (Female thousands)	Attributable proportion (Female %)
Gambia	Esophageal cancer	-0.01	-3.38	0	-3.43	0	-3.42
Georgia	Esophageal cancer	-0.06	-1.7	-0.04	-1.71	-0.02	-2.42
Germany	Esophageal cancer	-75.85	-70.86	-59.14	-67.98	-15.83	-79.03
Ghana	Esophageal cancer	-0.29	-6.5	-0.13	-4.9	-0.17	-8.92
Greece	Esophageal cancer	-1.46	-19.64	-1.12	-20.72	-0.42	-20.66
Greenland	Esophageal cancer	-0.02	-12.76	-0.02	-12.95	-0.01	-15.26
Grenada	Esophageal cancer	-0.01	-5.06	0	-5.6	0	-5.42
Guam	Esophageal cancer	-0.01	-10.73	0	-11.01	0	-15.19
Guatemala	Esophageal cancer	-0.23	-9.88	-0.13	-9.52	-0.11	-11.4
Guinea	Esophageal cancer	-0.02	-1.67	-0.01	-1.18	-0.01	-2.79
Guinea-Bissau	Esophageal cancer	-0.03	-5.27	-0.02	-6.03	-0.01	-4.37
Guyana	Esophageal cancer	-0.01	-4.55	-0.01	-5.2	0	-4.78
Haiti	Esophageal cancer	-0.18	-3.67	-0.11	-3.61	-0.07	-3.85
Honduras	Esophageal cancer	-0.02	-3.97	-0.01	-3.35	-0.01	-5.46
Hungary	Esophageal cancer	-1.95	-10.91	-1.62	-10.33	-0.31	-14.44
Iceland	Esophageal cancer	-0.16	-52.95	-0.11	-53.17	-0.05	-56.81
India	Esophageal cancer	-50.29	-9.56	-25.96	-8.61	-25.01	-11.15
Indonesia	Esophageal cancer	-5.91	-10.49	-2.39	-10.48	-3.98	-11.85
Iran (Islamic Republic of)	Esophageal cancer	-18.24	-52.23	-9.13	-44.94	-9.35	-64.05

Location	Cause	Attributable number (Both thousands)	Attributable proportion (Both %)	Attributable number (Male thousands)	Attributable proportion (Male %)	Attributable number (Female thousands)	Attributable proportion (Female %)
Iraq	Esophageal cancer	-0.98	-36.52	-0.45	-28.82	-0.54	-48.6
Ireland	Esophageal cancer	-4.25	-59.52	-2.75	-61.39	-1.61	-60.63
Israel	Esophageal cancer	-1.03	-47.49	-0.64	-45.13	-0.41	-53.71
Italy	Esophageal cancer	-20.11	-29.01	-12.68	-23.12	-6.66	-46.08
Jamaica	Esophageal cancer	-0.06	-4.92	-0.05	-5.75	-0.02	-5.82
Japan	Esophageal cancer	-127.53	-62.34	-117.07	-67.51	-14.6	-46.84
Jordan	Esophageal cancer	-0.29	-69.57	-0.16	-64.75	-0.15	-86.36
Kazakhstan	Esophageal cancer	-2.56	-3.92	-1.38	-3.69	-1.28	-4.59
Kenya	Esophageal cancer	-0.86	-4	-0.29	-3.18	-0.68	-5.47
Kiribati	Esophageal cancer	0	-4.06	0	-4.14	0	-3.87
Kuwait	Esophageal cancer	-0.19	-62.92	-0.12	-63.84	-0.09	-78.22
Kyrgyzstan	Esophageal cancer	-0.25	-3.58	-0.16	-3.46	-0.1	-4.08
Lao People's Democratic Republic	Esophageal cancer	-0.25	-8.72	-0.15	-8.47	-0.11	-9.52
Latvia	Esophageal cancer	-0.3	-9.76	-0.18	-6.82	-0.15	-30.92
Lebanon	Esophageal cancer	-0.43	-57.21	-0.24	-49.71	-0.19	-74.71
Lesotho	Esophageal cancer	0.17	7.03	0.12	7.54	0.05	5.74
Liberia	Esophageal cancer	-0.08	-8.18	-0.06	-8.63	-0.03	-8.23
Libya	Esophageal cancer	-0.21	-29.18	-0.17	-27.24	-0.06	-42.99
Lithuania	Esophageal cancer	-0.5	-12.48	-0.39	-11.25	-0.16	-29

Location	Cause	Attributable number (Both thousands)	Attributable proportion (Both %)	Attributable number (Male thousands)	Attributable proportion (Male %)	Attributable number (Female thousands)	Attributable proportion (Female %)
Luxembourg	Esophageal cancer	-0.32	-49.92	-0.25	-48.91	-0.08	-56.28
Madagascar	Esophageal cancer	-0.59	-2.66	-0.29	-2.58	-0.26	-2.37
Malawi	Esophageal cancer	-1.14	-4.59	-0.6	-4.54	-0.66	-5.69
Malaysia	Esophageal cancer	-1.91	-28.95	-1.15	-27.85	-0.84	-33.87
Maldives	Esophageal cancer	-0.03	-37.82	-0.02	-37.15	-0.02	-41.7
Mali	Esophageal cancer	-0.13	-4.53	-0.07	-4.34	-0.06	-4.98
Malta	Esophageal cancer	-0.14	-46.1	-0.1	-42.84	-0.04	-55.66
Marshall Islands	Esophageal cancer	0	-6.02	0	-6.69	0	-4.4
Mauritania	Esophageal cancer	-0.11	-11.1	-0.07	-11.94	-0.04	-10.81
Mauritius	Esophageal cancer	-0.15	-20.39	-0.11	-24.81	-0.07	-21.53
Mexico	Esophageal cancer	-2.26	-9.59	-1.5	-9.43	-0.92	-12.15
Micronesia (Federated States of)	Esophageal cancer	0	-5.15	0	-5.2	0	-6.01
Monaco	Esophageal cancer	-0.02	-33.64	-0.01	-32.93	-0.01	-34.8
Mongolia	Esophageal cancer	-0.35	-5.42	-0.15	-5.14	-0.23	-6.41
Montenegro	Esophageal cancer	-0.02	-5.3	-0.02	-5.24	0	-7.18
Morocco	Esophageal cancer	-0.67	-21.1	-0.24	-15.8	-0.44	-26.91
Mozambique	Esophageal cancer	0.11	0.96	0.13	1.44	-0.04	-1.32
Myanmar	Esophageal cancer	-2.95	-10.28	-1.64	-9.91	-1.38	-11.36
Namibia	Esophageal cancer	-0.02	-4.24	-0.02	-4.68	-0.01	-5.43

Location	Cause	Attributable number (Both thousands)	Attributable proportion (Both %)	Attributable number (Male thousands)	Attributable proportion (Male %)	Attributable number (Female thousands)	Attributable proportion (Female %)
Nauru	Esophageal cancer	0	-4.1	0	-3.8	0	-5.43
Nepal	Esophageal cancer	-1.12	-7.9	-0.49	-6.81	-0.66	-9.48
Netherlands	Esophageal cancer	-14.21	-64.97	-11.51	-74.99	-3.27	-50.18
New Zealand	Esophageal cancer	-2.31	-55.92	-1.45	-52.45	-0.92	-67.69
Nicaragua	Esophageal cancer	-0.05	-11.22	-0.03	-10.56	-0.02	-14.46
Niger	Esophageal cancer	-0.14	-5.94	-0.09	-6.45	-0.05	-5.48
Nigeria	Esophageal cancer	-1.83	-5.53	-1.33	-5.79	-0.57	-5.6
Niue	Esophageal cancer	0	-6.84	0	-7.31	0	-7.49
North Macedonia	Esophageal cancer	-0.07	-8.73	-0.05	-8.72	-0.02	-9.71
Northern Mariana Islands	Esophageal cancer	0	-21.07	0	-22.99	0	-24.34
Norway	Esophageal cancer	-2.14	-56.78	-1.27	-45.54	-0.85	-87.41
Oman	Esophageal cancer	-0.2	-55.32	-0.12	-53.13	-0.09	-67.48
Pakistan	Esophageal cancer	-2.54	-2.55	-1.09	-2.27	-1.52	-2.92
Palau	Esophageal cancer	0	-12.13	0	-12.48	0	-13.5
Palestine	Esophageal cancer	-0.09	-30.95	-0.05	-27.04	-0.05	-39.87
Panama	Esophageal cancer	-0.09	-13.69	-0.06	-13.25	-0.03	-17.09
Papua New Guinea	Esophageal cancer	-0.05	-5.14	-0.04	-5.1	-0.02	-5.67
Paraguay	Esophageal cancer	-0.15	-7.78	-0.12	-7.95	-0.04	-10.26
Peru	Esophageal cancer	-0.98	-18.9	-0.61	-16.96	-0.37	-23.73

Location	Cause	Attributable number (Both thousands)	Attributable proportion (Both %)	Attributable number (Male thousands)	Attributable proportion (Male %)	Attributable number (Female thousands)	Attributable proportion (Female %)
Philippines	Esophageal cancer	-0.73	-5.59	-0.46	-4.91	-0.29	-7.73
Poland	Esophageal cancer	-3.26	-7.61	-2.42	-7.15	-0.91	-10.11
Portugal	Esophageal cancer	-4.37	-27.4	-3.1	-24.58	-1.42	-42.74
Puerto Rico	Esophageal cancer	-0.85	-15.64	-0.66	-15.05	-0.21	-19.76
Qatar	Esophageal cancer	-0.19	-141.35	-0.13	-141.73	-0.07	-162.41
Republic of Korea	Esophageal cancer	-66.55	-136.53	-60.83	-141.61	-6.96	-120.32
Republic of Moldova	Esophageal cancer	-0.35	-11.04	-0.27	-10.27	-0.13	-22.86
Romania	Esophageal cancer	-1.53	-12.37	-1.26	-12.98	-0.33	-12.62
Russian Federation	Esophageal cancer	-36.45	-14.43	-29.37	-15.14	-7.57	-12.9
Rwanda	Esophageal cancer	-1.45	-7.73	-0.72	-7.29	-0.7	-7.9
Saint Kitts and Nevis	Esophageal cancer	0	-9.35	0	-10.32	0	-11.17
Saint Lucia	Esophageal cancer	-0.01	-10.38	-0.01	-11.72	0	-10.82
Saint Vincent and the Grenadines	Esophageal cancer	0	-4.45	0	-5.39	0	-5.25
Samoa	Esophageal cancer	0	-9.17	0	-7.67	0	-10.61
San Marino	Esophageal cancer	0	-28.68	0	-28.41	0	-30.93
Sao Tome and Principe	Esophageal cancer	0	-7.36	0	-8.81	0	-8.09
Saudi Arabia	Esophageal cancer	-1.86	-54.45	-0.82	-47.6	-1.13	-66.84
Senegal	Esophageal cancer	-0.19	-6.86	-0.14	-7.82	-0.06	-6.42
Serbia	Esophageal cancer	-1	-13.1	-0.85	-13.61	-0.17	-11.69

Location	Cause	Attributable number (Both thousands)	Attributable proportion (Both %)	Attributable number (Male thousands)	Attributable proportion (Male %)	Attributable number (Female thousands)	Attributable proportion (Female %)
Seychelles	Esophageal cancer	-0.01	-16.39	-0.01	-16.48	0	-19.06
Sierra Leone	Esophageal cancer	-0.09	-5.52	-0.07	-6.35	-0.02	-4.19
Singapore	Esophageal cancer	-3.14	-134.33	-2.56	-138.44	-0.62	-127.12
Slovakia	Esophageal cancer	-0.8	-11.46	-0.71	-11.36	-0.1	-12.95
Slovenia	Esophageal cancer	-0.86	-37.12	-0.76	-38.78	-0.09	-26.41
Solomon Islands	Esophageal cancer	-0.01	-6.9	-0.01	-6.56	0	-7.89
Somalia	Esophageal cancer	-0.5	-3.05	-0.27	-2.84	-0.17	-2.34
South Africa	Esophageal cancer	0.12	0.17	0.29	0.6	-0.26	-0.99
South Sudan	Esophageal cancer	-0.25	-1.92	-0.14	-1.58	-0.07	-1.57
Spain	Esophageal cancer	-22.01	-39.6	-17.53	-36.37	-4.16	-56.3
Sri Lanka	Esophageal cancer	-5.61	-32.65	-2.65	-28.46	-3.03	-38.4
Sudan	Esophageal cancer	-1.76	-13.84	-0.83	-13.07	-1.06	-16.64
Suriname	Esophageal cancer	-0.01	-4.34	0	-4.68	0	-5.16
Sweden	Esophageal cancer	-2.45	-28.89	-1.64	-28.22	-0.92	-34.34
Switzerland	Esophageal cancer	-4.29	-40.85	-3.41	-40.81	-0.91	-42.22
Syrian Arab Republic	Esophageal cancer	-0.46	-37.11	-0.25	-33.08	-0.23	-46.89
Taiwan (Province of China)	Esophageal cancer	-28.41	-71.42	-24.77	-67.12	-3.45	-119.87
Tajikistan	Esophageal cancer	0.01	0.12	-0.01	-0.14	0.01	0.16
Thailand	Esophageal cancer	-21.25	-41.26	-15.83	-40.3	-5.91	-48.39

Location	Cause	Attributable number (Both thousands)	Attributable proportion (Both %)	Attributable number (Male thousands)	Attributable proportion (Male %)	Attributable number (Female thousands)	Attributable proportion (Female %)
Timor-Leste	Esophageal cancer	-0.02	-7.93	-0.01	-6.27	-0.01	-10.23
Togo	Esophageal cancer	-0.06	-5.1	-0.04	-5.48	-0.03	-6.82
Tokelau	Esophageal cancer	0	-8.2	0	-7.9	0	-9.86
Tonga	Esophageal cancer	0	-6.13	0	-6.32	0	-7.46
Trinidad and Tobago	Esophageal cancer	-0.05	-8.76	-0.04	-9.31	-0.01	-9.81
Tunisia	Esophageal cancer	-0.38	-44.57	-0.2	-38.11	-0.19	-58.51
Turkey	Esophageal cancer	-12.1	-50.31	-5.7	-42.28	-6.57	-62.15
Turkmenistan	Esophageal cancer	-0.33	-2.1	-0.17	-1.98	-0.19	-2.53
Tuvalu	Esophageal cancer	0	-7.75	0	-8.4	0	-7.95
Uganda	Esophageal cancer	-2.14	-7.97	-1.38	-8.06	-0.71	-7.32
Ukraine	Esophageal cancer	-4.24	-6.47	-3.79	-6.68	-0.66	-7.39
United Arab Emirates	Esophageal cancer	-0.2	-60.57	-0.12	-56.75	-0.09	-81.71
United Kingdom	Esophageal cancer	-41.06	-28.54	-28.45	-30.49	-15.3	-30.26
United Republic of Tanzania	Esophageal cancer	-2.31	-4.49	-1.17	-4.07	-1.06	-4.66
United States of America	Esophageal cancer	-65.05	-22.77	-50.55	-22.82	-19.49	-30.41
United States Virgin Islands	Esophageal cancer	0	-4.42	0	-4.77	0	-7.96
Uruguay	Esophageal cancer	-0.88	-11.08	-0.61	-10.23	-0.27	-13.07
Uzbekistan	Esophageal cancer	0.05	0.11	0.21	0.75	-0.08	-0.41
Vanuatu	Esophageal cancer	0	-4.5	0	-4.46	0	-4.99

Location	Cause	Attributable number (Both thousands)	Attributable proportion (Both %)	Attributable number (Male thousands)	Attributable proportion (Male %)	Attributable number (Female thousands)	Attributable proportion (Female %)
Venezuela (Bolivarian Republic of)	Esophageal cancer	-0.59	-9.45	-0.38	-8.72	-0.23	-12.55
Viet Nam	Esophageal cancer	-7.14	-31.08	-6.17	-31.79	-1.21	-33.87
Yemen	Esophageal cancer	-0.82	-11.32	-0.37	-9.85	-0.46	-13.6
Zambia	Esophageal cancer	-0.79	-4.69	-0.33	-3.4	-0.45	-6.25
Zimbabwe	Esophageal cancer	0.47	3.27	0.31	3.07	0.26	6.08

Table S19. Absolute (the number) and relative contribution (the proportion) associated with disease severity changes for lip and oral cavity cancer (LOC) by sex at global, SDI regional, GBD regional level between 1990 and 2021.

Location	Cause	Attributable number (Both thousands)	Attributable proportion (Both %)	Attributable number (Male thousands)	Attributable proportion (Male %)	Attributable number (Female thousands)	Attributable proportion (Female %)
Global	Lip and oral cavity cancer	-1145.13	-39.35	-707.16	-34.45	-413.81	-48.27
High SDI	Lip and oral cavity cancer	-253.26	-44.76	-178.66	-42.18	-70.46	-49.54
High-middle SDI	Lip and oral cavity cancer	-381.19	-66.62	-244.71	-54.37	-118.51	-97.07
Middle SDI	Lip and oral cavity cancer	-735.12	-104.29	-531.19	-106.31	-213.3	-103.97
Low-middle SDI	Lip and oral cavity cancer	-501.33	-59.97	-279.17	-52.25	-209.33	-69.4
Low SDI	Lip and oral cavity cancer	-106.91	-46.91	-58.27	-40.94	-46.4	-54.22
High-income Asia Pacific	Lip and oral cavity cancer	-39.03	-73.37	-26.47	-72.38	-13.37	-80.46
High-income North America	Lip and oral cavity cancer	-62.34	-35.45	-44.14	-36.49	-18.86	-34.37
Western Europe	Lip and oral cavity cancer	-192.08	-55.49	-131.59	-47.27	-53.87	-79.49
Australasia	Lip and oral cavity cancer	-6.58	-59.28	-4.51	-56.9	-2.07	-65.23
Eastern Europe	Lip and oral cavity cancer	-113.84	-52.16	-73.44	-40.72	-25.2	-66.55
Central Europe	Lip and oral cavity cancer	-74.19	-60.85	-55.87	-55	-15.83	-77.78
Southern Latin America	Lip and oral cavity cancer	-11.69	-53.54	-7.74	-45.24	-3.55	-75.36
East Asia	Lip and oral cavity cancer	-577.4	-185.24	-460.08	-213.24	-151.16	-157.54
Central Asia	Lip and oral cavity cancer	-9.39	-34.98	-5.06	-25.89	-3.02	-41.21
North Africa and Middle East	Lip and oral cavity cancer	-42.1	-118.8	-24.24	-108.37	-17.24	-131.91
Andean Latin America	Lip and oral cavity cancer	-6.95	-121.09	-3.2	-100.27	-3.55	-139.65
Southeast Asia	Lip and oral cavity cancer	-141.17	-80.02	-97.76	-87.49	-50.41	-77.95

Location	Cause	Attributable number (Both thousands)	Attributable proportion (Both %)	Attributable number (Male thousands)	Attributable proportion (Male %)	Attributable number (Female thousands)	Attributable proportion (Female %)
Tropical Latin America	Lip and oral cavity cancer	-49.09	-71.01	-34.88	-63.22	-12.6	-90.32
Southern Sub-Saharan Africa	Lip and oral cavity cancer	-5.1	-21.11	-2.97	-16.56	-1.58	-25.17
Caribbean	Lip and oral cavity cancer	-6.17	-36.02	-4.6	-37.05	-1.69	-35.94
Central Latin America	Lip and oral cavity cancer	-22.97	-82.42	-12.18	-71.59	-10.26	-94.46
South Asia	Lip and oral cavity cancer	-838.26	-71.59	-494.82	-63.7	-327.13	-83.02
Central Sub-Saharan Africa	Lip and oral cavity cancer	-5.04	-44.22	-2.73	-39.69	-2.31	-51.13
Oceania	Lip and oral cavity cancer	-0.18	-13.47	-0.16	-17.87	-0.03	-8.25
Western Sub-Saharan Africa	Lip and oral cavity cancer	-9.1	-42.05	-3.12	-30.52	-5.59	-48.96
Eastern Sub-Saharan Africa	Lip and oral cavity cancer	-26.78	-43.26	-14.73	-37.15	-11.69	-52.51
Afghanistan	Lip and oral cavity cancer	-0.65	-42.17	-0.21	-32.39	-0.42	-46.51
Albania	Lip and oral cavity cancer	-0.88	-92.08	-0.6	-87.51	-0.27	-99.41
Algeria	Lip and oral cavity cancer	-3.18	-102.29	-2.13	-92.46	-0.98	-122.38
American Samoa	Lip and oral cavity cancer	0	-12.81	0	-19.34	0	-11.44
Andorra	Lip and oral cavity cancer	-0.01	-34.5	-0.01	-33.6	0	-62.66
Angola	Lip and oral cavity cancer	-1.61	-74.27	-0.89	-63.84	-0.68	-88.34
Antigua and Barbuda	Lip and oral cavity cancer	-0.01	-35.78	-0.01	-33.86	0	-40.72
Argentina	Lip and oral cavity cancer	-7.13	-44.08	-4.68	-36.7	-2.15	-62.81
Armenia	Lip and oral cavity cancer	-0.38	-47.39	-0.28	-44.98	-0.1	-55.64
Australia	Lip and oral cavity cancer	-5.59	-59.93	-3.85	-57.05	-1.73	-66.64
Austria	Lip and oral cavity cancer	-3.98	-60.25	-2.85	-52.61	-0.95	-79.09

Location	Cause	Attributable number (Both thousands)	Attributable proportion (Both %)	Attributable number (Male thousands)	Attributable proportion (Male %)	Attributable number (Female thousands)	Attributable proportion (Female %)
Azerbaijan	Lip and oral cavity cancer	-0.53	-47.35	-0.31	-44.32	-0.22	-51.76
Bahamas	Lip and oral cavity cancer	-0.05	-33.76	-0.04	-31.44	-0.01	-40.4
Bahrain	Lip and oral cavity cancer	-0.16	-183.82	-0.13	-174.07	-0.03	-218.1
Bangladesh	Lip and oral cavity cancer	-88.94	-95.54	-56.81	-82.71	-29.22	-119.7
Barbados	Lip and oral cavity cancer	-0.06	-35.14	-0.04	-33.51	-0.02	-39.23
Belarus	Lip and oral cavity cancer	-8.94	-57.6	-6.31	-46.45	-1.55	-80.15
Belgium	Lip and oral cavity cancer	-3.29	-41.93	-2.17	-35.18	-1.06	-63.07
Belize	Lip and oral cavity cancer	-0.01	-40.57	-0.01	-36.47	-0.01	-49.4
Benin	Lip and oral cavity cancer	-0.21	-44.09	-0.08	-38.42	-0.13	-49.78
Bermuda	Lip and oral cavity cancer	-0.04	-70.63	-0.04	-71.93	-0.01	-73.66
Bhutan	Lip and oral cavity cancer	-0.34	-76.85	-0.21	-67.6	-0.13	-92.49
Bolivia (Plurinational State of)	Lip and oral cavity cancer	-1.02	-81.74	-0.45	-69.54	-0.56	-92.11
Bosnia and Herzegovina	Lip and oral cavity cancer	-1.33	-60.68	-1	-58.38	-0.34	-69.2
Botswana	Lip and oral cavity cancer	-0.18	-33.23	-0.12	-28.97	-0.06	-41.92
Brazil	Lip and oral cavity cancer	-48.58	-71.12	-34.49	-63.26	-12.48	-90.56
Brunei Darussalam	Lip and oral cavity cancer	-0.07	-69.56	-0.05	-66.54	-0.02	-71.67
Bulgaria	Lip and oral cavity cancer	-2.45	-42.56	-1.95	-40.74	-0.46	-46.83
Burkina Faso	Lip and oral cavity cancer	-0.28	-25.49	-0.11	-21.65	-0.19	-30.58
Burundi	Lip and oral cavity cancer	-0.55	-22.79	-0.38	-23.62	-0.21	-25.76

Location	Cause	Attributable number (Both thousands)	Attributable proportion (Both %)	Attributable number (Male thousands)	Attributable proportion (Male %)	Attributable number (Female thousands)	Attributable proportion (Female %)
Cabo Verde	Lip and oral cavity cancer	-0.07	-1018.14	-0.04	-991.57	-0.03	-1060.47
Cambodia	Lip and oral cavity cancer	-2.41	-83.22	-1.58	-90.71	-0.98	-85.32
Cameroon	Lip and oral cavity cancer	-0.56	-40.57	-0.22	-35.55	-0.35	-47.3
Canada	Lip and oral cavity cancer	-6.52	-35.55	-4.21	-32.11	-2.23	-42.46
Central African Republic	Lip and oral cavity cancer	-0.05	-7.86	-0.02	-5.87	-0.03	-10.04
Chad	Lip and oral cavity cancer	-0.08	-14.8	-0.03	-12.75	-0.07	-20.71
Chile	Lip and oral cavity cancer	-3.15	-101.74	-2.05	-89.64	-1.05	-130.25
China	Lip and oral cavity cancer	-542.7	-186.18	-423.68	-212.21	-149.52	-162.79
Colombia	Lip and oral cavity cancer	-7.73	-101.82	-3.84	-90.47	-3.77	-112.96
Comoros	Lip and oral cavity cancer	-0.06	-31.88	-0.03	-26.89	-0.03	-37.8
Congo	Lip and oral cavity cancer	-0.4	-59.2	-0.21	-55.41	-0.19	-64.09
Cook Islands	Lip and oral cavity cancer	0	-82.83	0	-72.25	0	-105.03
Costa Rica	Lip and oral cavity cancer	-0.38	-55.51	-0.23	-50.91	-0.15	-63.63
Coted'Ivoire	Lip and oral cavity cancer	-0.93	-42.88	-0.47	-36.97	-0.45	-50.14
Croatia	Lip and oral cavity cancer	-3.76	-53.4	-2.84	-45.79	-0.58	-69.54
Cuba	Lip and oral cavity cancer	-3.28	-45.01	-2.56	-48.65	-0.95	-47.03
Cyprus	Lip and oral cavity cancer	-0.28	-106.41	-0.18	-97.55	-0.11	-128.06
Czechia	Lip and oral cavity cancer	-7.87	-74.9	-5.41	-63.15	-1.88	-97.15
Democratic People's Republic of Korea	Lip and oral cavity cancer	-2.07	-36.55	-1.71	-45.58	-0.72	-37.42

Location	Cause	Attributable number (Both thousands)	Attributable proportion (Both %)	Attributable number (Male thousands)	Attributable proportion (Male %)	Attributable number (Female thousands)	Attributable proportion (Female %)
Democratic Republic of the Congo	Lip and oral cavity cancer	-2.6	-35.27	-1.38	-31.45	-1.25	-41.83
Denmark	Lip and oral cavity cancer	-1.54	-57.03	-0.86	-48.14	-0.67	-73.33
Djibouti	Lip and oral cavity cancer	-0.07	-48.87	-0.04	-43.56	-0.03	-59.81
Dominica	Lip and oral cavity cancer	0	-11.14	0	-12.13	0	-15.87
Dominican Republic	Lip and oral cavity cancer	-1.25	-55.58	-0.7	-48.75	-0.53	-66.03
Ecuador	Lip and oral cavity cancer	-1.41	-111.49	-0.64	-84.62	-0.69	-137.37
Egypt	Lip and oral cavity cancer	-3.8	-140.88	-1.9	-112.98	-1.74	-171.45
El Salvador	Lip and oral cavity cancer	-0.91	-93.92	-0.46	-78.95	-0.42	-109.72
Equatorial Guinea	Lip and oral cavity cancer	-0.17	-161.81	-0.1	-149.18	-0.07	-179.11
Eritrea	Lip and oral cavity cancer	-0.43	-33.19	-0.22	-29.23	-0.19	-37.55
Estonia	Lip and oral cavity cancer	-1.02	-77.5	-0.73	-69.38	-0.29	-106.61
Eswatini	Lip and oral cavity cancer	-0.03	-11.84	-0.01	-6.7	-0.01	-17.23
Ethiopia	Lip and oral cavity cancer	-8.74	-66.31	-5.26	-62.57	-3.48	-72.72
Fiji	Lip and oral cavity cancer	-0.03	-12.38	-0.02	-14.29	-0.01	-11.04
Finland	Lip and oral cavity cancer	-1.61	-63.53	-0.82	-50.3	-0.77	-84.87
France	Lip and oral cavity cancer	-53.93	-63.89	-39.38	-53.11	-10.74	-104.47
Gabon	Lip and oral cavity cancer	-0.19	-51.08	-0.11	-48.09	-0.08	-56.32
Gambia	Lip and oral cavity cancer	-0.04	-37.73	-0.01	-22.42	-0.02	-43.5
Georgia	Lip and oral cavity cancer	-0.42	-14.84	-0.25	-10.29	-0.09	-20.85

Location	Cause	Attributable number (Both thousands)	Attributable proportion (Both %)	Attributable number (Male thousands)	Attributable proportion (Male %)	Attributable number (Female thousands)	Attributable proportion (Female %)
Germany	Lip and oral cavity cancer	-40.41	-49.53	-26.93	-40.77	-12.08	-77.69
Ghana	Lip and oral cavity cancer	-0.38	-52.99	-0.05	-23.21	-0.28	-57.32
Greece	Lip and oral cavity cancer	-0.91	-22.18	-0.37	-13.16	-0.52	-40.42
Greenland	Lip and oral cavity cancer	-0.03	-46.41	-0.02	-45.66	-0.01	-50.12
Grenada	Lip and oral cavity cancer	-0.02	-34.42	-0.01	-34.21	0	-37.52
Guam	Lip and oral cavity cancer	0	-5.65	0	-17.19	0	-6.7
Guatemala	Lip and oral cavity cancer	-1	-80.54	-0.46	-65.1	-0.51	-94.24
Guinea	Lip and oral cavity cancer	-0.58	-20.37	-0.23	-15.71	-0.37	-26.64
Guinea-Bissau	Lip and oral cavity cancer	-0.04	-31.12	-0.01	-25.18	-0.03	-35.01
Guyana	Lip and oral cavity cancer	-0.05	-27.98	-0.03	-25.49	-0.02	-33.58
Haiti	Lip and oral cavity cancer	-0.57	-24.22	-0.37	-21.58	-0.2	-30.92
Honduras	Lip and oral cavity cancer	-0.37	-66.74	-0.14	-48.35	-0.2	-78.1
Hungary	Lip and oral cavity cancer	-12.64	-58.1	-9.38	-50.37	-2.41	-77.16
Iceland	Lip and oral cavity cancer	-0.06	-50.71	-0.03	-35.95	-0.03	-71.99
India	Lip and oral cavity cancer	-648.93	-77.26	-391.66	-68.8	-248.59	-91.87
Indonesia	Lip and oral cavity cancer	-24.95	-52.56	-16.07	-57.35	-10.62	-54.6
Iran (Islamic Republic of)	Lip and oral cavity cancer	-3.75	-139.24	-2.03	-127.22	-1.67	-151.55
Iraq	Lip and oral cavity cancer	-2.62	-113.03	-1.37	-98.24	-1.19	-127.9
Ireland	Lip and oral cavity cancer	-1.48	-64.35	-0.96	-54.24	-0.5	-93.68
Israel	Lip and oral cavity cancer	-0.91	-77.95	-0.47	-62.63	-0.44	-103.58

Location	Cause	Attributable number (Both thousands)	Attributable proportion (Both %)	Attributable number (Male thousands)	Attributable proportion (Male %)	Attributable number (Female thousands)	Attributable proportion (Female %)
Italy	Lip and oral cavity cancer	-29.36	-55.98	-18.97	-44.83	-8.32	-82.02
Jamaica	Lip and oral cavity cancer	-0.13	-24.08	-0.08	-21.37	-0.06	-29.35
Japan	Lip and oral cavity cancer	-25.76	-59.78	-17.03	-58.05	-9.36	-68.05
Jordan	Lip and oral cavity cancer	-0.78	-171.74	-0.53	-178.68	-0.27	-173.72
Kazakhstan	Lip and oral cavity cancer	-4.6	-43.45	-2.73	-34.14	-1.35	-52.15
Kenya	Lip and oral cavity cancer	-2.97	-42.42	-1.24	-30.22	-1.61	-55.1
Kiribati	Lip and oral cavity cancer	-0.01	-17.82	-0.01	-18.59	0	-16.75
Kuwait	Lip and oral cavity cancer	-0.24	-69.8	-0.14	-59.5	-0.08	-81.08
Kyrgyzstan	Lip and oral cavity cancer	-1.26	-36.06	-0.71	-24.67	-0.28	-45.63
Lao People's Democratic Republic	Lip and oral cavity cancer	-0.78	-60.08	-0.48	-58.51	-0.31	-64.62
Latvia	Lip and oral cavity cancer	-1.54	-55.75	-0.85	-36.08	-0.33	-84.24
Lebanon	Lip and oral cavity cancer	-0.85	-115.17	-0.55	-114	-0.31	-121.06
Lesotho	Lip and oral cavity cancer	0.05	9.35	0.05	12.49	0.01	5.08
Liberia	Lip and oral cavity cancer	-0.17	-63.03	-0.07	-55.72	-0.09	-68.63
Libya	Lip and oral cavity cancer	-0.53	-75.99	-0.25	-62.2	-0.25	-86.39
Lithuania	Lip and oral cavity cancer	-1.44	-45.28	-0.96	-36.45	-0.35	-66.29
Luxembourg	Lip and oral cavity cancer	-0.23	-57.6	-0.16	-49.51	-0.06	-92.05
Madagascar	Lip and oral cavity cancer	-1.04	-23.02	-0.5	-17.79	-0.48	-28.62
Malawi	Lip and oral cavity cancer	-0.87	-36.06	-0.37	-32.16	-0.54	-42.22

Location	Cause	Attributable number (Both thousands)	Attributable proportion (Both %)	Attributable number (Male thousands)	Attributable proportion (Male %)	Attributable number (Female thousands)	Attributable proportion (Female %)
Malaysia	Lip and oral cavity cancer	-7.08	-88.79	-4.1	-88.57	-3	-89.59
Maldives	Lip and oral cavity cancer	-0.1	-165.51	-0.07	-189.46	-0.04	-158.33
Mali	Lip and oral cavity cancer	-0.55	-33.08	-0.22	-28.42	-0.35	-38.65
Malta	Lip and oral cavity cancer	-0.11	-62.69	-0.06	-47.31	-0.05	-96.52
Marshall Islands	Lip and oral cavity cancer	0	-23.4	0	-24.71	0	-19.96
Mauritania	Lip and oral cavity cancer	-0.23	-85.33	-0.08	-74.9	-0.15	-91.54
Mauritius	Lip and oral cavity cancer	-0.21	-36.86	-0.21	-51.16	-0.05	-33.54
Mexico	Lip and oral cavity cancer	-9.41	-77.66	-5.11	-65.33	-3.95	-92.07
Micronesia (Federated States of)	Lip and oral cavity cancer	-0.01	-33.52	-0.01	-33.17	0	-33.83
Monaco	Lip and oral cavity cancer	0	-28.49	0	-18.19	0	-46.68
Mongolia	Lip and oral cavity cancer	-0.48	-54.95	-0.26	-49.37	-0.23	-63.63
Montenegro	Lip and oral cavity cancer	-0.09	-23.19	-0.08	-23.25	-0.01	-24.85
Morocco	Lip and oral cavity cancer	-2.36	-82.87	-1.09	-69.12	-1.19	-94.29
Mozambique	Lip and oral cavity cancer	-0.41	-16.83	-0.25	-13.79	-0.16	-26.66
Myanmar	Lip and oral cavity cancer	-8.34	-62.74	-4.85	-61.67	-3.6	-66.09
Namibia	Lip and oral cavity cancer	-0.52	-51.71	-0.32	-48.29	-0.2	-59.75
Nauru	Lip and oral cavity cancer	0	-20.26	0	-17.45	0	-18.85
Nepal	Lip and oral cavity cancer	-11.39	-75.69	-6.58	-66	-4.59	-90.41
Netherlands	Lip and oral cavity cancer	-3.4	-43.66	-1.7	-31.88	-1.55	-63.54

Location	Cause	Attributable number (Both thousands)	Attributable proportion (Both %)	Attributable number (Male thousands)	Attributable proportion (Male %)	Attributable number (Female thousands)	Attributable proportion (Female %)
New Zealand	Lip and oral cavity cancer	-0.97	-54.94	-0.69	-57.85	-0.32	-54.87
Nicaragua	Lip and oral cavity cancer	-0.23	-87.59	-0.13	-77.6	-0.1	-99.86
Niger	Lip and oral cavity cancer	-0.22	-35.19	-0.07	-25.61	-0.14	-41.31
Nigeria	Lip and oral cavity cancer	-3.66	-47.33	-0.97	-27.41	-2.3	-54.79
Niue	Lip and oral cavity cancer	0	-25.8	0	-27.4	0	-24.51
North Macedonia	Lip and oral cavity cancer	-0.62	-66.02	-0.46	-62.35	-0.15	-74.92
Northern Mariana Islands	Lip and oral cavity cancer	-0.01	-45.77	-0.01	-36.73	0	-34.22
Norway	Lip and oral cavity cancer	-1.27	-51.73	-0.7	-40.42	-0.56	-76.75
Oman	Lip and oral cavity cancer	-0.34	-129.41	-0.25	-127.02	-0.08	-137.54
Pakistan	Lip and oral cavity cancer	-80.64	-36.26	-32.29	-25.11	-42.83	-45.63
Palau	Lip and oral cavity cancer	-0.01	-24.93	-0.01	-32.93	-0.01	-24.92
Palestine	Lip and oral cavity cancer	-0.12	-95.81	-0.07	-93.75	-0.05	-102.2
Panama	Lip and oral cavity cancer	-0.43	-74.56	-0.24	-64.43	-0.17	-86.04
Papua New Guinea	Lip and oral cavity cancer	-0.09	-13.96	-0.07	-16.13	-0.02	-10.72
Paraguay	Lip and oral cavity cancer	-0.5	-60.12	-0.38	-57.25	-0.12	-71.79
Peru	Lip and oral cavity cancer	-4.4	-136.39	-2.08	-116.12	-2.21	-153.85
Philippines	Lip and oral cavity cancer	-4.22	-23.09	-2.53	-20.46	-1.27	-21.5
Poland	Lip and oral cavity cancer	-26.83	-85.49	-19.6	-78.15	-6.37	-101.02
Portugal	Lip and oral cavity cancer	-8.91	-92.89	-6.91	-86.42	-1.83	-114.64
Puerto Rico	Lip and oral cavity cancer	-1.23	-46.34	-0.91	-42.03	-0.28	-58.33

Location	Cause	Attributable number (Both thousands)	Attributable proportion (Both %)	Attributable number (Male thousands)	Attributable proportion (Male %)	Attributable number (Female thousands)	Attributable proportion (Female %)
Qatar	Lip and oral cavity cancer	-0.15	-429.14	-0.1	-401.4	-0.04	-491.25
Republic of Korea	Lip and oral cavity cancer	-20.94	-228.19	-14.67	-222.35	-6.37	-247.09
Republic of Moldova	Lip and oral cavity cancer	-1.85	-44.91	-1.38	-38.03	-0.34	-68.66
Romania	Lip and oral cavity cancer	-16.64	-86.35	-14.38	-88.31	-2.61	-87.35
Russian Federation	Lip and oral cavity cancer	-89.92	-62.35	-53.83	-45.36	-21.56	-84.45
Rwanda	Lip and oral cavity cancer	-1.77	-50.23	-1.04	-48.65	-0.76	-55
Saint Kitts and Nevis	Lip and oral cavity cancer	-0.01	-62.55	-0.01	-68.17	0	-59.71
Saint Lucia	Lip and oral cavity cancer	-0.04	-53.24	-0.03	-55.98	-0.01	-55.1
Saint Vincent and the Grenadines	Lip and oral cavity cancer	-0.02	-22.66	-0.01	-24.52	0	-26.8
Samoa	Lip and oral cavity cancer	-0.01	-31.01	-0.01	-29.73	0	-29.47
San Marino	Lip and oral cavity cancer	0	-21.23	0	-14.64	0	-35.12
Sao Tome and Principe	Lip and oral cavity cancer	0	-61.95	0	-58.08	0	-62.58
Saudi Arabia	Lip and oral cavity cancer	-4.37	-178.02	-2.33	-158.36	-1.94	-197.99
Senegal	Lip and oral cavity cancer	-0.39	-48.9	-0.15	-38.95	-0.23	-55.47
Serbia	Lip and oral cavity cancer	-5.71	-66.88	-4.22	-60.96	-1.44	-88.84
Seychelles	Lip and oral cavity cancer	-0.05	-57.79	-0.04	-60.2	0	-49.15
Sierra Leone	Lip and oral cavity cancer	-0.15	-34.46	-0.06	-26.91	-0.08	-38.94
Singapore	Lip and oral cavity cancer	-1.29	-157.08	-0.86	-150.39	-0.42	-167.87
Slovakia	Lip and oral cavity cancer	-3.68	-39.42	-3.06	-36.5	-0.5	-52.17

Location	Cause	Attributable number (Both thousands)	Attributable proportion (Both %)	Attributable number (Male thousands)	Attributable proportion (Male %)	Attributable number (Female thousands)	Attributable proportion (Female %)
Slovenia	Lip and oral cavity cancer	-1.27	-66.45	-1.07	-65.14	-0.21	-77.17
Solomon Islands	Lip and oral cavity cancer	-0.02	-35.17	-0.01	-31.42	0	-34.16
Somalia	Lip and oral cavity cancer	-0.22	-9.31	-0.09	-6.05	-0.11	-12.65
South Africa	Lip and oral cavity cancer	-4.96	-25.3	-2.94	-19.72	-1.55	-33.01
South Sudan	Lip and oral cavity cancer	-0.34	-16.41	-0.17	-11.28	-0.13	-21.79
Spain	Lip and oral cavity cancer	-16.33	-43.35	-11.93	-37.27	-4.93	-87.19
Sri Lanka	Lip and oral cavity cancer	-12.92	-104.55	-10.33	-118.34	-3.51	-96.81
Sudan	Lip and oral cavity cancer	-1.19	-72.34	-0.62	-62.49	-0.55	-83.26
Suriname	Lip and oral cavity cancer	-0.03	-29.68	-0.02	-27.45	-0.01	-36.66
Sweden	Lip and oral cavity cancer	-1.84	-40.83	-0.88	-31.65	-0.94	-54.05
Switzerland	Lip and oral cavity cancer	-1.78	-45.02	-1.26	-42.93	-0.5	-49.21
Syrian Arab Republic	Lip and oral cavity cancer	-0.75	-99.92	-0.41	-90.65	-0.32	-108.74
Taiwan (Province of China)	Lip and oral cavity cancer	-17.46	-120.11	-18.32	-148.36	-1.92	-87.66
Tajikistan	Lip and oral cavity cancer	-0.1	-14.18	-0.05	-11.2	-0.05	-16.87
Thailand	Lip and oral cavity cancer	-36.28	-97.91	-25.01	-101.84	-12.74	-101.94
Timor-Leste	Lip and oral cavity cancer	-0.07	-50.99	-0.04	-49.31	-0.03	-57.33
Togo	Lip and oral cavity cancer	-0.15	-44.46	-0.06	-35.65	-0.1	-52.92
Tokelau	Lip and oral cavity cancer	0	-43.6	0	-44.29	0	-45.55
Tonga	Lip and oral cavity cancer	0	-21.23	0	-23.97	0	-20.49
Trinidad and Tobago	Lip and oral cavity cancer	-0.18	-42.82	-0.13	-42.33	-0.05	-47.92

Location	Cause	Attributable number (Both thousands)	Attributable proportion (Both %)	Attributable number (Male thousands)	Attributable proportion (Male %)	Attributable number (Female thousands)	Attributable proportion (Female %)
Tunisia	Lip and oral cavity cancer	-1.8	-92.88	-1.15	-84.56	-0.62	-106.64
Turkey	Lip and oral cavity cancer	-12.2	-130.76	-7.52	-122.38	-4.58	-143.76
Turkmenistan	Lip and oral cavity cancer	-0.62	-38.79	-0.31	-28.52	-0.22	-44.57
Tuvalu	Lip and oral cavity cancer	0	-45.08	0	-50.84	0	-46.49
Uganda	Lip and oral cavity cancer	-2.74	-40.96	-1.48	-31.45	-1.03	-51.95
Ukraine	Lip and oral cavity cancer	-12.89	-27.35	-10.25	-26.67	-2.75	-31.54
United Arab Emirates	Lip and oral cavity cancer	-0.66	-171.93	-0.53	-175.95	-0.16	-181.96
United Kingdom	Lip and oral cavity cancer	-21.65	-65.24	-15.65	-71.82	-6.69	-58.69
United Republic of Tanzania	Lip and oral cavity cancer	-3.13	-29.01	-1.76	-24.32	-1.26	-35.5
United States of America	Lip and oral cavity cancer	-55.85	-35.47	-39.68	-36.8	-16.86	-33.98
United States Virgin Islands	Lip and oral cavity cancer	-0.01	-22.98	-0.01	-20.74	0	-33.46
Uruguay	Lip and oral cavity cancer	-1.06	-41.4	-0.73	-35.18	-0.29	-60.76
Uzbekistan	Lip and oral cavity cancer	-1.31	-27.42	-0.66	-23.26	-0.59	-30.22
Vanuatu	Lip and oral cavity cancer	0	-13.47	0	-11.38	0	-7.72
Venezuela (Bolivarian Republic of)	Lip and oral cavity cancer	-2.78	-71.65	-1.6	-67.73	-1.21	-79.36
Viet Nam	Lip and oral cavity cancer	-35.52	-102.44	-26.8	-120.43	-11.79	-94.91
Yemen	Lip and oral cavity cancer	-0.57	-58.57	-0.3	-47.01	-0.23	-71.37
Zambia	Lip and oral cavity cancer	-1.57	-55.87	-1.06	-62.97	-0.69	-62.02
Zimbabwe	Lip and oral cavity cancer	0.34	15.98	0.28	20.94	0.18	21.58

Table S20. Absolute (the number) and relative contribution (the proportion) associated with disease severity changes for larynx cancer (LarC) by sex at global, SDI regional, GBD regional level between 1990 and 2021.

Location	Cause	Attributable number (Both thousands)	Attributable proportion (Both %)	Attributable number (Male thousands)	Attributable proportion (Male %)	Attributable number (Female thousands)	Attributable proportion (Female %)
Global	Larynx cancer	-835.51	-33.75	-726.39	-33.3	-105.41	-35.77
High SDI	Larynx cancer	-207.64	-43.75	-184.57	-44.09	-22.36	-39.91
High-middle SDI	Larynx cancer	-404.49	-50.54	-363.04	-48.93	-40.39	-69.22
Middle SDI	Larynx cancer	-388.23	-67.49	-329.19	-67.97	-59.13	-65.03
Low-middle SDI	Larynx cancer	-149.88	-31.99	-129.17	-31.99	-19.77	-30.55
Low SDI	Larynx cancer	-35.32	-23.05	-29.34	-22.77	-5.7	-23.44
High-income Asia Pacific	Larynx cancer	-26.01	-61.38	-22.66	-60.8	-3.47	-68.15
High-income North America	Larynx cancer	-38.77	-31.63	-31.96	-32.45	-6.94	-28.85
Western Europe	Larynx cancer	-160.83	-45.35	-146.72	-44.54	-12.49	-49.5
Australasia	Larynx cancer	-3.68	-51.79	-3.33	-51.45	-0.28	-43.37
Eastern Europe	Larynx cancer	-84.87	-28.08	-81.65	-28.24	-3.96	-30.38
Central Europe	Larynx cancer	-75.05	-44.72	-67.84	-43.53	-6.36	-52.98
Southern Latin America	Larynx cancer	-13.89	-34.67	-12.61	-33.95	-1.13	-38.49
East Asia	Larynx cancer	-386.22	-103.65	-320.09	-103.2	-66.29	-106.15
Central Asia	Larynx cancer	-8.85	-20.72	-7.61	-20.72	-1.21	-20.31
North Africa and Middle East	Larynx cancer	-90.18	-80.04	-79.79	-83.59	-10.56	-61.33
Andean Latin America	Larynx cancer	-3.15	-51.48	-2.42	-51.21	-0.73	-52.01

Location	Cause	Attributable number (Both thousands)	Attributable proportion (Both %)	Attributable number (Male thousands)	Attributable proportion (Male %)	Attributable number (Female thousands)	Attributable proportion (Female %)
Southeast Asia	Larynx cancer	-56.85	-60.73	-50.45	-64.7	-5.93	-37.92
Tropical Latin America	Larynx cancer	-37	-47.94	-32.85	-48.56	-4.16	-43.62
Southern Sub-Saharan Africa	Larynx cancer	-1.42	-8.62	-1.24	-8.98	-0.16	-6.1
Caribbean	Larynx cancer	-7.17	-37.56	-6.52	-40.73	-0.81	-26.16
Central Latin America	Larynx cancer	-16.38	-38.94	-13.37	-40.46	-3.19	-35.36
South Asia	Larynx cancer	-207.78	-36.09	-178.04	-35.47	-27.56	-37.34
Central Sub-Saharan Africa	Larynx cancer	-2.23	-20.31	-1.98	-20.55	-0.24	-17.97
Oceania	Larynx cancer	-0.05	-10.57	-0.04	-11.07	0	-6.39
Western Sub-Saharan Africa	Larynx cancer	-5.63	-19.05	-5.42	-19.16	-0.17	-13.69
Eastern Sub-Saharan Africa	Larynx cancer	-8.53	-21.45	-6.89	-21.84	-1.6	-19.47
Afghanistan	Larynx cancer	-1.3	-20.6	-1.03	-23.19	-0.32	-17.64
Albania	Larynx cancer	-1.05	-55.96	-0.86	-54.15	-0.18	-64.67
Algeria	Larynx cancer	-4.38	-75.61	-3.97	-78.04	-0.42	-59.99
American Samoa	Larynx cancer	0	-16.53	0	-16.97	0	-5.31
Andorra	Larynx cancer	-0.01	-50.31	-0.01	-50.23	0	-51.14
Angola	Larynx cancer	-0.7	-30.19	-0.61	-29.55	-0.07	-30.06
Antigua and Barbuda	Larynx cancer	-0.01	-32.04	-0.01	-32.18	0	-24.09
Argentina	Larynx cancer	-9.41	-30.35	-8.55	-29.68	-0.72	-32.9
Armenia	Larynx cancer	-0.96	-27.19	-0.89	-27.44	-0.09	-30.04
Australia	Larynx cancer	-3.39	-54.34	-3.04	-52.93	-0.22	-43.81

Location	Cause	Attributable number (Both thousands)	Attributable proportion (Both %)	Attributable number (Male thousands)	Attributable proportion (Male %)	Attributable number (Female thousands)	Attributable proportion (Female %)
Austria	Larynx cancer	-2.37	-42.04	-2.13	-41.02	-0.21	-48.11
Azerbaijan	Larynx cancer	-1.45	-29.33	-1.18	-28.74	-0.25	-29.57
Bahamas	Larynx cancer	-0.03	-29.04	-0.03	-30.44	0	-20.52
Bahrain	Larynx cancer	-0.18	-136.03	-0.17	-142.8	-0.02	-103.1
Bangladesh	Larynx cancer	-25.38	-47.98	-21.69	-46.02	-3.3	-57.14
Barbados	Larynx cancer	-0.03	-28.22	-0.02	-29.71	0	-22.51
Belarus	Larynx cancer	-5.45	-38.64	-5.29	-38.64	-0.17	-43.37
Belgium	Larynx cancer	-3.91	-36.27	-3.53	-36.21	-0.42	-41.16
Belize	Larynx cancer	-0.01	-30.36	-0.01	-31.79	0	-25.26
Benin	Larynx cancer	-0.09	-18.74	-0.09	-19.16	-0.01	-18.09
Bermuda	Larynx cancer	-0.03	-72.76	-0.03	-75.49	0	-57.63
Bhutan	Larynx cancer	-0.07	-38.34	-0.06	-37.4	-0.01	-42.18
Bolivia (Plurinational State of)	Larynx cancer	-0.46	-34.57	-0.36	-34.41	-0.1	-34.96
Bosnia and Herzegovina	Larynx cancer	-2.06	-39.55	-1.78	-38.64	-0.28	-45.67
Botswana	Larynx cancer	-0.07	-17.03	-0.06	-17.45	-0.01	-15.34
Brazil	Larynx cancer	-36.65	-47.99	-32.53	-48.6	-4.13	-43.69
Brunei Darussalam	Larynx cancer	-0.03	-58.39	-0.02	-57.5	-0.01	-59.81
Bulgaria	Larynx cancer	-3.47	-30.97	-3.32	-31.41	-0.16	-24.45
Burkina Faso	Larynx cancer	-0.12	-10.44	-0.11	-10.7	-0.01	-10.43
Burundi	Larynx cancer	-0.28	-17.39	-0.24	-19.15	-0.04	-13.06

Location	Cause	Attributable number (Both thousands)	Attributable proportion (Both %)	Attributable number (Male thousands)	Attributable proportion (Male %)	Attributable number (Female thousands)	Attributable proportion (Female %)
Cabo Verde	Larynx cancer	-0.02	-29.89	-0.02	-29.39	0	-43.1
Cambodia	Larynx cancer	-1.07	-46.72	-0.91	-49.22	-0.16	-37.34
Cameroon	Larynx cancer	-0.24	-18.3	-0.23	-18.63	-0.02	-17.53
Canada	Larynx cancer	-4.92	-38.91	-4.25	-39.69	-0.68	-34.91
Central African Republic	Larynx cancer	-0.05	-6.01	-0.04	-6.38	0	-2.56
Chad	Larynx cancer	-0.01	-2.48	-0.01	-2.35	0	-4.7
Chile	Larynx cancer	-2.54	-62.71	-2.2	-61.82	-0.33	-68.39
China	Larynx cancer	-381.93	-105.36	-316.15	-104.94	-65.98	-107.77
Colombia	Larynx cancer	-5.57	-50.33	-4.29	-52.7	-1.32	-45.46
Comoros	Larynx cancer	-0.02	-18.16	-0.02	-18.37	0	-16.63
Congo	Larynx cancer	-0.2	-29.95	-0.18	-30.77	-0.02	-25.1
Cook Islands	Larynx cancer	0	-57.68	0	-58.97	0	-49.32
Costa Rica	Larynx cancer	-0.27	-36.36	-0.24	-37.34	-0.03	-31.07
Coted'Ivoire	Larynx cancer	-0.38	-23.73	-0.37	-23.86	-0.01	-20.55
Croatia	Larynx cancer	-3.2	-38.59	-2.98	-37.88	-0.2	-47.11
Cuba	Larynx cancer	-4.7	-43.94	-4.18	-47.81	-0.65	-33.29
Cyprus	Larynx cancer	-0.26	-91.98	-0.24	-93.12	-0.02	-85.19
Czechia	Larynx cancer	-5.03	-49.57	-4.48	-47.09	-0.4	-63.22
Democratic People's Republic of Korea	Larynx cancer	-1.9	-38.94	-1.68	-41.23	-0.22	-27.51
Democratic Republic of the Congo	Larynx cancer	-1.12	-16.52	-0.99	-16.78	-0.13	-14.82

Location	Cause	Attributable number (Both thousands)	Attributable proportion (Both %)	Attributable number (Male thousands)	Attributable proportion (Male %)	Attributable number (Female thousands)	Attributable proportion (Female %)
Denmark	Larynx cancer	-1.55	-40.28	-1.32	-41.29	-0.24	-35.69
Djibouti	Larynx cancer	-0.03	-27.68	-0.02	-29.36	0	-22.66
Dominica	Larynx cancer	0	-11.29	0	-13.32	0	-7.93
Dominican Republic	Larynx cancer	-0.44	-27.89	-0.34	-28.76	-0.1	-27.24
Ecuador	Larynx cancer	-0.61	-43.84	-0.47	-45.71	-0.15	-40.4
Egypt	Larynx cancer	-5.78	-63.16	-4.99	-69.05	-0.82	-42.89
El Salvador	Larynx cancer	-0.33	-46.42	-0.26	-46.7	-0.07	-45.8
Equatorial Guinea	Larynx cancer	-0.07	-58	-0.06	-60.46	-0.01	-58.93
Eritrea	Larynx cancer	-0.2	-21.86	-0.16	-23.02	-0.03	-17.44
Estonia	Larynx cancer	-0.75	-49.02	-0.7	-49.57	-0.04	-39.27
Eswatini	Larynx cancer	-0.01	-4.96	-0.01	-5.26	0	-2.73
Ethiopia	Larynx cancer	-3.15	-32.05	-2.61	-31.82	-0.52	-31.85
Fiji	Larynx cancer	-0.01	-11.32	-0.01	-11.37	0	-7.74
Finland	Larynx cancer	-0.74	-48.6	-0.74	-53.94	-0.02	-16.33
France	Larynx cancer	-45.17	-53.56	-42.03	-52.35	-3.07	-76.05
Gabon	Larynx cancer	-0.08	-26.6	-0.08	-26.88	-0.01	-23.4
Gambia	Larynx cancer	-0.01	-14.64	-0.01	-14.22	0	-15.32
Georgia	Larynx cancer	-0.81	-10.59	-0.78	-11.13	-0.1	-14.93
Germany	Larynx cancer	-25.76	-46.58	-23.12	-45.44	-2.71	-61.14
Ghana	Larynx cancer	-0.29	-20.3	-0.26	-20.42	-0.03	-21.69

Location	Cause	Attributable number (Both thousands)	Attributable proportion (Both %)	Attributable number (Male thousands)	Attributable proportion (Male %)	Attributable number (Female thousands)	Attributable proportion (Female %)
Greece	Larynx cancer	-2.58	-25.47	-2.4	-25.66	-0.18	-23.46
Greenland	Larynx cancer	-0.01	-37.36	-0.01	-37.88	0	-36.52
Grenada	Larynx cancer	-0.01	-24.29	-0.01	-26.73	0	-20.13
Guam	Larynx cancer	0	-18.56	0	-19.71	0	-9.9
Guatemala	Larynx cancer	-0.49	-35.47	-0.34	-36.48	-0.15	-33.44
Guinea	Larynx cancer	-0.04	-5.78	-0.04	-5.43	-0.01	-9.58
Guinea-Bissau	Larynx cancer	-0.02	-14.3	-0.02	-14.69	0	-11.43
Guyana	Larynx cancer	-0.02	-19.19	-0.02	-20.05	0	-15.97
Haiti	Larynx cancer	-0.36	-14	-0.33	-14.1	-0.03	-13.04
Honduras	Larynx cancer	-0.16	-25.33	-0.11	-23.94	-0.05	-27.49
Hungary	Larynx cancer	-8.78	-46.14	-7.89	-45.06	-0.81	-52.5
Iceland	Larynx cancer	-0.02	-43.12	-0.02	-43	0	-47.91
India	Larynx cancer	-166.61	-38.32	-143.37	-37.66	-21.68	-40.12
Indonesia	Larynx cancer	-10.62	-34.94	-9.04	-37.17	-1.56	-25.67
Iran (Islamic Republic of)	Larynx cancer	-18.39	-93.02	-15.25	-98.22	-3.18	-74.9
Iraq	Larynx cancer	-4.69	-83.64	-3.78	-87.76	-0.92	-70.37
Ireland	Larynx cancer	-1.09	-59.18	-0.9	-58.5	-0.19	-64.28
Israel	Larynx cancer	-0.92	-63.76	-0.79	-63.4	-0.14	-65.91
Italy	Larynx cancer	-26.35	-39.56	-24.03	-38.17	-1.97	-53.87
Jamaica	Larynx cancer	-0.09	-18.19	-0.09	-19.21	-0.01	-13.82

Location	Cause	Attributable number (Both thousands)	Attributable proportion (Both %)	Attributable number (Male thousands)	Attributable proportion (Male %)	Attributable number (Female thousands)	Attributable proportion (Female %)
Japan	Larynx cancer	-10.08	-43.29	-9.01	-42.99	-1.19	-51.19
Jordan	Larynx cancer	-0.74	-124.39	-0.66	-129.72	-0.09	-99.8
Kazakhstan	Larynx cancer	-3.46	-25.79	-3.12	-25.85	-0.39	-28.49
Kenya	Larynx cancer	-0.3	-10.76	-0.24	-10.79	-0.06	-11.76
Kiribati	Larynx cancer	0	-11.03	0	-12.33	0	-8.27
Kuwait	Larynx cancer	-0.22	-76.14	-0.19	-73.56	-0.03	-73.76
Kyrgyzstan	Larynx cancer	-0.47	-24.7	-0.43	-24.62	-0.05	-28.61
Lao People's Democratic Republic	Larynx cancer	-0.35	-33.85	-0.31	-35.44	-0.05	-26.95
Latvia	Larynx cancer	-0.86	-26.22	-0.81	-26.41	-0.05	-27.02
Lebanon	Larynx cancer	-1.67	-94.4	-1.46	-97.74	-0.22	-79.99
Lesotho	Larynx cancer	0.07	13.52	0.05	13.19	0.01	11.96
Liberia	Larynx cancer	-0.07	-24.91	-0.06	-24.93	-0.01	-27.07
Libya	Larynx cancer	-1.11	-65.24	-1.01	-66.97	-0.09	-48.4
Lithuania	Larynx cancer	-1.14	-24.87	-1.1	-25.18	-0.05	-24.42
Luxembourg	Larynx cancer	-0.21	-54.84	-0.19	-54.86	-0.02	-61.13
Madagascar	Larynx cancer	-0.31	-12.68	-0.25	-12.97	-0.06	-10.99
Malawi	Larynx cancer	-0.11	-15.93	-0.08	-16.05	-0.03	-16.76
Malaysia	Larynx cancer	-2.68	-70.11	-2.42	-72.49	-0.27	-54.69
Maldives	Larynx cancer	-0.03	-102.49	-0.03	-110.49	0	-87.66
Mali	Larynx cancer	-0.2	-15.93	-0.15	-16.03	-0.05	-16.04

Location	Cause	Attributable number (Both thousands)	Attributable proportion (Both %)	Attributable number (Male thousands)	Attributable proportion (Male %)	Attributable number (Female thousands)	Attributable proportion (Female %)
Malta	Larynx cancer	-0.12	-52.65	-0.11	-52.71	-0.01	-60.82
Marshall Islands	Larynx cancer	0	-17.91	0	-18.88	0	-7.73
Mauritania	Larynx cancer	-0.09	-38.22	-0.09	-38.24	-0.01	-36.74
Mauritius	Larynx cancer	-0.19	-45.93	-0.18	-45.91	-0.01	-54.24
Mexico	Larynx cancer	-7	-35.18	-5.8	-35.73	-1.27	-34.37
Micronesia (Federated States of)	Larynx cancer	0	-20.24	0	-20.67	0	-15.83
Monaco	Larynx cancer	-0.03	-30.74	-0.03	-30.87	0	-32.05
Mongolia	Larynx cancer	-0.15	-32.58	-0.12	-32.49	-0.04	-35.98
Montenegro	Larynx cancer	-0.27	-27.25	-0.21	-26.35	-0.06	-30.41
Morocco	Larynx cancer	-4.21	-54.69	-3.93	-55.7	-0.27	-43.59
Mozambique	Larynx cancer	-0.19	-5.45	-0.14	-5.58	-0.05	-6.69
Myanmar	Larynx cancer	-3.66	-34.28	-2.97	-36.11	-0.67	-27.31
Namibia	Larynx cancer	-0.12	-24.87	-0.1	-25.77	-0.03	-23.44
Nauru	Larynx cancer	0	-12.42	0	-12.65	0	-8.86
Nepal	Larynx cancer	-2.75	-33.28	-2.13	-31.83	-0.59	-37.69
Netherlands	Larynx cancer	-2.58	-37.11	-2.26	-37.49	-0.37	-40.48
New Zealand	Larynx cancer	-0.4	-45.57	-0.34	-46.57	-0.05	-36.8
Nicaragua	Larynx cancer	-0.2	-43.29	-0.17	-44.87	-0.04	-38.53
Niger	Larynx cancer	-0.1	-14.93	-0.09	-14.95	-0.01	-14.92
Nigeria	Larynx cancer	-3.6	-20.4	-3.53	-20.15	0.01	8.31

Location	Cause	Attributable number (Both thousands)	Attributable proportion (Both %)	Attributable number (Male thousands)	Attributable proportion (Male %)	Attributable number (Female thousands)	Attributable proportion (Female %)
Niue	Larynx cancer	0	-19.29	0	-19.89	0	-14.15
North Macedonia	Larynx cancer	-1.02	-43.48	-0.92	-43.45	-0.09	-42.16
Northern Mariana Islands	Larynx cancer	0	-23.26	0	-22.66	0	46.15
Norway	Larynx cancer	-0.48	-44.23	-0.41	-45.59	-0.09	-49.3
Oman	Larynx cancer	-0.17	-99.73	-0.15	-102.58	-0.02	-80.64
Pakistan	Larynx cancer	-13.44	-16.88	-11.25	-16.73	-2	-16.12
Palau	Larynx cancer	0	-29.45	0	-30.61	0	-18.26
Palestine	Larynx cancer	-0.21	-70.98	-0.2	-74.2	-0.02	-52.73
Panama	Larynx cancer	-0.22	-39.99	-0.19	-41.39	-0.03	-34.41
Papua New Guinea	Larynx cancer	-0.03	-11.9	-0.03	-12.71	0	-5.76
Paraguay	Larynx cancer	-0.34	-42.02	-0.31	-43.21	-0.03	-35.33
Peru	Larynx cancer	-2.1	-61.74	-1.6	-60.64	-0.49	-64.2
Philippines	Larynx cancer	-1.27	-15.95	-1.13	-17.59	-0.15	-9.71
Poland	Larynx cancer	-23.56	-43	-20.44	-40.27	-2.42	-60.18
Portugal	Larynx cancer	-5.47	-42.86	-4.84	-40.32	-0.48	-63.15
Puerto Rico	Larynx cancer	-0.83	-40.67	-0.75	-40.9	-0.09	-38.93
Qatar	Larynx cancer	-0.24	-289.92	-0.22	-301.92	-0.02	-204.54
Republic of Korea	Larynx cancer	-20.14	-110.92	-17.86	-115.21	-2.65	-100.05
Republic of Moldova	Larynx cancer	-1.59	-32.61	-1.52	-32.94	-0.07	-27.23
Romania	Larynx cancer	-16.25	-57.22	-15.35	-57.53	-0.89	-52.08

Location	Cause	Attributable number (Both thousands)	Attributable proportion (Both %)	Attributable number (Male thousands)	Attributable proportion (Male %)	Attributable number (Female thousands)	Attributable proportion (Female %)
Russian Federation	Larynx cancer	-61.91	-31.62	-59.85	-32.03	-3.16	-35.25
Rwanda	Larynx cancer	-0.67	-28.47	-0.52	-28.49	-0.15	-27.68
Saint Kitts and Nevis	Larynx cancer	-0.01	-47.7	-0.01	-48.65	0	-35.29
Saint Lucia	Larynx cancer	-0.02	-42.47	-0.02	-45.33	0	-34.19
Saint Vincent and the Grenadines	Larynx cancer	-0.01	-21.25	-0.01	-22.72	0	-15.82
Samoa	Larynx cancer	0	-22.58	0	-24.55	0	-15.36
San Marino	Larynx cancer	-0.01	-31.52	-0.01	-31.81	0	-30.17
Sao Tome and Principe	Larynx cancer	0	-21.06	0	-21.09	0	-24.68
Saudi Arabia	Larynx cancer	-2.14	-140.39	-1.85	-142	-0.28	-128.44
Senegal	Larynx cancer	-0.18	-19.57	-0.17	-19.77	-0.01	-18.61
Serbia	Larynx cancer	-7.01	-50.22	-6.22	-48.34	-0.68	-61.86
Seychelles	Larynx cancer	-0.04	-47.33	-0.04	-49.46	0	-25.87
Sierra Leone	Larynx cancer	-0.07	-14.12	-0.07	-14.4	0	-11.81
Singapore	Larynx cancer	-0.88	-100.95	-0.8	-104.07	-0.08	-87.13
Slovakia	Larynx cancer	-2.37	-34.05	-2.22	-33.32	-0.13	-45.63
Slovenia	Larynx cancer	-1.13	-58.47	-1.04	-58.38	-0.09	-62.41
Solomon Islands	Larynx cancer	-0.01	-19.09	-0.01	-19.6	0	-12.15
Somalia	Larynx cancer	-0.13	-7.3	-0.11	-7.66	-0.02	-4.42
South Africa	Larynx cancer	-1.57	-12.72	-1.35	-13.07	-0.21	-10.33
South Sudan	Larynx cancer	-0.14	-9.25	-0.11	-8.94	-0.02	-8.34

Location	Cause	Attributable number (Both thousands)	Attributable proportion (Both %)	Attributable number (Male thousands)	Attributable proportion (Male %)	Attributable number (Female thousands)	Attributable proportion (Female %)
Spain	Larynx cancer	-29.69	-49.25	-28.49	-48.65	-1.13	-66.14
Sri Lanka	Larynx cancer	-2.49	-95	-2.14	-96.11	-0.37	-94.27
Sudan	Larynx cancer	-2.83	-45.49	-2.37	-47.49	-0.47	-37.62
Suriname	Larynx cancer	-0.01	-19.73	-0.01	-20.45	0	-17.78
Sweden	Larynx cancer	-0.55	-33.67	-0.48	-34.31	-0.08	-36.17
Switzerland	Larynx cancer	-1.51	-41.62	-1.39	-43.5	-0.14	-32.69
Syrian Arab Republic	Larynx cancer	-1.68	-77.77	-1.47	-83.3	-0.23	-57.52
Taiwan (Province of China)	Larynx cancer	-3.99	-75.97	-3.71	-76.99	-0.22	-52.18
Tajikistan	Larynx cancer	-0.19	-14.12	-0.15	-14.11	-0.04	-11.95
Thailand	Larynx cancer	-16.64	-84.03	-15.25	-87.67	-1.34	-55.69
Timor-Leste	Larynx cancer	-0.02	-28.99	-0.02	-29.76	0	-25.22
Togo	Larynx cancer	-0.06	-16.78	-0.05	-17.02	-0.01	-17.59
Tokelau	Larynx cancer	0	-25.32	0	-26.01	0	-20.41
Tonga	Larynx cancer	0	-16.52	0	-17.03	0	-10.61
Trinidad and Tobago	Larynx cancer	-0.11	-36.2	-0.1	-37.7	-0.01	-29.24
Tunisia	Larynx cancer	-2.94	-82.69	-2.76	-84.03	-0.18	-65.16
Turkey	Larynx cancer	-32.21	-90.64	-29.84	-91.69	-2.34	-78.26
Turkmenistan	Larynx cancer	-0.35	-22.55	-0.26	-21.65	-0.08	-23.1
Tuvalu	Larynx cancer	0	-28.33	0	-30	0	-19.05
Uganda	Larynx cancer	-0.97	-22.92	-0.74	-22.44	-0.2	-21.44

Location	Cause	Attributable number (Both thousands)	Attributable proportion (Both %)	Attributable number (Male thousands)	Attributable proportion (Male %)	Attributable number (Female thousands)	Attributable proportion (Female %)
Ukraine	Larynx cancer	-12.95	-16.6	-12.05	-16.03	-0.71	-24.69
United Arab Emirates	Larynx cancer	-0.45	-172.94	-0.42	-183.75	-0.04	-123.07
United Kingdom	Larynx cancer	-10.17	-39.92	-8.13	-40.1	-2.08	-39.92
United Republic of Tanzania	Larynx cancer	-1.08	-17.58	-0.86	-18.15	-0.22	-15.55
United States of America	Larynx cancer	-33.82	-30.78	-27.65	-31.51	-6.28	-28.39
United States Virgin Islands	Larynx cancer	-0.01	-20.5	-0.01	-20.73	0	-22.18
Uruguay	Larynx cancer	-1.44	-28.66	-1.35	-28.37	-0.07	-28.95
Uzbekistan	Larynx cancer	-1.53	-19.49	-1.06	-17.71	-0.42	-22.55
Vanuatu	Larynx cancer	0	-10.38	0	-10.87	0	-4.58
Venezuela (Bolivarian Republic of)	Larynx cancer	-2.62	-39.46	-2.26	-43	-0.43	-31.39
Viet Nam	Larynx cancer	-13.46	-94.4	-12.46	-98.47	-0.99	-61.15
Yemen	Larynx cancer	-1.54	-38.69	-1.32	-40.85	-0.22	-29.41
Zambia	Larynx cancer	-0.43	-23.26	-0.33	-23.77	-0.1	-23.06
Zimbabwe	Larynx cancer	0.3	12.14	0.25	11.57	0.06	18.12

Table S21. Absolute (the number) and relative contribution (the proportion) associated with disease severity changes for Gallbladder and biliary tract cancer (GBTC), by sex at global, SDI regional, GBD regional level between 1990 and 2021.

Location	Cause	Attributable number (Both thousands)	Attributable proportion (Both %)	Attributable number (Male thousands)	Attributable proportion (Male %)	Attributable number (Female thousands)	Attributable proportion (Female %)
Global	Gallbladder and biliary tract cancer	-1151.94	-49.52	-605.51	-66.46	-509.16	-35.98
High SDI	Gallbladder and biliary tract cancer	-515.12	-61.27	-236.43	-71.2	-267.28	-52.55
High-middle SDI	Gallbladder and biliary tract cancer	-486.23	-77.48	-246.87	-101.1	-232.87	-60.74
Middle SDI	Gallbladder and biliary tract cancer	-355.17	-66.55	-195.55	-88.75	-152.99	-48.82
Low-middle SDI	Gallbladder and biliary tract cancer	-33.91	-13.27	-15.4	-16.8	-18.63	-11.36
Low SDI	Gallbladder and biliary tract cancer	-6.89	-10.56	-2.75	-12.59	-4.19	-9.64
High-income Asia Pacific	Gallbladder and biliary tract cancer	-193.31	-55.45	-104.28	-64.84	-88.46	-47.11
High-income North America	Gallbladder and biliary tract cancer	-57.46	-55.8	-25.89	-65.61	-29.69	-46.75
Western Europe	Gallbladder and biliary tract cancer	-235.78	-64.71	-103.59	-83.96	-124.97	-51.86
Australasia	Gallbladder and biliary tract cancer	-9.23	-105.27	-3.95	-116.34	-5.23	-97.21
Eastern Europe	Gallbladder and biliary tract cancer	-57.79	-57.25	-25.77	-75.58	-29.47	-44.08
Central Europe	Gallbladder and biliary tract cancer	-41.29	-27.4	-15.43	-35.1	-25.26	-23.67
Southern Latin America	Gallbladder and biliary tract cancer	-32.18	-34.21	-10.54	-44.57	-21.25	-30.18
East Asia	Gallbladder and biliary tract cancer	-612.37	-130.08	-343.78	-148.66	-264.87	-110.59
Central Asia	Gallbladder and biliary tract cancer	-0.63	-4.8	-0.28	-5.9	-0.36	-4.27
North Africa and Middle East	Gallbladder and biliary tract cancer	-25.73	-42.98	-12.2	-55.8	-13.23	-34.83

Andean Latin America	Gallbladder and biliary tract cancer	-7.74	-29.85	-2.74	-36.99	-4.94	-26.67
Southeast Asia	Gallbladder and biliary tract cancer	-56.09	-50.3	-33.32	-62.18	-22.06	-38.09
Tropical Latin America	Gallbladder and biliary tract cancer	-15.03	-20.8	-6.11	-27.62	-8.72	-17.38
Southern Sub-Saharan Africa	Gallbladder and biliary tract cancer	-0.21	-4.24	-0.13	-6.71	-0.07	-2.46
Caribbean	Gallbladder and biliary tract cancer	-1.34	-13.07	-0.73	-18	-0.58	-9.26
Central Latin America	Gallbladder and biliary tract cancer	-18.24	-20.36	-6.53	-25.84	-11.48	-17.86
South Asia	Gallbladder and biliary tract cancer	-49.08	-18	-21.76	-21.35	-27.71	-16.23
Central Sub-Saharan Africa	Gallbladder and biliary tract cancer	-0.12	-6.58	-0.06	-8.52	-0.07	-5.58
Oceania	Gallbladder and biliary tract cancer	-0.03	-5.68	-0.01	-6.9	-0.02	-4.81
Western Sub-Saharan Africa	Gallbladder and biliary tract cancer	-0.07	-6.79	-0.03	-7.22	-0.04	-6.65
Eastern Sub-Saharan Africa	Gallbladder and biliary tract cancer	-1.85	-8.64	-0.73	-10.49	-1.12	-7.77
Afghanistan	Gallbladder and biliary tract cancer	-0.14	-5.02	-0.04	-7.24	-0.1	-4.61
Albania	Gallbladder and biliary tract cancer	-0.18	-25.86	-0.1	-29.75	-0.08	-22.31
Algeria	Gallbladder and biliary tract cancer	-3.88	-36.3	-1.24	-44.58	-2.62	-33.15
American Samoa	Gallbladder and biliary tract cancer	0	-7.67	0	-9.53	0	-6.95
Andorra	Gallbladder and biliary tract cancer	-0.02	-66.71	-0.01	-74.81	-0.01	-57.92
Angola	Gallbladder and biliary tract cancer	-0.04	-11.01	-0.02	-12.62	-0.02	-9.92
Antigua and Barbuda	Gallbladder and biliary tract cancer	0	-12.39	0	-14.35	0	-10.17
Argentina	Gallbladder and biliary tract cancer	-8.71	-18.93	-3.17	-26.37	-5.29	-15.57
Armenia	Gallbladder and biliary tract cancer	-0.09	-16.8	-0.04	-19.68	-0.05	-15.42

Australia	Gallbladder and biliary tract cancer	-7.8	-105.13	-3.22	-111.61	-4.59	-101.17
Austria	Gallbladder and biliary tract cancer	-4.98	-47.56	-2.03	-65.5	-2.61	-35.47
Azerbaijan	Gallbladder and biliary tract cancer	-0.06	-5.28	-0.03	-6.9	-0.03	-4.31
Bahamas	Gallbladder and biliary tract cancer	-0.01	-11.31	-0.01	-14.15	0	-8.02
Bahrain	Gallbladder and biliary tract cancer	-0.04	-98.83	-0.03	-116.67	-0.02	-78.57
Bangladesh	Gallbladder and biliary tract cancer	-5.19	-23.1	-2.47	-24.96	-2.77	-21.97
Barbados	Gallbladder and biliary tract cancer	-0.02	-12.98	-0.01	-16.68	-0.01	-10.76
Belarus	Gallbladder and biliary tract cancer	-0.91	-23.84	-0.28	-21.48	-0.64	-25.58
Belgium	Gallbladder and biliary tract cancer	-2.58	-43.58	-1.17	-60.43	-1.3	-32.65
Belize	Gallbladder and biliary tract cancer	0	-9.73	0	-12.24	0	-8.22
Benin	Gallbladder and biliary tract cancer	0	-6.74	0	-6.75	0	-6.83
Bermuda	Gallbladder and biliary tract cancer	-0.01	-51.28	-0.01	-61.61	-0.01	-41.53
Bhutan	Gallbladder and biliary tract cancer	-0.02	-17.36	-0.01	-19.66	-0.01	-15.9
Bolivia (Plurinational State	Gallbladder and biliary tract cancer	-0.69	-11.38	-0.21	-14.44	-0.47	-10.24
Bosnia and Herzegovina	Gallbladder and biliary tract cancer	-0.74	-14.77	-0.3	-17.85	-0.43	-12.86
Botswana	Gallbladder and biliary tract cancer	0	-5.02	0	-7.3	0	-3.77
Brazil	Gallbladder and biliary tract cancer	-14.87	-20.86	-6.04	-27.7	-8.62	-17.44
Brunei Darussalam	Gallbladder and biliary tract cancer	-0.02	-20.36	-0.01	-24.09	-0.01	-18.26
Bulgaria	Gallbladder and biliary tract cancer	-1.3	-28.41	-0.47	-29.74	-0.91	-30.19
Burkina Faso	Gallbladder and biliary tract cancer	0	-4.78	0	-4.72	0	-4.81

Burundi	Gallbladder and biliary tract cancer	-0.04	-5.68	-0.02	-8.36	-0.02	-4.5
Cabo Verde	Gallbladder and biliary tract cancer	0	-26.25	0	-28.93	0	-25.07
Cambodia	Gallbladder and biliary tract cancer	-0.24	-17.42	-0.11	-20.17	-0.13	-15.59
Cameroon	Gallbladder and biliary tract cancer	0	-6.22	0	-6.92	0	-5.92
Canada	Gallbladder and biliary tract cancer	-7.54	-55.23	-3.57	-65.92	-3.84	-46.65
Central African Republic	Gallbladder and biliary tract cancer	0	-1.31	0	-2.44	0	-0.88
Chad	Gallbladder and biliary tract cancer	0	-2.2	0	-2.56	0	-2.1
Chile	Gallbladder and biliary tract cancer	-21.6	-50.13	-6.75	-65.8	-14.62	-44.54
China	Gallbladder and biliary tract cancer	-600.67	-132.79	-337.84	-152.6	-259.36	-112.3
Colombia	Gallbladder and biliary tract cancer	-6.68	-33.79	-2.32	-42.56	-4.28	-29.92
Comoros	Gallbladder and biliary tract cancer	0	-6.36	0	-8.08	0	-5.68
Congo	Gallbladder and biliary tract cancer	-0.01	-9.4	-0.01	-13.7	-0.01	-7.31
Cook Islands	Gallbladder and biliary tract cancer	0	-38.5	0	-50.19	0	-32.67
Costa Rica	Gallbladder and biliary tract cancer	-0.42	-27.53	-0.17	-35.53	-0.24	-22.94
Coted'Ivoire	Gallbladder and biliary tract cancer	0	-8.4	0	-8.8	0	-7.92
Croatia	Gallbladder and biliary tract cancer	-3.18	-64.57	-1.18	-73.71	-1.99	-59.95
Cuba	Gallbladder and biliary tract cancer	-0.85	-20.77	-0.38	-25.85	-0.45	-17.29
Cyprus	Gallbladder and biliary tract cancer	-0.42	-100.53	-0.2	-121.3	-0.22	-85.38
Czechia	Gallbladder and biliary tract cancer	-9.86	-43.12	-3.95	-53.51	-5.75	-37.12
Democratic People's Republic of Korea	Gallbladder and biliary tract cancer	-1.48	-19.9	-0.74	-23.73	-0.74	-17.04

Democratic Republic of the	Gallbladder and biliary tract cancer	-0.06	-5.22	-0.03	-6.77	-0.04	-4.47
Denmark	Gallbladder and biliary tract cancer	-2.21	-87.9	-1	-112.22	-1.06	-65.28
Djibouti	Gallbladder and biliary tract cancer	0	-9.02	0	-11.98	0	-7.17
Dominica	Gallbladder and biliary tract cancer	0	-4.3	0	-6.14	0	-2.89
Dominican Republic	Gallbladder and biliary tract cancer	-0.1	-9.95	-0.06	-11.23	-0.05	-8.73
Ecuador	Gallbladder and biliary tract cancer	-1.28	-20.69	-0.48	-26.44	-0.78	-17.73
Egypt	Gallbladder and biliary tract cancer	-2.02	-26.98	-1.09	-29.85	-0.94	-24.52
El Salvador	Gallbladder and biliary tract cancer	-0.49	-22.26	-0.15	-26.19	-0.35	-21.05
Equatorial Guinea	Gallbladder and biliary tract cancer	0	-23.33	0	-30.8	0	-19.16
Eritrea	Gallbladder and biliary tract cancer	-0.03	-8.74	-0.01	-11.3	-0.02	-7.73
Estonia	Gallbladder and biliary tract cancer	-0.33	-33.19	-0.1	-28.01	-0.23	-36.31
Eswatini	Gallbladder and biliary tract cancer	0	-0.31	0	-1.2	0	0.31
Ethiopia	Gallbladder and biliary tract cancer	-1.13	-11.14	-0.46	-14	-0.67	-9.76
Fiji	Gallbladder and biliary tract cancer	-0.01	-6.58	0	-7.87	0	-5.68
Finland	Gallbladder and biliary tract cancer	-2.72	-57.01	-1.05	-73.29	-1.61	-48.2
France	Gallbladder and biliary tract cancer	-23.21	-55.05	-11.41	-67.54	-11.34	-44.85
Gabon	Gallbladder and biliary tract cancer	-0.01	-8.29	0	-11.21	0	-6.57
Gambia	Gallbladder and biliary tract cancer	0	-5.73	0	-6.66	0	-4.7
Georgia	Gallbladder and biliary tract cancer	-0.02	-0.71	-0.02	-1.45	-0.01	-0.42
Germany	Gallbladder and biliary tract cancer	-75.69	-58.7	-33.4	-89.02	-41.51	-45.41

Ghana	Gallbladder and biliary tract cancer	-0.01	-9.47	0	-9.24	0	-9.66
Greece	Gallbladder and biliary tract cancer	-1.61	-26.75	-0.93	-34.83	-0.58	-17.37
Greenland	Gallbladder and biliary tract cancer	-0.01	-23.74	0	-27.76	0	-20.35
Grenada	Gallbladder and biliary tract cancer	0	-9.98	0	-12.43	0	-7.6
Guam	Gallbladder and biliary tract cancer	0	-13.7	0	-15.55	0	-11.47
Guatemala	Gallbladder and biliary tract cancer	-0.49	-11.46	-0.18	-13.93	-0.31	-10.3
Guinea	Gallbladder and biliary tract cancer	0	-2.98	0	-2.68	0	-3.52
Guinea-Bissau	Gallbladder and biliary tract cancer	0	-4.53	0	-4.76	0	-3.86
Guyana	Gallbladder and biliary tract cancer	-0.01	-5.41	0	-6.44	0	-4.67
Haiti	Gallbladder and biliary tract cancer	-0.07	-3.88	-0.03	-4.64	-0.04	-3.45
Honduras	Gallbladder and biliary tract cancer	-0.11	-8.31	-0.04	-9.8	-0.08	-7.9
Hungary	Gallbladder and biliary tract cancer	-4.05	-17.11	-1.48	-23.91	-2.53	-14.51
Iceland	Gallbladder and biliary tract cancer	-0.07	-64.41	-0.03	-82.84	-0.03	-47.83
India	Gallbladder and biliary tract cancer	-40.93	-20.1	-18.43	-22.83	-22.84	-18.59
Indonesia	Gallbladder and biliary tract cancer	-2.95	-14	-1.37	-16.48	-1.54	-12.08
Iran (Islamic Republic of)	Gallbladder and biliary tract cancer	-2.62	-79.1	-1.28	-104.15	-1.29	-62.27
Iraq	Gallbladder and biliary tract cancer	-0.86	-38.87	-0.41	-45.12	-0.45	-34.15
Ireland	Gallbladder and biliary tract cancer	-1.09	-71.26	-0.49	-89.08	-0.58	-59.34
Israel	Gallbladder and biliary tract cancer	-1.14	-60.74	-0.48	-87.36	-0.61	-46.25
Italy	Gallbladder and biliary tract cancer	-33.8	-49	-14.88	-59.59	-19.35	-43.97

Jamaica	Gallbladder and biliary tract cancer	-0.05	-8.31	-0.02	-10.53	-0.02	-6.41
Japan	Gallbladder and biliary tract cancer	-130.47	-47.11	-69.17	-55.52	-60.83	-39.92
Jordan	Gallbladder and biliary tract cancer	-0.47	-79.94	-0.22	-107.05	-0.24	-63.07
Kazakhstan	Gallbladder and biliary tract cancer	-0.35	-8.3	-0.16	-10.26	-0.19	-7.19
Kenya	Gallbladder and biliary tract cancer	-0.09	-4.7	-0.02	-4.78	-0.07	-4.68
Kiribati	Gallbladder and biliary tract cancer	0	-3.95	0	-4.85	0	-3.73
Kuwait	Gallbladder and biliary tract cancer	-0.25	-99.85	-0.15	-119.66	-0.09	-76.05
Kyrgyzstan	Gallbladder and biliary tract cancer	-0.06	-6.92	-0.03	-9.24	-0.03	-5.59
Lao People's Democratic	Gallbladder and biliary tract cancer	-0.07	-11.04	-0.03	-13.05	-0.04	-9.7
Latvia	Gallbladder and biliary tract cancer	-0.16	-15.72	-0.05	-13.92	-0.11	-17.29
Lebanon	Gallbladder and biliary tract cancer	-0.59	-62.88	-0.3	-77.91	-0.29	-51.83
Lesotho	Gallbladder and biliary tract cancer	0.01	6.09	0	5.84	0	6.2
Liberia	Gallbladder and biliary tract cancer	0	-8.53	0	-8.79	0	-8.87
Libya	Gallbladder and biliary tract cancer	-0.41	-25.49	-0.14	-31.37	-0.27	-23.1
Lithuania	Gallbladder and biliary tract cancer	-0.55	-28.34	-0.06	-11.18	-0.51	-36.88
Luxembourg	Gallbladder and biliary tract cancer	-0.17	-65.39	-0.08	-88.5	-0.09	-50.5
Madagascar	Gallbladder and biliary tract cancer	-0.03	-3.29	-0.01	-4.33	-0.02	-2.87
Malawi	Gallbladder and biliary tract cancer	-0.01	-5.38	0	-6.16	-0.01	-5.32
Malaysia	Gallbladder and biliary tract cancer	-1.11	-37.75	-0.61	-42.89	-0.49	-32.67
Maldives	Gallbladder and biliary tract cancer	-0.01	-59.01	-0.01	-71.03	-0.01	-49.95

Mali	Gallbladder and biliary tract cancer	-0.01	-5.79	-0.01	-6.45	-0.01	-4.98
Malta	Gallbladder and biliary tract cancer	-0.09	-65.94	-0.04	-83.5	-0.04	-52.15
Marshall Islands	Gallbladder and biliary tract cancer	0	-5.25	0	-7.77	0	-3.76
Mauritania	Gallbladder and biliary tract cancer	0	-12.97	0	-13.84	0	-12.81
Mauritius	Gallbladder and biliary tract cancer	-0.06	-20.16	-0.03	-20.54	-0.03	-19.87
Mexico	Gallbladder and biliary tract cancer	-8.05	-16.31	-2.88	-20.8	-5.07	-14.3
Micronesia (Federated States of)	Gallbladder and biliary tract cancer	0	-6.46	0	-7.2	0	-5.98
Monaco	Gallbladder and biliary tract cancer	0	-37.95	0	-48.75	0	-25.77
Mongolia	Gallbladder and biliary tract cancer	-0.07	-6.97	-0.03	-9.78	-0.05	-6.57
Montenegro	Gallbladder and biliary tract cancer	-0.01	-5.84	-0.01	-8.14	-0.01	-4.23
Morocco	Gallbladder and biliary tract cancer	-0.41	-17.65	-0.12	-21.63	-0.29	-16.24
Mozambique	Gallbladder and biliary tract cancer	-0.01	-1.09	0	-0.79	-0.01	-1.37
Myanmar	Gallbladder and biliary tract cancer	-0.82	-13.12	-0.36	-15.4	-0.46	-11.78
Namibia	Gallbladder and biliary tract cancer	-0.01	-8	0	-10.6	0	-7.06
Nauru	Gallbladder and biliary tract cancer	0	-4.78	0	-4.78	0	-4.84
Nepal	Gallbladder and biliary tract cancer	-0.66	-15.09	-0.25	-16.72	-0.41	-14.21
Netherlands	Gallbladder and biliary tract cancer	-8.05	-74.28	-3.6	-94.89	-3.71	-52.64
New Zealand	Gallbladder and biliary tract cancer	-2.04	-151.21	-0.91	-179.49	-0.85	-101.77
Nicaragua	Gallbladder and biliary tract cancer	-0.23	-18.81	-0.07	-24.22	-0.16	-16.99
Niger	Gallbladder and biliary tract cancer	0	-5.38	0	-5.16	0	-5.76

Nigeria	Gallbladder and biliary tract cancer	-0.03	-6.84	-0.01	-8.02	-0.02	-6.41
Niue	Gallbladder and biliary tract cancer	0	-9.36	0	-10.99	0	-8.27
North Macedonia	Gallbladder and biliary tract cancer	-0.2	-16.18	-0.07	-19.09	-0.12	-14.8
Northern Mariana Islands	Gallbladder and biliary tract cancer	0	-19	0	-24.68	0	-16.13
Norway	Gallbladder and biliary tract cancer	-2.12	-104.2	-1.08	-136.51	-0.98	-79.26
Oman	Gallbladder and biliary tract cancer	-0.1	-63.78	-0.06	-78.32	-0.04	-47.91
Pakistan	Gallbladder and biliary tract cancer	-2.09	-4.96	-0.58	-5.9	-1.58	-4.87
Palau	Gallbladder and biliary tract cancer	0	-16.29	0	-17.23	0	-12.65
Palestine	Gallbladder and biliary tract cancer	-0.11	-33.27	-0.06	-41.87	-0.05	-25.82
Panama	Gallbladder and biliary tract cancer	-0.21	-23.1	-0.09	-27.34	-0.12	-20.45
Papua New Guinea	Gallbladder and biliary tract cancer	-0.02	-5.18	-0.01	-5.84	-0.01	-4.63
Paraguay	Gallbladder and biliary tract cancer	-0.17	-16.17	-0.06	-21.3	-0.1	-13.58
Peru	Gallbladder and biliary tract cancer	-5.51	-40.41	-1.99	-48.37	-3.5	-36.76
Philippines	Gallbladder and biliary tract cancer	-0.42	-10.27	-0.25	-11.32	-0.17	-9.07
Poland	Gallbladder and biliary tract cancer	-5.12	-9.94	-2	-16.01	-2.9	-7.42
Portugal	Gallbladder and biliary tract cancer	-6.71	-91.08	-3.58	-114.57	-3	-70.78
Puerto Rico	Gallbladder and biliary tract cancer	-0.41	-34.76	-0.23	-40.73	-0.18	-28.24
Qatar	Gallbladder and biliary tract cancer	-0.13	-247.34	-0.08	-310.62	-0.04	-171.29
Republic of Korea	Gallbladder and biliary tract cancer	-85.07	-120.46	-47.09	-131.51	-38.04	-109.27
Republic of Moldova	Gallbladder and biliary tract cancer	-0.17	-10.59	-0.05	-10.07	-0.12	-11.21

Romania	Gallbladder and biliary tract cancer	-2.61	-15.6	-1.18	-19.22	-1.43	-13.43
Russian Federation	Gallbladder and biliary tract cancer	-49.21	-68.5	-21.74	-89.56	-24.9	-52.35
Rwanda	Gallbladder and biliary tract cancer	-0.09	-9.77	-0.03	-12.2	-0.06	-8.83
Saint Kitts and Nevis	Gallbladder and biliary tract cancer	0	-13.51	0	-16.8	0	-11.61
Saint Lucia	Gallbladder and biliary tract cancer	-0.01	-16.16	0	-19.91	0	-12.06
Saint Vincent and the Grenadines	Gallbladder and biliary tract cancer	0	-7.26	0	-8.7	0	-5.24
Samoa	Gallbladder and biliary tract cancer	0	-10.19	0	-10.59	0	-9.84
San Marino	Gallbladder and biliary tract cancer	-0.01	-31.38	0	-38.87	0	-21.52
Sao Tome and Principe	Gallbladder and biliary tract cancer	0	-8.97	0	-11.76	0	-8.05
Saudi Arabia	Gallbladder and biliary tract cancer	-2.06	-85.86	-1.04	-106.1	-1	-70.89
Senegal	Gallbladder and biliary tract cancer	0	-6.84	0	-7.2	0	-6.75
Serbia	Gallbladder and biliary tract cancer	-1.49	-20.9	-0.83	-33.45	-0.57	-12.26
Seychelles	Gallbladder and biliary tract cancer	0	-17.65	0	-21.65	0	-14.43
Sierra Leone	Gallbladder and biliary tract cancer	0	-4.72	0	-5.33	0	-4.41
Singapore	Gallbladder and biliary tract cancer	-0.98	-106.47	-0.47	-119.26	-0.5	-96.31
Slovakia	Gallbladder and biliary tract cancer	-4.13	-55.33	-1.53	-68.1	-2.58	-49.36
Slovenia	Gallbladder and biliary tract cancer	-1.79	-78.5	-0.71	-102.46	-1.07	-67.75
Solomon Islands	Gallbladder and biliary tract cancer	0	-6.77	0	-7.24	0	-6.46
Somalia	Gallbladder and biliary tract cancer	-0.01	-2.41	-0.01	-3.22	-0.01	-2
South Africa	Gallbladder and biliary tract cancer	-0.23	-6.57	-0.13	-9.05	-0.1	-4.61

South Sudan	Gallbladder and biliary tract cancer	-0.01	-2.38	-0.01	-2.92	0	-1.92
Spain	Gallbladder and biliary tract cancer	-26.13	-84.58	-12.15	-114.55	-13.06	-64.4
Sri Lanka	Gallbladder and biliary tract cancer	-2.01	-31.59	-0.94	-32.87	-1.07	-30.53
Sudan	Gallbladder and biliary tract cancer	-0.33	-15.53	-0.14	-21.63	-0.19	-12.59
Suriname	Gallbladder and biliary tract cancer	-0.01	-6.68	0	-7.99	0	-5.63
Sweden	Gallbladder and biliary tract cancer	2.62	19.03	-0.15	-3.37	2.64	28.54
Switzerland	Gallbladder and biliary tract cancer	-2.42	-82.93	-1.06	-95.57	-1.44	-79.61
Syrian Arab Republic	Gallbladder and biliary tract cancer	-0.04	-43.15	-0.01	-53.53	-0.03	-39.03
Taiwan (Province of China)	Gallbladder and biliary tract cancer	-10.34	-94.34	-6.21	-92.25	-4.11	-97.18
Tajikistan	Gallbladder and biliary tract cancer	0	0.79	0	0.12	0	1.12
Thailand	Gallbladder and biliary tract cancer	-40.18	-69.19	-25.05	-82.52	-15.01	-54.15
Timor-Leste	Gallbladder and biliary tract cancer	-0.01	-10.09	0	-10.4	0	-9.78
Togo	Gallbladder and biliary tract cancer	0	-6.63	0	-6.25	0	-7
Tokelau	Gallbladder and biliary tract cancer	0	-10.92	0	-11.28	0	-10.4
Tonga	Gallbladder and biliary tract cancer	0	-8.61	0	-9.18	0	-7.93
Trinidad and Tobago	Gallbladder and biliary tract cancer	-0.05	-12.78	-0.02	-15.22	-0.03	-11.06
Tunisia	Gallbladder and biliary tract cancer	-1.19	-44.4	-0.46	-53.1	-0.72	-39.89
Turkey	Gallbladder and biliary tract cancer	-8.96	-49.75	-4.57	-59.91	-4.31	-41.5
Turkmenistan	Gallbladder and biliary tract cancer	-0.04	-2.52	-0.02	-3.2	-0.02	-2.11
Tuvalu	Gallbladder and biliary tract cancer	0	-9.1	0	-11.3	0	-7.92

Uganda	Gallbladder and biliary tract cancer	-0.1	-8.83	-0.06	-9.81	-0.04	-7.73
Ukraine	Gallbladder and biliary tract cancer	-2.84	-14.37	-1.08	-16.09	-1.76	-13.43
United Arab Emirates	Gallbladder and biliary tract cancer	-0.46	-79.51	-0.26	-106.14	-0.19	-55.61
United Kingdom	Gallbladder and biliary tract cancer	-20.86	-94.28	-8.13	-96.26	-12.25	-89.58
United Republic of Tanzania	Gallbladder and biliary tract cancer	-0.14	-6.13	-0.06	-7.64	-0.08	-5.35
United States of America	Gallbladder and biliary tract cancer	-49.11	-54.99	-21.92	-64.4	-25.47	-46.09
United States Virgin Islands	Gallbladder and biliary tract cancer	0	-11	0	-12.02	0	-9.23
Uruguay	Gallbladder and biliary tract cancer	-1.08	-21.78	-0.4	-29.01	-0.67	-18.56
Uzbekistan	Gallbladder and biliary tract cancer	0.01	1.47	0	0.84	0.01	1.8
Vanuatu	Gallbladder and biliary tract cancer	0	-4.55	0	-5.11	0	-4.12
Venezuela (Bolivarian Republic	Gallbladder and biliary tract cancer	-1.3	-14.46	-0.49	-18.33	-0.79	-12.51
Viet Nam	Gallbladder and biliary tract cancer	-4.82	-47.28	-2.72	-54.26	-2.1	-40.51
Yemen	Gallbladder and biliary tract cancer	-0.14	-11.77	-0.06	-16.47	-0.08	-9.7
Zambia	Gallbladder and biliary tract cancer	-0.05	-7	-0.02	-7.73	-0.03	-6.87
Zimbabwe	Gallbladder and biliary tract cancer	0.05	4.96	0.02	3.81	0.03	5.6

Table S22. Absolute (the number) and relative contribution (the proportion) associated with disease severity changes for bladder cancer (BlaC), by sex at global, SDI regional, GBD regional level between 1990 and 2021.

Location	Cause	Attributable number (Both thousands)	Attributable proportion (Both %)	Attributable number (Male thousands)	Attributable proportion (Male %)	Attributable number (Female thousands)	Attributable proportion (Female %)
Global	Bladder cancer	-1443.24	-52.95	-1130.03	-55.79	-314.02	-44.85
High SDI	Bladder cancer	-474.9	-48.38	-364.52	-51.35	-111.6	-41.06
High-middle SDI	Bladder cancer	-619.27	-69.56	-496.13	-70.52	-124.31	-66.59
Middle SDI	Bladder cancer	-617.9	-126.42	-460.24	-130.39	-149.22	-109.89
Low-middle SDI	Bladder cancer	-152.15	-61.12	-114.1	-61.97	-38.09	-58.74
Low SDI	Bladder cancer	-53.67	-47.76	-34.34	-47.61	-19	-47.22
High-income Asia Pacific	Bladder cancer	-64.28	-68	-46.34	-67.97	-17.14	-65.02
High-income North America	Bladder cancer	-79.7	-28.97	-62.44	-32.27	-18.08	-22.14
Western Europe	Bladder cancer	-392.55	-54.27	-304.01	-55.48	-90.49	-51.61
Australasia	Bladder cancer	-6.98	-37.7	-5.01	-38.42	-1.85	-33.8
Eastern Europe	Bladder cancer	-91.9	-45.08	-76.15	-45.85	-16.1	-42.6
Central Europe	Bladder cancer	-112.34	-73.24	-86.8	-71.25	-24.87	-78.8
Southern Latin America	Bladder cancer	-22.6	-52.16	-17.33	-50.81	-5.36	-58.1
East Asia	Bladder cancer	-892.42	-154.36	-670.17	-160.44	-213.77	-133.25
Central Asia	Bladder cancer	-8.64	-34.41	-6.89	-35.44	-1.87	-33.05
North Africa and Middle East	Bladder cancer	-195.91	-128.69	-161.61	-132.95	-29.17	-95.07
Andean Latin America	Bladder cancer	-7.87	-120.77	-4.99	-118.04	-2.91	-127.02
Southeast Asia	Bladder cancer	-87.13	-117.25	-63.67	-118.38	-21.87	-106.59
Tropical Latin America	Bladder cancer	-37.84	-81.03	-26.19	-78.07	-11.82	-89.9

**Location**	**Cause**	**Attributable number (Both thousands)**	**Attributable proportion (Both %)**	**Attributable number (Male thousands)**	**Attributable proportion (Male %)**	**Attributable number (Female thousands)**	**Attributable proportion (Female %)**
Southern Sub-Saharan Africa	Bladder cancer	-2.83	-17.49	-1.89	-19.76	-0.86	-12.98
Caribbean	Bladder cancer	-7.91	-53.37	-5.53	-54	-2.34	-51.15
Central Latin America	Bladder cancer	-23.59	-86.92	-15.19	-84.94	-8.41	-90.91
South Asia	Bladder cancer	-139.85	-76.97	-105.81	-77.34	-33.88	-75.47
Central Sub-Saharan Africa	Bladder cancer	-6.16	-48.71	-4.18	-48.6	-1.95	-48.32
Oceania	Bladder cancer	-0.38	-44.72	-0.26	-41.91	-0.14	-60.94
Western Sub-Saharan Africa	Bladder cancer	-12.71	-39.58	-8.37	-38.1	-4.27	-42.1
Eastern Sub-Saharan Africa	Bladder cancer	-22.72	-50.15	-12.49	-49.92	-10.13	-49.94
Afghanistan	Bladder cancer	-2.94	-48.14	-2.25	-57.22	-0.9	-41.26
Albania	Bladder cancer	-0.09	-69.45	-0.06	-67.53	-0.03	-75.49
Algeria	Bladder cancer	-4.59	-121.6	-3.64	-125.55	-0.87	-99.87
American Samoa	Bladder cancer	0	-42.87	0	-44.7	0	-73.9
Andorra	Bladder cancer	-0.04	-55.47	-0.04	-55.74	0	-48.44
Angola	Bladder cancer	-1.66	-77.28	-1.16	-75.82	-0.5	-79.88
Antigua and Barbuda	Bladder cancer	-0.01	-41.07	-0.01	-40.52	-0.01	-42.3
Argentina	Bladder cancer	-13.12	-40.74	-10.51	-40.38	-2.69	-43.45
Armenia	Bladder cancer	-1.64	-54.11	-1.4	-54.24	-0.24	-54.77
Australia	Bladder cancer	-5.9	-38.52	-4.24	-39.04	-1.54	-34.6
Austria	Bladder cancer	-6.55	-56.47	-4.72	-59.1	-1.79	-49.55
Azerbaijan	Bladder cancer	-1.29	-49.85	-1.07	-50.61	-0.24	-48.98
Bahamas	Bladder cancer	-0.02	-41.61	-0.02	-41.67	-0.01	-41.59

Location	Cause	Attributable number (Both thousands)	Attributable proportion (Both %)	Attributable number (Male thousands)	Attributable proportion (Male %)	Attributable number (Female thousands)	Attributable proportion (Female %)
Bahrain	Bladder cancer	-0.46	-183.02	-0.39	-189.27	-0.07	-155.85
Bangladesh	Bladder cancer	-15.53	-109.92	-12.2	-108.9	-3.32	-113.74
Barbados	Bladder cancer	-0.06	-40.65	-0.04	-40.47	-0.02	-40.75
Belarus	Bladder cancer	-4.74	-48.66	-3.94	-48.71	-0.82	-49.29
Belgium	Bladder cancer	-9.48	-47.41	-7.4	-49.92	-2.25	-43.53
Belize	Bladder cancer	-0.02	-48.87	-0.01	-46.02	-0.01	-52.69
Benin	Bladder cancer	-0.38	-40.27	-0.27	-39.2	-0.12	-42.88
Bermuda	Bladder cancer	-0.08	-93.98	-0.06	-98.37	-0.02	-82.26
Bhutan	Bladder cancer	-0.06	-98.61	-0.04	-100.07	-0.02	-93.47
Bolivia (Plurinational State of)	Bladder cancer	-1.37	-89.17	-0.85	-87.47	-0.52	-93.14
Bosnia and Herzegovina	Bladder cancer	-2.06	-73.87	-1.57	-73.07	-0.5	-78.03
Botswana	Bladder cancer	-0.11	-37.74	-0.07	-37.14	-0.04	-38.32
Brazil	Bladder cancer	-37.51	-81.13	-25.95	-78.16	-11.73	-89.99
Brunei Darussalam	Bladder cancer	-0.04	-68.39	-0.03	-68.43	-0.01	-69.42
Bulgaria	Bladder cancer	-4.61	-43.62	-3.67	-42.84	-0.95	-47.3
Burkina Faso	Bladder cancer	-0.54	-26.96	-0.34	-25.47	-0.19	-28.72
Burundi	Bladder cancer	-0.53	-32.52	-0.33	-34.74	-0.2	-29.49
Cabo Verde	Bladder cancer	-0.01	-50.55	0	-20.18	0	-96.11
Cambodia	Bladder cancer	-1.7	-117.21	-1.17	-119.65	-0.5	-106.68
Cameroon	Bladder cancer	-0.99	-41.88	-0.71	-41.18	-0.27	-41.21

Location	Cause	Attributable number (Both thousands)	Attributable proportion (Both %)	Attributable number (Male thousands)	Attributable proportion (Male %)	Attributable number (Female thousands)	Attributable proportion (Female %)
Canada	Bladder cancer	-15.17	-44.47	-12.29	-47.88	-3.01	-35.62
Central African Republic	Bladder cancer	-0.09	-12.09	-0.06	-11.8	-0.03	-12.08
Chad	Bladder cancer	-0.21	-18.04	-0.13	-16.48	-0.07	-19
Chile	Bladder cancer	-6.36	-99.99	-4.28	-99.92	-2.07	-100.07
China	Bladder cancer	-873.7	-156.52	-659.48	-163.35	-206.12	-133.41
Colombia	Bladder cancer	-7.77	-110.14	-5.11	-107.08	-2.69	-117.59
Comoros	Bladder cancer	-0.04	-41.24	-0.02	-39.45	-0.02	-43.33
Congo	Bladder cancer	-0.47	-61.83	-0.34	-64.3	-0.14	-56.91
Cook Islands	Bladder cancer	-0.01	-104.69	-0.01	-105.46	0	-102.01
Costa Rica	Bladder cancer	-0.53	-68.23	-0.38	-65.91	-0.16	-74.92
Coted'Ivoire	Bladder cancer	-0.91	-46.9	-0.56	-45.97	-0.35	-48.1
Croatia	Bladder cancer	-4.5	-72.91	-3.52	-75.53	-1	-66.77
Cuba	Bladder cancer	-4.24	-56.46	-3.1	-55.81	-1.13	-58.08
Cyprus	Bladder cancer	-1.18	-137.26	-0.98	-141.84	-0.2	-118.13
Czechia	Bladder cancer	-13.52	-85.18	-10.27	-83.59	-3.2	-89.48
Democratic People's Republic of Korea	Bladder cancer	-5.53	-75.55	-3.68	-74.3	-1.72	-72.91
Democratic Republic of the Congo	Bladder cancer	-3.48	-41.48	-2.28	-40.65	-1.18	-42.2
Denmark	Bladder cancer	-4.81	-54.46	-3.52	-57.15	-1.32	-49.2
Djibouti	Bladder cancer	-0.05	-67.9	-0.03	-69.66	-0.02	-64.27
Dominica	Bladder cancer	-0.01	-20.09	0	-20.18	0	-19.31

Location	Cause	Attributable number (Both thousands)	Attributable proportion (Both %)	Attributable number (Male thousands)	Attributable proportion (Male %)	Attributable number (Female thousands)	Attributable proportion (Female %)
Dominican Republic	Bladder cancer	-0.44	-58.21	-0.28	-56.45	-0.16	-60.31
Ecuador	Bladder cancer	-1.44	-111.47	-0.88	-108.2	-0.57	-117.78
Egypt	Bladder cancer	-42.04	-74.26	-34.46	-75.96	-5.91	-52.51
El Salvador	Bladder cancer	-0.57	-105.17	-0.3	-97.54	-0.28	-117.81
Equatorial Guinea	Bladder cancer	-0.18	-149.87	-0.14	-160.52	-0.05	-138.57
Eritrea	Bladder cancer	-0.33	-45.65	-0.18	-46.86	-0.15	-43.94
Estonia	Bladder cancer	-1.3	-72.96	-0.91	-73.43	-0.36	-65.86
Eswatini	Bladder cancer	-0.03	-14.59	-0.01	-13.38	-0.01	-14.58
Ethiopia	Bladder cancer	-9.7	-74.83	-5.49	-75.96	-4.21	-73.39
Fiji	Bladder cancer	-0.04	-37.64	-0.03	-33.12	-0.02	-51.99
Finland	Bladder cancer	-3.41	-61.04	-2.65	-64.98	-0.84	-55.93
France	Bladder cancer	-63.73	-60.76	-51.14	-62.44	-13.36	-58.14
Gabon	Bladder cancer	-0.23	-53.54	-0.18	-54.48	-0.06	-50.28
Gambia	Bladder cancer	-0.03	-32.11	-0.02	-29.82	-0.01	-38.36
Georgia	Bladder cancer	-0.84	-14.53	-0.67	-15.68	-0.25	-16.04
Germany	Bladder cancer	-75.32	-54.95	-52.53	-55.07	-22.25	-53.38
Ghana	Bladder cancer	-1.58	-45.07	-1.14	-43.04	-0.43	-50.28
Greece	Bladder cancer	-6.84	-29.4	-5.92	-30.54	-1.01	-25.99
Greenland	Bladder cancer	-0.02	-45.4	-0.01	-49.4	0	-44.55
Grenada	Bladder cancer	-0.02	-40.29	-0.01	-42.21	-0.01	-40.67
Guam	Bladder cancer	-0.01	-49.23	-0.01	-47.57	0	-80.78

Location	Cause	Attributable number (Both thousands)	Attributable proportion (Both %)	Attributable number (Male thousands)	Attributable proportion (Male %)	Attributable number (Female thousands)	Attributable proportion (Female %)
Guatemala	Bladder cancer	-0.69	-97.4	-0.38	-94.05	-0.31	-102.94
Guinea	Bladder cancer	-0.48	-24.29	-0.35	-22.92	-0.12	-26.68
Guinea-Bissau	Bladder cancer	-0.08	-30.64	-0.06	-30.22	-0.02	-31.18
Guyana	Bladder cancer	-0.05	-37.66	-0.03	-37.32	-0.02	-38.16
Haiti	Bladder cancer	-0.71	-35.3	-0.44	-34.82	-0.27	-36.18
Honduras	Bladder cancer	-0.15	-68.16	-0.09	-64.7	-0.06	-76.2
Hungary	Bladder cancer	-11.96	-69.25	-8.53	-65.66	-3.33	-77.84
Iceland	Bladder cancer	-0.14	-49.47	-0.1	-50.11	-0.04	-48.32
India	Bladder cancer	-99.68	-86.33	-75.36	-86.74	-24.15	-84.47
Indonesia	Bladder cancer	-19.68	-82.61	-13.66	-82.18	-5.62	-78.08
Iran (Islamic Republic of)	Bladder cancer	-17.64	-143.71	-14.33	-147.27	-2.85	-112.03
Iraq	Bladder cancer	-10.69	-141.98	-8.01	-148.55	-2.38	-111.39
Ireland	Bladder cancer	-2.76	-76.24	-2.03	-78.86	-0.75	-71.53
Israel	Bladder cancer	-3.59	-80.59	-2.88	-82.93	-0.72	-72.83
Italy	Bladder cancer	-78.4	-58.04	-65.6	-59.24	-14.05	-57.72
Jamaica	Bladder cancer	-0.28	-31.91	-0.16	-31.88	-0.11	-31.46
Japan	Bladder cancer	-42.78	-55.65	-30.09	-54.91	-12.14	-54.97
Jordan	Bladder cancer	-1.88	-212.88	-1.55	-217.97	-0.26	-149.5
Kazakhstan	Bladder cancer	-3.38	-44.4	-2.74	-45.64	-0.69	-42.09
Kenya	Bladder cancer	-0.55	-36.4	-0.23	-30.43	-0.31	-40.64
Kiribati	Bladder cancer	0	-44.03	0	-40.61	0	-48.81

Location	Cause	Attributable number (Both thousands)	Attributable proportion (Both %)	Attributable number (Male thousands)	Attributable proportion (Male %)	Attributable number (Female thousands)	Attributable proportion (Female %)
Kuwait	Bladder cancer	-0.59	-186.62	-0.41	-186.48	-0.1	-105.63
Kyrgyzstan	Bladder cancer	-0.54	-45.57	-0.44	-44.4	-0.09	-48.6
Lao People's Democratic Republic	Bladder cancer	-0.66	-89.68	-0.49	-91.25	-0.16	-80.97
Latvia	Bladder cancer	-1.51	-48.95	-1.16	-49.55	-0.37	-48.7
Lebanon	Bladder cancer	-6.32	-155.65	-5.09	-161.33	-1.13	-125.3
Lesotho	Bladder cancer	0.03	8.52	0.02	11.54	0.01	5.94
Liberia	Bladder cancer	-0.28	-50.82	-0.2	-50.14	-0.08	-53.73
Libya	Bladder cancer	-2.07	-100.67	-1.72	-103.07	-0.28	-73.51
Lithuania	Bladder cancer	-1.31	-31.54	-0.96	-29.13	-0.34	-39.11
Luxembourg	Bladder cancer	-0.46	-70.75	-0.35	-72.27	-0.12	-68.92
Madagascar	Bladder cancer	-0.71	-28.48	-0.37	-27.1	-0.34	-30.36
Malawi	Bladder cancer	-2.75	-46.52	-1.38	-46.6	-1.34	-45.1
Malaysia	Bladder cancer	-6.41	-141.21	-4.8	-141.01	-1.56	-137.19
Maldives	Bladder cancer	-0.05	-190.34	-0.04	-202.12	-0.01	-187.62
Mali	Bladder cancer	-2.88	-41.74	-1.95	-41.55	-0.9	-41.06
Malta	Bladder cancer	-0.36	-67.09	-0.27	-66.29	-0.09	-69.86
Marshall Islands	Bladder cancer	0	-54.44	0	-54.79	0	-56.29
Mauritania	Bladder cancer	-0.33	-68.97	-0.22	-69.12	-0.11	-68.63
Mauritius	Bladder cancer	-0.47	-63.58	-0.31	-64.88	-0.14	-53.2
Mexico	Bladder cancer	-10.7	-77.83	-6.99	-77.11	-3.67	-78.38

Location	Cause	Attributable number (Both thousands)	Attributable proportion (Both %)	Attributable number (Male thousands)	Attributable proportion (Male %)	Attributable number (Female thousands)	Attributable proportion (Female %)
Micronesia (Federated States of)	Bladder cancer	-0.01	-56.82	-0.01	-55.73	0	-65.14
Monaco	Bladder cancer	-0.03	-34.05	-0.02	-36.01	-0.01	-29.92
Mongolia	Bladder cancer	-0.37	-61.24	-0.2	-69.23	-0.18	-56.83
Montenegro	Bladder cancer	-0.12	-23.58	-0.08	-21.82	-0.04	-28.47
Morocco	Bladder cancer	-3.44	-98.29	-3.04	-99.68	-0.36	-79.5
Mozambique	Bladder cancer	-0.83	-25.34	-0.4	-22.81	-0.4	-26.97
Myanmar	Bladder cancer	-7.03	-89.63	-4.68	-92.17	-2.18	-78.94
Namibia	Bladder cancer	-0.11	-53.97	-0.06	-54.33	-0.05	-52.91
Nauru	Bladder cancer	0	-35.94	0	-34.83	0	-49.59
Nepal	Bladder cancer	-2.1	-90.47	-1.47	-92.75	-0.63	-84.06
Netherlands	Bladder cancer	-12.08	-52.58	-10.05	-56.15	-2.45	-48.14
New Zealand	Bladder cancer	-1.08	-33.72	-0.81	-36.91	-0.31	-31
Nicaragua	Bladder cancer	-0.18	-96.53	-0.1	-93.59	-0.08	-101.42
Niger	Bladder cancer	-0.33	-34.14	-0.22	-32.36	-0.12	-39.42
Nigeria	Bladder cancer	-2.05	-35.67	-1.11	-32.84	-0.99	-41.56
Niue	Bladder cancer	0	-43.99	0	-44.04	0	-44.27
North Macedonia	Bladder cancer	-1.19	-65.65	-0.99	-65.5	-0.2	-64.31
Northern Mariana Islands	Bladder cancer	0	-61.18	0	-60.14	0	-165.31
Norway	Bladder cancer	-3.97	-51.82	-3.03	-54.44	-0.97	-46.61
Oman	Bladder cancer	-0.34	-137.45	-0.24	-141.61	-0.08	-109.29

Location	Cause	Attributable number (Both thousands)	Attributable proportion (Both %)	Attributable number (Male thousands)	Attributable proportion (Male %)	Attributable number (Female thousands)	Attributable proportion (Female %)
Pakistan	Bladder cancer	-18.36	-36.93	-13.27	-35.77	-5.12	-40.56
Palau	Bladder cancer	0	-57.27	0	-57.31	0	-55.09
Palestine	Bladder cancer	-0.77	-112.71	-0.64	-115.99	-0.1	-78.89
Panama	Bladder cancer	-0.29	-87.48	-0.18	-85.52	-0.11	-91.18
Papua New Guinea	Bladder cancer	-0.22	-46.65	-0.15	-44.2	-0.08	-59.15
Paraguay	Bladder cancer	-0.32	-69.16	-0.24	-67.92	-0.08	-73.35
Peru	Bladder cancer	-4.88	-132.38	-3.15	-128.97	-1.74	-140.16
Philippines	Bladder cancer	-3.13	-53.77	-2.2	-51.93	-0.95	-60.11
Poland	Bladder cancer	-48.15	-94.84	-37.77	-89.32	-9.17	-107.98
Portugal	Bladder cancer	-13.62	-100.24	-10.46	-104.48	-3.09	-86.49
Puerto Rico	Bladder cancer	-1.49	-74.01	-0.97	-74.32	-0.51	-72.89
Qatar	Bladder cancer	-0.33	-314.64	-0.28	-321.19	-0.05	-281.52
Republic of Korea	Bladder cancer	-32.98	-197.95	-25.55	-201.84	-7.25	-181.07
Republic of Moldova	Bladder cancer	-1.81	-55.68	-1.46	-56.12	-0.35	-54.52
Romania	Bladder cancer	-19.14	-77.85	-14.99	-78.22	-4.2	-77.38
Russian Federation	Bladder cancer	-71.27	-52.29	-58.51	-52.61	-12.81	-51.11
Rwanda	Bladder cancer	-1.25	-61.2	-0.66	-60.12	-0.59	-62.11
Saint Kitts and Nevis	Bladder cancer	-0.02	-70.95	-0.01	-73.03	-0.01	-67.8
Saint Lucia	Bladder cancer	-0.04	-65.17	-0.03	-67.95	-0.02	-60.95
Saint Vincent and the Grenadines	Bladder cancer	-0.01	-33.44	-0.01	-33.67	0	-31.47

Location	Cause	Attributable number (Both thousands)	Attributable proportion (Both %)	Attributable number (Male thousands)	Attributable proportion (Male %)	Attributable number (Female thousands)	Attributable proportion (Female %)
Samoa	Bladder cancer	-0.01	-50.74	-0.01	-50.94	0	-46.95
San Marino	Bladder cancer	-0.02	-39.64	-0.02	-42.13	0	-34.56
Sao Tome and Principe	Bladder cancer	-0.03	-57.28	-0.02	-59.41	-0.01	-51.42
Saudi Arabia	Bladder cancer	-3.78	-168.13	-2.98	-168.7	-0.7	-145.68
Senegal	Bladder cancer	-0.69	-41.6	-0.49	-40.74	-0.2	-43.81
Serbia	Bladder cancer	-8.66	-72.96	-6.49	-71.22	-2.17	-79.08
Seychelles	Bladder cancer	-0.04	-70.98	-0.03	-71.92	-0.01	-69.27
Sierra Leone	Bladder cancer	-0.28	-29.57	-0.2	-29.19	-0.07	-31.62
Singapore	Bladder cancer	-1.2	-128.44	-0.88	-127.5	-0.33	-132.31
Slovakia	Bladder cancer	-3.92	-61.3	-3.16	-62.73	-0.76	-56.36
Slovenia	Bladder cancer	-1.8	-80.87	-1.38	-83.04	-0.48	-84.41
Solomon Islands	Bladder cancer	-0.03	-59.32	-0.02	-57.31	-0.01	-80.64
Somalia	Bladder cancer	-0.22	-15.37	-0.1	-14.01	-0.11	-16.63
South Africa	Bladder cancer	-2.78	-28.11	-1.78	-29.23	-0.95	-24.92
South Sudan	Bladder cancer	-0.27	-19.78	-0.17	-18.56	-0.1	-22.55
Spain	Bladder cancer	-48.09	-59.25	-40.54	-60.09	-7.91	-57.69
Sri Lanka	Bladder cancer	-2.95	-129.31	-2.35	-126.35	-0.68	-160.09
Sudan	Bladder cancer	-5.54	-93.99	-4.32	-97.58	-0.99	-68.04
Suriname	Bladder cancer	-0.03	-40.04	-0.02	-38.76	-0.01	-42.9
Sweden	Bladder cancer	-5.33	-44.3	-3.89	-46.66	-1.47	-39.82
Switzerland	Bladder cancer	-4.02	-69.26	-3.25	-80.11	-0.78	-44.52

**Location**	**Cause**	**Attributable number (Both thousands)**	**Attributable proportion (Both %)**	**Attributable number (Male thousands)**	**Attributable proportion (Male %)**	**Attributable number (Female thousands)**	**Attributable proportion (Female %)**
Syrian Arab Republic	Bladder cancer	-3.24	-127.49	-2.82	-133.13	-0.35	-84.66
Taiwan (Province of China)	Bladder cancer	-15.38	-121.94	-10.58	-116.91	-4.9	-137.4
Tajikistan	Bladder cancer	-0.12	-20.9	-0.09	-19.86	-0.03	-25.4
Thailand	Bladder cancer	-27.77	-150.95	-21.07	-152.37	-6.36	-139.15
Timor-Leste	Bladder cancer	-0.06	-93.38	-0.04	-91.08	-0.02	-91.14
Togo	Bladder cancer	-0.24	-40.02	-0.15	-37.21	-0.08	-44.64
Tokelau	Bladder cancer	0	-57.35	0	-56.19	0	-63.56
Tonga	Bladder cancer	-0.01	-43.77	0	-42.78	0	-49.59
Trinidad and Tobago	Bladder cancer	-0.22	-59.14	-0.14	-60.26	-0.07	-55.79
Tunisia	Bladder cancer	-4.02	-119.67	-3.41	-122.04	-0.55	-97.76
Turkey	Bladder cancer	-62.54	-173.43	-54.19	-177.35	-7.1	-128.94
Turkmenistan	Bladder cancer	-0.28	-38.93	-0.2	-41.31	-0.09	-37.59
Tuvalu	Bladder cancer	0	-73.68	0	-74.06	0	-72.67
Uganda	Bladder cancer	-1.59	-41.8	-0.94	-40.31	-0.64	-43.82
Ukraine	Bladder cancer	-9.97	-21.89	-8.51	-22.82	-1.84	-22.28
United Arab Emirates	Bladder cancer	-1.33	-233.77	-1.15	-242.27	-0.16	-163.24
United Kingdom	Bladder cancer	-36.39	-29.69	-27.26	-31.81	-9.11	-24.7
United Republic of Tanzania	Bladder cancer	-2.3	-36.86	-1.23	-35.99	-1.07	-37.65
United States of America	Bladder cancer	-63.38	-26.3	-47.98	-28.6	-15.63	-21.35
United States Virgin Islands	Bladder cancer	-0.01	-34.09	-0.01	-31.33	0	-41.83
Uruguay	Bladder cancer	-1.9	-40.01	-1.52	-40.02	-0.39	-40.86

Location	Cause	Attributable number (Both thousands)	Attributable proportion (Both %)	Attributable number (Male thousands)	Attributable proportion (Male %)	Attributable number (Female thousands)	Attributable proportion (Female %)
Uzbekistan	Bladder cancer	-0.78	-26.57	-0.55	-24.36	-0.24	-33.78
Vanuatu	Bladder cancer	-0.01	-40.9	-0.01	-39.58	0	-57.9
Venezuela (Bolivarian Republic of)	Bladder cancer	-2.84	-79.69	-1.76	-75.63	-1.1	-88.46
Viet Nam	Bladder cancer	-12.37	-146.87	-9.69	-147.14	-2.51	-136.74
Yemen	Bladder cancer	-2.78	-90.16	-2.07	-93.31	-0.53	-61.13
Zambia	Bladder cancer	-1.04	-59.72	-0.58	-58.12	-0.45	-59.87
Zimbabwe	Bladder cancer	0.86	16.25	0.53	18.35	0.35	14.31

Table S23. Absolute (the number) and relative contribution (the proportion) associated with disease severity changes for ovarian cancer (OC), by sex at global, SDI regional, GBD regional level between 1990 and 2021.

Location	Cause	Attributable number (Female thousands)	Attributable proportion (Female %)
Global	Ovarian cancer	-456.42	-15.89
High SDI	Ovarian cancer	-212.26	-17.88
High-middle SDI	Ovarian cancer	-188.66	-22.95
Middle SDI	Ovarian cancer	-244.32	-48.93
Low-middle SDI	Ovarian cancer	-97.95	-37.9
Low SDI	Ovarian cancer	-32.88	-32.15
High-income Asia Pacific	Ovarian cancer	-53.29	-41.85
High-income North America	Ovarian cancer	-28.53	-7.37
Western Europe	Ovarian cancer	-117.64	-17.93
Australasia	Ovarian cancer	-4.56	-14.59
Eastern Europe	Ovarian cancer	-41.38	-12.95
Central Europe	Ovarian cancer	-31.21	-17.84
Southern Latin America	Ovarian cancer	-9.51	-22.52
East Asia	Ovarian cancer	-215.54	-52.95
Central Asia	Ovarian cancer	-3.85	-15.23
North Africa and Middle East	Ovarian cancer	-34.19	-52.34
Andean Latin America	Ovarian cancer	-5.77	-66.14
Southeast Asia	Ovarian cancer	-81.53	-55.72
Tropical Latin America	Ovarian cancer	-18.93	-31.75
Southern Sub-Saharan Africa	Ovarian cancer	-1.76	-10.58
Caribbean	Ovarian cancer	-2.77	-21.27

Location	Cause	Attributable number (Female thousands)	Attributable proportion (Female %)
Central Latin America	Ovarian cancer	-23.53	-45.16
South Asia	Ovarian cancer	-108.57	-42.06
Central Sub-Saharan Africa	Ovarian cancer	-2.75	-31.45
Oceania	Ovarian cancer	-0.23	-29.75
Western Sub-Saharan Africa	Ovarian cancer	-6.4	-28.56
Eastern Sub-Saharan Africa	Ovarian cancer	-16.53	-33.11
Afghanistan	Ovarian cancer	-0.65	-30.26
Albania	Ovarian cancer	-0.2	-27.35
Algeria	Ovarian cancer	-1.29	-48.38
American Samoa	Ovarian cancer	0	-26.77
Andorra	Ovarian cancer	0	-17.62
Angola	Ovarian cancer	-0.74	-56.96
Antigua and Barbuda	Ovarian cancer	-0.01	-19.15
Argentina	Ovarian cancer	-5.65	-17.59
Armenia	Ovarian cancer	-0.3	-15.88
Australia	Ovarian cancer	-3.97	-14.92
Austria	Ovarian cancer	-2.75	-16.82
Azerbaijan	Ovarian cancer	-0.42	-19.48
Bahamas	Ovarian cancer	-0.04	-20.06
Bahrain	Ovarian cancer	-0.14	-76.84
Bangladesh	Ovarian cancer	-11.43	-63.5
Barbados	Ovarian cancer	-0.04	-14.36
Belarus	Ovarian cancer	-1.9	-16.7

Location	Cause	Attributable number (Female thousands)	Attributable proportion (Female %)
Belgium	Ovarian cancer	-2.54	-12.52
Belize	Ovarian cancer	-0.01	-30.36
Benin	Ovarian cancer	-0.13	-28.76
Bermuda	Ovarian cancer	-0.02	-26.56
Bhutan	Ovarian cancer	-0.05	-49.24
Bolivia (Plurinational State of)	Ovarian cancer	-0.8	-56.83
Bosnia and Herzegovina	Ovarian cancer	-0.65	-18.62
Botswana	Ovarian cancer	-0.07	-22.21
Brazil	Ovarian cancer	-18.68	-31.76
Brunei Darussalam	Ovarian cancer	-0.1	-68.1
Bulgaria	Ovarian cancer	-0.95	-8.5
Burkina Faso	Ovarian cancer	-0.21	-19.42
Burundi	Ovarian cancer	-0.36	-20.2
Cabo Verde	Ovarian cancer	-0.01	-60.65
Cambodia	Ovarian cancer	-2.5	-93.27
Cameroon	Ovarian cancer	-0.36	-27.57
Canada	Ovarian cancer	-4.98	-16.7
Central African Republic	Ovarian cancer	-0.05	-10.14
Chad	Ovarian cancer	-0.07	-13.88
Chile	Ovarian cancer	-2.9	-44.27
China	Ovarian cancer	-201.58	-51.32
Colombia	Ovarian cancer	-6.75	-47.32
Comoros	Ovarian cancer	-0.04	-27.16

Location	Cause	Attributable number (Female thousands)	Attributable proportion (Female %)
Congo	Ovarian cancer	-0.21	-39.42
Cook Islands	Ovarian cancer	0	-49.67
Costa Rica	Ovarian cancer	-0.26	-39.37
Coted'Ivoire	Ovarian cancer	-0.52	-28.33
Croatia	Ovarian cancer	-1.38	-17.93
Cuba	Ovarian cancer	-1.01	-25.02
Cyprus	Ovarian cancer	-0.29	-38.19
Czechia	Ovarian cancer	-3.98	-24.82
Democratic People's Republic of Korea	Ovarian cancer	-3.15	-40.78
Democratic Republic of the Congo	Ovarian cancer	-1.6	-26.3
Denmark	Ovarian cancer	-2.6	-29.18
Djibouti	Ovarian cancer	-0.04	-37.4
Dominica	Ovarian cancer	0	-7.36
Dominican Republic	Ovarian cancer	-0.34	-27.83
Ecuador	Ovarian cancer	-1	-180.58
Egypt	Ovarian cancer	-3.44	-61.23
El Salvador	Ovarian cancer	-0.66	-53.83
Equatorial Guinea	Ovarian cancer	-0.07	-100.3
Eritrea	Ovarian cancer	-0.29	-30.74
Estonia	Ovarian cancer	-0.46	-17.46
Eswatini	Ovarian cancer	-0.02	-8.59
Ethiopia	Ovarian cancer	-6.55	-50.96
Fiji	Ovarian cancer	-0.03	-22.63

Location	Cause	Attributable number (Female thousands)	Attributable proportion (Female %)
Finland	Ovarian cancer	-1.84	-22.06
France	Ovarian cancer	-25.08	-28.83
Gabon	Ovarian cancer	-0.08	-29.54
Gambia	Ovarian cancer	-0.02	-24.8
Georgia	Ovarian cancer	-0.24	-10.8
Germany	Ovarian cancer	-21.81	-12.46
Ghana	Ovarian cancer	-0.65	-36.38
Greece	Ovarian cancer	-1.15	-9.03
Greenland	Ovarian cancer	-0.02	-22.95
Grenada	Ovarian cancer	-0.01	-20.58
Guam	Ovarian cancer	-0.01	-26.77
Guatemala	Ovarian cancer	-0.55	-71.92
Guinea	Ovarian cancer	-0.14	-16.65
Guinea-Bissau	Ovarian cancer	-0.03	-23.77
Guyana	Ovarian cancer	-0.08	-21.36
Haiti	Ovarian cancer	-0.57	-24.8
Honduras	Ovarian cancer	-0.6	-38.67
Hungary	Ovarian cancer	-2.9	-15.91
Iceland	Ovarian cancer	-0.05	-15.02
India	Ovarian cancer	-85.66	-43.42
Indonesia	Ovarian cancer	-29.73	-50.4
Iran (Islamic Republic of)	Ovarian cancer	-3.83	-59.27
Iraq	Ovarian cancer	-1.91	-57.75

Location	Cause	Attributable number (Female thousands)	Attributable proportion (Female %)
Ireland	Ovarian cancer	-1.37	-23.59
Israel	Ovarian cancer	-1.25	-23.21
Italy	Ovarian cancer	-14.3	-18.75
Jamaica	Ovarian cancer	-0.16	-13.92
Japan	Ovarian cancer	-40.89	-36.86
Jordan	Ovarian cancer	-0.52	-85.47
Kazakhstan	Ovarian cancer	-1.87	-16.72
Kenya	Ovarian cancer	-1.2	-30.91
Kiribati	Ovarian cancer	0	-32.84
Kuwait	Ovarian cancer	-0.17	-54.83
Kyrgyzstan	Ovarian cancer	-0.41	-24.44
Lao People's Democratic Republic	Ovarian cancer	-0.83	-75.4
Latvia	Ovarian cancer	-0.49	-12.35
Lebanon	Ovarian cancer	-0.64	-48.87
Lesotho	Ovarian cancer	0.02	3.78
Liberia	Ovarian cancer	-0.09	-38.52
Libya	Ovarian cancer	-0.43	-41.5
Lithuania	Ovarian cancer	-0.56	-10.2
Luxembourg	Ovarian cancer	-0.18	-20.84
Madagascar	Ovarian cancer	-0.67	-20.01
Malawi	Ovarian cancer	-0.47	-27.2
Malaysia	Ovarian cancer	-2.98	-64.39
Maldives	Ovarian cancer	-0.06	-121.41

Location	Cause	Attributable number (Female thousands)	Attributable proportion (Female %)
Mali	Ovarian cancer	-0.15	-23.63
Malta	Ovarian cancer	-0.12	-20.99
Marshall Islands	Ovarian cancer	0	-36.22
Mauritania	Ovarian cancer	-0.11	-44.13
Mauritius	Ovarian cancer	-0.22	-43.77
Mexico	Ovarian cancer	-12.8	-42.28
Micronesia (Federated States of)	Ovarian cancer	-0.01	-37.85
Monaco	Ovarian cancer	0	-8.54
Mongolia	Ovarian cancer	-0.26	-36.04
Montenegro	Ovarian cancer	-0.03	-6.41
Morocco	Ovarian cancer	-1.84	-40.67
Mozambique	Ovarian cancer	-0.66	-15.68
Myanmar	Ovarian cancer	-9.06	-56.69
Namibia	Ovarian cancer	-0.08	-27.23
Nauru	Ovarian cancer	0	-26.54
Nepal	Ovarian cancer	-1.91	-48.94
Netherlands	Ovarian cancer	-3.29	-12.95
New Zealand	Ovarian cancer	-0.6	-12.91
Nicaragua	Ovarian cancer	-0.24	-52.52
Niger	Ovarian cancer	-0.12	-25.05
Nigeria	Ovarian cancer	-3.27	-29.04
Niue	Ovarian cancer	0	-23.56
North Macedonia	Ovarian cancer	-0.39	-21.09

Location	Cause	Attributable number (Female thousands)	Attributable proportion (Female %)
Northern Mariana Islands	Ovarian cancer	0	-44.45
Norway	Ovarian cancer	-1.37	-17.49
Oman	Ovarian cancer	-0.08	-61.16
Pakistan	Ovarian cancer	-10.16	-26.17
Palau	Ovarian cancer	0	-29.71
Palestine	Ovarian cancer	-0.18	-47.22
Panama	Ovarian cancer	-0.25	-51.16
Papua New Guinea	Ovarian cancer	-0.15	-35.88
Paraguay	Ovarian cancer	-0.24	-30.49
Peru	Ovarian cancer	-4.04	-59.81
Philippines	Ovarian cancer	-8.71	-36.56
Poland	Ovarian cancer	-12.95	-20.13
Portugal	Ovarian cancer	-1.35	-14.18
Puerto Rico	Ovarian cancer	-0.41	-31.75
Qatar	Ovarian cancer	-0.12	-138.68
Republic of Korea	Ovarian cancer	-14.87	-105.21
Republic of Moldova	Ovarian cancer	-0.61	-12.57
Romania	Ovarian cancer	-5.77	-21.63
Russian Federation	Ovarian cancer	-33.23	-14.64
Rwanda	Ovarian cancer	-1.11	-40.19
Saint Kitts and Nevis	Ovarian cancer	-0.01	-30
Saint Lucia	Ovarian cancer	-0.03	-29.17
Saint Vincent and the Grenadines	Ovarian cancer	-0.01	-15.43

Location	Cause	Attributable number (Female thousands)	Attributable proportion (Female %)
Samoa	Ovarian cancer	-0.01	-29.84
San Marino	Ovarian cancer	0	-8.97
Sao Tome and Principe	Ovarian cancer	-0.01	-32.59
Saudi Arabia	Ovarian cancer	-1.64	-87.88
Senegal	Ovarian cancer	-0.23	-30.2
Serbia	Ovarian cancer	-2.18	-19.12
Seychelles	Ovarian cancer	-0.02	-41.41
Sierra Leone	Ovarian cancer	-0.08	-21.3
Singapore	Ovarian cancer	-1.47	-69.02
Slovakia	Ovarian cancer	-1.55	-21.16
Slovenia	Ovarian cancer	-0.45	-16.17
Solomon Islands	Ovarian cancer	-0.01	-55.49
Somalia	Ovarian cancer	-0.22	-13.73
South Africa	Ovarian cancer	-1.72	-13.7
South Sudan	Ovarian cancer	-0.19	-14.53
Spain	Ovarian cancer	-4.13	-9.91
Sri Lanka	Ovarian cancer	-2.94	-63
Sudan	Ovarian cancer	-0.93	-45.03
Suriname	Ovarian cancer	-0.04	-19.24
Sweden	Ovarian cancer	-0.01	-0.05
Switzerland	Ovarian cancer	-0.56	-7.8
Syrian Arab Republic	Ovarian cancer	-0.65	-47.08
Taiwan (Province of China)	Ovarian cancer	-4.86	-73.96

Location	Cause	Attributable number (Female thousands)	Attributable proportion (Female %)
Tajikistan	Ovarian cancer	-0.06	-7.64
Thailand	Ovarian cancer	-14.89	-67.1
Timor-Leste	Ovarian cancer	-0.08	-61.31
Togo	Ovarian cancer	-0.11	-30.53
Tokelau	Ovarian cancer	0	-34.54
Tonga	Ovarian cancer	0	-25.32
Trinidad and Tobago	Ovarian cancer	-0.21	-22.32
Tunisia	Ovarian cancer	-0.8	-41.16
Turkey	Ovarian cancer	-14.08	-50.57
Turkmenistan	Ovarian cancer	-0.18	-21.06
Tuvalu	Ovarian cancer	0	-41.65
Uganda	Ovarian cancer	-1.55	-33.13
Ukraine	Ovarian cancer	-4.89	-7.63
United Arab Emirates	Ovarian cancer	-0.51	-90.9
United Kingdom	Ovarian cancer	-30.02	-23.51
United Republic of Tanzania	Ovarian cancer	-1.96	-23.75
United States of America	Ovarian cancer	-23.98	-6.71
United States Virgin Islands	Ovarian cancer	-0.01	-14.71
Uruguay	Ovarian cancer	-0.6	-16.84
Uzbekistan	Ovarian cancer	-0.49	-12.81
Vanuatu	Ovarian cancer	0	-37.57
Venezuela (Bolivarian Republic of)	Ovarian cancer	-1.74	-72.97
Viet Nam	Ovarian cancer	-8.75	-77.14

Location	Cause	Attributable number (Female thousands)	Attributable proportion (Female %)
Yemen	Ovarian cancer	-0.38	-44.96
Zambia	Ovarian cancer	-0.79	-34.23
Zimbabwe	Ovarian cancer	0.12	4.54

Table S24. Absolute (the number) and relative contribution (the proportion) associated with disease severity changes for uterine cancer (UC), by sex at global, SDI regional, GBD regional level between 1990 and 2021.

Location	Cause	Attributable number (Female thousands)	Attributable proportion (Female %)
Global	Uterine cancer	-888.04	-59.15
High SDI	Uterine cancer	-215.77	-50.1
High-middle SDI	Uterine cancer	-370.9	-73.61
Middle SDI	Uterine cancer	-386.36	-104.97
Low-middle SDI	Uterine cancer	-100.04	-72.45
Low SDI	Uterine cancer	-33.77	-58
High-income Asia Pacific	Uterine cancer	-54.21	-101.63
High-income North America	Uterine cancer	-53.59	-36.24
Western Europe	Uterine cancer	-121.97	-57.06
Australasia	Uterine cancer	-4.87	-76.33
Eastern Europe	Uterine cancer	-113.12	-50.87
Central Europe	Uterine cancer	-67.78	-72.99
Southern Latin America	Uterine cancer	-11.49	-59.28
East Asia	Uterine cancer	-436.65	-123.68
Central Asia	Uterine cancer	-11.92	-39.92
North Africa and Middle East	Uterine cancer	-49	-147.37
Andean Latin America	Uterine cancer	-13.34	-104.67
Southeast Asia	Uterine cancer	-61.57	-69.11
Tropical Latin America	Uterine cancer	-28.58	-81.34
Southern Sub-Saharan Africa	Uterine cancer	-2.93	-39.23
Caribbean	Uterine cancer	-6.75	-41.89

Location	Cause	Attributable number (Female thousands)	Attributable proportion (Female %)
Central Latin America	Uterine cancer	-27.03	-111.06
South Asia	Uterine cancer	-92.29	-97.88
Central Sub-Saharan Africa	Uterine cancer	-4.2	-58.41
Oceania	Uterine cancer	-0.21	-13.99
Western Sub-Saharan Africa	Uterine cancer	-11.08	-64.44
Eastern Sub-Saharan Africa	Uterine cancer	-14.57	-60.08
Afghanistan	Uterine cancer	-1.41	-68.56
Albania	Uterine cancer	-0.69	-101.89
Algeria	Uterine cancer	-1.07	-139.07
American Samoa	Uterine cancer	0	-15.79
Andorra	Uterine cancer	-0.01	-53.89
Angola	Uterine cancer	-1.07	-94.35
Antigua and Barbuda	Uterine cancer	-0.01	-44.52
Argentina	Uterine cancer	-7.03	-47.05
Armenia	Uterine cancer	-1.14	-60.85
Australia	Uterine cancer	-3.79	-81.57
Austria	Uterine cancer	-2.86	-48.03
Azerbaijan	Uterine cancer	-1.55	-58.57
Bahamas	Uterine cancer	-0.04	-33.09
Bahrain	Uterine cancer	-0.13	-236.36
Bangladesh	Uterine cancer	-7.84	-119.44
Barbados	Uterine cancer	-0.07	-35.58
Belarus	Uterine cancer	-4.93	-74.98

Location	Cause	Attributable number (Female thousands)	Attributable proportion (Female %)
Belgium	Uterine cancer	-2.96	-51.26
Belize	Uterine cancer	-0.02	-39.86
Benin	Uterine cancer	-0.32	-61.71
Bermuda	Uterine cancer	-0.03	-79.77
Bhutan	Uterine cancer	-0.04	-99.77
Bolivia (Plurinational State of)	Uterine cancer	-2.23	-90.43
Bosnia and Herzegovina	Uterine cancer	-1.28	-80.51
Botswana	Uterine cancer	-0.11	-55.75
Brazil	Uterine cancer	-27.95	-81.96
Brunei Darussalam	Uterine cancer	-0.05	-108.76
Bulgaria	Uterine cancer	-2.98	-32.56
Burkina Faso	Uterine cancer	-0.47	-38.83
Burundi	Uterine cancer	-0.33	-35.94
Cabo Verde	Uterine cancer	-0.06	-72.08
Cambodia	Uterine cancer	-2.05	-83.54
Cameroon	Uterine cancer	-0.85	-56.61
Canada	Uterine cancer	-6.71	-51.88
Central African Republic	Uterine cancer	-0.07	-16.91
Chad	Uterine cancer	-0.19	-30.87
Chile	Uterine cancer	-3.31	-114.63
China	Uterine cancer	-426.48	-123.49
Colombia	Uterine cancer	-6.85	-142.64
Comoros	Uterine cancer	-0.04	-50.9

Location	Cause	Attributable number (Female thousands)	Attributable proportion (Female %)
Congo	Uterine cancer	-0.36	-73.39
Cook Islands	Uterine cancer	0	-63.63
Costa Rica	Uterine cancer	-0.52	-103.5
Coted'Ivoire	Uterine cancer	-0.43	-63.62
Croatia	Uterine cancer	-2.11	-62.24
Cuba	Uterine cancer	-3.15	-46.01
Cyprus	Uterine cancer	-0.44	-117.44
Czechia	Uterine cancer	-7.04	-63.68
Democratic People's Republic of Korea	Uterine cancer	-2.28	-38.76
Democratic Republic of the Congo	Uterine cancer	-2.44	-50.1
Denmark	Uterine cancer	-2.36	-57.12
Djibouti	Uterine cancer	-0.04	-79.38
Dominica	Uterine cancer	0	-10.31
Dominican Republic	Uterine cancer	-1.01	-48.48
Ecuador	Uterine cancer	-3.42	-78.25
Egypt	Uterine cancer	-5.48	-112.38
El Salvador	Uterine cancer	-1.1	-110.57
Equatorial Guinea	Uterine cancer	-0.11	-171.87
Eritrea	Uterine cancer	-0.32	-54.33
Estonia	Uterine cancer	-0.78	-60.03
Eswatini	Uterine cancer	-0.03	-29.66
Ethiopia	Uterine cancer	-4.73	-78.94
Fiji	Uterine cancer	-0.04	-16.63

Location	Cause	Attributable number (Female thousands)	Attributable proportion (Female %)
Finland	Uterine cancer	-2.33	-66.26
France	Uterine cancer	-22.85	-67.11
Gabon	Uterine cancer	-0.13	-66.33
Gambia	Uterine cancer	-0.04	-58.25
Georgia	Uterine cancer	-1.58	-22.94
Germany	Uterine cancer	-15.64	-28.01
Ghana	Uterine cancer	-1.94	-76.39
Greece	Uterine cancer	-1.68	-34.12
Greenland	Uterine cancer	0	-55.56
Grenada	Uterine cancer	-0.02	-38
Guam	Uterine cancer	0	-10.78
Guatemala	Uterine cancer	-1.87	-109.29
Guinea	Uterine cancer	-0.3	-35.97
Guinea-Bissau	Uterine cancer	-0.06	-44.33
Guyana	Uterine cancer	-0.09	-33.25
Haiti	Uterine cancer	-0.8	-26.46
Honduras	Uterine cancer	-1.33	-92.84
Hungary	Uterine cancer	-6.97	-65.22
Iceland	Uterine cancer	-0.06	-58.07
India	Uterine cancer	-69.41	-107.25
Indonesia	Uterine cancer	-23.1	-57.45
Iran (Islamic Republic of)	Uterine cancer	-4.15	-156.62
Iraq	Uterine cancer	-2.07	-162.66

Location	Cause	Attributable number (Female thousands)	Attributable proportion (Female %)
Ireland	Uterine cancer	-1.32	-83.22
Israel	Uterine cancer	-1.48	-93.05
Italy	Uterine cancer	-17.22	-127.79
Jamaica	Uterine cancer	-0.21	-27.14
Japan	Uterine cancer	-39.01	-96.22
Jordan	Uterine cancer	-0.7	-202.2
Kazakhstan	Uterine cancer	-4.68	-49.34
Kenya	Uterine cancer	-0.75	-59.4
Kiribati	Uterine cancer	-0.01	-22.09
Kuwait	Uterine cancer	-0.29	-300.48
Kyrgyzstan	Uterine cancer	-0.99	-54.63
Lao People's Democratic Republic	Uterine cancer	-0.72	-65.67
Latvia	Uterine cancer	-1.13	-49.57
Lebanon	Uterine cancer	-0.71	-127.98
Lesotho	Uterine cancer	0	-1.71
Liberia	Uterine cancer	-0.22	-75.73
Libya	Uterine cancer	-0.42	-105.14
Lithuania	Uterine cancer	-1.19	-44.8
Luxembourg	Uterine cancer	-0.24	-66.67
Madagascar	Uterine cancer	-0.66	-38.23
Malawi	Uterine cancer	-0.43	-52.45
Malaysia	Uterine cancer	-2.72	-88.91
Maldives	Uterine cancer	-0.02	-120.93

Location	Cause	Attributable number (Female thousands)	Attributable proportion (Female %)
Mali	Uterine cancer	-0.45	-48.27
Malta	Uterine cancer	-0.14	-73.97
Marshall Islands	Uterine cancer	0	-23.06
Mauritania	Uterine cancer	-0.31	-92.58
Mauritius	Uterine cancer	-0.26	-41.66
Mexico	Uterine cancer	-10.83	-117.28
Micronesia (Federated States of)	Uterine cancer	-0.01	-35.98
Monaco	Uterine cancer	0	-35.39
Mongolia	Uterine cancer	-0.42	-100.41
Montenegro	Uterine cancer	-0.11	-41.19
Morocco	Uterine cancer	-1.19	-115.48
Mozambique	Uterine cancer	-0.74	-34.2
Myanmar	Uterine cancer	-7.86	-63.15
Namibia	Uterine cancer	-0.13	-67.86
Nauru	Uterine cancer	0	-19.9
Nepal	Uterine cancer	-1.31	-95.78
Netherlands	Uterine cancer	-3.68	-52.45
New Zealand	Uterine cancer	-1.01	-58.56
Nicaragua	Uterine cancer	-0.28	-107.84
Niger	Uterine cancer	-0.35	-55.18
Nigeria	Uterine cancer	-3.66	-70.4
Niue	Uterine cancer	0	-26.86
North Macedonia	Uterine cancer	-1	-87.6

Location	Cause	Attributable number (Female thousands)	Attributable proportion (Female %)
Northern Mariana Islands	Uterine cancer	0	-28.87
Norway	Uterine cancer	-1.99	-69.95
Oman	Uterine cancer	-0.06	-156
Pakistan	Uterine cancer	-13.52	-62.61
Palau	Uterine cancer	0	-30.62
Palestine	Uterine cancer	-0.54	-112.51
Panama	Uterine cancer	-0.52	-127.57
Papua New Guinea	Uterine cancer	-0.12	-13.98
Paraguay	Uterine cancer	-0.64	-62.01
Peru	Uterine cancer	-7.65	-129.5
Philippines	Uterine cancer	-2.84	-26.5
Poland	Uterine cancer	-28.01	-104.64
Portugal	Uterine cancer	-5.79	-86.42
Puerto Rico	Uterine cancer	-0.89	-74.71
Qatar	Uterine cancer	-0.13	-406.51
Republic of Korea	Uterine cancer	-13.89	-115.8
Republic of Moldova	Uterine cancer	-1.43	-53.01
Romania	Uterine cancer	-11.27	-82.69
Russian Federation	Uterine cancer	-83.89	-52.97
Rwanda	Uterine cancer	-1.01	-68.73
Saint Kitts and Nevis	Uterine cancer	-0.02	-70.23
Saint Lucia	Uterine cancer	-0.03	-57.4
Saint Vincent and the Grenadines	Uterine cancer	-0.01	-22.82

Location	Cause	Attributable number (Female thousands)	Attributable proportion (Female %)
Samoa	Uterine cancer	-0.01	-32.5
San Marino	Uterine cancer	0	-30.24
Sao Tome and Principe	Uterine cancer	-0.02	-70.18
Saudi Arabia	Uterine cancer	-1.94	-290.53
Senegal	Uterine cancer	-0.55	-64.73
Serbia	Uterine cancer	-4.03	-65
Seychelles	Uterine cancer	-0.01	-44.11
Sierra Leone	Uterine cancer	-0.19	-48.33
Singapore	Uterine cancer	-1.17	-157.97
Slovakia	Uterine cancer	-3.88	-71.77
Slovenia	Uterine cancer	-1.12	-78.38
Solomon Islands	Uterine cancer	-0.02	-33.83
Somalia	Uterine cancer	-0.23	-22.67
South Africa	Uterine cancer	-2.62	-52.26
South Sudan	Uterine cancer	-0.23	-32.39
Spain	Uterine cancer	-14.76	-61.02
Sri Lanka	Uterine cancer	-2.34	-89.47
Sudan	Uterine cancer	-1.3	-112.46
Suriname	Uterine cancer	-0.03	-30.42
Sweden	Uterine cancer	-2.6	-47.08
Switzerland	Uterine cancer	-1.8	-43.19
Syrian Arab Republic	Uterine cancer	-1.21	-122.18
Taiwan (Province of China)	Uterine cancer	-2.55	-141.35

Location	Cause	Attributable number (Female thousands)	Attributable proportion (Female %)
Tajikistan	Uterine cancer	-0.47	-33.5
Thailand	Uterine cancer	-9.49	-103.72
Timor-Leste	Uterine cancer	-0.06	-56.22
Togo	Uterine cancer	-0.22	-66.04
Tokelau	Uterine cancer	0	-40.11
Tonga	Uterine cancer	-0.01	-20.74
Trinidad and Tobago	Uterine cancer	-0.31	-50.72
Tunisia	Uterine cancer	-0.72	-118.25
Turkey	Uterine cancer	-21.62	-151.23
Turkmenistan	Uterine cancer	-0.36	-47.72
Tuvalu	Uterine cancer	0	-43.48
Uganda	Uterine cancer	-1.62	-67.46
Ukraine	Uterine cancer	-11.31	-23.34
United Arab Emirates	Uterine cancer	-0.66	-238.83
United Kingdom	Uterine cancer	-14.04	-44.99
United Republic of Tanzania	Uterine cancer	-1.85	-47.94
United States of America	Uterine cancer	-47.18	-34.96
United States Virgin Islands	Uterine cancer	-0.01	-22.69
Uruguay	Uterine cancer	-0.7	-44.87
Uzbekistan	Uterine cancer	-1.91	-41.84
Vanuatu	Uterine cancer	0	-13.18
Venezuela (Bolivarian Republic of)	Uterine cancer	-3.75	-74.82
Viet Nam	Uterine cancer	-6.42	-99.68

Location	Cause	Attributable number (Female thousands)	Attributable proportion (Female %)
Yemen	Uterine cancer	-0.54	-94.33
Zambia	Uterine cancer	-0.86	-72.8
Zimbabwe	Uterine cancer	0.18	10.51

Table S25. Number of countries and territories with different relative contribution (the proportion) levels associated with disease severity changes for 20 types of cancer by sex at national level between 1990 and 2021.

Cancer	Sex	Relative contribution levels (number of countries/territories)				
		R< -100%	-100% ≤R< -60%	-60% ≤R< -20%	-20% ≤R< 0%	R≥ 0%
Nasopharynx cancer	both	6	13	100	83	2
	male	8	13	95	86	2
	female	4	19	106	73	2
Larynx cancer	both	9	22	123	48	2
	male	10	22	124	46	2
	female	6	28	112	54	4
Lip and oral cavity cancer	both	26	53	108	15	2
	male	21	47	112	22	2
	female	35	68	86	13	2
Thyroid cancer	both	83	81	39	1	
	male	96	69	37	1	1
	female	86	88	29	1	

**Cancer**	**Sex**	**Relative contribution levels (number of countries/territories)**				
		**R< -100%**	**-100% ≤R< -60%**	**-60% ≤R< -20%**	**-20% ≤R< 0%**	**R≥ 0%**
Tracheal, bronchus, and lung cancer	both	3	12	56	131	2
	male	2	9	55	134	4
	female	13	17	50	122	2
Mesothelioma	both	0	0	10	157	37
	male	0	0	6	86	112
	female	2	1	43	139	19
Oesophageal cancer	both	3	10	51	133	7
	male	3	10	50	135	6
	female	4	18	51	127	4
Stomach cancer	both	1	7	77	116	3
	male	1	12	74	114	3
	female	0	4	64	133	3
Colon and rectum cancer	both	43	51	95	13	2

Cancer	Sex	Relative contribution levels (number of countries/territories)				
		R< -100%	-100% ≤R< -60%	-60% ≤R< -20%	-20% ≤R< 0%	R≥ 0%
	male	46	54	87	15	2
	female	41	47	101	13	2
Liver cancer	both	6	11	34	146	7
	male	7	10	36	145	6
	female	4	8	38	148	6
Gallbladder and biliary tract cancer	both	8	25	52	114	5
	male	18	26	51	105	4
	female	5	15	55	123	6
Pancreatic cancer	both	0	4	30	167	3
	male	0	2	28	170	4
	female	0	4	34	164	2
Kidney cancer	both	77	73	40	14	
	male	79	72	40	13	

Cancer	Sex	Relative contribution levels (number of countries/territories)				
		R< -100%	-100% ≤R< -60%	-60% ≤R< -20%	-20% ≤R< 0%	R≥ 0%
	female	80	68	45	11	
Bladder cancer	both	35	58	103	6	2
	male	35	60	100	7	2
	female	34	59	103	6	2
Prostate cancer	male	100	67	35	1	1
Testicular cancer	male	164	28	11		1
Breast cancer	female	29	42	106	20	7
Ovarian cancer	female	5	20	120	57	2
Cervical cancer	female	9	38	143	12	2
Uterine cancer	female	43	63	88	9	1

Note: R= Relative contribution.

Figure S1. Changes in neoplasms-related DALYs attributable to disease severity in males and females from 1990 to 2021 with 1990 as the reference for each year.

Panel A. Absolute contribution in males at SDI regional level; **Panel B**. Relative contribution in males at SDI regional level; **Panel C**. Absolute contribution in females at SDI regional level; **Panel D**. Relative contribution in females at SDI regional level.

Note: DALYs = disability-adjusted life-years. GBD = Global Burden of Disease. SDI = Socio-demographic index.

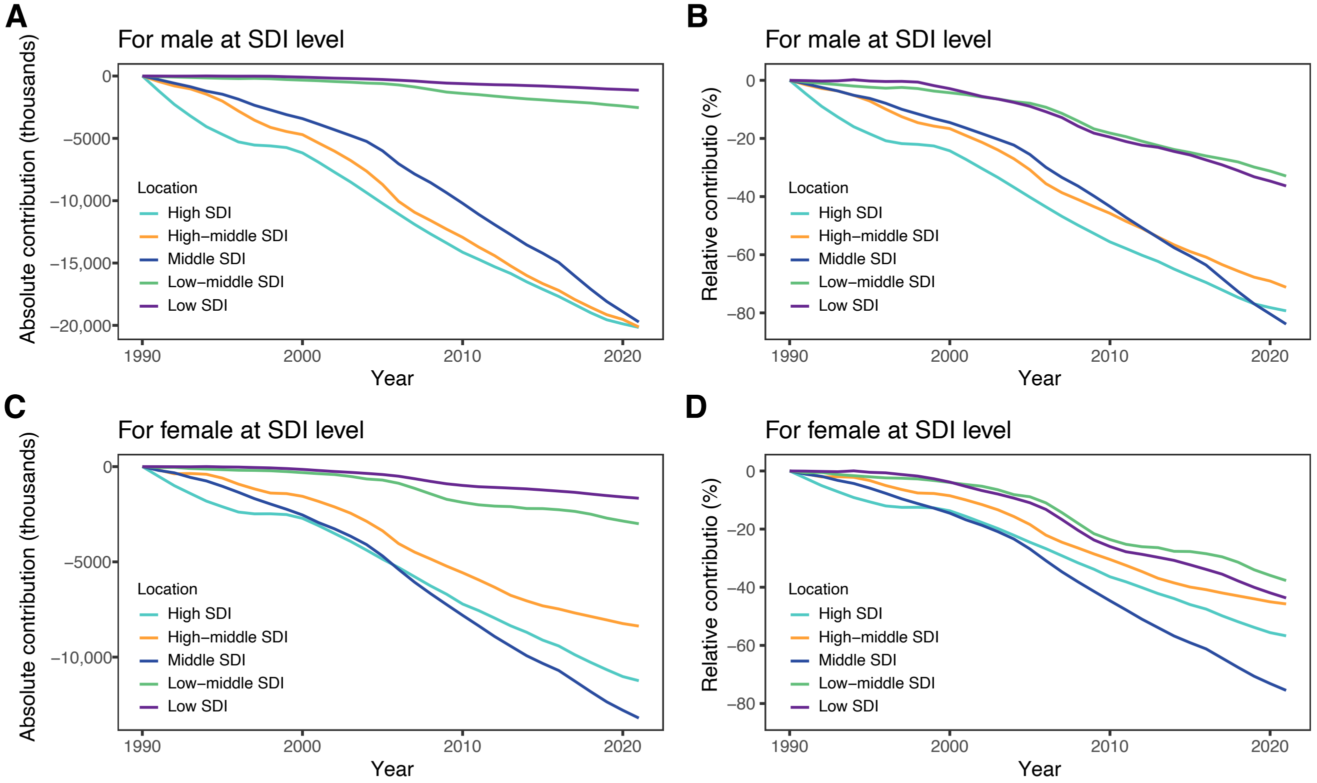

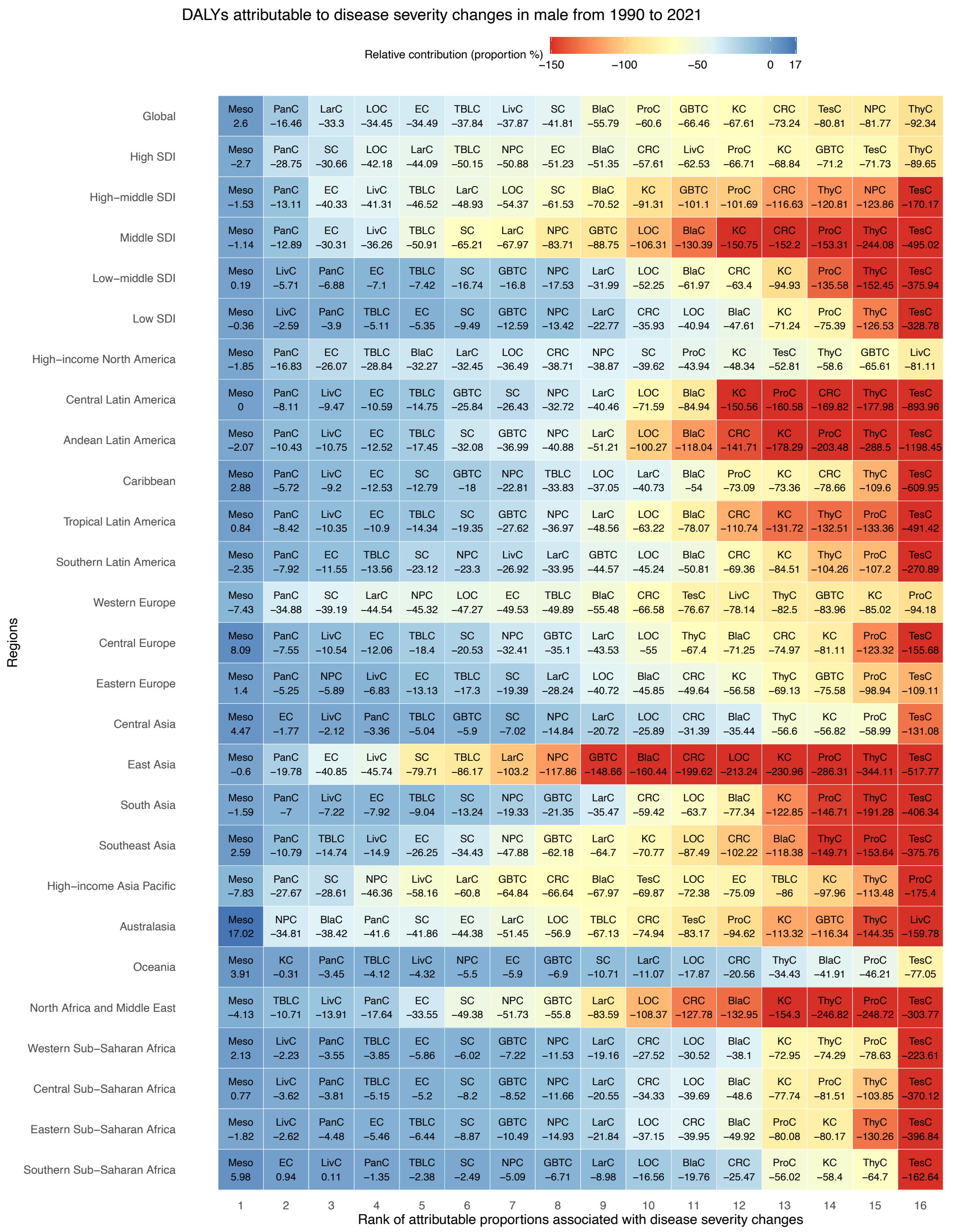

Figure S2. Relative contribution (the proportion %) associated with disease severity changes for 16 types of cancer in male between 1990 and 2021 at global, GBD regional, and SDI regional levels.

Note: NPC = Nasopharynx cancer. LarC = Larynx cancer. LOC = Lip and oral cavity cancer. ThyC = Thyroid cancer. TBLC = Tracheal, bronchus, and lung cancer. Meso = Mesothelioma. EC = Oesophageal cancer. SC = Stomach cancer. CRC = Colon and rectum cancer. LivC = Liver cancer. GBTC = Gallbladder and biliary tract cancer. PanC = Pancreatic cancer. KC = Kidney cancer. BlaC = Bladder cancer. ProC = Prostate cancer. TesC = Testicular cancer. BreC = Breast cancer. OC = Ovarian cancer. CC = Cervical cancer. UC = Uterine cancer. DALYs = disability-adjusted life-years. GBD = Global Burden of Disease. SDI = Socio-demographic index.

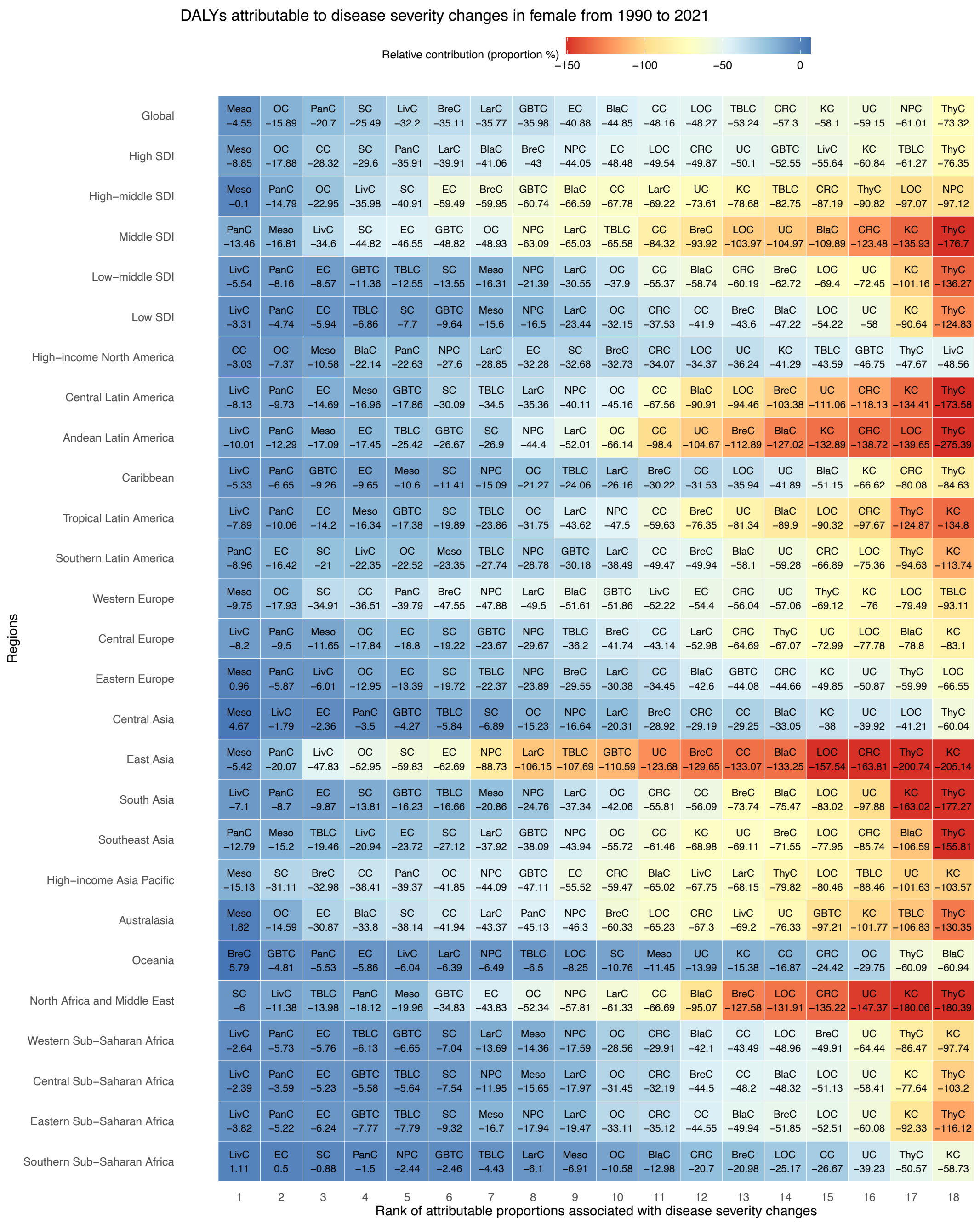

Figure S3. Relative contribution (the proportion %) associated with disease severity changes for 18 types of cancer in female between 1990 and 2021 at global, GBD regional, and SDI regional levels.

Note: NPC = Nasopharynx cancer. LarC = Larynx cancer. LOC = Lip and oral cavity cancer. ThyC = Thyroid cancer. TBLC = Tracheal, bronchus, and lung cancer. Meso = Mesothelioma. EC = Oesophageal cancer. SC = Stomach cancer. CRC = Colon and rectum cancer. LivC = Liver cancer. GBTC = Gallbladder and biliary tract cancer. PanC = Pancreatic cancer. KC = Kidney cancer. BlaC = Bladder cancer. ProC = Prostate cancer. TesC = Testicular cancer. BreC = Breast cancer. OC = Ovarian cancer. CC = Cervical cancer. UC = Uterine cancer. DALYs = disability-adjusted life-years. GBD = Global Burden of Disease. SDI = Socio-demographic index.

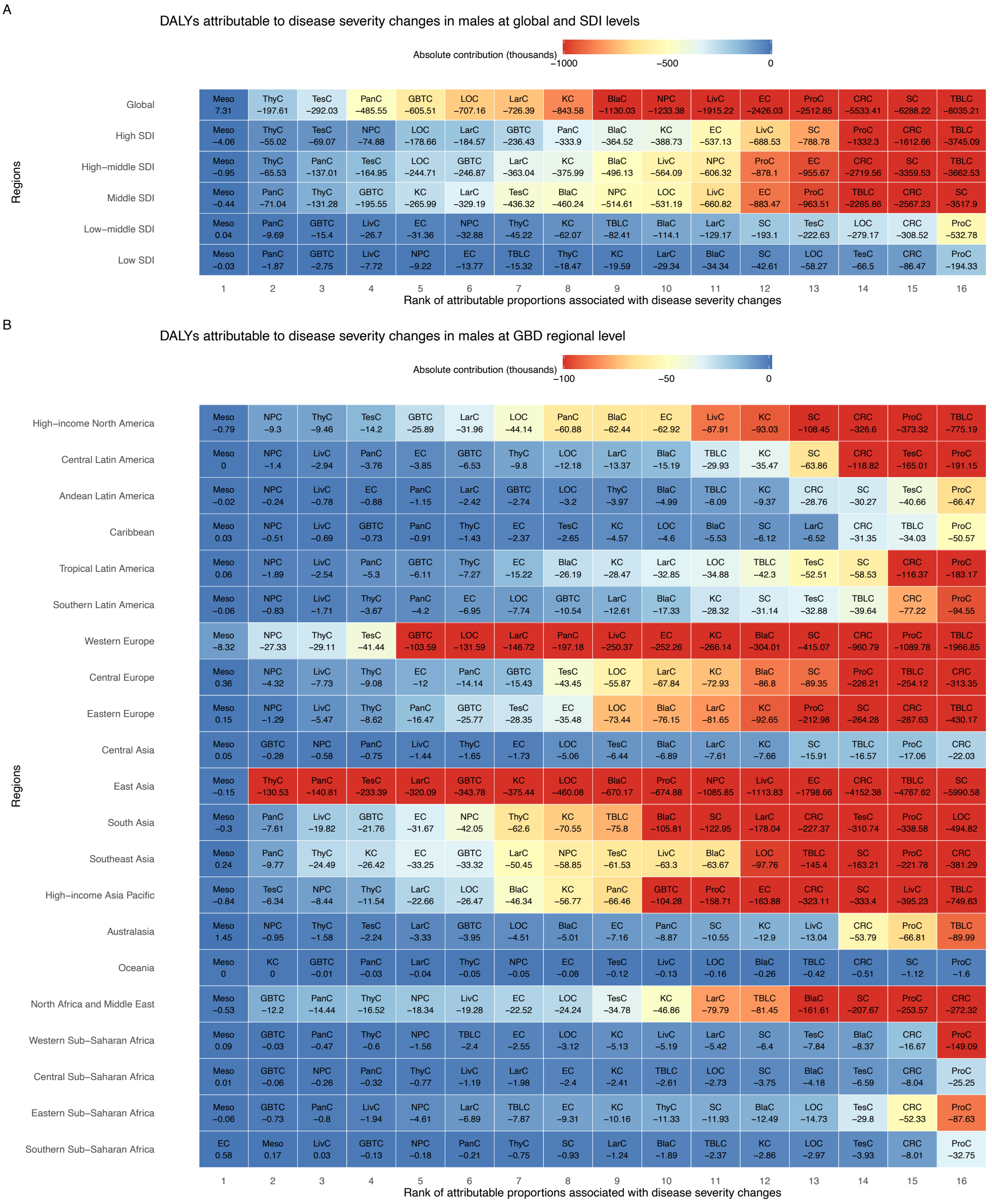

Figure S4. Absolute contribution (the number) associated with disease severity changes for 16 types of cancer in male between 1990 and 2021 at global, GBD regional, and SDI regional levels.

Note: NPC = Nasopharynx cancer. LarC = Larynx cancer. LOC = Lip and oral cavity cancer. ThyC = Thyroid cancer. TBLC = Tracheal, bronchus, and lung cancer. Meso = Mesothelioma. EC = Oesophageal cancer. SC = Stomach cancer. CRC = Colon and rectum cancer. LivC = Liver cancer. GBTC = Gallbladder and biliary tract cancer. PanC = Pancreatic cancer. KC = Kidney cancer. BlaC = Bladder cancer. ProC = Prostate cancer. TesC = Testicular cancer. BreC = Breast cancer. OC = Ovarian cancer. CC = Cervical cancer. UC = Uterine cancer. DALYs = disability-adjusted life-years. GBD = Global Burden of Disease. SDI = Socio-demographic index.

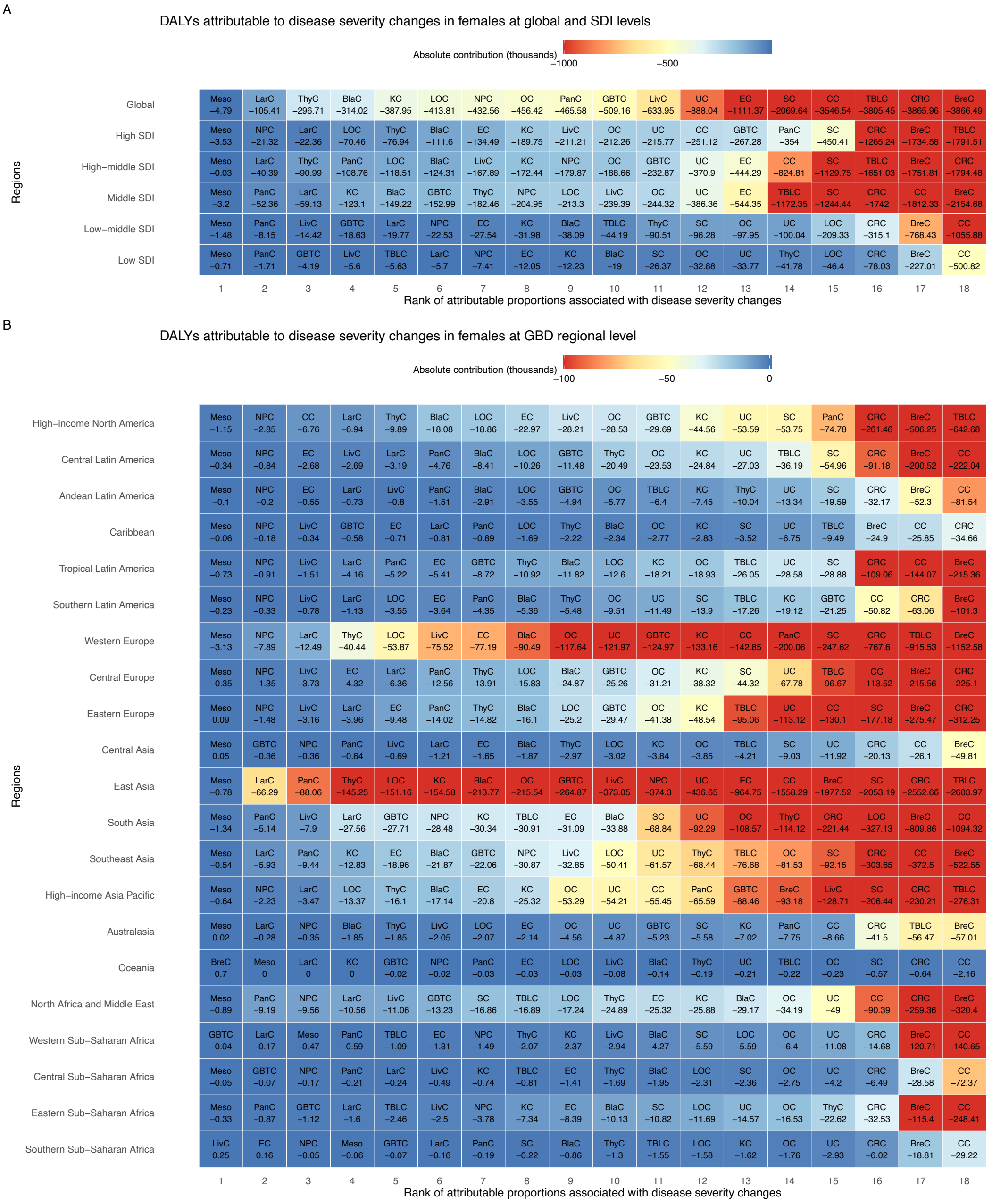

Figure S5. Absolute contribution (the number) associated with disease severity changes for 18 types of cancer in female between 1990 and 2021 at global, GBD regional, and SDI regional levels.

Note: NPC = Nasopharynx cancer. LarC = Larynx cancer. LOC = Lip and oral cavity cancer. ThyC = Thyroid cancer. TBLC = Tracheal, bronchus, and lung cancer. Meso = Mesothelioma. EC = Oesophageal cancer. SC = Stomach cancer. CRC = Colon and rectum cancer. LivC = Liver cancer. GBTC = Gallbladder and biliary tract cancer. PanC = Pancreatic cancer. KC = Kidney cancer. BlaC = Bladder cancer. ProC = Prostate cancer. TesC = Testicular cancer. BreC = Breast cancer. OC = Ovarian cancer. CC = Cervical cancer. UC = Uterine cancer. DALYs = disability-adjusted life-years. GBD = Global Burden of Disease. SDI = Socio-demographic index.

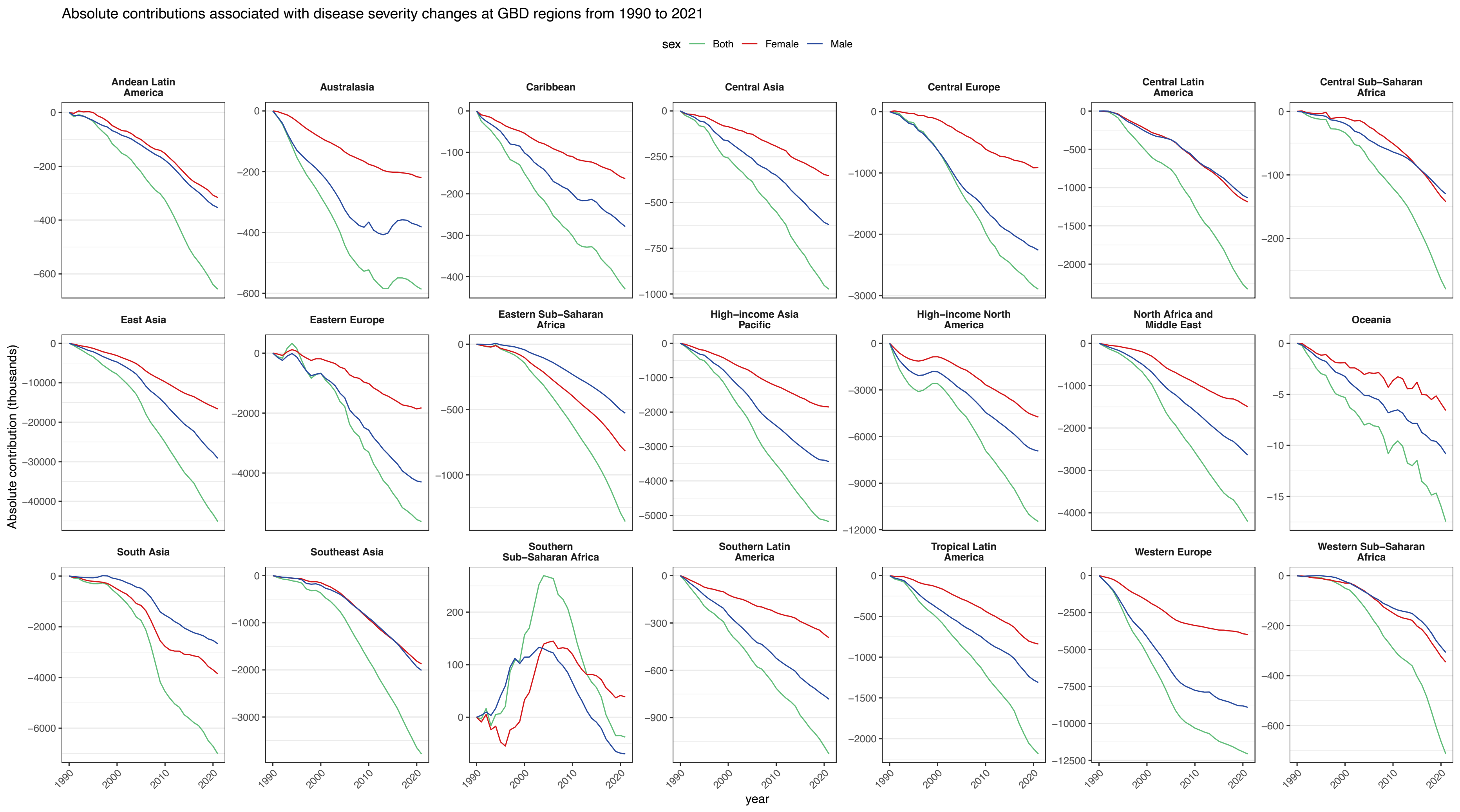

Figure S6. Absolute contribution (the number) associated with disease severity changes across 21 GBD regions between 1990 and 2021.

Figure S7. Relative contribution (the proportion %) associated with disease severity changes across 21 GBD regions between 1990 and 2021

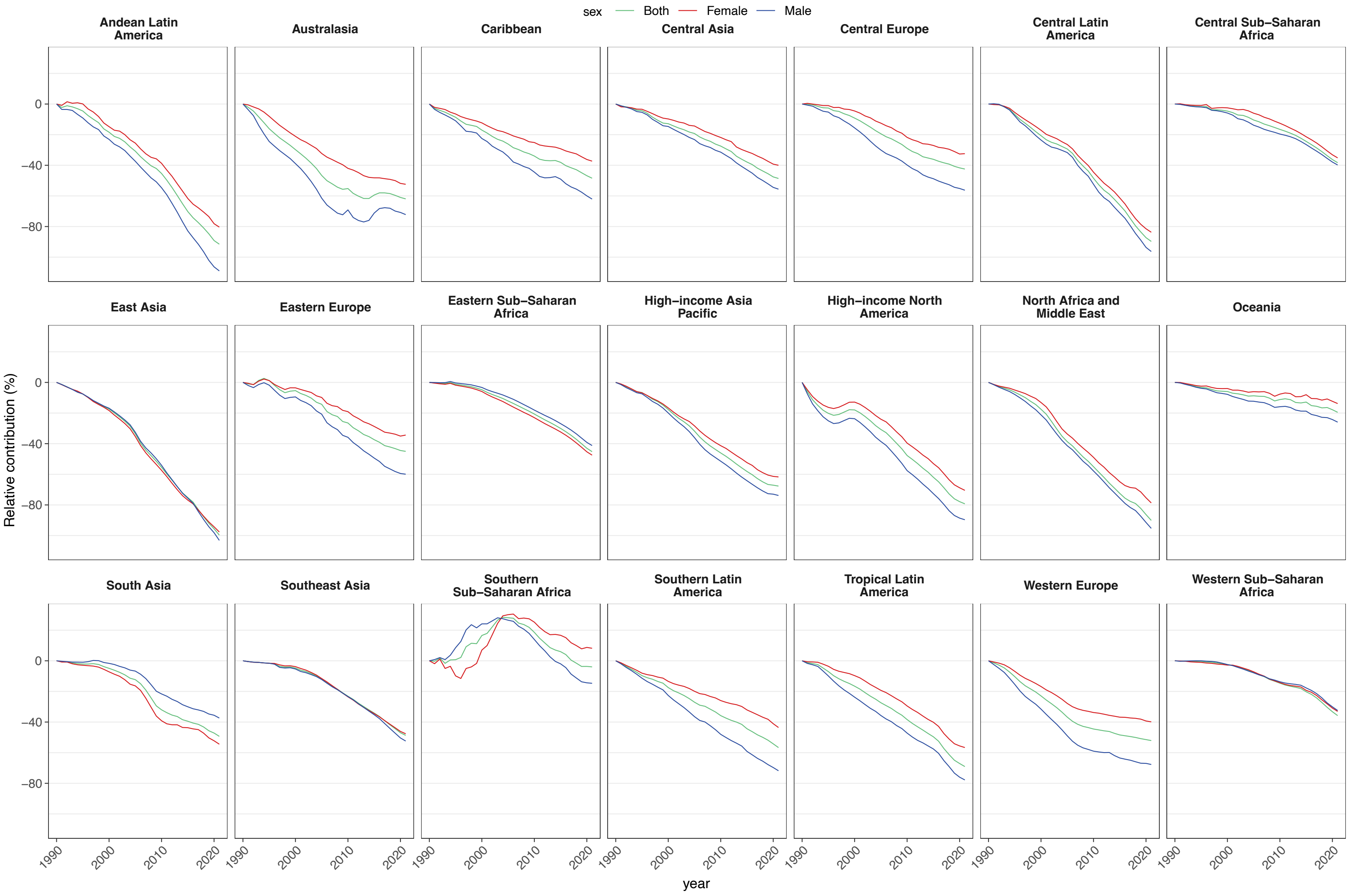

Figure S8. Changes in neoplasms-related DALYs attributable to disease severity between 1990 and 2021 at national levels.

Panel A. Absolute contribution for both sexes combined. **Panel B.** Relative contribution for both sexes combined. **Panel C.** Absolute contribution for male. **Panel D**. Relative contribution for male. **Panel E**. Absolute contribution for female. **Panel F**. Relative contribution for female.

Note: DALYs – disability-adjusted life-years.

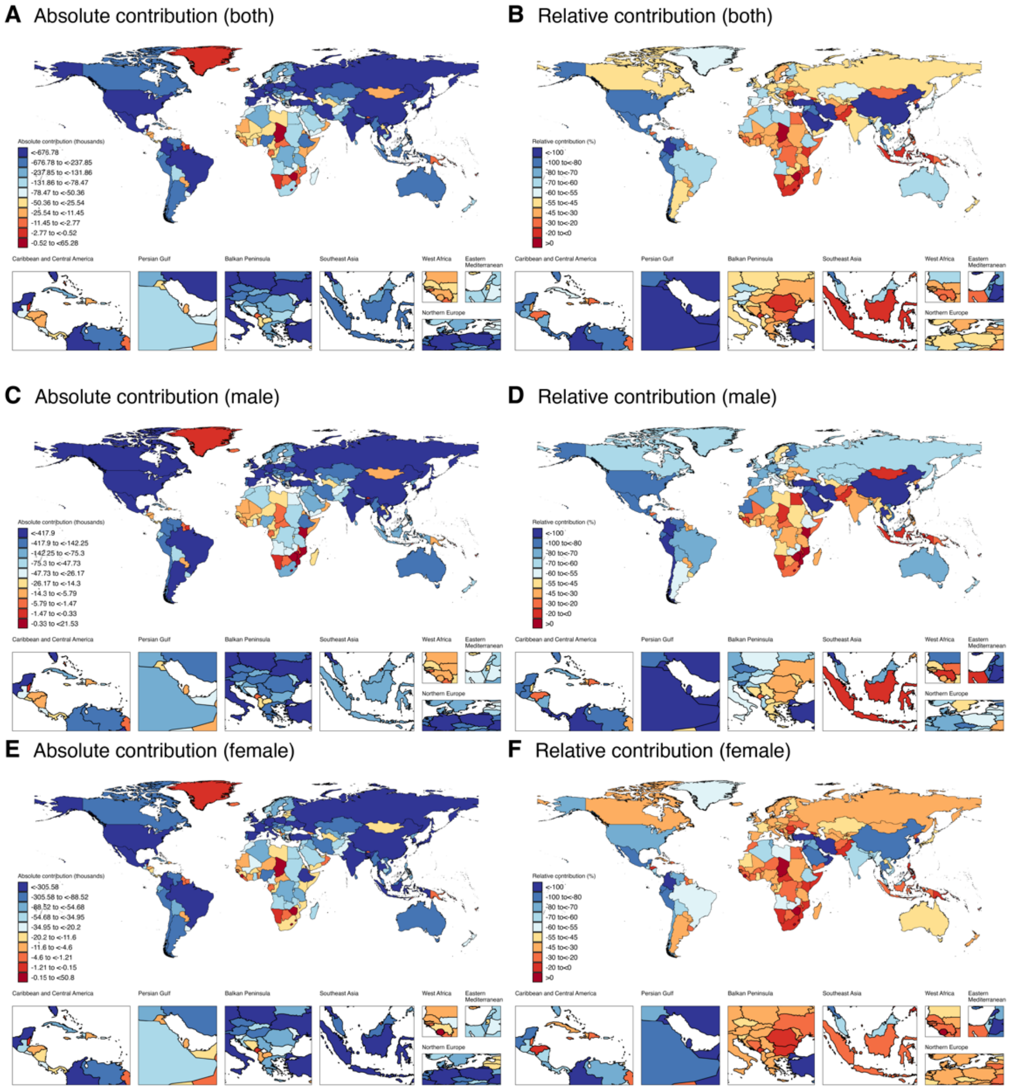

R codes

Code for decomposition analysis

The code below demonstrates the decomposition analysis of global DALY changes from 1990 to 2021, with d representing disease severity.

```
#Decomposition analysis####
Global_population_1990 <- population %>%
  dplyr::filter(location == a &
                  year == byear &
                  sex == b) %>%
  dplyr::mutate(age=factor(age,levels=ages,ordered = T)) %>%
  dplyr::arrange(age)
Global_1990 <- sum(Global_population_1990$val)
Global_population_1990$percent <- Global_population_1990$val/Global_1990
Global_population_2021 <- population %>%
  dplyr::filter(location == a &
                  year == compareyear &
```

```
                    sex == b)    %>%
    dplyr::mutate(age=factor(age,levels=ages,ordered = T)) %>%
    dplyr::arrange(age)
Global_2021 <- sum(Global_population_2021$val)
Global_population_2021$percent <- Global_population_2021$val/Global_2021
a_1990 <- Global_1990
b_1990 <- Global_population_1990$percent
a_2021 <- Global_2021
b_2021 <- Global_population_2021$percent
data_2021 <- subset(data,data$year==compareyear &
                        age %in% ages &
                        metric== 'Rate' &
                        sex== b &
                        cause == c &
                        location== a &
                        measure == 'Prevalence') %>%
```

```
    dplyr::mutate(age=factor(age,levels=ages,ordered = T)) %>% dplyr::arrange(age)
c_1990 <- as.numeric(data_1990$val)/10^5
c_2021 <- as.numeric(data_2021$val)/10^5
### servity
prevalence_1990 <- subset(data,year==byear &
                          age %in% ages &
                          metric== 'Number' &
                          sex== b &
                          cause == c &
                          location== a &
                          measure == 'Prevalence') %>%
    dplyr::mutate(age=factor(age,levels=ages,ordered = T)) %>% dplyr::arrange(age)

prevalence_2021 <- subset(data,year==compareyear &
                          age %in% ages &
                          metric== 'Number' &
```

```
                                        sex== b &
                                        location== a &
                                        cause == c &
                                        measure == 'Prevalence') %>%
    dplyr::mutate(age=factor(age,levels=ages,ordered = T)) %>% dplyr::arrange(age)
servity_1990 <- subset(data,year==byear &
                                    age %in% ages &
                                    metric== 'Number' &
                                    sex== b &
                                    location== a &
                                    cause == c &
                                    measure == d)
temp_age <- ages[!(ages %in% unique(servity_1990$age))]
temp <- data.frame(measure=rep(d,length(temp_age)),location=rep(a,length(temp_age)),
                   sex=rep(b,length(temp_age)),age=temp_age,cause=rep(c,length(temp_age)),
```

```
                              metric=rep('Rate',length(temp_age)),year =
rep(byear,length(temp_age)),val=rep(0,length(temp_age)),upper=rep(0,length(temp_age)),lower=rep(0,length(temp_age)))

servity_1990 <- servity_1990 %>% rbind(temp)

servity_1990 <-      servity_1990 %>%

  dplyr::left_join(prevalence_1990,by=c('location','sex','age','cause','year')) %>%

  dplyr::mutate(age=factor(age,levels=ages,ordered = T)) %>% dplyr::arrange(age) %>%

  dplyr::mutate(servity=val.x/val.y)

servity_2021 <- subset(data,data$year==compareyear &

                           age %in% ages &

                           metric== 'Number' &

                           sex== b &

                           location== a &

                           cause == c &

                           measure == d)

temp_age <- ages[!(ages %in% unique(servity_2021$age))]

temp <- data.frame(measure=rep(d,length(temp_age)),location=rep(a,length(temp_age)),
```

```
                                        sex=rep(b,length(temp_age)),age=temp_age,cause=rep(c,length(temp_age)),
                                        metric=rep('Rate',length(temp_age)),year =
rep(byear,length(temp_age)),val=rep(0,length(temp_age)),upper=rep(0,length(temp_age)),lower=rep(0,length(temp_age)))
servity_2021 <- servity_2021 %>% rbind(temp)
servity_2021 <-       servity_2021 %>%
    dplyr::left_join(prevalence_2021,by=c('location','sex','age','cause','year')) %>%
    dplyr::mutate(age=factor(age,levels=ages,ordered = T)) %>% dplyr::arrange(age) %>%
    dplyr::mutate(servity=val.x/val.y)
d_1990 <- servity_1990$servity
d_2021 <- servity_2021$servity

a_effect <- (sum(a_2021*b_2021*c_2021*d_2021) + sum(a_2021*b_1990*c_1990*d_1990))/4 +
    (sum(a_2021*b_1990*c_2021*d_2021) + sum(a_2021*b_2021*c_1990*d_2021) +
        sum(a_2021*b_2021*c_2021*d_1990) + sum(a_2021*b_1990*c_1990*d_2021) +
        sum(a_2021*b_1990*c_2021*d_1990) + sum(a_2021*b_2021*c_1990*d_1990))/12 -
    (sum(a_1990*b_2021*c_2021*d_2021) + sum(a_1990*b_1990*c_1990*d_1990))/4 -
```

```
    (sum(a_1990*b_1990*c_2021*d_2021) + sum(a_1990*b_2021*c_1990*d_2021) +
       sum(a_1990*b_2021*c_2021*d_1990) + sum(a_1990*b_1990*c_1990*d_2021) +
       sum(a_1990*b_1990*c_2021*d_1990) + sum(a_1990*b_2021*c_1990*d_1990))/12

b_effect <- (sum(b_2021*a_2021*c_2021*d_2021) + sum(b_2021*a_1990*c_1990*d_1990))/4 +
    (sum(b_2021*a_1990*c_2021*d_2021) + sum(b_2021*a_2021*c_1990*d_2021) +
       sum(b_2021*a_2021*c_2021*d_1990) + sum(b_2021*a_1990*c_1990*d_2021) +
       sum(b_2021*a_1990*c_2021*d_1990) + sum(b_2021*a_2021*c_1990*d_1990))/12 -
    (sum(b_1990*a_2021*c_2021*d_2021) + sum(b_1990*a_1990*c_1990*d_1990))/4 -
    (sum(b_1990*a_1990*c_2021*d_2021) + sum(b_1990*a_2021*c_1990*d_2021) +
       sum(b_1990*a_2021*c_2021*d_1990) + sum(b_1990*a_1990*c_1990*d_2021) +
       sum(b_1990*a_1990*c_2021*d_1990) + sum(b_1990*a_2021*c_1990*d_1990))/12

c_effect <- (sum(c_2021*b_2021*a_2021*d_2021) + sum(c_2021*b_1990*a_1990*d_1990))/4 +
    (sum(c_2021*b_1990*a_2021*d_2021) + sum(c_2021*b_2021*a_1990*d_2021) +
       sum(c_2021*b_2021*a_2021*d_1990) + sum(c_2021*b_1990*a_1990*d_2021) +
```

```
        sum(c_2021*b_1990*a_2021*d_1990) + sum(c_2021*b_2021*a_1990*d_1990))/12 -
    (sum(c_1990*b_2021*a_2021*d_2021) + sum(c_1990*b_1990*a_1990*d_1990))/4 -
    (sum(c_1990*b_1990*a_2021*d_2021) + sum(c_1990*b_2021*a_1990*d_2021) +
        sum(c_1990*b_2021*a_2021*d_1990) + sum(c_1990*b_1990*a_1990*d_2021) +
        sum(c_1990*b_1990*a_2021*d_1990) + sum(c_1990*b_2021*a_1990*d_1990))/12

d_effect <- (sum(d_2021*b_2021*c_2021*a_2021) + sum(d_2021*b_1990*c_1990*a_1990))/4 +
    (sum(d_2021*b_1990*c_2021*a_2021) + sum(d_2021*b_2021*c_1990*a_2021) +
        sum(d_2021*b_2021*c_2021*a_1990) + sum(d_2021*b_1990*c_1990*a_2021) +
        sum(d_2021*b_1990*c_2021*a_1990) + sum(d_2021*b_2021*c_1990*a_1990))/12 -
    (sum(d_1990*b_2021*c_2021*a_2021) + sum(d_1990*b_1990*c_1990*a_1990))/4 -
    (sum(d_1990*b_1990*c_2021*a_2021) + sum(d_1990*b_2021*c_1990*a_2021) +
        sum(d_1990*b_2021*c_2021*a_1990) + sum(d_1990*b_1990*c_1990*a_2021) +
        sum(d_1990*b_1990*c_2021*a_1990) + sum(d_1990*b_2021*c_1990*a_1990))/12
overll_differ <- a_effect + b_effect + c_effect + d_effect
```

Code for Figure 1

```
#Figure 1####

rm(list=ls())

library(tidyverse)

library(patchwork)

#global####

temp1 <- df1 |> filter(location=="Global")|> filter(sex%in%c('Male','Female'))

fig1 <- ggplot(data=temp1,aes(x=year,y=severity1,color=sex,fill=sex,shape=sex))+

  geom_line()+

  theme_bw()+

  scale_color_manual(values = c("Male" = '#002250', "Female" = "#c30001")) +

  scale_fill_manual(values = c("Male" = '#002250', "Female" ="#c30001")) +

  scale_y_continuous(limits = c(-58000000,0))+

  theme(

    legend.position =    c(0.175, 0.175),

    legend.justification = c(1, 1)) +
```

```
    theme(
        panel.grid.major     = element_blank(),
        panel.grid.minor     = element_blank()
    )+
    labs(x="year",y="Absolute contribution (thousands)",
              title = "Global level by sex")

fig2 <- ggplot(data=temp1,aes(x=year,y=severity2,color=sex,fill=sex,shape=sex))+
    geom_line()+
    theme_bw()+
    scale_color_manual(values = c("Male" = '#002250', "Female" = "#c30001")) +
    scale_fill_manual(values = c("Male" = '#002250', "Female" ="#c30001")) +
    scale_y_continuous(limits = c(-85, 1.5))+
    theme(
        legend.position =     c(0.175, 0.175),
        legend.justification = c(1, 1)) +
```

```
  theme(
    panel.grid.major    = element_blank(),
    panel.grid.minor    = element_blank()
  )+
  labs(x="year",y="Relative contributio (%)",
       title = "Global level by sex")

#SDI region####
location_1 <- c("High SDI"    ,"High-middle SDI","Middle SDI"    ,"Low-middle SDI" ,    "Low SDI" )
temp2 <- df1 |> filter(location%in%location_1) |> filter(sex=='Both')
SDI_color <- c('#50C9C3','#ffa036','#2A4C9F','#63BE7B','#672890')
temp2$location <- factor(temp2$location,levels = location_1,labels = location_1)
fig3 <- ggplot(data=temp2,aes(x=year,y=severity1,color=location,fill=location))+
  geom_line()+
  theme_bw()+
  scale_color_manual(values = SDI_color) +
```

```
    scale_fill_manual(values = SDI_color) +
    theme( legend.position =    c(0.25, 0.25),
                legend.justification = c(1, 1)) +
    theme(
        panel.grid.major    = element_blank(),
        panel.grid.minor    = element_blank()
    )+
    labs(x="year",y="Absolute contribution (thousands)",
            title = "For both sexes combined at SDI level")

fig4 <- ggplot(data=temp2,aes(x=year,y=severity2,color=location,fill=location))+
    geom_line()+
    theme_bw()+
    scale_color_manual(values = SDI_color) +
    scale_fill_manual(values = SDI_color) +
    scale_y_continuous(limits = c(-85, 1.5))+
```

```
    theme( legend.position =     c(0.25, 0.25),
                legend.justification = c(1, 1)) +
    theme(
        panel.grid.major     = element_blank(),
        panel.grid.minor     = element_blank()
    )+
    labs(x="year",y="Relative contributio (%)",
            title = "For both sexes combined at SDI level")

p <- fig1+fig2+fig3+fig4+plot_annotation(tag_levels = "A")+plot_layout(heights = c(5, 5))
```

Code for Figure 2

```
#Figure 2####
rm(list=ls())
library(tidyverse)
location1 <- c("Global","High SDI"     ,"High-middle SDI","Middle SDI"     ,"Low-middle SDI" ,     "Low SDI"       ,
```

```
                    "High-income North America", "Central Latin America","Andean Latin America","Caribbean" ,"Tropical Latin America","Southern Latin America" ,
                    "Western Europe" ,"Central Europe" ,"Eastern Europe" , "Central Asia"    ,    "East Asia" ,"South Asia" ,"Southeast Asia" ,
                    "High-income Asia Pacific","Australasia", "Oceania"    ,
                    "North Africa and Middle East" ,"Western Sub-Saharan Africa" ,"Central Sub-Saharan Africa"    ,"Eastern Sub-Saharan Africa","Southern Sub-Saharan Africa" )
cause_order <- c( "Nasopharynx cancer" ,"Larynx cancer","Lip and oral cavity cancer" ,"Thyroid cancer" ,
                        "Tracheal, bronchus, and lung cancer" ,"Mesothelioma",
                        "Esophageal cancer" ,"Stomach cancer" ,"Colon and rectum cancer" ,"Liver cancer" ,"Gallbladder and biliary tract cancer", "Pancreatic cancer" ,
                        "Kidney cancer","Bladder cancer" ,
                        "Prostate cancer" ,"Testicular cancer" ,
                        "Breast cancer","Ovarian cancer","Cervical cancer" ,"Uterine cancer" )
cause_order_lable <- c("NPC" ,"LarC","LOC" ,"ThyC" ,"TBLC" ,"Meso","EC" ,"SC" ,"CRC" ,"LivC" ,"GBTC", "PanC" ,"KC","BlaC" , "ProC" ,"TesC" , "BreC","OC","CC" ,"UC" )
data2021_ranked <- data2021 %>%
  group_by(location) %>%
```

```
    mutate(Rank = rank(-severity, ties.method = "first")) %>%
    ungroup()
data2021_ranked$cause <- factor(data2021_ranked$cause, levels = cause_order, labels = cause_order_lable)
data2021_ranked$location <- factor(data2021_ranked$location, levels = location1, labels = location1)
data2021_ranked <- data2021_ranked[order(data2021_ranked$location), ]
data2021_ranked$location <- factor(data2021_ranked$location, levels = location1, labels = location1)
data2021_ranked$Rank <- as.numeric(as.character(data2021_ranked$Rank))
max(data2021_ranked$severity)
min(data2021_ranked$severity)
p <- ggplot(data2021_ranked, aes(x = Rank, y = location, fill = pmin(pmax(severity, -150), 14))) +
    geom_tile(color = "white") +
    scale_fill_distiller(palette = "RdYlBu", direction = 1,
                                          breaks = c(-250, -200, -150, -100, -50, 0),
                                          labels = c(-250, -200, -150, -100, -50, 0),
                                          guide = guide_colourbar(
                                          title = "Relative contribution (proportion %)",
```

```
                                  title.position = "left",
                                  title.theme = element_text(size = 10),
                                  ticks = TRUE,
                                  nbin = 100,
                                  barwidth = 15,
                                  barheight = 1,
                                  breaks = c(-250,-200,-150,-100,-50,0),
                                  labels = c(-250,-200,-150,-100,-50,0))) +
geom_text(aes(label = paste0(cause, "\n", round(severity,2))), size = 3, color = "black") +
scale_y_discrete(limits = rev(levels(data2021_ranked$location))) +
labs(title = "DALYs attributable to disease severity changes from 1990 to 2021",
     x = "Rank of attributable proportions associated with disease severity changes",
     y = "Regions") +
theme_minimal(base_size = 12) +
scale_x_continuous(
  breaks = unique(data2021_ranked$Rank),
```

```
    labels = unique(data2021_ranked$Rank)
  ) +
  theme(axis.text.x = element_text(angle = 0, vjust = 0.5)) +
  theme(panel.grid = element_blank(),
        legend.position = "top")
```

Code for Figure 3

```
#Figure 3####
rm(list=ls())
library(tidyverse)
library(patchwork)
location1 <- c("Global","High SDI"     ,"High-middle SDI","Middle SDI"     ,"Low-middle SDI" ,     "Low SDI"       ,
               "High-income North America", "Central Latin America","Andean Latin America","Caribbean" ,"Tropical Latin America","Southern Latin America" ,
               "Western Europe" ,"Central Europe" ,"Eastern Europe" , "Central Asia"     ,     "East Asia" ,"South Asia" ,"Southeast Asia" ,
               "High-income Asia Pacific","Australasia", "Oceania"     ,
```

```
                    "North Africa and Middle East" ,"Western Sub-Saharan Africa" ,"Central Sub-Saharan Africa"     ,"Eastern Sub-Saharan Africa","Southern Sub-Saharan Africa" )

cause_order <- c( "Nasopharynx cancer" ,"Larynx cancer","Lip and oral cavity cancer" ,"Thyroid cancer" ,
                        "Tracheal, bronchus, and lung cancer" ,"Mesothelioma",
                        "Esophageal cancer" ,"Stomach cancer" ,"Colon and rectum cancer" ,"Liver cancer" ,"Gallbladder and biliary tract cancer", "Pancreatic cancer" ,
                        "Kidney cancer","Bladder cancer" ,
                        "Prostate cancer" ,"Testicular cancer" ,
                        "Breast cancer","Ovarian cancer","Cervical cancer" ,"Uterine cancer" )

cause_order_lable <- c("NPC" ,"LarC","LOC" ,"ThyC" ,"TBLC" ,"Meso","EC" ,"SC" ,"CRC" ,"LivC" ,"GBTC", "PanC" ,"KC","BlaC" , "ProC" ,"TesC" , "BreC","OC","CC" ,"UC" )

data2021_ranked <- data2021 %>%
  group_by(location) %>%
  mutate(Rank = rank(-severity, ties.method = "first")) %>%
  ungroup()

unique(data2021_ranked$cause)

#SDI####
```

```
location2 <- c("Global","High SDI"     ,"High-middle SDI","Middle SDI"     ,"Low-middle SDI" ,     "Low SDI"     )
df1 <- data2021 |>     filter(location%in%location2)
df1_ranked <- df1 %>%
    group_by(location) %>%
    mutate(Rank = rank(-severity, ties.method = "first")) %>%
    ungroup()
df1_ranked$cause <- factor(df1_ranked$cause, levels = cause_order, labels = cause_order_lable)
df1_ranked$location <- factor(df1_ranked$location, levels = location2, labels = location2)
df1_ranked <- df1_ranked[order(df1_ranked$location), ]
df1_ranked$location <- factor(df1_ranked$location, levels = location2, labels = location2)
df1_ranked$Rank <- as.numeric(as.character(df1_ranked$Rank))
max(df1_ranked$severity)
min(df1_ranked$severity)
p_SDI <- ggplot(df1_ranked, aes(x = Rank, y = location, fill = pmin(pmax(severity, -1000000), 1000))) +
    geom_tile(color = "white") +
    scale_fill_distiller(palette = "RdYlBu", direction = 1,
```

```
                    breaks = c(-1000000,-500000,0,1500),
                    labels = c(-1000,-500,0,1.5),
                    guide = guide_colourbar(
                    title = "Absolute contribution (thousands)",
                    title.position = "left",
                    title.theme = element_text(size = 10),
                    ticks = TRUE,
                    nbin = 100,
                    barwidth = 15,
                    barheight = 1,
                    breaks =    c(-1000000,-500000,0,1500),
                    labels =    c(-1000,-500,0,1.5))) +
geom_text(aes(label = paste0(cause, "\n", round(severity/1000,2))), size = 3, color = "black") +
scale_y_discrete(limits = rev(levels(df1_ranked$location))) +
labs(title = "DALYs attributable to disease severity changes at global and SDI levels",
     x = "Rank of attributable proportions associated with disease severity changes",
```

```
            y = "Global and SDI Regions") +
    theme_minimal(base_size = 12) +
    scale_x_continuous(
        breaks = unique(df1_ranked$Rank),
        labels = unique(df1_ranked$Rank)
    ) +
    theme(axis.text.x = element_text(angle = 0, vjust = 0.5)) +
    theme(panel.grid = element_blank(),
              legend.position = "top")
p_SDI

#21GBD####
location3 <- c("High-income North America", "Central Latin America","Andean Latin America","Caribbean" ,"Tropical Latin America","Southern Latin America" ,
                        "Western Europe" ,"Central Europe" ,"Eastern Europe" , "Central Asia"     ,     "East Asia" ,"South Asia" ,"Southeast Asia" ,
                        "High-income Asia Pacific","Australasia", "Oceania"     ,
```

```
                         "North Africa and Middle East" ,"Western Sub-Saharan Africa" ,"Central Sub-Saharan Africa"    ,"Eastern Sub-Saharan Africa","Southern Sub-Saharan Africa" )

df3 <- data2021 |>    filter(location%in%location3)

df3_ranked <- df3 %>%

   group_by(location) %>%

   mutate(Rank = rank(-severity, ties.method = "first")) %>%

   ungroup()

df3_ranked$cause <- factor(df3_ranked$cause, levels = cause_order, labels = cause_order_lable)

df3_ranked$location <- factor(df3_ranked$location, levels = location3, labels = location3)

df3_ranked <- df3_ranked[order(df3_ranked$location), ]

df3_ranked$location <- factor(df3_ranked$location, levels = location3, labels = location3)

df3_ranked$Rank <- as.numeric(as.character(df3_ranked$Rank))

breaks_values <- c(-250,-200,-150,-100,-50,0)

p_21 <- ggplot(df3_ranked, aes(x = Rank, y = location, fill = pmin(pmax(severity, -100000), 1000))) +

   geom_tile(color = "white") +

   scale_fill_distiller(palette = "RdYlBu", direction = 1,
```

```
                    breaks = c(-100000,-50000,0,1500),
                    labels = c(-100,-50,0,1.5),
                    guide = guide_colourbar(
                    title = "Absolute contribution (thousands)",
                    title.position = "left",
                    title.theme = element_text(size = 10),
                    ticks = TRUE,
                    nbin = 100,
                    barwidth = 15,
                    barheight = 1,
                    breaks = c(-100000,-50000,0,1500),
                    labels = c(-100,-50,0,1.5))) +
geom_text(aes(label = paste0(cause, "\n", round(severity/1000,2))), size = 3, color = "black") +
scale_y_discrete(limits = rev(levels(df3_ranked$location))) +
labs(title = "DALYs attributable to disease severity changes at GBD regional level",
     x = "Rank of attributable proportions associated with disease severity changes",
```

```
        y = "GBD Regions") +
  theme_minimal(base_size = 12) +
  scale_x_continuous(
    breaks = unique(df3_ranked$Rank),
    labels = unique(df3_ranked$Rank)
  ) +
  theme(axis.text.x = element_text(angle = 0, vjust = 0.5)) +
  theme(panel.grid = element_blank(),
        legend.position = "top")
p_21
fig <- p_SDI+p_21+plot_annotation(tag_levels = "A")+plot_layout(ncol = 1,heights = c(1, 4))
```

Code for Figure 4

```
#Figure 4####
rm(list=ls())
library(tidyverse)
```

```
library(patchwork)
library(RColorBrewer)
library(sf)
fig1 <- data |>
  filter(year == 2021) |>
  filter(sex == "Both") |>
  ggplot() +
  geom_sf(aes(group = NAME, fill = severity1))+
  scale_fill_brewer(palette = "RdYlBu", direction = -1)
ggsave("figure_4A.tiff", fig1, width = 12, height = 8.5, dpi = 600)

fig2 <- data |>
  filter(year == 2021) |>
  filter(sex == "Both") |>
  ggplot() +
  geom_sf(aes(group = NAME, fill = severity2))+
```

```
    scale_fill_brewer(palette = "RdYlBu", direction = -1)
ggsave("figure_4B.tiff", fig2, width = 12, height = 8.5, dpi = 600)

fig3 <- data |>
    filter(year == 2021) |>
    filter(sex == "Male") |>
    ggplot() +
    geom_sf(aes(group = NAME, fill = severity1))+
    scale_fill_brewer(palette = "RdYlBu", direction = -1)
ggsave("figure_4C.tiff", fig3, width = 12, height = 8.5, dpi = 600)

fig4 <- data |>
    filter(year == 2021) |>
    filter(sex == "Male") |>
    ggplot() +
    geom_sf(aes(group = NAME, fill = severity2))+
```

```
    scale_fill_brewer(palette = "RdYlBu", direction = -1)
ggsave("figure_4D.tiff", fig4, width = 12, height = 8.5, dpi = 600)

fig5 <- data |>
    filter(year == 2021) |>
    filter(sex == "Female") |>
    ggplot() +
    geom_sf(aes(group = NAME, fill = severity1))+
    scale_fill_brewer(palette = "RdYlBu", direction = -1)
ggsave("figure_4E.tiff", fig5, width = 12, height = 8.5, dpi = 600)

fig6 <- data |>
    filter(year == 2021) |>
    filter(sex == "Female") |>
    ggplot() +
    geom_sf(aes(group = NAME, fill = severity2))+
```

```
    scale_fill_brewer(palette = "RdYlBu", direction = -1)
ggsave("figure_4F.tiff", fig6, width = 12, height = 8.5, dpi = 600)
```
